# A Versatile “Synthesis Tag” (SynTag) for the Chemical Synthesis of Aggregating Peptides and Proteins

**DOI:** 10.1021/jacs.4c14247

**Published:** 2024-12-06

**Authors:** Héloïse Bürgisser, Elyse T. Williams, Aliénor Jeandin, Robin Lescure, Adhvitha Premanand, Songlin Wang, Nina Hartrampf

**Affiliations:** † Department of Chemistry, University of Zurich, Winterthurerstrasse 190, Zurich 8057, Switzerland; ‡ National Magnetic Resonance Facility at Madison (NMRFAM), University of Wisconsin–Madison, Madison, Wisconsin 53706, United States

## Abstract

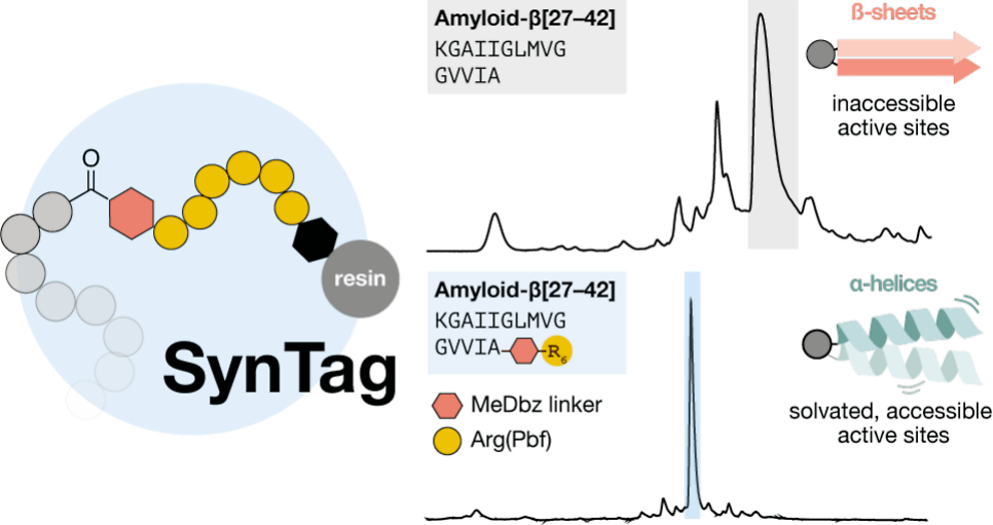

Solid-phase peptide synthesis (SPPS) and native chemical ligation (NCL) are powerful methods for obtaining peptides and proteins that are otherwise inaccessible. Nonetheless, numerous sequences are difficult to prepare via SPPS, and cleaved peptides often have low aqueous solubility. To address these challenges, we developed a “Synthesis Tag” consisting of six arginines connected to the target sequence via a cleavable MeDbz linker. “SynTag” effectively improves batch- and flow-SPPS of “difficult sequences”, enhances the solubility of the cleaved peptides, and provides direct access to native sequences by hydrolysis, or peptide thioesters for NCL. We demonstrate its utility in the first chemical synthesis of the MYC transactivation domain with a single NCL. We envisage SynTag to become a broadly applicable tool that enables the synthesis and study of previously unattainable peptides and proteins.

## Introduction

**Figure 1:**
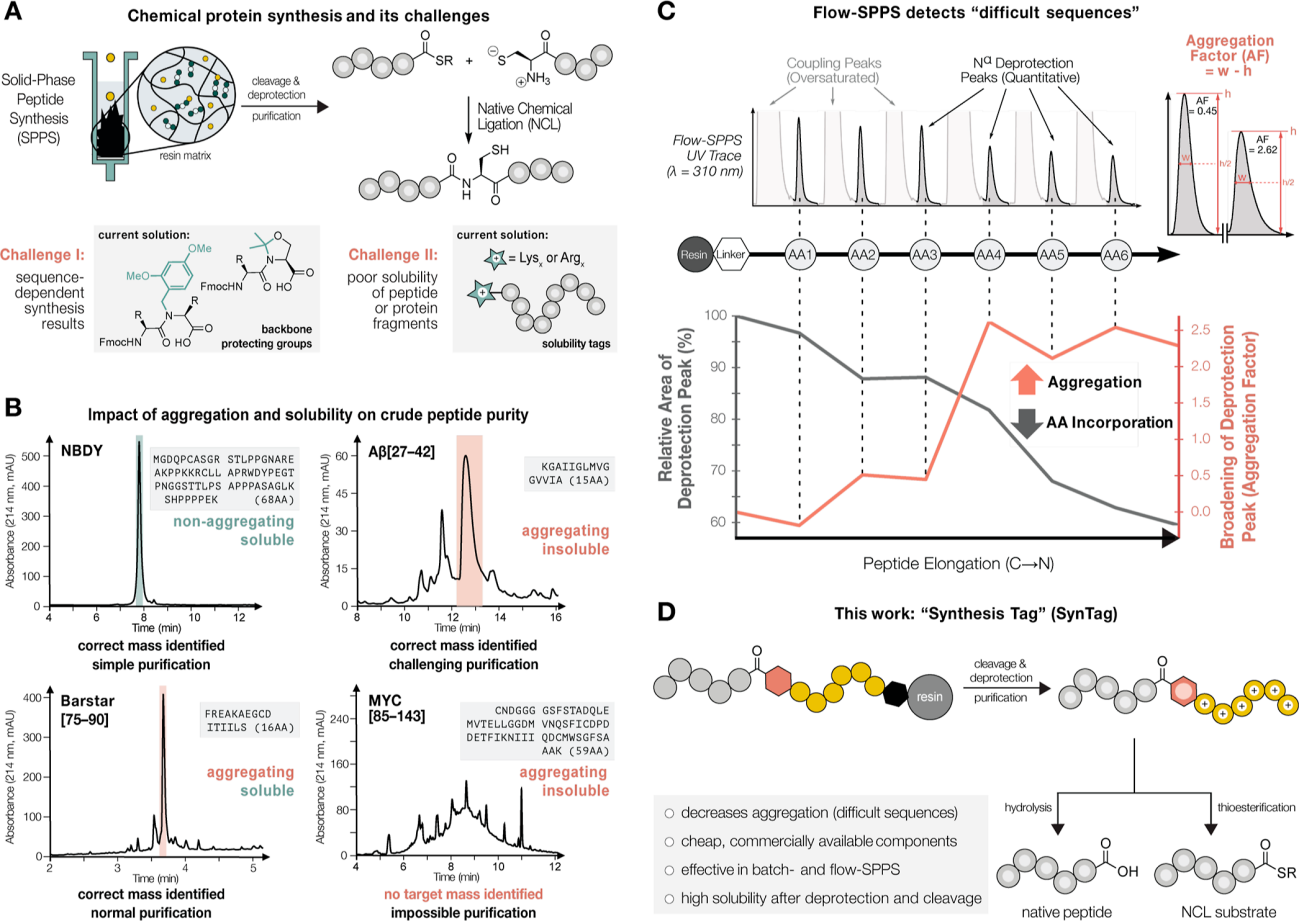
**Flow-based solid-phase peptide synthesis enabled the development of a broadly applicable synthesis tag (SynTag), that simultaneously improves peptide crude purities and solubilities.** (A) Chemical protein synthesis often consists of two steps: stepwise SPPS followed by native chemical ligation. Major challenges are (I) sequence dependence of SPPS, often framed as “difficult peptides” (aggregation), associated with low yield and purity, and (II) low solubility of final peptide fragments for HPLC and NCL. (B) Impact of aggregation and solubility on crude peptide analysis (UHPLC) displayed for four cases: NBDY, Barstar[75–90], Aβ [27–42] and MYC[85–143]. While aggregation leads to increased side products, low solubility leads to characteristic UHPLC peak broadening. Note that only small amounts of Aβ [27–42] could be loaded onto the UHPLC column to minimize further peak broadening, resulting in comparatively low absorbance. (C) Flow-based peptide synthesis allows for in-line UV–vis monitoring and the detection of aggregation through shape change of Fmoc-deprotection peaks. Upon aggregation, the resin starts to agglomerate, reducing coupling efficiencies and impacting crude purities. (D) This work: a versatile SynTag that reduces aggregation during SPPS, improves crude peptide solubility, and delivers either native peptides or substrates for NCL.

Chemical synthesis enables the production of uniquely modified peptides and proteins for application in chemical biology and medicinal chemistry.^1^ Unlike recombinant expression, this approach enables the incorporation of a theoretically unlimited number of noncanonical amino acids, including post-translational modifications (PTMs), functional handles, and backbone modifications into the sequence. Solid-phase peptide synthesis (SPPS) is a general method to prepare target peptides,^2^ as well as fragments that can be combined using native chemical ligation (NCL) to afford large proteins.^3^ Recent advances have increased the length of peptides that can be synthesized,^4^ and several elegant ligation strategies have been developed for chemical protein synthesis.^5,6^ However, two major challenges remain: (I) aggregation of the growing peptide chain during SPPS, occurring in >40% of all syntheses,^7^ and (II) poor solubility of the cleaved peptide fragments, impeding HPLC purification and ligation in aqueous buffers (Figure 1A).^8,9^

Aggregation during SPPS was already noted in the early days of peptide synthesis by Sheppard, Kent, Milton, Mutter, and others,^10−13^ and has impeded the study of many biologically relevant peptides and proteins (Figure 1A, challenge I).^14,15^ In SPPS, a cleavable linker is connected to a solid support (resin), upon which iterative amino acid couplings and deprotections enable the stepwise assembly of the desired peptide chain.^2^ Aggregation of the growing sequence occurs through sequence-dependent inter- and intrachain interactions, reducing the efficiency of subsequent couplings and deprotections (Figure 1B).^16,17^ Among the few methods that allow aggregation monitoring in SPPS, flow-SPPS—such as automated fast-flow peptide synthesis (AFPS)^4,18^—provides the unique opportunity for time-resolved, in-line UV–vis monitoring of Fmoc deprotection steps, enabling detection in real time (Figure 1C).^19^ For the entire duration of aggregation, the deprotection peaks broaden due to reduced mass transfer and reaction efficiency.^7^ Several approaches have been developed to address aggregation,^20−23^ with backbone protecting groups (e.g., pseudoproline building blocks) being the most popular.^13,14^ However, such strategies must be tailored to specific peptide sequences, as there is currently no universal tool to suppress aggregation during SPPS. After elongation by SPPS, the peptides are globally deprotected and cleaved from the resin for subsequent solution-phase steps (e.g., HPLC purification and NCL). However, poor solubility of the cleaved peptides poses a second major challenge in chemical protein synthesis (Figure 1A, challenge II).^8,9,24^ As opposed to the unsolved challenge of aggregation in SPPS, peptide solubility has been addressed with solubility tags.^8,15,25−39^ These usually consist of positively charged amino acids (lysine, arginine) linked to the peptide covalently or by a cleavable linker at amino acid side-chains, N- or C-termini. Peptide tags (e.g., polyhistidine and FLAG tags) are also often used as affinity purification handles or recognition sequences in chemical protein synthesis and recombinant expression.^40−42^ However, generally applicable and cleavable tags have never been applied to improve peptide assembly by SPPS.

Here, we set out to develop a “Synthesis Tag” (SynTag) that can be used routinely in SPPS as an aggregation suppressor and solubility enhancer (Figure 1D). Such a tool would significantly reduce the need for tedious synthesis optimization. To ensure the practicality of our strategy, we focused our investigation on the use of commercially available linkers and amino acid building blocks. Additionally, we sought an orthogonally cleavable linker connecting the target sequence to the synthesis tag. To our surprise, the most effective combination of amino acid tag and linker (“SynTag”) consisted of six Arg(Pbf) residues and a MeDbz linker,^43^ which not only successfully improved synthesis results but also the solubility of cleaved and unprotected peptides. Notably, while tags have been used to improve peptide solubility, their impact on peptide synthesis itself has never been investigated.^8,15,25−39^ Finally, the use of SynTag for NCL was demonstrated in the synthesis of the transactivation domain (TAD) of the oncogenic protein MYC (143 AA) using a single ligation step. Importantly, this synthesis also marks the first reported combination of AFPS and NCL.

## Results and Discussion

### Adding a C-Terminal Amino Acid Sequence Drastically Alters Synthesis Results

**Figure 2:**
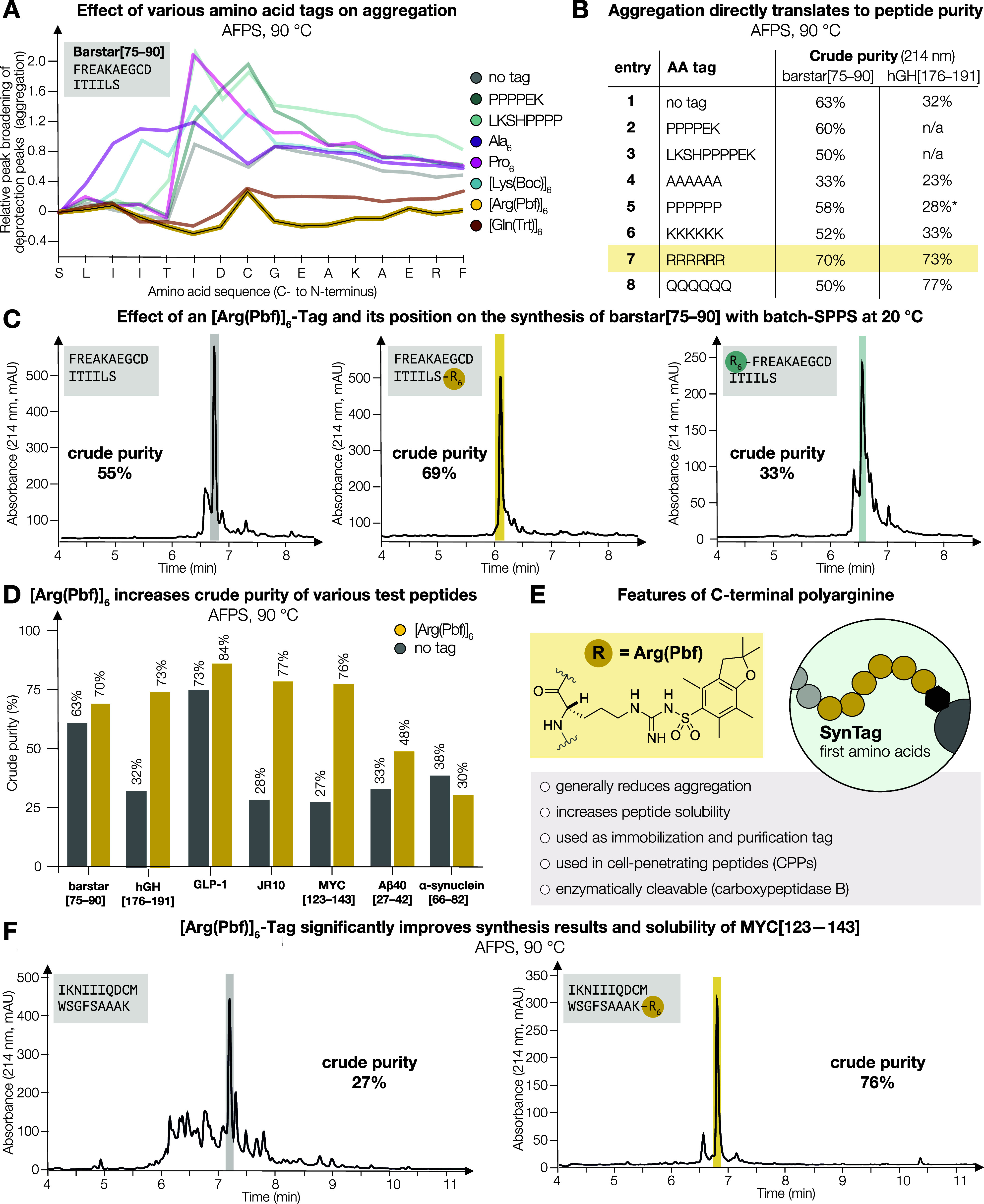
**A C-terminal sequence of six Arg(Pbf) decreases aggregation in flow- and batch-SPPS.** (A) Aggregation as a function of Fmoc deprotection peak broadening by in-line UV–vis (310 nm) in flow-SPPS for various amino acid tags. The first amino acids coupled alter the severity and onset of aggregation for Barstar[75–90]. All peptides were synthesized by AFPS at 90 °C. (B) Aggregation detected by in-line UV–vis directly translates to crude purity of cleaved and deprotected Barstar[75–90] and hGH[176–191]F176Y (abbreviated as hGH[176–191]). Crude purity was determined via UHPLC (214 nm). (C) Adding C-terminal, but not N-terminal [Arg(Pbf)]_6_ improves crude peptide purity of Barstar[75–90] synthesized by batch-SPPS at room temperature. (D) C-terminal [Arg(Pbf)]_6_ reduces aggregation in various aggregating test peptides as determined by UHPLC (214 nm). These peptides were synthesized by AFPS at 90 °C. (E) C-terminal [Arg(Pbf)]_6_ not only decreases aggregation and improves peptide crude purity, but also affords the positively charged “free” Arg_6_ after cleavage from the resin and global deprotection, which provides several advantageous features. (F) The C-terminal [Arg(Pbf)]_6_-tag improves crude purity of MYC[123–143] 3-fold, potentially enabling the synthesis of longer MYC fragments. *Two peaks with the same mass were observed for hGH[176–191]F176Y -Pro_6_.

We first investigated if additional amino acids coupled prior to the target peptide sequence could reduce aggregation during flow-SPPS.^44,45^ For this purpose, we chose the short and severely aggregating peptide Barstar[75–90] as a test peptide. Synthesis by AFPS upon a polyethylene glycol (PEG) resin with Rink amide linker (without any amino acid tags), showed severe aggregation starting after the coupling of Thr[86], giving a final crude purity of 63% (Figure 2A,B, entry 1). We then investigated the impact of two sequences (PPPPEK and LKSHPPPPEK) from the nonaggregating protein NBDY (Figure 1B),^46^ speculating that they might decrease Barstar[75–90] aggregation. Surprisingly, both NBDY sequences increased aggregation, leading to decreased crude purity (Figure 2A,B, entries 2&3). Ala_6_, in line with literature reports, also led to increased aggregation and low peptide purity (entry 4).^47,48^ Unexpectedly, Pro_6_—despite lacking the ability to form H-bonds^45^—did not reduce aggregation in the synthesis of Barstar[75–90] (entry 5). Next, [Lys(Boc)]_6_ (entry 6) and [Arg(Pbf)]_6_ (entry 7) were investigated, which would also provide positively charged side chains after cleavage and global deprotection. While [Lys(Boc)]_6_ increased aggregation, [Arg(Pbf)]_6_ effectively suppressed it, resulting in an improved crude purity of 70%. Six Arg(Pbf) residues were found to be the optimum tag length (Supporting Information Figure S133).

Intrigued by these results, we wondered if side chain and protecting group bulkiness could be responsible for the reduced aggregation, and therefore investigated [Gln(Trt)]_6_. Interestingly, this tag also reduced aggregation in Barstar[75–90] synthesis, albeit to a smaller extent than [Arg(Pbf)]_6_ (Figure 2A,B, entry 8). To further investigate the impact of side-chain protecting groups on aggregation, we studied lysine due to the larger variety of available side-chain protecting groups and modifications compared to arginine. As C-terminal tags for Barstar[75–90] (see Supporting Information Section S3.13), [Lys(Boc)]_6_ had the earliest onset of aggregation and resulted in the lowest crude yield and purity, [Lys(Trt)]_6_ delayed aggregation and [Lys(Ac)]_6_ successfully mitigated it and improved the crude purity. This supported the notion that side chain protecting groups play an important role in secondary structure formation during SPPS.

To affirm our results and exclude Barstar[75–90]-specific effects, we carried out a similar screening with human growth hormone (hGH[176–191]F176Y), a known difficult peptide that could previously only be synthesized by introducing several pseudoprolines (Figure 2B).^14^ As with Barstar[75–90], we observed that C-terminal [Lys(Boc)]_6_ and Ala_6_ tags caused an earlier onset of aggregation. Similarly, Pro_6_ did not reduce aggregation while [Gln(Trt)]_6_ and [Arg(Pbf)]_6_ tags effectively mitigated aggregation with a remarkably increased purity, from 32% up to 73% in the latter case (see Supporting Information Figure S59).

We next explored the temperature and method dependence (i.e., batch-SPPS vs AFPS) of aggregation suppression by [Arg(Pbf)]_6_. AFPS is usually carried out at 90 °C to achieve rapid coupling, while commercial synthesizers typically operate at 20–70 °C. We therefore investigated the synthesis of Barstar[75–90] using AFPS at 70 °C and observed an even stronger impact of [Arg(Pbf)]_6_ on peptide crude purity (no tag: 38% vs [Arg(Pbf)]_6_: 61%; see Supporting Information Figure S132). Encouraged by these results, we explored the use of [Arg(Pbf)]_6_ for batch-SPPS at room temperature, where aggregation usually has the largest impact (Figure 2C). Two test peptides, Barstar[75–90] and hGH[176–191]F(176)Y were therefore synthesized with and without [Arg(Pbf)]_6_ using standard batch-SPPS protocols (5 eq. Fmoc-amino acid, HATU), with a striking effect: For Barstar[75–90], inclusion of C-terminal [Arg(Pbf)]_6_ significantly improved crude purity from 55% to 69% (Figure 2C). To exclude increased solubility of the cleaved peptide as possible reason, we also tested the effect of [Arg(Pbf)]_6_ at the N-terminus of the peptide, which led to a lower purity of 33%. Similar observations were made for hGH[176–191]F(176)Y, where crude purity increased from 16% to 32% when the [Arg(Pbf)]_6_ tag was added to the C-terminus, and decreased to 13% when the tag was added to the N-terminus. These results indicate that [Arg(Pbf)]_6_ not only impacts the solubility of the cleaved peptide, but facilitates the subsequent synthesis (Supporting Information Figure S121).

To determine if [Arg(Pbf)]_6_ could broadly reduce aggregation during peptide synthesis, we evaluated its application to a panel of difficult sequences; GLP-1, JR-10, MYC[123–143], Aβ42[27–42], and α-synuclein[66–82] (Figure 2D). For GLP-1, the introduction of [Arg(Pbf)]_6_ successfully delayed the onset of aggregation and thereby increased overall crude purity from 73% to 84% (see Supporting Information Section S3.2). JR-10, another literature-known difficult sequence,^49^ showed drastically decreased aggregation and a purity improvement from 28% to 77% (see Supporting Information Section S3.4). Similarly, MYC[123–143] also showed an improvement in crude purity from 27% to 76% (see Figure 2F and Supporting Information Section S3.5). For Aβ42[27–42], we noticed a delayed onset of aggregation which resulted in an improvement of crude purity from 33% to 48%, accompanied by enhanced solubility (see Supporting Information Section S3.7). However, aggregation remained challenging in α-synuclein[66–82] synthesis (no tag: 38% vs [Arg(Pbf)]_6_: 30% crude purity, see Supporting Information Section S3.8.

In summary, most syntheses showed reduced aggregation upon introduction of C-terminal [Arg(Pbf)]_6_ and resulted in higher crude purities. In a next step, we wanted to gain insight into how [Arg(Pbf)]_6_ suppresses aggregation. Hypothesizing that the tag disrupts β-sheet formation, we employed infrared spectroscopy (IR) and solid-state NMR (SSNMR) of resin-bound peptides to determine possible changes in secondary structure and flexibility.

Analysis of resin-bound peptides shows a correlation between on-resin aggregation, β-sheet formation, and dynamics. In contrast to other techniques such as X-ray crystallography or circular dichroism, IR spectroscopy requires little to no sample preparation, and in the case of polypeptides, has been historically used for secondary structure determination in solution and in the solid state.^50−55^ The amide I (1700–1600 cm^–1^, average 1652 cm^–1^) and amide II region (1580–1510 cm^–1^, average 1540 cm^–1^) show consistent values for α-helices.^53−55^ In the case of β-sheets, the main component of amide I region is found at an average frequency of 1629 cm^–1^ and the secondary frequency at 1696 cm^–1^.^53−55^

**Figure 3:**
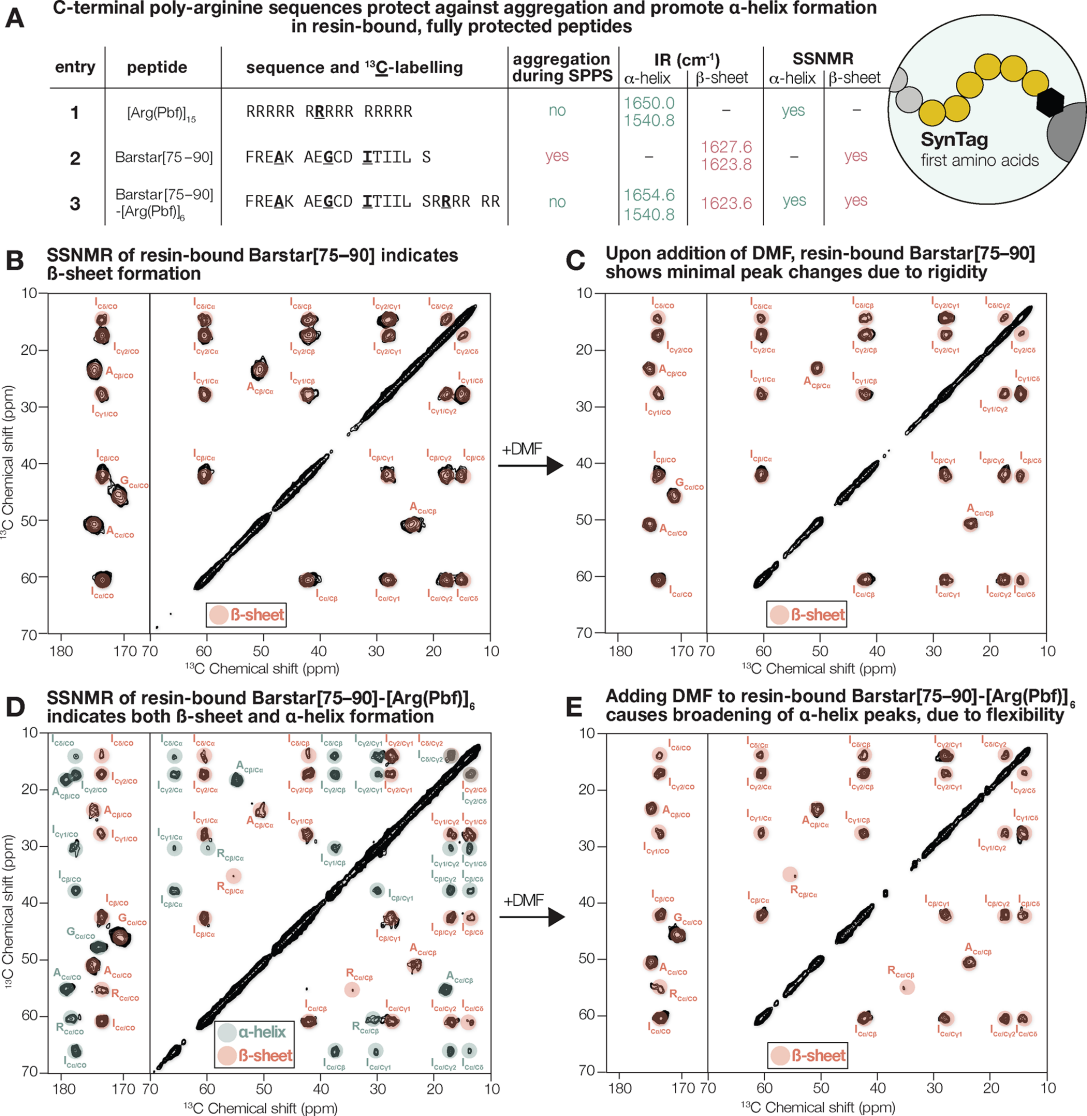
**IR and SSNMR experiments indicate that [Arg(Pbf)]_6_ changes the structure of difficult peptides on resin from rigid β-sheets to flexible α-helices.** (A) IR and SSNMR analysis indicates a correlation between secondary peptide structure and aggregation during SPPS. (B) Resin-bound and fully protected Barstar[75–90] (no tag) shows only β-sheet SSNMR signals. (C) Upon solvation with DMF, the β-sheet signals remain unchanged. (D) SSNMR spectrum of resin-bound and fully protected Barstar[75–90]-[Arg(Pbf)]_6_ shows both α-helix and β-sheet signals for all ^13^C-labeled residues. (E) Upon solvation with DMF, the α-helix signals disappear, yet the β-sheet signals remain.

To determine the effect of [Arg(Pbf)] on the conformation of Barstar[75–90] (on resin), we analyzed three resin-bound, protected peptides by IR: [Arg(Pbf)]_15_, as well as Barstar[75–90] with and without C-terminal [Arg(Pbf)]_6_. For [Arg(Pbf)]_15_, bands at 1655 and 1542 cm^–1^ suggested an α-helix conformation, while IR analysis of Barstar[75–90] (no tag) showed bands at 1628 and 1624 cm^–1^, indicating exclusive β-sheet conformation. The Barstar[75–90]-[Arg(Pbf)]_6_ peptide showed both α-helix bands at 1655 and 1541 cm^–1^ and a β-sheet band at 1624 cm^–1^, suggesting the simultaneous presence of both conformations (Figure 3A, Supporting Information Section S4). Even if the limited resolution of IR precludes discrimination between conformations existing within a peptide sequence and the different conformations of single peptide strands, these observations pointed toward a structural influence of the [Arg(Pbf)]_6_ tag, shifting the aggregation-prone β-strand conformation of Barstar[75–90] to a mixed α-helix/β-sheet conformation.

Intrigued by these findings, we then employed SSNMR to elucidate the structure of individual resin-bound peptides, both with and without the [Arg(Pbf)]_6_ tag. We used Barstar[75–90] as a model peptide with ^13^C-labeling at Ala[78], Gly[82], Ile[85], and Arg[92] of the [Arg(Pbf)]_6_ tag to investigate the secondary structure of the resin-bound peptide. First, a “dry” sample (i.e., without DMF solvation) of Barstar[75–90] (no tag) was measured by SSNMR spectroscopy (Figure 3B), and gave a single set of resonance peaks for all labeled residues, indicating the presence of a single conformation. Secondary chemical shift analysis suggests that untagged Barstar[75–90] adopts a β-strand structure throughout the peptide chain (Supporting Information Table S2). In contrast, SSNMR measurement of resin-bound Barstar[75–90]-[Arg(Pbf)]_6_ under “dry” conditions shows two sets of resonance peaks for all labeled residues (Figure 3D). Secondary chemical shift analysis indicates the coexistence of both α-helix and β-strand conformations (Supporting Information Tables S2 and S3), in an approximately 1:2 ratio in resin-bound Barstar[75–90]-[Arg(Pbf)]_6_ (Supporting Information Table S5).

Furthermore, the dynamics of the resin-bound peptides were uncovered using SSNMR. Cross-polarization-based SSNMR relies on dipolar couplings between ^1^H and ^13^C nuclei for polarization transfer. Since these dipolar couplings are orientation-dependent, rapid and random molecular motion can significantly suppress them, causing SSNMR signals from more flexible regions of the peptides to weaken or even disappear from the spectrum. Upon adding DMF, the SSNMR signals from the resin-bound Barstar[75–90] (no tag) persist after resolvation (Figure 3C), indicating that the β-strand component remains highly rigid. For resin-bound Barstar[75–90]-[Arg(Pbf)]_6_, the SSNMR signals from the β-strand component also remain visible after DMF addition (Figure 3E), similar to untagged Barstar[75–90]. However, the disappearance of resonance peaks corresponding to the α-helix component implies that the α-helix becomes flexible in the presence of DMF (Figure 3E).

As a control, [Arg(Pbf)]_15_ with ^13^C-labeling at Arg[7] was prepared by batch SPPS. The SSNMR spectrum of resin-bound [Arg(Pbf)]_15_ under “dry” conditions suggests an exclusively α-helical structure (Supporting Information Table S4). Upon adding DMF, the SSNMR signal disappeared (Figure S155), indicating that the α-helix becomes flexible upon resolvation and also confirming that loss of α-helix signal is due to dynamic motion rather than a DMF-induced conformational transition.

In summary, the combination of multiple analytical techniques was key to elucidate the interplay of β-strand formation, peptide dynamics and ultimately on-resin aggregation. This supports the notion that aggregation and rigid β-strand formation decrease reaction kinetics and hinder the access of deprotection and coupling agents to reaction sites, thereby reducing synthesis efficiency.^56^ The [Arg(Pbf)]_6_ tag induces the formation of a flexible α-helix component, exposing the reaction sites to the reagents and thereby facilitating peptide assembly. Importantly, a limitation of both IR and SSNMR is that both analytical experiments were performed at much lower temperatures than synthesis on AFPS (IR = 23 °C, SSNMR = 10 °C, AFPS synthesis = 90 °C). Aggregation is highly temperature-dependent and we therefore reason that a combination of [Arg(Pbf)]_6_ and higher temperature overcomes aggregation for Barstar[75–90], but it persists for α-synuclein[66–82]. While IR and SSNMR give valuable insights into structural changes with and without [Arg(Pbf)]_6_-tag—especially for synthesis at room temperature—the temperature difference to AFPS must be considered when assessing aggregation quantitatively.

While [Arg(Pbf)]_6_ tagging proved to be a straightforward and powerful method to suppress aggregation and enhance solubility, removing the tag would require enzymatic cleavage using carboxypeptidase B.^57^ We reasoned that attaching the [Arg(Pbf)]_6_ tag to the peptide via an orthogonal, cleavable, and nonaggregation-promoting linker could mitigate this issue. We, therefore, next explored linkers to ultimately design a versatile, cleavable “Synthesis Tag” to obtain native peptides.

### Commonly Used Cleavable Linkers have a Major Impact on Aggregation

**Figure 4:**
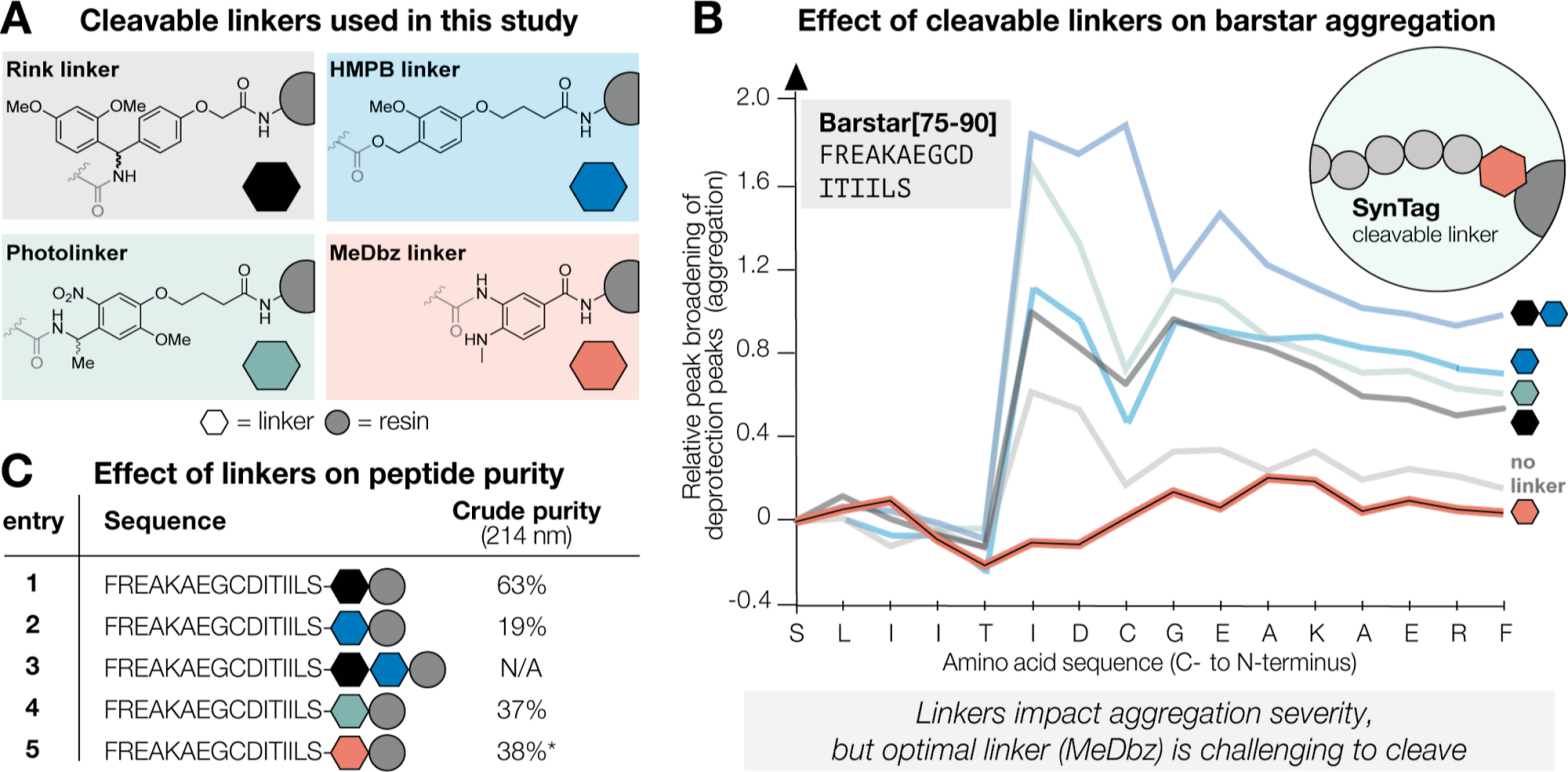
**Common cleavable linkers impact the severity of aggregation, but not its onset.** (A) Chemical structures of cleavable linkers used in this study. The gray circle indicates the SPPS resin. (B) Aggregation as a function of Fmoc-deprotection peak broadening by in-line UV–vis (310 nm) in AFPS for various cleavable linkers. No linker and double linker (Rink/HMPB) were used as negative and positive control experiments, respectively. (C) The impact of the linker on aggregation (maximum values for relative peak broadening throughout the synthesis) translates to the purity of the cleaved Barstar[75–90] in most cases. *Cyclization and immediate cleavage from MeDbz were accompanied by side-products originating from the cleavage protocol, not synthesis itself.

It has previously been predicted that aromatic groups, especially close to the resin, play a major role in aggregation, yet commonly used SPPS-linkers have never been investigated for their impact on aggregation.^7^ We thus synthesized two aggregating test peptides—Barstar[75–90] and GLP-1—on four commonly used SPPS linkers: 2,4-dimethoxybenzyl]-phenoxyacetic acid (Rink amide linker), hydroxymethylmethoxyphenoxybutyric acid (HMPB linker), 4-{4-[1-aminoethyl]-2-methoxy-5-nitrophenoxy}butanoic acid (photolinker), and 3-amino-4-(methylamino)benzoic acid (MeDbz, second generation Dawson linker) (Figure 4A). As a control, we also synthesized both peptides on PEG resin without a cleavable linker.

Linkers had a strong impact on aggregation as confirmed by in-line UV–vis analysis (310 nm) during synthesis and by peptide crude purity (Figure 4B). For Barstar[75–90] synthesized on Rink- and HMPB linkers, strong aggregation was observed, and the comparatively electron-poor photolinker resulted in even greater aggregation (Figure 4B). Confirming our initial hypothesis, the control syntheses without any linker led to much lower aggregation. Notably, in all cases aggregation occurred at the same position, only varying in severity. To our surprise, the best-performing, effectively aggregation-negating linker is the MeDbz linker—even surpassing a synthesis without a linker, as judged by in-line UV–vis analysis. The same trend was observed for GLP-1 (see Supporting Information Section S6.1): while Rink-, HMPB-, photolinker and “no linker” led to significant aggregation, the MeDbz linker successfully negated aggregation. These trends generally translated to the crude purity of the cleaved and deprotected peptides (Figure 4C). However, direct cleavage of the peptide from the resin-bound MeDbz linker requires an activation step,^43^ causing side-products and decreased crude purity (Figure 4C, entry 5). Next, we screened for any additive effects of two linkers coupled to the resin. Combining HMPB and Rink linker indeed led to drastically increased aggregation in Barstar[75–90] and very low peptide purity (Figure 4C, entry 3), and the same was observed for Rink and photolinker (see Supporting Information Section S6.2).

Overall, the MeDbz linker performed significantly better than all other commonly used SPPS linkers. While the MeDbz linker is highly versatile and can be applied to NCL, it can be challenging to cleave the peptide directly off the MeDbz-functionalized resin. We therefore decided to combine MeDbz with the [Arg(Pbf)]_6_ tag coupled to a Rink amide resin (“double linker strategy”)^15,26^ in our final synthesis tag design, wherein MeDbz would link target sequence and [Arg(Pbf)]_6_.

### A Combination of [Arg(Pbf)]_6_ and MeDbz Linker Improves Synthesis Results and Peptide Solubility

**Figure 5:**
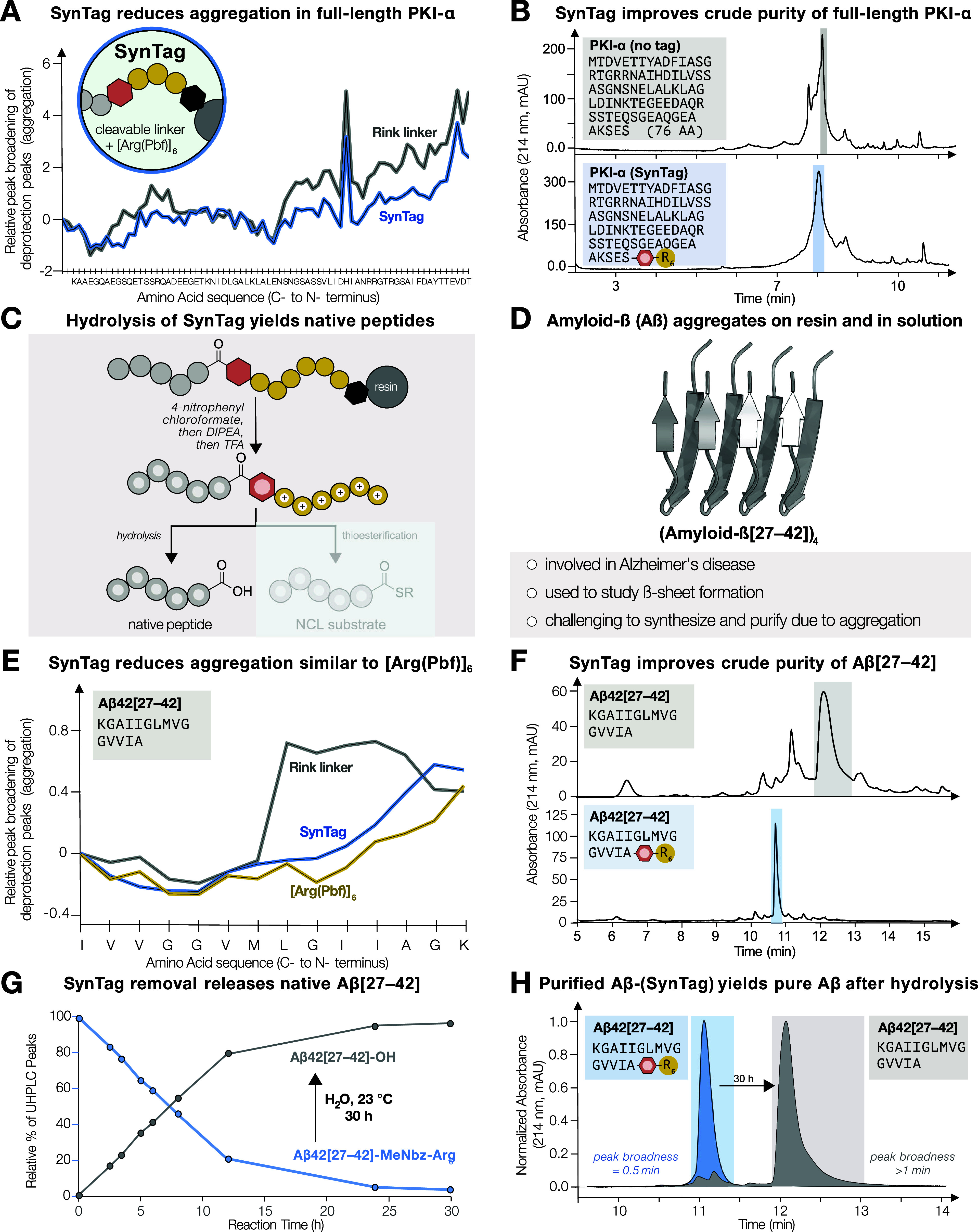
**SynTag improves the chemical synthesis and purification of difficult peptides, including Aβ42[27–42].** (A) SynTag successfully mitigated the onset and severity of aggregation for the synthesis of PKI-α. (B) Adding SynTag to the C-terminus also improved the crude purity of PKI-α. (C) After MeDbz cyclization, cleavage, and global deprotection, C-terminal SynTag improves solubility and can be hydrolyzed to give the native peptide with a C-terminal carboxylic acid. (D) Aggregating and insoluble Aβ42[27–42] serves as test case to demonstrate that SynTag simultaneously improves synthesis and solubility. (E) Aβ42[27–42] synthesis without tag, with Arg_6_ and with SynTag; synthesis was followed by in-line UV–vis analysis. Both Arg_6_ and SynTag have the same effect on deprotection peak broadening (aggregation) during synthesis. (F) SynTag markedly improves crude purity of Aβ42[27–42]. Peaks are less broad with SynTag, indicating increased solubility. (G) After HPLC purification of crude Aβ42[27–42] with SynTag, hydrolysis in water at 23 °C for 30 h afforded native Aβ42[27–42]. (H) Analysis of the start and end points of hydrolysis by UHPLC (214 nm) shows that purified and hydrolyzed Aβ42[27–42] elutes later than Aβ42[27–42]-MeNbz-Arg_6_ and results in a broadening of the peak, as expected.

For the final SynTag design, we investigated a combination of [Arg(Pbf)]_6_ and MeDbz linker for peptide synthesis (Figure 5). To a PEG resin with Rink linker, we first coupled [Arg(Pbf)]_6_, then MeDbz, followed by a manual coupling of the first amino acid of the target peptide. To test our hypothesis, we first synthesized Barstar[75–90] on this SynTag and confirmed that aggregation during synthesis was comparable to the use of [Arg(Pbf)]_6_ or MeDbz alone (see Supporting Information Figure S190). In a negative control experiment, employing [Arg(Pbf)]_6_ followed by a photolinker for Barstar[75–90] synthesis, the beneficial effect of [Arg(Pbf)]_6_ was canceled out and aggregation was again observed (see Supporting Information Figure S186). We then applied our SynTag design to the synthesis of full-length protein PKI-α (cAMP-dependent protein kinase inhibitor-α, 76 AA)^58^ and the “difficult peptide” Crambin (46 AA).^59,60^ Aggregation of PKI-α during AFPS was successfully mitigated using SynTag (Figure 5A), and after activation of the MeDbz linker (to afford MeNbz) and TFA-mediated cleavage, UHPLC analysis showed an improvement in the protein crude purity compared to the synthesis without SynTag (Figure 5B). Similarly, the crude purity of Crambin was improved when SynTag was employed (see Supporting Information Section S7.4).

To demonstrate how SynTag not only improves the synthesis, but also the solubility of peptides, and that SynTag can be removed to afford the native peptide (Figure 5C), we selected amyloid-β (Aβ42[27–42]) as case study. Amyloid-β is a biologically relevant peptide that is investigated for its role in Alzheimer’s disease (Figure 5D). The peptide is notoriously difficult to synthesize because it severely aggregates at the very beginning of the synthesis (i.e., residues 27–42). Once the peptide is successfully synthesized, it is barely soluble and difficult to purify owing to its numerous hydrophobic amino acids.^61^ Acidic conditions traditionally used for HPLC purification of peptides result in Aβ42 aggregation on the column as indicated by a broad, non-resolvable HPLC peak.^62^

SynTag efficiently reduces aggregation during Aβ42[27–42] synthesis and can be used as a solubility handle for purification. Similar to [Arg(Pbf)]_6_ alone, aggregation was suppressed during the synthesis of Aβ42[27–42] with SynTag, and only reappears for the last few couplings (Figure 5E). After MeDbz linker activation,^43^ peptide cleavage, and global deprotection, a crude purity of 73% was determined by UHPLC, a significant improvement over Aβ42[27–42] without SynTag (33% crude purity). A direct comparison of UHPLC traces with and without SynTag highlights improved synthesis results (fewer side-products) and sharper peaks owing to increased hydrophilicity (Figure 5F). The crude material bearing the SynTag was then purified using a standard HPLC protocol, affording pure Aβ42[27–42]-SynTag (1.3 mg, 96% purity by UHPLC, 7% overall yield). SynTag removal was achieved by hydrolysis in water at room temperature for 30 h, releasing native Aβ42[27–42] peptide (Figure 5G,H). As expected, the peak corresponding to the hydrolyzed, native Aβ42[27–42] is broadened under standard UHPLC conditions (Figure 5H).

### The Chemical Synthesis of MYC Transactivation Domain MYC[1–143] can be Achieved Using SynTag

**Figure 6:**
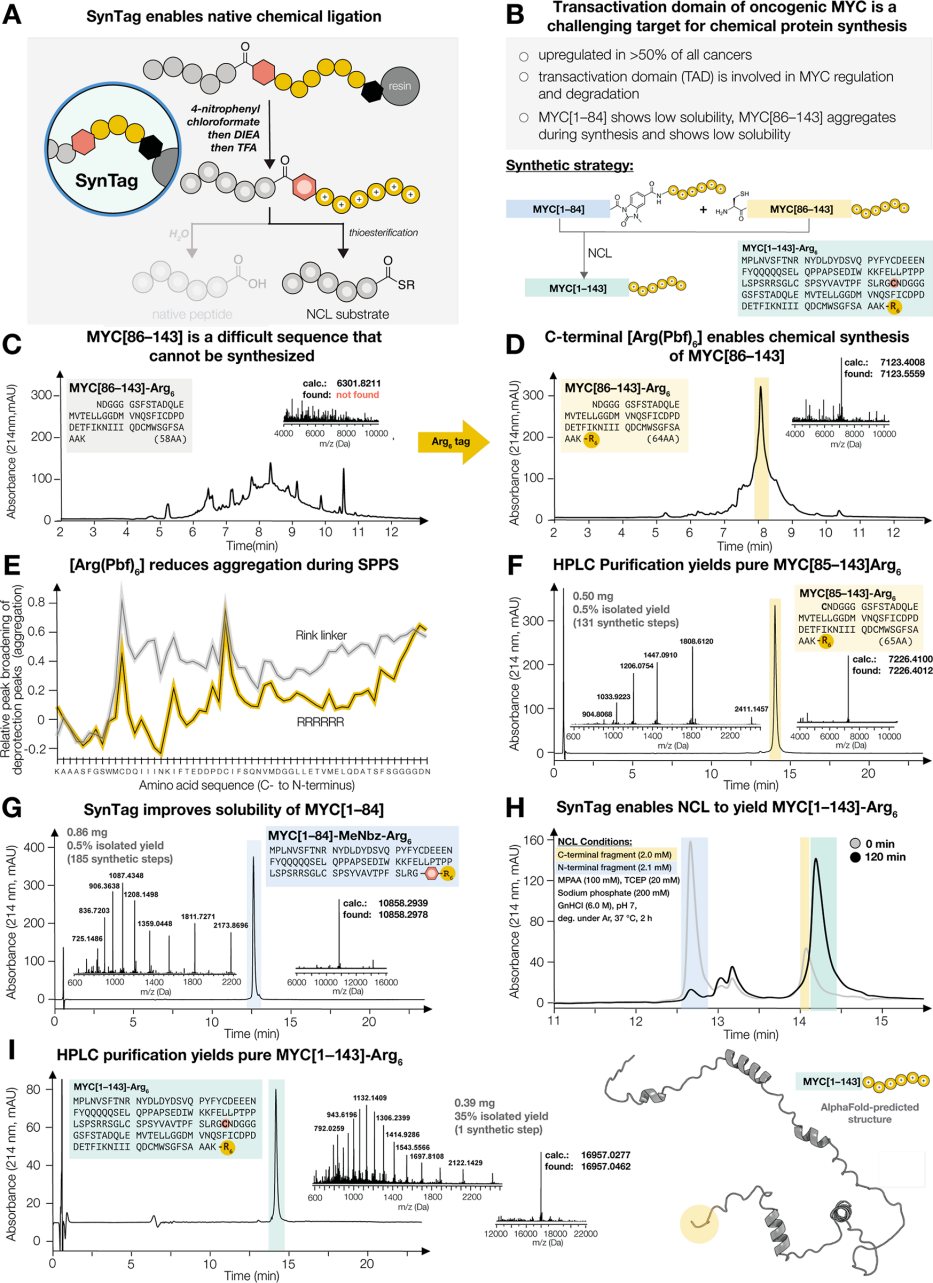
**SynTag enables the chemical synthesis of MYC transactivation domain (TAD) using a single NCL.** (A) After cyclization, cleavage, and deprotection, C-terminal SynTag improves solubility and can be transformed into a thioester for NCL. (B) MYC[1–143] TAD was chosen as a target protein to demonstrate the utility of SynTag in chemical protein synthesis. Solubility issues are expected due to low pI of both envisaged fragments. Furthermore, the C-terminal fragment severely aggregates during synthesis. (C) Synthesis of untagged MYC[86–143] is unsuccessful, and the desired mass was not detected by LC–MS. (D) Successful synthesis of MYC[86–143] by using an Arg_6_ tag, demonstrating suppression of aggregation during long peptide synthesis. (E) Arg_6_ tag effectively suppresses aggregation of MYC[86–143] during synthesis compared to Rink amide linker alone, as illustrated by in-line UV–vis analysis. (F) HPLC purification yields clean MYC[85–143](D85C)-Arg_6_. (G) Synthesis of MYC[1–84] with SynTag improves solubility and yields clean peptide after HPLC purification. (H) Combination of purified peptides in NCL buffer results in complete ligation after 120 min. (I) HPLC purification yields clean MYC[1–143](D85C)-Arg_6_ in 35% isolated yield. MYC[1–143](D85C)-Arg_6_ structure as predicted using AlphaFold, Arg_6_ tag is highlighted in yellow.

To further demonstrate the use of SynTag in chemical protein synthesis (Figure 6A), we set out to synthesize the transactivation domain (TAD) of the oncogenic transcription factor MYC using a single NCL step (Figure 6B). Synthetic, full-length MYC TAD could help decipher its posttranslational modifications (PTMs) and the mechanisms of MYC regulation in cancer. However, the MYC TAD has not been synthesized to date and investigations are limited to much shorter fragments.^63^ To explore MYC TAD synthesis, we first synthesized the two fragments MYC[1–84] and MYC[85–143] (see Supporting Information Section S8) using standard AFPS protocols. This ligation site was chosen to minimize base exposure of the aspartimide-prone Asp[87]-Gly[88] motif. MYC[1–84] could be successfully synthesized despite decreased solubility at high concentrations (calculated pI = 4.41), whereas MYC[86–143] severely aggregated during synthesis, and the crude peptide mass was not detected by LC–MS (Figure 6C). Therefore, we investigated the synthesis of these fragments upon SynTag (to increase solubility and enable thioester formation for NCL for MYC[1–84]), and upon [Arg(Pbf)]_6_ (to improve synthesis and solubility for MYC[85–143]). To facilitate NCL, a cysteine residue was incorporated at position Asp[85].

The N- and C-terminal fragments were both prepared successfully using SynTag and [Arg(Pbf)]_6_, respectively. MYC[86–143] was synthesized on [Arg(Pbf)]_6_-functionalized resin, which successfully suppressed aggregation (Figure 6D,E). The cleaved and deprotected C-terminal fragment showed enhanced solubility (pI increased from 3.48 to 4.98 with Arg_6_), and HPLC purification gave clean MYC[85–143](D85C) (0.50 mg, 0.5% isolated yield) (Figure 6F). The synthesis of MYC[1–84] on SynTag afforded the pure desired fragment (0.86 mg, 0.5% isolated yield after HPLC purification) (Figure 6G). For the ligation to afford MYC[1–143](D85C), the N-terminal fragment (MYC[1–84]-SynTag, 2.1 mM) and C-terminal fragment (MYC[85–143](D85C)-Arg_6_, 2.0 mM) were dissolved in degassed ligation buffer (pH 7.0) containing MPAA and TCEP. To our delight, UHPLC and LC–MS analysis indicated complete ligation after 2 h (Figure 6H), and HPLC purification yielded clean MYC[1–143](D85C)-Arg_6_ (0.39 mg, 35% isolated yield, 96% purity by UHPLC) (Figure 6I).

## Conclusions

Using analytical data obtained from AFPS, we determined that [Arg(Pbf)]_6_ effectively mitigates aggregation during the synthesis by changing the secondary structure of the resin-bound peptide chain. Building on this discovery, we developed a versatile SynTag that improves synthesis outcome, peptide solubility, and can be removed from the target sequence. Screening of various C-terminal amino acid and linker combinations led to a final SynTag design consisting of [Arg(Pbf)]_6_ and MeDbz. While polyarginines and -lysines have been used as solubility tags before,^8,15,25−39^ our results reveal a key benefit of Arg(Pbf): Suppression of peptide β-sheet formation and aggregation during SPPS. In contrast, incorporation of [Lys(Boc)]_6_ at the C-terminus of Barstar[75–90] failed to prevent aggregation and decreased peptide crude purity, despite its potential to improve solubility. Therefore, benefits of [Arg(Pbf)]_6_ at the C-terminus are not only linked to enhanced peptide solubility. SynTag significantly improved the synthesis results of several aggregating sequences, including Barstar[75–90], PKI-α (full-length), Crambin (full-length) and Aβ42[27–42]. While SynTag suppresses aggregation during SPPS, after activation of MeDbz, cleavage, and global deprotection of the peptide, the tag also improves solubility for HPLC and solution-phase reactions. SynTag is a versatile tool in peptide and protein synthesis, as hydrolysis in aqueous media yields the native peptide with a C-terminal carboxylic acid (as demonstrated for Aβ42[27–42]), or that alternatively affords peptide thioesters in situ for NCL, as demonstrated in the first synthesis of MYC[1–143](D85C). Ultimately, SynTag represents a single, unified tool that simultaneously addresses two major challenges in chemical peptide and protein synthesis.

Cleavable peptide tags are popular tools in protein biology and chemistry to enable purification or immobilization, detection of proteins in a complex environment, and to increase solubility (e.g., arginine or lysine tags),^8,15,25−39^ yet their ability to reduce aggregation during SPPS itself had not been realized. While previous methods to suppress aggregation require trial-and-error optimization, SynTag is a broadly applicable tool for both batch- and flow-SPPS. Most tools to improve SPPS—such as backbone protecting groups—are installed just before or at the onset of aggregation, require specialized building blocks, and must be used repetitively to prolong their effect.^48,64^ Here, we introduce a fundamentally different approach by altering the first building blocks coupled to the resin rather than changing the synthesis protocols themselves: We demonstrate that the first amino acids and the cleavable linker, together, have a major impact on aggregation. While linkers only modulate severity, amino acid tags can shift the onset of aggregation and, as a consequence, change the side-product profile. It is, therefore, assumed that amino acid tags alter the conformation of the growing peptide chain on the resin,^44^ while linkers may only intensify existing interactions (e.g., β-sheets). Adding “non-aggregating” building blocks at the very beginning of the synthesis (i.e., directly on the resin) efficiently suppressed aggregation for aggregation-prone peptides of various lengths (10–69 amino acids), with the only exception being α-synuclein[66–82], thus highlighting room for future improvement.

Using IR and SSNMR, we discovered that [Arg(Pbf)]_6_ induces flexible α-helical structures along the resin-bound peptide chains, combatting aggregation by disrupting β-sheet formation. Therefore, the beneficial effects of [Arg(Pbf)]_6_ at the peptide C-terminus is largely due to structural changes of the growing peptide chain during SPPS, with the added advantage of improved solubility upon cleavage and global deprotection of the peptidyl resin.

Combining the methodologies of AFPS and NCL will significantly expand the synthetically accessible protein space. AFPS has been demonstrated to yield peptides >160 amino acids within hours of synthesis time,^4,18^ and numerous ligation methods, such as NCL and KAHA ligation,^3,65^ have been developed to enable ligation for almost every possible amino acid junction.^5^ However, the combination of AFPS and ligation methods has not yet been reported, and aggregation during synthesis as well as solubility of final fragments still require screening efforts to identify suitable fragments. SynTag marks the first report of a combination of AFPS and NCL. The positively charged Arg_6_ of SynTag afforded after resin cleavage has several additional advantageous features (Figure 2E): polyarginine can be used as a protein fusion tag to improve solubility, assist purification, and allow for immobilization.^57^ We, therefore, envisage that our versatile SynTag will become a valuable addition to the chemical protein synthesis toolbox to handle difficult and insoluble sequences, making a broader array of peptides and proteins available for further study.

## Supporting Information Available

The Supporting Information is available free of charge at https://pubs.acs.org/doi/10.1021/jacs.4c14247. In addition, all data supporting this paper are available through Zenodo (DOI: 10.5281/zenodo.10005722).

Experimental methods and additional characterization data (PDF)

## Abbreviations

Aβ: amyloid β
AFPS: automated fast-flow peptide synthesizer
Boc: *tert*-butyloxycarbonyl
calc.: calculated
CPP: cell-penetrating peptide
DIPEA: *N*,*N*-diisopropylethylamine
Fmoc: 9-fluorenylmethyloxycarbonyl
GLP-1: glucagon-like peptide 1
h: hours
hGH: human growth hormone
HATU: hexafluorophosphate azabenzotriazole tetramethyl uronium
HMPB: hydroxymethylmethoxyphenoxybutyric acid
HPLC: high-performance liquid chromatography
JR10: Jung-Redmann 10-mer
KAHA: α-ketoacid-hydroxylamine
LC–MS: liquid chromatography–mass spectrometry
MeDbz: *N*-methyl-diaminobenzoic acid
MeNbz: *N*-acyl-*N*′-methyl-benzimidazolinone
min: minutes
MPAA: 4-mercaptophenylacetic acid
NCL: native chemical ligation
Pbf: 2,2,4,6,7-pentamethyldihydrobenzofuran-5-sulfonyl
PEG: polyethylene glycol
PTM: post-translational modification
SPPS: solid-phase peptide synthesis
SynTag: synthesis tag
TAD: transactivation domain
TCEP: tris(2-carboxyethyl)phosphine
TFA: trifluoroacetic acid
UHPLC: ultrahigh-performance liquid chromatography
UV–Vis: ultraviolet–visible.
