# Supplementary material for: A Versatile “Synthesis Tag” (SynTag) for the Chemical Synthesis of Aggregating Peptides and Proteins
Source: J Am Chem Soc. 2024 Dec 6;146(50):34887–99. doi: 10.1021/jacs.4c14247 (PMC11664589; doi:10.1021/jacs.4c14247)
Supplement: Supplementary file 1 — ja4c14247_si_001.pdf [file ja4c14247_si_001.pdf]

Supplementary Material

Title: A versatile "Synthesis Tag" (SynTag) for the chemical synthesis of aggregating peptides and proteins

Authors: Héloïse Bürgisser[†,1], Elyse T. Williams[†,1], Aliénor Jeandin[1], Robin Lescure[1], Adhvitha Premanand[1], Songlin Wang[2], Nina Hartrampf[1,*]

Affiliations:
[1] Department of Chemistry, University of Zurich, Winterthurerstrasse 190, 8057 Zurich, Switzerland

[2] National Magnetic Resonance Facility at Madison (NMRFAM), University of Wisconsin-Madison, Madison, WI, 53706, United States

[†] These authors contributed equally to this work

* Corresponding author. Email: nina.hartrampf@chem.uzh.ch

1 Table of Contents

1 Table of Contents 2

2 Material and general methods 4

2.1 Reagents and solvents 4
2.2 Peptide Synthesis 4
2.3 Coupling of cleavable linkers to SPPS resin 6
2.4 Determination of resin loading 8
2.5 Peptidyl-resin cleavage and global deprotection 8
2.6 Analytical Ultra-High Performance Liquid Chromatography (UHPLC) 10
2.7 Liquid Chromatography with High-Resolution Electrospray Ionization Mass Spectrometry (LCMS) 10
2.8 Semi-Preparative Reverse-Phase High Performance Liquid Chromatography (RP-HPLC) 11
2.9 Infrared Spectroscopy (IR) 11
2.10 Solid-state Nuclear Magnetic Resonance (SSNMR) 11

3 Amino acid tags 12

3.1 Screening of C-terminal tags: Synthesis of Barstar[75–90] 12
3.2 Evaluation of [Arg(Pbf)]$_6$-Tag: GLP-1 24
3.3 Evaluation of [Arg(Pbf)]$_6$-Tag: hGH[176–191] 28
3.4 Evaluation of [Arg(Pbf)]$_6$-Tag: JR-10 37
3.5 Evaluation of [Arg(Pbf)]$_6$-Tag: MYC[123–143] 41
3.6 Evaluation of [Arg(Pbf)]$_6$-Tag: MYC[85–143] 44
3.7 Evaluation of [Arg(Pbf)]$_6$-Tag: Amyloid-b42[27–42] 48
3.8 Evaluation of [Arg(Pbf)]$_6$-Tag: a-Synuclein[66–82] 51
3.9 Effect of [Arg(Pbf)]$_6$ Tag position, temperature, and resin loading: Barstar[75–90] 55
3.10 Effect of [Arg(Pbf)]$_6$ Tag position and temperature: hGH[176–191](F176Y) 66
3.11 Effect of Arg(Pbf) tag length and position: GLP-1 70
3.12 Recapitulative table of crude purities for amino acid tags 75
3.13 Impact of the protecting groups 77

4 IR evaluation 84

4.1 Summary of the IR constructs and measurements 84
4.2 [Arg(Pbf)]$_9$ for IR measurement 84
4.3 Resin-Bound Barstar[75–90] 85
4.4 Resin-Bound [Arg(Pbf)]$_{15}$ 85
4.5 Resin-Bound Barstar[75–90]-[Arg(Pbf)]$_6$ 85

5 NMR evaluation 86

5.1 Barstar[75–90] ^{13}C-labeled 86
5.2 Barstar[75–90]: [Arg(Pbf)]$_6$-Tag ^{13}C-labeled 87
5.3 [Arg(Pbf)]$_{15}$ ^{13}C-labeled 89
5.4 Solid-State NMR (SSNMR) 90

6 Linker screening 92

6.1 Evaluation of cleavable linkers: GLP-1 92
6.2 Evaluation of cleavable linkers: Barstar[75–90] 98
6.3 Recapitulative table of crude purities for test peptides determined by UHPLC 110

7 SynTag – combining [Arg(Pbf)$_6$] and MeDbz linker into a versatile synthesis tag .. 110

7.1 Preparation of MeDbz-[Arg(Pbf)$_6$]-Rink amide resin 110
7.2 Barstar[75–90]-SynTag 110
7.3 cAMP-dependent protein kinase inhibitor alpha (PKI-a) 113
7.4 Crambin 117

7.5 Amyloid-B42[27–42]-SynTag 120
7.6 Hydrolysis of SynTag to afford Amyloid-B42[27–42]-OH 123

8 **Chemical Synthesis of MYC[1–143] using SynTag 125**

8.1 SynTag: MYC[1–84]-MeNbz-Arg6 125
8.2 MYC[85–143]-(D85C)-Arg6 127
8.3 Native Chemical Ligation: MYC[1–143]-(D85C)-Arg6 130

9 **Appendix: IR Spectras 133**

9.1 [Arg(Pbf)]9 (photocleaved from resin) 133
9.2 Resin-Bound [Arg(Pbf)]15 134
9.3 Resin-Bound Barstar[75–90] 135
9.4 Resin-Bound Barstar[75–90]-[Arg(Pbf)]6 136

2 Material and general methods

2.1 Reagents and solvents

Fmoc- and side chain-protected L-amino acids (Fmoc-Ala-OH, Fmoc-Arg(Pbf)-OH, Fmoc-Asn(Trt)-OH, Fmoc-Asp(O*t*Bu)-OH, Fmoc-Cys(Trt)-OH, Fmoc-Gln(Trt)-OH, Fmoc-Glu(O*t*Bu)-OH, Fmoc-Gly-OH, Fmoc-His(Trt)-OH, Fmoc-Ile-OH, Fmoc-Leu-OH, Fmoc-Lys(Boc)-OH, Fmoc-Met-OH, Fmoc-Phe-OH, Fmoc-Pro-OH, Fmoc-Ser(*t*Bu)-OH, Fmoc-Thr(*t*Bu)-OH, Fmoc-Trp(Boc)-OH, Fmoc-Tyr(*t*Bu)-OH, Fmoc-Val-OH) were purchased from the Novabiochem-line from Sigma-Aldrich Canada Ltd.; Boc-L-Met-OH was purchased from Sigma-Aldrich Canada Ltd; Boc-L-Phe-OH was purchased from Fluorochem Ltd; Boc-L-Lys(Boc)-OH was purchased from Carl Rotha GmbH & Co. KG; ^{13}C-labeled Fmoc-Ala-OH, Fmoc-Gly-OH, Fmoc-Ile-OH and Fmoc-Arg(Pbf)-OH were purchased from Cambridge Isotope Laboratories Inc; *O*-(7-azabenzotriazol-1-yl)-*N,N,N',N'*-tetramethyluronium hexafluorophosphate (HATU) and (7-azabenzotriazol-1-yloxy)tripyrrolidinophosphonium hexafluorophosphate (PyAOP) were purchased from Advanced ChemTech CreoSalus; 4-(dimethylamino)pyridine (DMAP) was purchased from Merck; *N*,*N*'-diisopropylcarbodiimide (DIC) was purchased from Sigma-Aldrich; *N*,*N*-diisopropylethylamine (iPr$_2$NEt, DIPEA, 99.5%) was purchased from Sigma-Aldrich; trifluoroacetic acid (TFA, for HPLC, ≥99.0%), triisopropylsilane (TIPS, 98%) and 3,6-dioxa-1,8-octane-dithiol (DODT, 95%) were purchased from Sigma-Aldrich. *N,N*-Dimethylformamide (DMF) was purchased from the Supelco-line from Sigma-Aldrich Canada Ltd.; dichloromethane (DCM, ≥99.8%) was purchased from Fisher Scientific Ltd.; diethyl ether was purchased from Honeywell Riedel-de Haën; acetonitrile (MeCN, for HPLC gradient grade, ≥99.9%) was purchased from Sigma-Aldrich. NovaPEG Rink Amide resin (0.41 or 0.20 mmol/g loading), NovaPEG amino resin (0.48 mmol/g loading) and NovaPEG HMPB resin was purchased from the Novabiochem-line from Sigma-Aldrich Canada Ltd; HMPB ChemMatrix® resin was purchased from Sigma-Aldrich Canada Ltd. 4-[(2,4-Dimethoxyphenyl)(Fmoc-amino)methyl]phenoxyacetic acid (Fmoc-Rink amide linker-OH) was purchased from Fluorochem Ltd; 4-[4-[1-(9*H*-fluoren-9-ylmethoxycarbonylamino)ethyl]-2-methoxy-5-nitrophenoxy]butanoic acid (Fmoc-photolinker-OH) was purchased from Iris Biotech GmbH; 3-(Fmoc-amino)-4-(methylamino)benzoic acid (Fmoc-MeDbz-OH) was purchased from Iris Biotech. Tris(2-carboxyethyl)phosphine (TCEP) was purchased from Fluorochem Ltd; Guanidinium chloride (GnHCl) was purchased from Apollo Scientific Ltd; 4-mercaptophenylacetic acid (MPAA) was purchased from Sigma-Aldrich Canada Ltd; sodium phosphate (di- and mono-basic) was purchased from Avantor Inc.

2.2 Peptide Synthesis

2.2.1 Automated flow-based peptide synthesis (AFPS)

Peptides were synthesized on an automated-flow system built in the Hartrampf lab, which is similar to the published AFPS system.(*4*) Capitalized letters refer to L-amino acids. Unless otherwise noted, the following settings were used for peptide synthesis: flow rate = 20 mL/min or 40 mL/min for coupling and deprotection steps (as specified), wherein the reactor base (containing resin) was kept at 90 °C, with pre-activation at 90 °C or 30 °C (heating loop) as specified. The standard synthetic cycle involves a first step of prewashing the resin at 90 °C for 60 s at 40 mL/min. During the coupling step, three HPLC pumps are used: a 50 mL/min pump head pumps the activating agent,

a second 50 mL/min pump head pumps the amino acid, and a 5.0 mL/min pump head pumps *i*Pr_2NEt (*neat*). The 50 mL/min pump head pumps delivered 0.398679 mL of liquid per pump stroke, the 5.0 mL/min pump head pumps 3.9239 × 10^{-2} mL of liquid per pump stroke.

All peptides were prepared by AFPS on NovaPEG Rink Amide resin (0.41 or 0.20 mmol/g) and standard Fmoc/*t*Bu protected amino acids (0.40 M in DMF, 0.20 M final concentration) were coupled using HATU (0.38 M in DMF, 0.19 M final concentration) or PyAOP (0.38 M in DMF, 0.19 M final concentration) with DIPEA (delivered *neat*, approx. 0.27 M final concentration).

2.2.2 AFPS method at 20 mL/min flow rate

For 20 mL/min method, for amino acids D, E, F, G, I, K, L, M, P, W, and Y, a total volume of 6.4 mL of the "coupling solution" (i.e., amino acid [0.20 M], HATU or PyAOP [0.19 M], and DIPEA [0.27 M] in DMF) was applied for each coupling. For amino acids A, C, H, N, Q, R, S, T, and V, a total of 10.4 mL of "coupling solution" was applied for each coupling. Removal of the N^{α}-Fmoc group was achieved using 20% piperidine with 1% formic acid in DMF (6.4 mL, *v*/*v*/*v*) at a flow rate of 20 mL/min with preheating at 90 °C for all Fmoc-protected amino acids except C and H, for which preheating of the deprotection solution was at 30 °C. Between each coupling and deprotection step, the resin was washed with DMF (32 mL) at a flow rate of 40 mL/min with preheating at 90 °C for all amino acids except C and H, for which the DMF was preheated at 30 °C. After completion of the peptide sequence, the resins were manually washed with DCM (3 × 5 mL) and dried under reduced pressure.

2.2.3 AFPS method at 40 mL/min flow rate

For the 40 mL/min method, for amino acids D, E, F, G, I, K, L, M, P, S, W, and Y, a total volume of 10.4 mL of the "coupling solution" (i.e., amino acid [0.20 M], HATU or PyAOP [0.19 M], and DIPEA [0.27 M] in DMF) was applied for each coupling. For amino acids A, C, H, N, Q, R, S, T, and V, a total of 20.8 mL of "coupling solution" was applied for each coupling. Removal of the N^{α}-Fmoc group was achieved using 20% piperidine with 1% formic acid in DMF (10.4 mL, *v*/*v*/*v*) at a flow rate of 40 mL/min with preheating at 90 °C for all Fmoc-protected amino acids except H, for which preheating of the deprotection solution was at 30 °C. Between each coupling and deprotection step, the resin was washed with DMF (32 mL) at a flow rate of 40 mL/min with preheating at 90 °C for all amino acids except H, for which the DMF was preheated at 30 °C. After completion of the peptide sequence, the resins were manually washed with DCM (3 × 5 mL) and dried under reduced pressure.

2.2.4 Batch Solid-Phase Peptide Synthesis (Batch-SPPS)

Unless otherwise noted, pre-functionalized NovaPEG Rink Amide resin (0.41 mmol/g loading) was used in all batch-SPPS experiments, and the resin loading was confirmed using the protocol described in **Section 2.4**.

Unless otherwise noted, the amino resin (50 mg, 21 μmol, 1.0 eq.) was swelled with DCM (1 × 5 mL) for 1 min, drained, and washed with DMF (1 × 5 mL). For each coupling, a solution of Fmoc- and side-chain-protected amino acid (0.50 mL, 0.20 M in DMF, 5.0 eq.) and HATU (0.50 mL, 0.19 M in DMF, 4.8 eq.) was prepared. To this solution, DIPEA (36 μL, 0.20 mmol, 10 eq.) was added, and the solution was gently agitated at 23 °C for 1 min. The solution was then added to the resin, and the reaction was gently stirred for 20 s, then left at 23 °C for 30 min. The

resin was then drained, washed with DMF (3 × 5 mL) and DCM (3 × 5 mL). For each deprotection step, 20% piperidine in DMF (*v*/*v*) (3.0 mL) was added to the resin, and the reaction was gently stirred for 20 s then left at 23 °C for 20 min. The resin was then drained, then washed with DMF (3 × 5 mL) and DCM (3 × 5 mL).

2.3 Coupling of cleavable linkers to SPPS resin

2.3.1 Rink amide linker

Unless otherwise noted, pre-functionalized NovaPEG Rink Amide resin (0.41 or 0.20 mmol/g loading) was used in all experiments, and the resin loading was confirmed using the protocol described in **Section 2.4**.

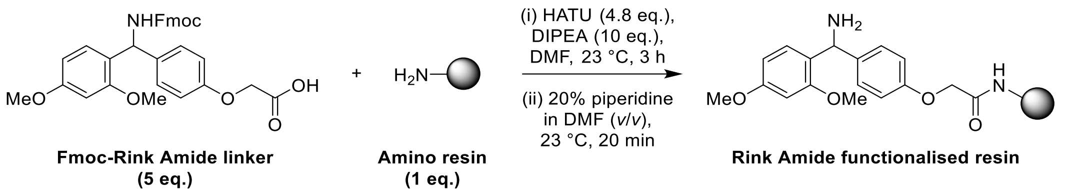

SI Figure 1. Coupling of Fmoc-Rink Amide linker to an amino resin.

Whenever manual Rink amide coupling was required, the following protocol was used: The amino resin (1.0 eq.) was swelled with DCM (1 × 5 mL) for 1 min, washed with DMF (1 × 5 mL) and the solvent was then removed by filtration under reduced pressure. To a solution of Fmoc-Rink linker (0.15 M in DMF, 5 eq.), HATU (0.38 M in DMF, 4.8 eq.) and DIPEA (10 eq.) were added, and the solution was gently agitated at 23 °C for 1 min. The solution was then added to the resin and the reaction was gently agitated at 23 °C for 3 h (**SI Figure 1**). Then the resin was drained, washed with DMF (3 × 5 mL) and DCM (3 × 5 mL), and dried under reduced pressure. Then, an Fmoc-loading test was performed as described in **Section 2.4**. Finally, the Fmoc-group was removed by treating the resin with a solution of 20% piperidine in DMF (*v*/*v*) (5.0 mL, 3 × 5 min). The resin was then drained, washed with DMF (2 × 5 mL) and DCM (2 × 5 mL), then dried under reduced pressure.

2.3.2 HMPB linker

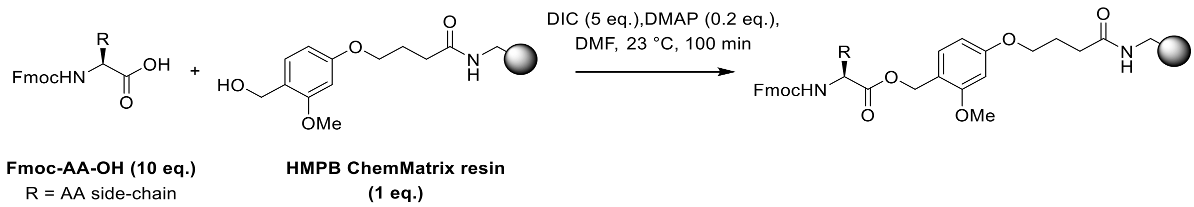

SI Figure 2. Introduction of first amino acid to HMPB ChemMatrix® resin.

Unless otherwise noted, pre-functionalized HMPB ChemMatrix® resin (0.62 mmol/g loading) was used, and the resin loading was confirmed using the protocol described in **Section 2.4**. The introduction of the first amino acid was carried out manually as follows: To the desired Fmoc- and side-chain protected amino acid (0.40 M, 10 eq.), DIC (5.0 eq.) and DMAP (0.20 M, 0.20 eq.) were added and gently agitated at 23 °C for 30 s. The solution was then transferred to the pre-swelled HMPB ChemMatrix® resin (1.0 eq.) and the mixture was left for 100 minutes at 23 °C (**SI Figure 2**). The resin was then drained, washed with DMF (3 × 3 mL) and DCM (3 × 3 mL), and dried under reduced pressure.

2.3.3 Photolinker

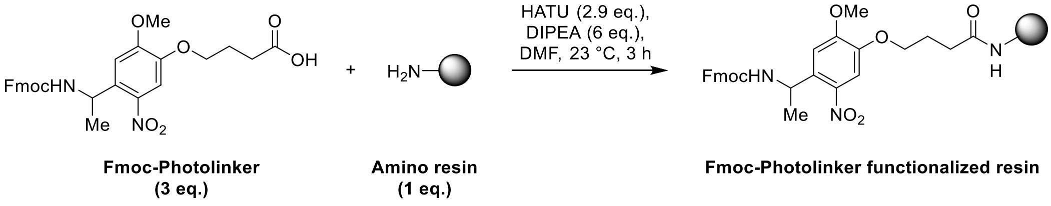

SI Figure 3. Coupling of Fmoc-Photolinker to an amino resin.

The NovaPEG amino resin (1 eq.) was swelled with DCM (1 × 5 mL), washed with DMF (1 × 5 mL) and the solvent was then removed by filtration under reduced pressure. To a solution of Fmoc-Photolinker (0.36 mmol, 0.10 M in DMF, 3.0 eq.), HATU (0.35 mL, 0.10 M in DMF, 2.9 eq.) and DIPEA (0.72 mmol, 6.0 eq.) were added, and the solution was manually shaken at 23 °C for 1 min. The solution was then added to the resin, and the reaction was gently shaken at 23 °C for 3 h (**SI Figure 3**). Then the reagent was removed from the resin, and the resin was washed with DMF (3 × 5 mL), and DCM (3 × 5 mL), and dried under reduced pressure. Then, a loading test was performed as described in **Section 2.4**.

2.3.4 MeDbz linker

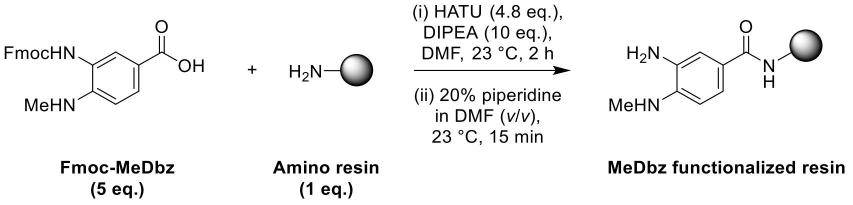

SI Figure 4. Coupling of Fmoc-MeDbz-OH to an amino resin.

The NovaPEG amino resin (1.0 eq.) was swelled with DCM (1 × 5 mL), washed with DMF (1 × 5 mL) and the solvent was then removed by filtration under reduced pressure. To a solution of Fmoc-MeDbz-OH linker (1.2 mmol, 0.13 M in DMF, 5.0 eq.) and HATU (1.1 mmol, 0.13 M in DMF, 4.8 eq.), DIPEA (0.42 mL, 2.4 mmol, 10 eq.) was added, and the solution was gently agitated at 23 °C for 1 min. The solution was then added to the resin, and the reaction was gently agitated at 23 °C for 2 h (**SI Figure 4**). The resin was then drained, washed with DMF (3 × 5 mL) and DCM (3 × 5 mL), and dried under reduced pressure. Then, a loading test was performed as described in **Section 2.4**. Finally, the Fmoc group was removed with a solution of 20% piperidine in DMF (*v*/*v*) (5 mL) at 23 °C for 15 min. The resin was then drained, washed with DMF (2 × 5 mL) and DCM (2 × 5 mL) and dried under reduced pressure.

Unless stated otherwise, the first amino acid was then manually coupled on the resin. The MeDbz-resin (1.0 eq.) was swollen with DCM (1 × 5 mL), DMF (1 × 5 mL) and the solvent was then removed under reduced pressure. A solution of Fmoc- and side-chain protected amino acid (0.40 M in DMF, 5.0 eq.) and HATU (0.38 M in DMF, 4.8 eq.) was prepared. To this solution, DIPEA (10 eq.) was added, and the solution was manually shaken for 1 min at 23 °C. The solution was then added transferred to the resin, the reaction was gently stirred for 20 s and the reaction was left at 23 °C for 2 h. The resin was then drained, washed with DMF (3 × 5 mL) and DCM (3 × 5 mL), and dried under reduced pressure.

2.4 Determination of resin loading

Three samples of the resin (2–3 mg, accurately weighed to three significant figures) were treated with a solution of 20% piperidine in DMF (*v*/*v*) (1.5 mL) and left to stand at 23 °C for 20 min with occasional agitation. UV absorbance (λ = 290 nm) of each sample was then measured in triplicate, with 20% piperidine in DMF (*v*/*v*) as a blank. The loading was calculated with the following equation:

$$\frac{Absorbance\ measurement}{Mass\ of\ resin\ (mg) \times 2.59} = Fmoc\ loading\ (mmol/g)$$

Wherein 'Absorbance measurement' is the mean average of triplicates for each sample. The final loading value was obtained by mean average of the calculated loadings from the three resin samples.

2.5 Peptidyl-resin cleavage and global deprotection

2.5.1 Protocol A: TFA-mediated cleavage and global deprotection

The peptides were cleaved using a solution of TFA/TIPS/DODT/H_2O (94:1:2.5:2.5, *v*/*v*/*v*/*v*, 1–3 mL) for 2 h at 23 °C with gentle mixing. TFA was then removed by evaporation under a light stream of N_2, and the peptides were precipitated and isolated by centrifugation from ice-cold diethyl ether (2 × 15 mL), twice. The resulting peptide pellets were then briefly dried under a light stream of N_2, then dissolved in an aqueous solution containing 10–50% MeCN and 0.1% TFA, and lyophilized. Crude peptides were then analyzed by LCMS and UHPLC.

2.5.2 Protocol B: TFA- and photolysis-mediated cleavage of photolinker

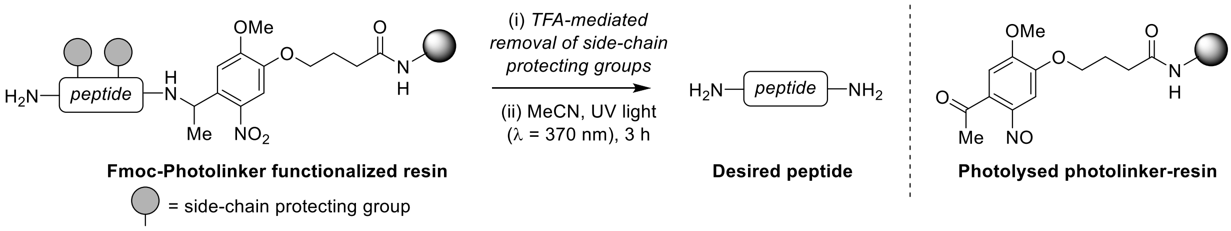

SI Figure 5. Deprotection and cleavage of photolinker peptidyl-resins. TFA-mediated removal of side-chain protecting groups, followed by UV-mediated cleavage of the peptidyl-resin.

The peptides were subjected to global deprotection on-resin using a solution of TFA/TIPS/DODT/H_2O (94:1:2.5:2.5, *v*/*v*/*v*/*v*, 1–3 mL) for 2 h at 23 °C with gentle mixing. The solid support was then washed with DCM (3 × 3 mL) and then acetonitrile (3 × 3 mL). The resin was then suspended in acetonitrile (1.5 mL) and exposed to UV light (370 nm) for 3 h, according to literature procedures.(*56*) (**SI Figure 5.**). The resulting supernatant was directly analyzed for purity by LCMS and UHPLC.

2.5.3 Protocol C: Activation of MeDbz linker directly attached to resin, followed by TFA-mediated global side-chain deprotection, then hydrolysis of the MeNbz-linked peptides.

The linker was activated according to reported literature procedures.(*43*) Note: this procedure was carried out for peptides directly attached to the resin via MeDbz linker, i.e. without an acid-labile (e.g. Rink amide) linker. Activation of the MeDbz-Arg_6-Rink amide linker system (SynTag) was

carried out as described in the corresponding experimental procedures, i.e. for activation and cleavage to afford MYC[1-84]-MeNbz-Arg_6, please see the procedures described in **Section 8**.

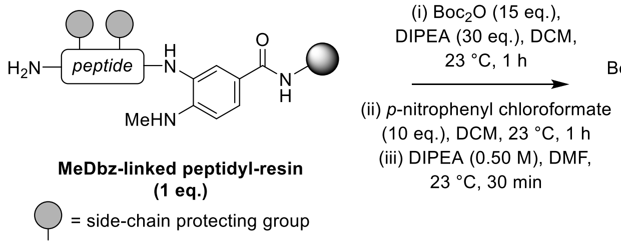

SI Figure 6. Activation of MeDbz linker, directly attached to an amino resin. (i) Boc protection of the N-terminal amino acid. (ii) Acylation of MeDbz with *p*-nitrophenyl chloroformate. (iii) Cyclisation of the linker to afford the MeNbz-linked peptidyl-resin.

Boc protection of the peptidyl-resin (**SI Figure 6**). To the peptidyl-resin (approx. 10 mg) was added a solution of Boc anhydride (15 eq.), DIPEA (30 eq.) in DCM (1.0 mL), and the reaction was left to stand at 23 °C for 1 h. The resin was then filtered and washed with DCM (3 × 3 mL).

Formation of the N-acylurea (MeNbz) (**SI Figure 6**). Unless stated otherwise, a solution of *p*-nitrophenyl chloroformate (10–20 eq.) in DCM (1.0 mL) was added to the Boc-protected peptidyl-resin, and the reaction was left to stand at 23 °C for 1 h. The resin was then filtered and washed with DCM (3 × 3 mL) and DMF (3 × 3 mL). Then, a solution of DIPEA (0.50 M in DMF, 1.0 mL) was added to the resin and the reaction was left to stand at 23 °C for 30 min. The resin was then filtered and washed with DMF (3 × 3 mL) then DCM (3 × 3 mL) and dried under reduced pressure.

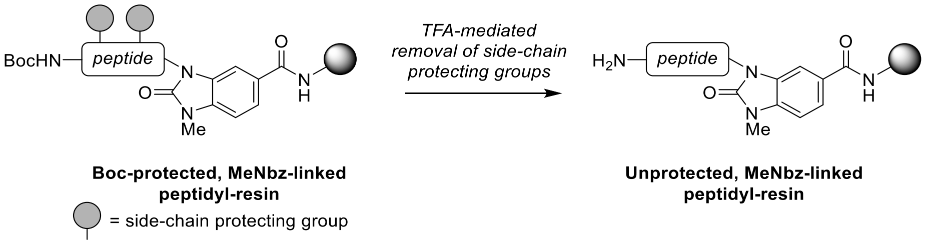

SI Figure 7. Side-chain deprotection of MeNbz-linked peptidyl resins (without TFA-cleavable linker).

Global side-chain deprotection of the peptidyl-resin (**SI Figure 7**). TFA-mediated side-chain protecting group removal was carried out according to Cleavage Protocol A (**Section 2.5.1**), then the filtrate was discarded and the resin was washed with DMF (3 × 3 mL).

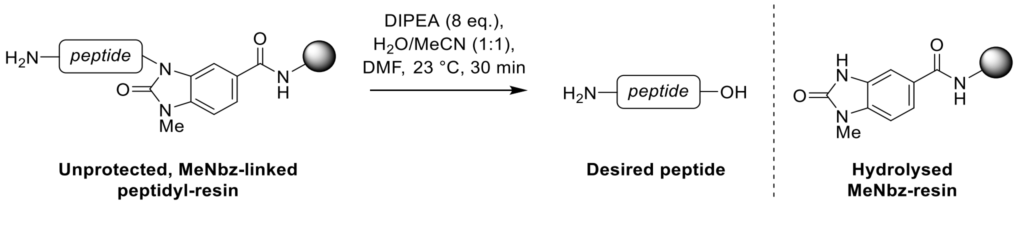

SI Figure 8. Hydrolysis of unprotected, MeNbz-linked peptidyl-resins.

Hydrolysis of the unprotected MeNbz-linked peptidyl-resin (**SI Figure 8**). A solution of H_2O/MeCN (1:1, *v/v,* 1.0 mL) with DIPEA (8.0 eq.) in DMF (0.50 mL) was added to the peptidyl-resin (1.0 eq.) and the reaction was left to stand at 23 °C for 30 min. The resulting supernatant was then collected by filtration, evaporated to dryness, then resuspended in a solution of 20% MeCN in H_2O and analyzed by UHPLC and LCMS.

2.6 Analytical Ultra-High Performance Liquid Chromatography (UHPLC)

For determination of purity by UHPLC, the filtered peptide solution was diluted in 10–50% acetonitrile (MeCN) in water with 0.1% TFA (500 µL) to a final concentration of approximately 0.5 mg/mL. The samples were analyzed on Agilent 1290 Infinity II Series, using Agilent OpenLab CDS and ChemStation software.

For standard analysis of all peptide samples, analytical UHPLC spectra were recorded on an analytical Agilent Zorbax 300SB-C18 column (2.1 mm × 150 mm, 5 µm particle size) kept at 40 °C, at a flow rate of 0.30 mL/min with UV detection at 214 nm. Where specified, an Agilent Poroshell 300SB-C8 column (2.1 mm × 75 mm, 5 µm particle size) was used instead, at a flow rate of 0.80 mL/min with UV detection at 214 nm. A binary solvent system was used, wherein Solvent A was 5% MeCN in 95% water with 0.1% TFA, and Solvent B was 95% MeCN containing 5% water and 0.1% TFA. A linear gradient of 0–100% Solvent B, corresponding to 5–95% MeCN, over 20 min (*ca.* 4.5% MeCN/min) or over 10 min (*ca.* 9.0% MeCN/min) was used, as specified. At the end of the gradient, 100% Solvent B was pumped at 0.3 mL/min for 2 min. Then, the column was re-equilibrated at 0% Solvent B for 5 min. Purities of the crude and purified peptides were determined by ChemStation integration of all UHPLC signals at 214 nm in the area of 3–20 min (20 min gradient) or 2–10 min (10 min gradient).

2.7 Liquid Chromatography with High-Resolution Electrospray Ionization Mass Spectrometry (LCMS)

For determination of peptide masses and purity by LCMS, the filtered peptide solution was diluted in either (i) 10–50% acetonitrile (MeCN) in water with 0.1% TFA (60–500 µL), or (ii) GnHCl (6.0 M) to a final concentration of approximately 0.1 mg/mL. The samples were analyzed on an Acquity UPLC (Waters, Milford, USA) connected to an Acquity eλ diode array detector and a Synapt G2HR-ESI-QTOF-MS (Waters, Milford, USA).

For standard analysis of all peptide samples, LCMS spectra were recorded on an Acquity BEH C8 HPLC column (2.1 × 100 mm, 1.7 µm particle size, Waters) kept at 30 °C at a flow rate of 0.4 mL/min with UV detection at 190–300 nm. A binary solvent system was used, wherein Solvent A was water containing 0.02% formic acid and 0.04% TFA, and Solvent B was MeCN containing 0.04% formic acid and 0.02% TFA. For samples containing GnHCl and for sample dissolved in ligation buffer the first 3 min of the LCMS method was diverted to the waste after passing through the column, to prevent salt contamination of the MS.

LCMS Gradient A: Isocratic at 10% Solvent B for 3 min, then linear gradient of 10–70% Solvent B over 9 min, followed by isocratic at 70% for 1 min.
LCMS Gradient B: Isocratic at 3% Solvent B for 3 min, then linear gradient of 3–95% Solvent B over 9 min, followed by isocratic at 95% Solvent B for 1 min.

UV spectra recorded at 1.2 nm resolution and 20 points s^{-1}; ESI: positive ionization mode, capillary voltage 3.0 kV, sampling cone 40V, extraction cone 4V, N_2 cone gas 4 L h^{-1}, N_2 desolvation gas 800 L min^{-1}, source temperature 120 °C; mass analyzer in resolution mode: mass range 150–3000 *m*/*z* with a scan rate of 1 Hz; mass calibration to <2 ppm within 50–2500 *m*/*z* with a 5.0 mM aq. Soln. of HCO_2Na, lock masses: *m*/*z* 195.08810 caffein, 0.7 ng mL^{-1}) and 556.2771 (leucine-enkephalin, 2 ng mL^{-1}).

LC-QTOF. Where specified, LC-QTOF was used to determine peptide masses, wherein the filtered peptide solution was diluted in 10–50% acetonitrile (MeCN) in water with 0.1% TFA (500 µL) to a final concentration of approximately 0.1 mM. The samples were then analyzed on an Agilent 1290 Infinity II Series UHPLC, which is connected to an Agilent 1260 Infinity II Series VWD, and an Agilent 6546 LC/Q-TOF. Separation was carried out on an Agilent Poroshell 300SB-C8 HPLC column (5 µm particle size, 2.1 × 75 mm) kept at 50 °C, with a sample injection volume of 5 µL. The elution was performed at a flow rate of 0.80 mL/min with solvent A: H_2O + 0.1% formic acid and solvent B: MeCN + 0.1 formic acid with the following LC-MS gradient**:** isocratic at 5% Solvent B for 1.5 min, followed by a linear gradient of 5–95% Solvent B over 10 min, followed by isocratic at 95% Solvent B for 1 min. Ion source parameters for ESI were: positive ionization mode, capillary voltage 3.5 kV, nozzle voltage 1 kV, gas temperature 320 °C, N_2 drying gas flow 8 L/min, nebulizer pressure 35 psi, sheath gas temperature 350 °C, N_2 sheath gas flow 11 L/min, fragmentor voltage 100 V, and skimmer voltage 65 V. Parameters for the mass analyzer in MS (Seg) mode were: mass range 10–3200 *m/z* with an acquisition rate of 1 spectra/sec and time of 1000 ms/spectra; Mass calibration took place using the Agilent low-concentration tune mix.

All mass spectra show deconvoluted masses from the raw *m/z* values, calculated using Mestrelab Research S.L.© MestReNova v. 14.1 Mnova MS Suite. Purity based on LCMS was calculated by calculating the Area Under the Curve (AUC) of desired product peak as a percentage of the AUC of all peaks (within 2–9 min) of the absorbance chromatogram (λ = 214 nm). Monoisotopic and average masses were calculated using ChemDraw Version 18.2.

2.8 Semi-Preparative Reverse-Phase High Performance Liquid Chromatography (RP-HPLC)

Semi-preparative RP-HPLC was performed on a Shimadzu prominence HPLC system (Shimadzu Corp., Japan) with a CBM-40 system controller module, an FRC-10A fraction collector, two LC-20AR pumps, and an SPD-40 UV/VIS detector, using either an Agilent Zorbax 300SB-C18 Semi-Preparative column (9.4 × 250 mm, 5 µm particle size), or an Agilent Eclipse XDB-C8 Semi-Preparative column (9.4 × 250 mm, 5 µm particle size) kept at 23 °C, with a flow rate of 3.5 mL/min. A binary solvent system was used, wherein Solvent A was H_2O containing 0.1% TFA, and Solvent B was MeCN containing 0.1% TFA. Purifications were executed using the gradients specified in each procedure.

2.9 Infrared Spectroscopy (IR)

Infrared spectra were recorded on a JASCO FT/IR-4100 spectrometer equipped with an ATR accessory. All samples were analyzed in the solid state without any prior preparation.

2.10 Solid-state Nuclear Magnetic Resonance (SSNMR)

The SSNMR experiments were performed at NMRFAM (the National Magnetic Resonance Facility at Madison) using a 600 MHz Bruker spectrometer with a Phoenix 3.2 mm HCN triple resonance probe at a 10 °C variable temperature setpoint. The MAS rate was 13.333 kHz. For the ^{13}C cross-polarization (CP) 1D experiment, the ^{13}C polarization was prepared with an adiabatic CP using a downward tangential ramp pulse for the ^{1}H channel and a rectangular pulse for the ^{13}C channel. The CP contact time was 2.0 ms, the ^{13}C rf amplitude was 45 kHz and the average ^{1}H rf amplitude was at 70 kHz. The ^{13}C chemical shift was acquired using an 85 kHz SPINAL-64 ^{1}H decoupling. The

acquisition time was 15.4 ms with 5 μs dwell time. The recycle delay was 1.5 s, and the total experimental time was 26 min. For the 2D $^{13}C/^{13}C$ correlation experiment, the ^{13}C polarization was prepared using the same CP condition as mentioned in the ^{13}C CP 1D experiment. The ^{13}C chemical shift was recorded with a t_1 increment of 25 μs and the maximum t_1 period of 6 ms (480 points). The $^{13}C/^{13}C$ polarization transfer was achieved using a 50ms DARR mixing with a 13.33 kHz ^1H rf amplitude.* ^{13}C chemical shifts were recorded during the t_2 period under an 85 kHz SPINAL-64 ^1H decoupling. The t_2 acquisition time was 15.4 ms with a 5 μs dwell time. The recycle delay was 1.5 s, and the total experimental time was 13 h for the dry samples and 34 h for the DMF rehydrated samples. All spectra are referenced to DSS by using Adamantane as a secondary external standard. The lefthand ^{13}C signal of Adamantane is referenced to 40.48 ppm.

3 Amino acid tags

3.1 Screening of C-terminal tags: Synthesis of Barstar[75–90]

3.1.1 Barstar[75–90]: Without tag (reference)

```
                    10        16
     H2N-FREAKAEGCD ITIILS-CONH2      (16 AA)
```

The peptide Barstar[75–90] was synthesized on commercially available Novabiochem® NovaPEG Rink Amide resin (0.41 mmol/g, 50 mg, 21 μmol) using the standard AFPS protocol (**Section 2.2.1**, 40 mL/min flowrate) (**SI Figure 9**). Total synthesis time to afford resin-bound Barstar[75–90] was approximately 0.8 h. Cleavage of the peptidyl-resin (13 mg, approx. 3.4 μmol) according to Cleavage Protocol A (**Section 2.5.1**) afforded the crude peptide as a colorless solid (3.6 mg, 62% purity by LCMS [**SI Figure 10**], 63% purity by UHPLC [**SI Figure 11**]).

UV-Vis synthesis trace

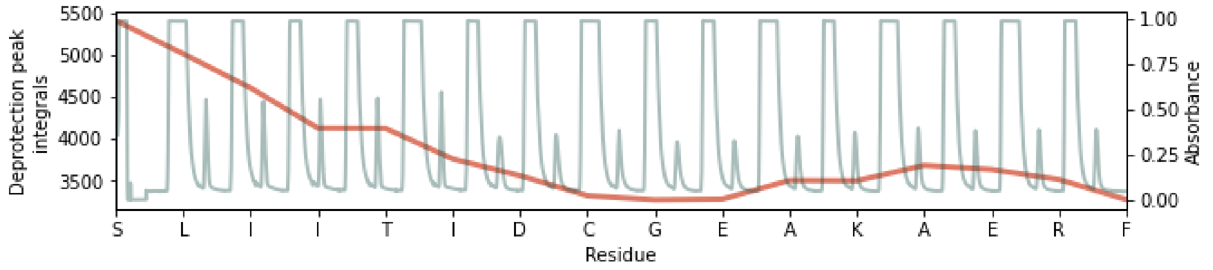

SI Figure 9. UV trace (λ = 310 nm) from AFPS of Barstar[75–90] (green) and deprotection peak integrals (red).

* Takegoshi, K.; Nakamura, S.; Terao, T. 13C–1H Dipolar-Assisted Rotational Resonance in Magic-Angle Spinning NMR. *Chem. Phys. Lett.* **2001**, *344* (5), 631–637. https://doi.org/10.1016/S0009-2614(01)00791-6.

LC-MS of crude Barstar[75–90]

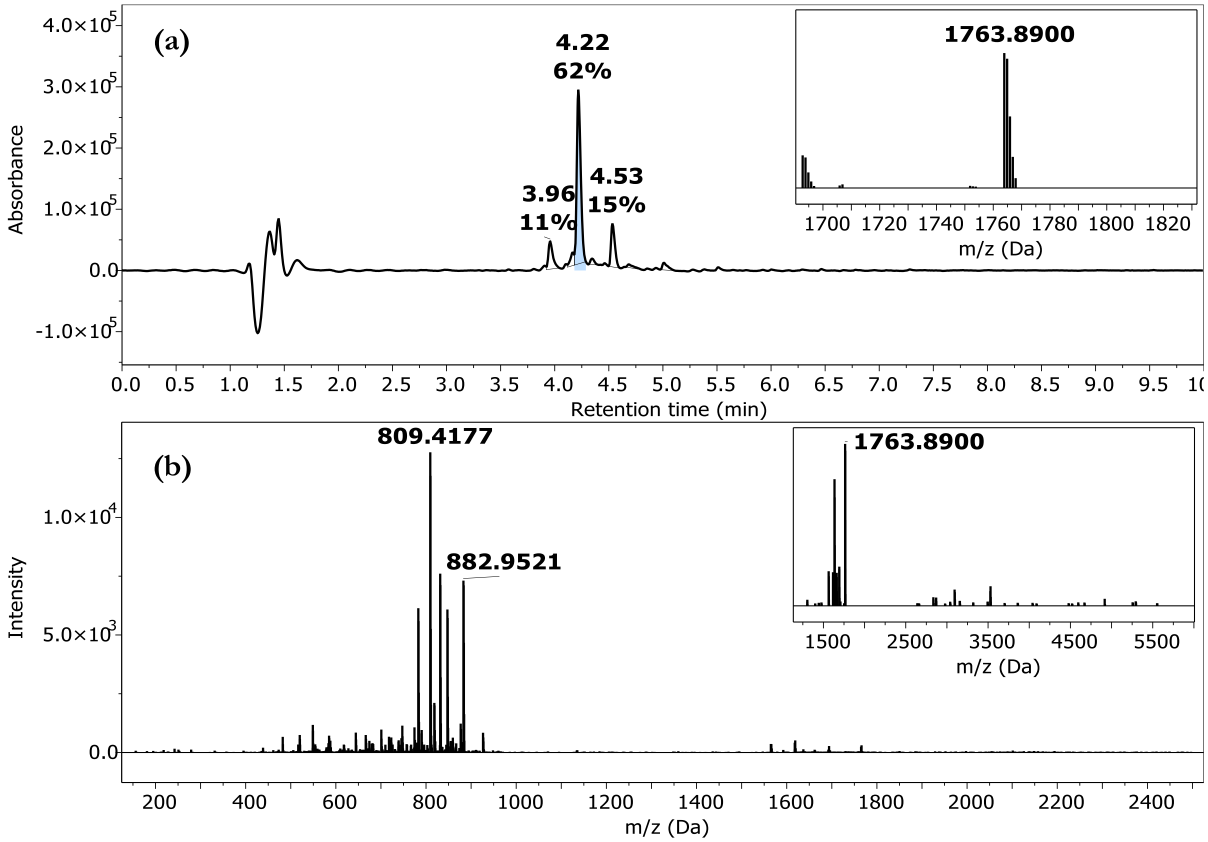

SI Figure 10. LCMS Profile of crude Barstar[75–90]. (**a**) Absorbance chromatogram (λ = 214 nm) of Barstar[75–90] ; Rt 4.22 min, 62% purity. (**b**) ESI-TOF spectrum found within Rt 2–9 min (insert: deconvoluted masses). Monoisotopic mass (ESI+) calcd. for $C_{77}H_{129}N_{21}O_{24}S$ 1763.9240, found 1763.8900. LCMS Gradient A (**Section 2.7**).

UHPLC of crude Barstar[75–90]

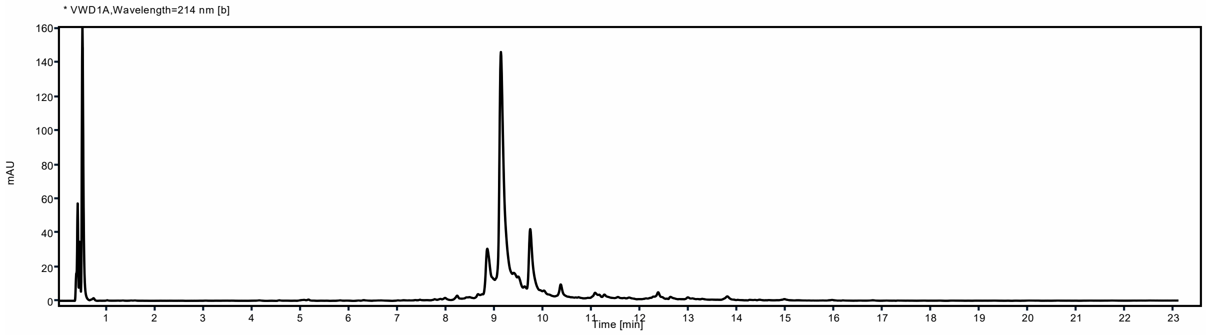

SI Figure 11: UHPLC profile of crude Barstar[75–90]. Rt 9.12 min (Agilent Zorbax 300SB-C18 column, 5 µm, 2.1 × 150 mm, 5–95% MeCN over 20 min, ca. 4.5%B/min), 63% purity based on Area Under Curve (AUC) at λ = 214 nm.

3.1.2 Barstar[75–90]: NBDY6 Tag

```
                   10                   20
H2N-FREAKAEGCD  ITIILSPPPP EK-CONH2        (22 AA)
```

The peptide Barstar[75–90] bearing the NBDY6 tag was synthesized on commercially available Novabiochem® NovaPEG Rink Amide resin (0.41 mmol/g, 51 mg, 21 µmol) using the standard AFPS protocol (**Section 2.2.1**, 40 mL/min flowrate) (**SI Figure 12**). Total synthesis time to afford resin-bound Barstar[75–90]-NBDY6 was approximately 1 h. Cleavage of the peptidyl-resin (16 mg,

approx. 3.3 μmol) according to Cleavage Protocol A (**Section 2.5.1**) afforded the crude peptide (4.0 mg, 46% purity by LCMS [**SI Figure 13**], 50% purity by UHPLC [**SI Figure 14**]).

UV-Vis synthesis trace

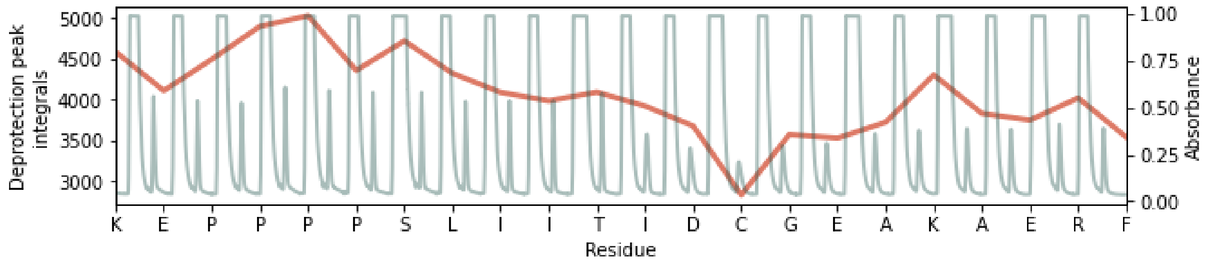

SI Figure 12. UV trace (λ = 310 nm) from AFPS of Barstar[75–90]-NBDY6(green) and deprotection peak integrals (red).

LC-MS of crude Barstar[75–90]-NBDY6

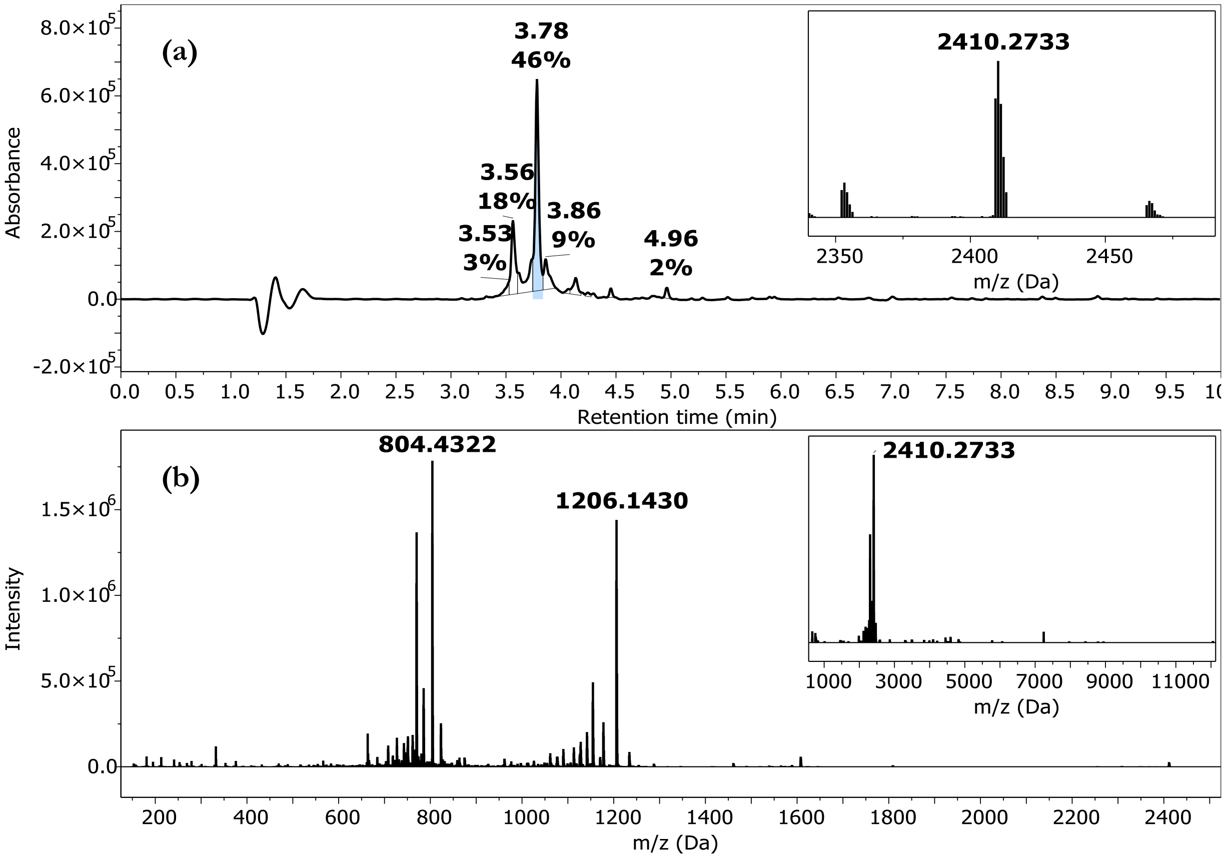

SI Figure 13. LCMS Profile of crude Barstar[75–90] bearing the NBDY6 Tag. (**a**) Absorbance chromatogram (λ = 214 nm) of Barstar[75–90]-NBDY6; Rt 3.78 min, 46% purity. (**b**) ESI-TOF spectrum found within Rt 2–9 min (insert: deconvoluted masses); Monoisotopic mass (ESI+) calcd. for $C_{108}H_{175}N_{27}O_{33}S$ 2409.2726, found 2409.2711. LCMS Gradient A (**Section 2.7**).

UHPLC of crude Barstar[75–90]-NBDY6

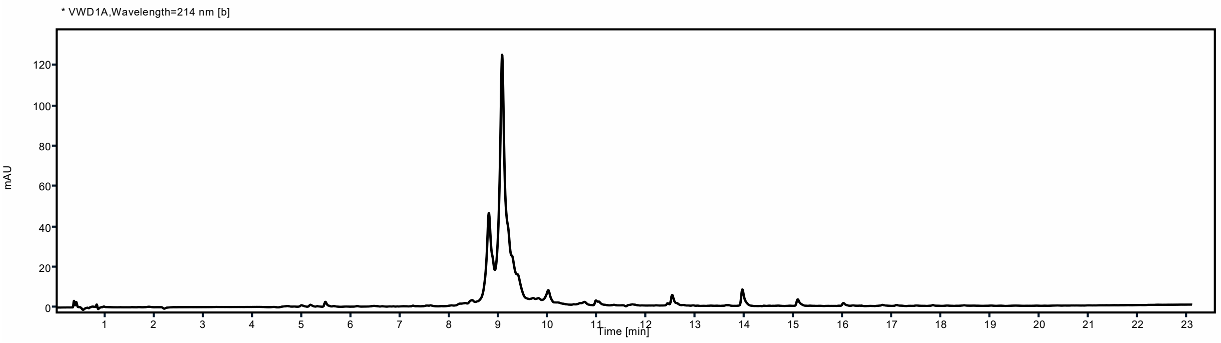

SI Figure 14. UHPLC profile of crude Barstar[75–90] bearing the NBDY6 Tag. Rt 9.01 min (Agilent Zorbax 300SB-C18 column, 5 µm, 2.1 × 150 mm, 5–95% MeCN over 20 min, ca. 4.5%B/min), 50% purity based on Area Under Curve (AUC) at λ = 214 nm.

3.1.3 Barstar[75–90]: NBDY10 Tag

```
                 10             20        26
H2N-FREAKAEGCD  ITIILSLKSH PPPPEK-CONH2   (26 AA)
```

The peptide Barstar[75–90] bearing the NBDY10 tag was synthesized on commercially available Novabiochem® NovaPEG Rink Amide resin (0.41 mmol/g, 51 mg, 21 µmol) using the standard AFPS protocol (**Section 2.2.1**, 40 mL/min flowrate) (**SI Figure 15**). Total synthesis time to afford resin-bound Barstar[75–90]-NBDY10 was approximately 1.2 h. Cleavage of the peptidyl-resin (17 mg, approx. 3.0 µmol) according to Cleavage Protocol A (**Section 2.5.1**) afforded the crude peptide (4.0 mg, 44% purity by LCMS [**SI Figure 16**], 50% purity by UHPLC [**SI Figure 17**]).

UV-Vis synthesis trace

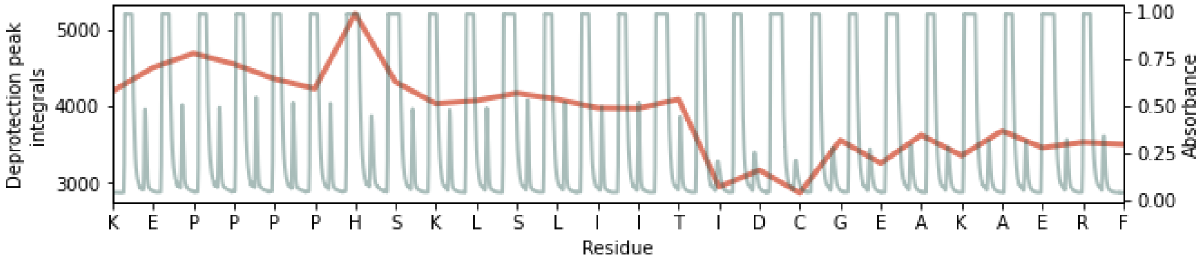

SI Figure 15. UV trace (λ = 310 nm) from AFPS of Barstar[75–90]-NBDY10 (green) and deprotection peak integrals (red).

LC-MS of crude Barstar[75–90]-NBDY10

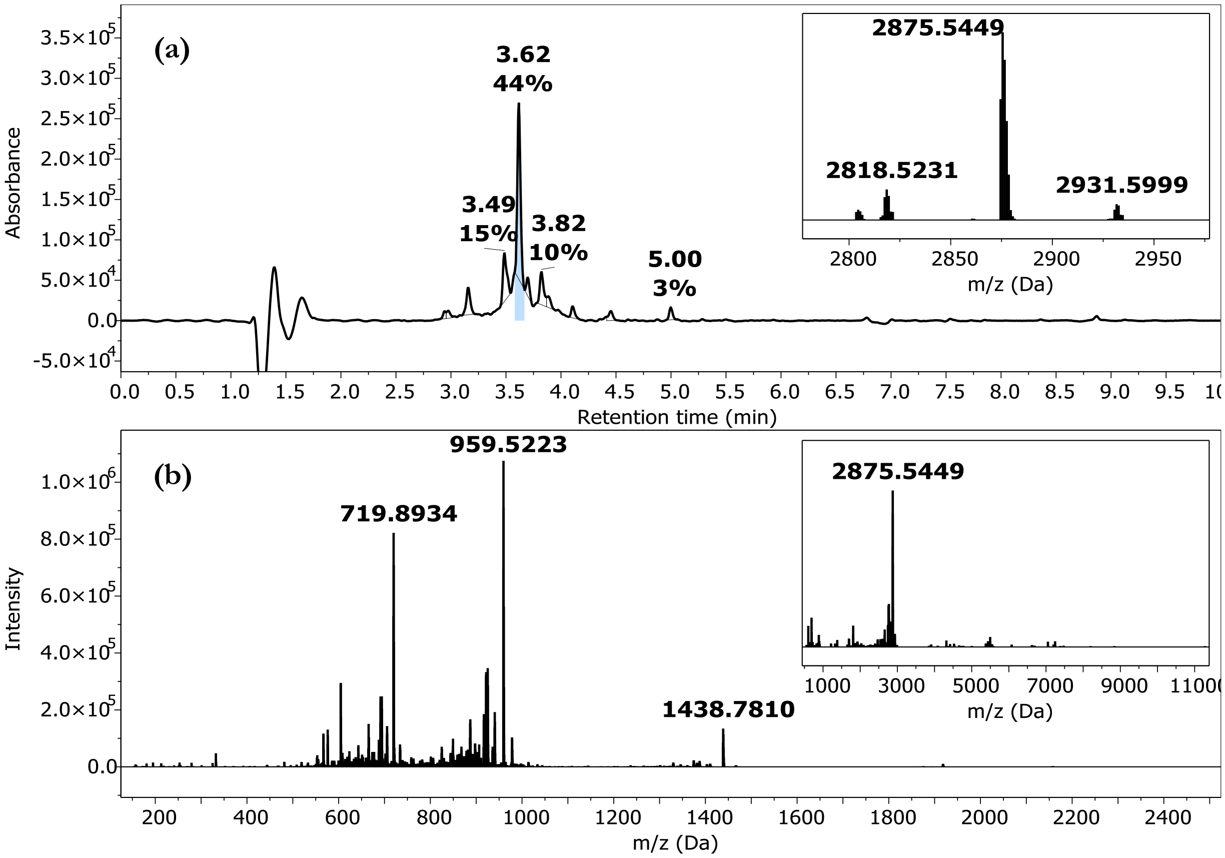

SI Figure 16. LCMS Profile of crude Barstar[75–90] bearing the NBDY10 Tag. (**a**) Absorbance chromatogram (λ = 214 nm) of Barstar[75–90]-NBDY10; Rt 3.62 min, 44% purity. (**b**) ESI-TOF spectrum found within Rt 2–9 min (insert: deconvoluted masses). Monoisotopic mass (ESI+) calcd. for $C_{129}H_{211}N_{35}O_{37}S$ 2874.5426, found 2874.5411. LCMS Gradient A (**Section 2.7**).

UHPLC of crude Barstar[75–90]-NBDY10

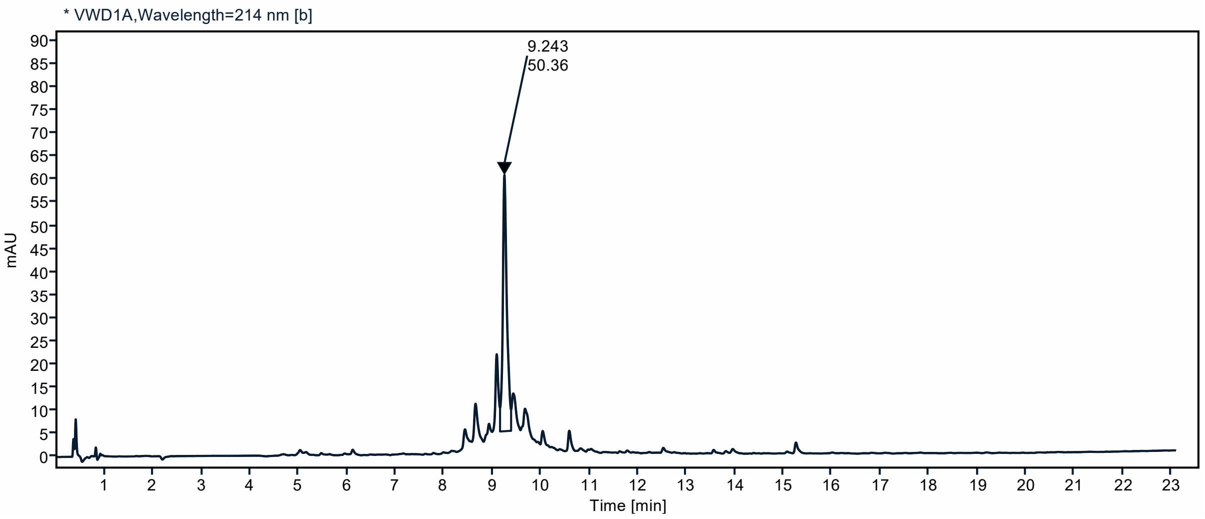

SI Figure 17. UHPLC profile of crude Barstar[75–90] bearing the NBDY 10-mer Tag. Rt 9.24 min (Agilent Zorbax 300SB-C18 column, 5 µm, 2.1 × 150 mm, 5–95% MeCN over 20 min, ca. 4.5%B/min), 50% purity based on Area Under Curve (AUC) at λ = 214 nm.

3.1.4 Barstar[75–90]: [Lys(Boc)]$_6$-Tag

```
          10                  20
H2N-FREAKAEGCD  ITIILSKKKK KK-CONH2          (22 AA)
```

The peptide Barstar[75–90] bearing the [Lys(Boc)]$_6$ tag was synthesized on commercially available Novabiochem® NovaPEG Rink Amide resin (0.41 mmol/g, 51 mg, 21 µmol) using the standard AFPS protocol (**Section 2.2.1**, 40 mL/min flowrate) (**SI Figure 18**). Total synthesis time to afford resin-bound Barstar[75–90]-[Lys(Boc)]$_6$ was approximately 1 h. Cleavage of the peptidyl-resin (18 mg, 3.3 µmol) according to Cleavage Protocol A (**Section 2.5.1**) afforded the crude peptide (4.5 mg, 51% purity by LCMS [**SI Figure 19**], 52% purity by UHPLC [**SI Figure 20**]).

UV-Vis synthesis trace

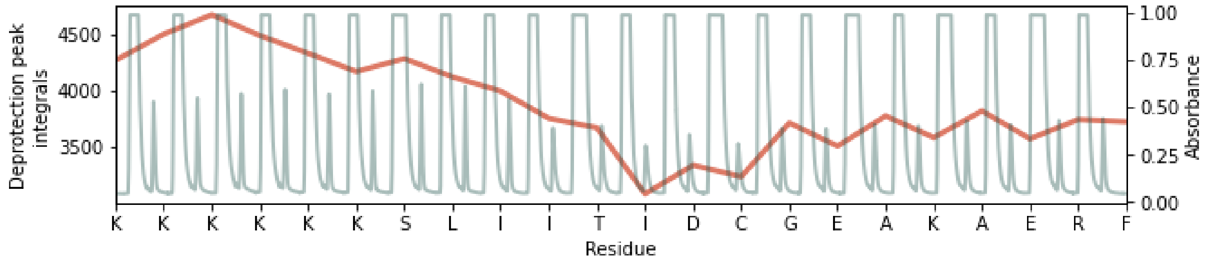

SI Figure 18. UV trace (λ = 310 nm) from AFPS of Barstar[75–90]-[Lys(Boc)]$_6$ (green) and deprotection peak integrals (red).

LC-MS of crude Barstar[75–90]-(Lys)$_6$

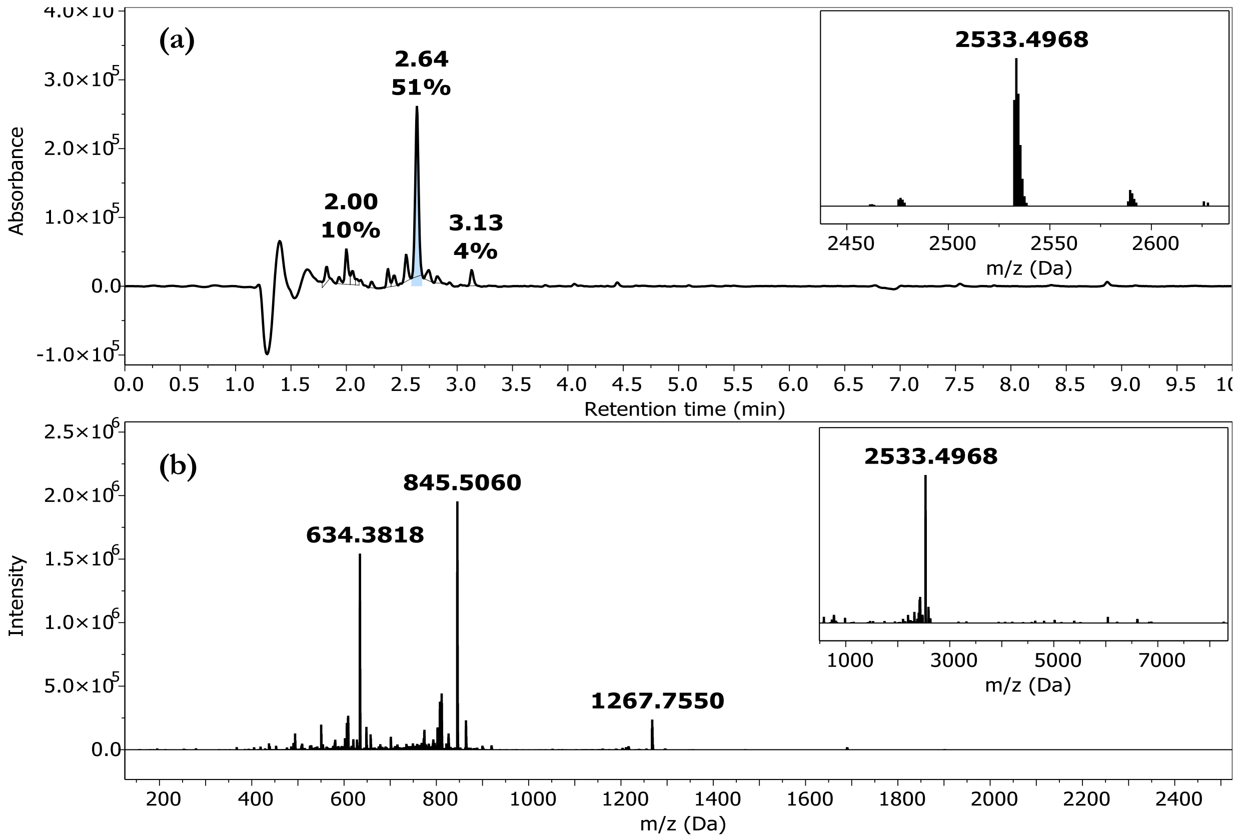

SI Figure 19. LCMS Profile of crude Barstar[75–90] bearing (Lys)$_6$. (**a**) Absorbance chromatogram (λ = 214 nm) of Barstar[75–90]-(Lys)$_6$ tag; Rt 2.64 min, 51% purity. (**b**) ESI-TOF spectrum found within Rt 2–9 min (insert: deconvoluted masses). Monoisotopic mass (ESI+) calcd. for $C_{113}H_{201}N_{33}O_{30}S$ 2532.4938, found 2532.4936. LCMS Gradient A (**Section 2.7**).

UHPLC of crude Barstar[75–90]-(Lys)$_6$

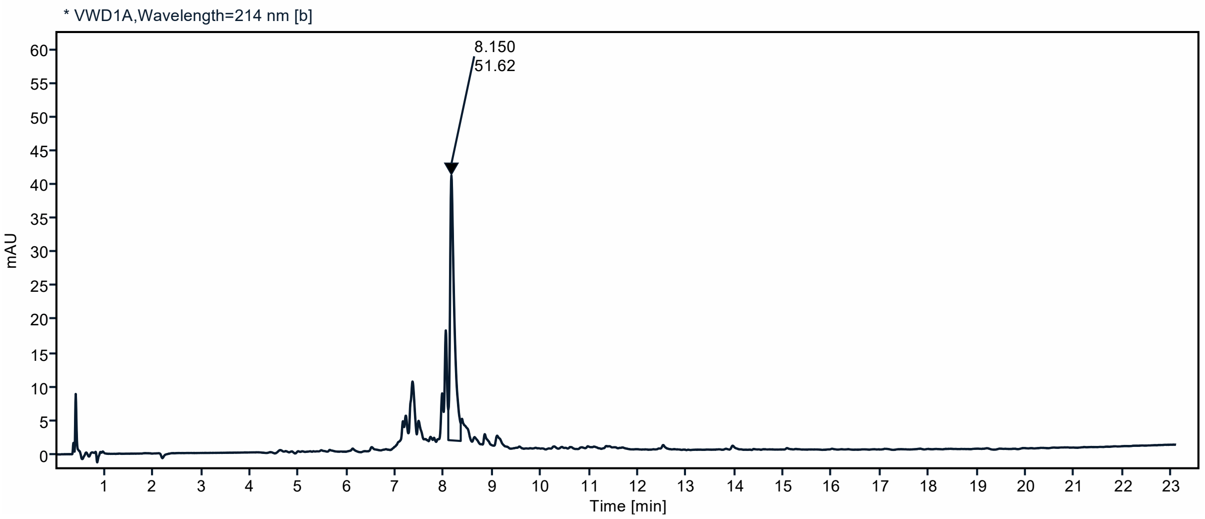

SI Figure 20. UHPLC profile of crude Barstar[75–90] bearing (Lys)$_6$. Rt 8.15 min (Agilent Zorbax 300SB-C18 column, 5 µm, 2.1 × 150 mm, 5–95% MeCN over 20 min, ca. 4.5%B/min), 52% purity based on Area Under Curve (AUC) at λ = 214 nm.

3.1.5 Barstar[75–90]: (Ala)$_6$-Tag

```
               10                  20
H2N-FREAKAEGCD  ITIILSAAAA AA-CONH2         (22 AA)
```

The peptide Barstar[75–90] bearing the (Ala)$_6$ tag was synthesized on commercially available Novabiochem® NovaPEG Rink Amide resin (0.41 mmol/g, 51 mg, 21 µmol) using the standard AFPS protocol (**Section 2.2.1**, 40 mL/min flowrate) (**SI Figure 21**). Total synthesis time to afford resin-bound Barstar[75–90]-(Ala)$_6$ was approximately 1 h. Cleavage of the peptidyl-resin (16 mg, approx. 3.0 µmol) according to Cleavage Protocol A (**Section 2.5.1**) afforded the crude peptide (2.8 mg, 7% purity by LCMS[**SI Figure 22**], 34% purity by UHPLC [**SI Figure 23**]).

UV-Vis synthesis trace

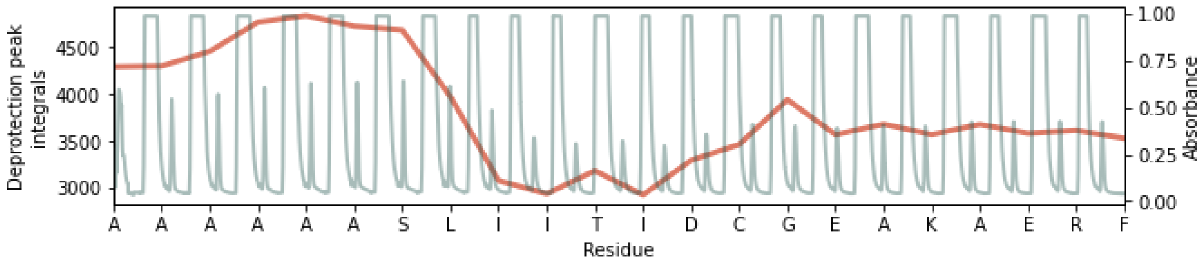

SI Figure 21. UV trace (λ = 310 nm) from AFPS of Barstar[75–90]-(Ala)$_6$ (green) and deprotection peak integrals (red).

LC-MS of crude Barstar[75–90]-(Ala)$_6$

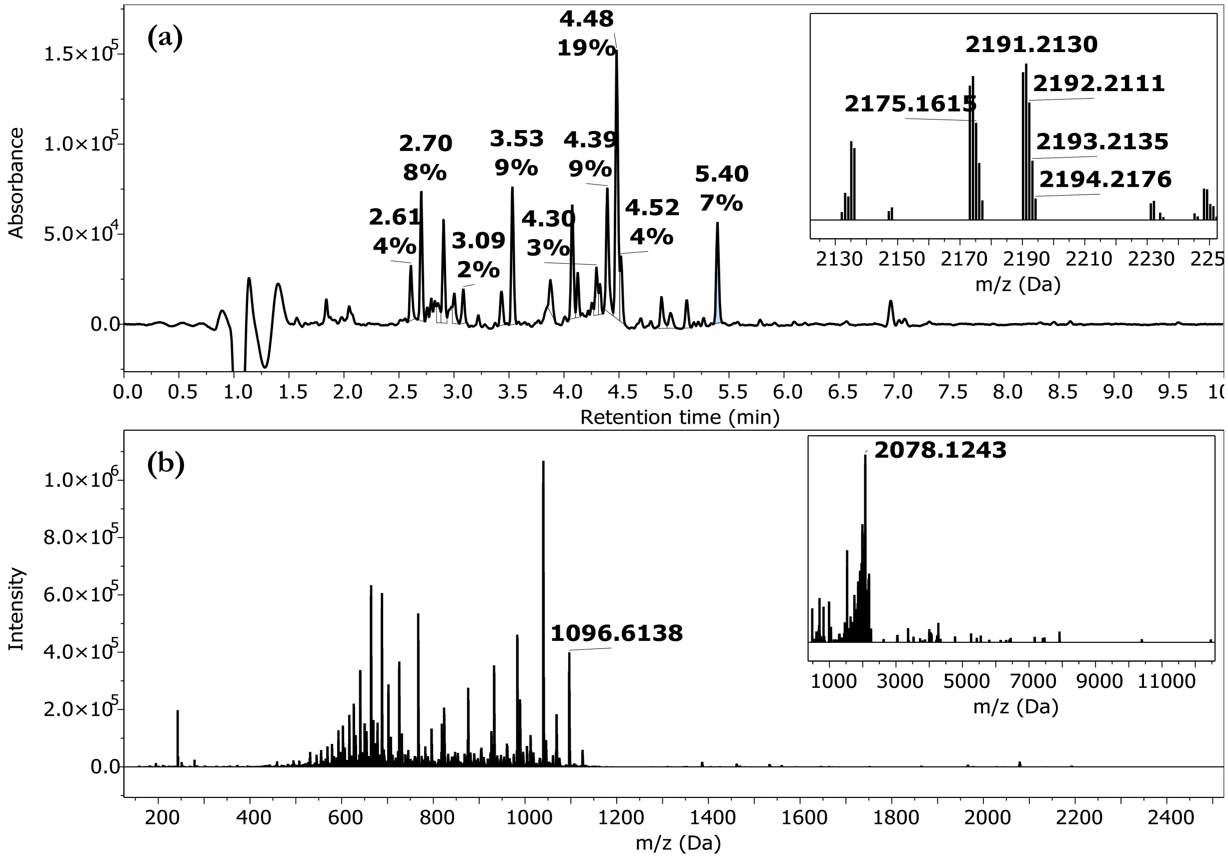

SI Figure 22. LCMS Profile of crude Barstar[75–90] bearing (Ala)$_6$. (**a**) Absorbance chromatogram (λ = 214 nm) of Barstar[75–90]-(Ala)$_6$; Rt 5.40 min, 7% purity. (**b**) ESI-TOF spectrum found within Rt 2–9 min (insert: deconvoluted masses). Monoisotopic mass (ESI+) calcd. for $C_{95}H_{159}N_{27}O_{30}S$ 2190.1467, found 2190.2101. LCMS Gradient A (**Section 2.7**).

UHPLC of crude Barstar[75–90]-(Ala)$_6$

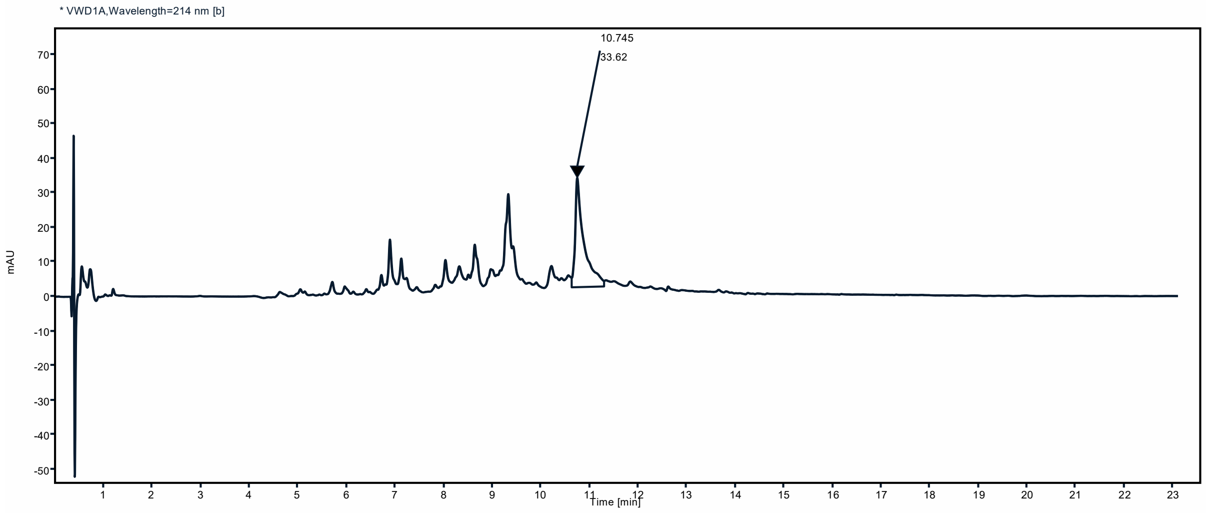

SI Figure 23. UHPLC profile of crude Barstar[75–90] bearing (Ala)$_6$. Rt 10.75 min (Agilent Zorbax 300SB-C18 column, 5 µm, 2.1 × 150 mm, 5–95% MeCN over 20 min, ca. 4.5%B/min), 34% purity based on Area Under Curve (AUC) at λ = 214 nm.

3.1.6 Barstar[75–90]: (Pro)$_6$-Tag

```
            10                  20
H2N-FREAKAEGCD  ITIILSPPPP PP-CONH2         (22 AA)
```

The peptide Barstar[75–90] bearing the (Pro)$_6$ tag was synthesized on commercially available Novabiochem® NovaPEG Rink Amide resin (0.41 mmol/g, 50 mg, 21 µmol) using the standard AFPS protocol (**Section 2.2.1**, 40 mL/min flowrate) (**SI Figure 24**). Total synthesis time to afford resin-bound Barstar[75–90]-(Pro)$_6$ was approximately 1 h. Cleavage of the peptidyl-resin (11 mg, 2.6 µmol) according to Cleavage Protocol A (**Section 2.5.1**) afforded the crude peptide (3.0 mg, 56% purity by LCMS [**SI Figure 25**], 58% purity by UHPLC [**SI Figure 26**]).

UV-Vis synthesis trace

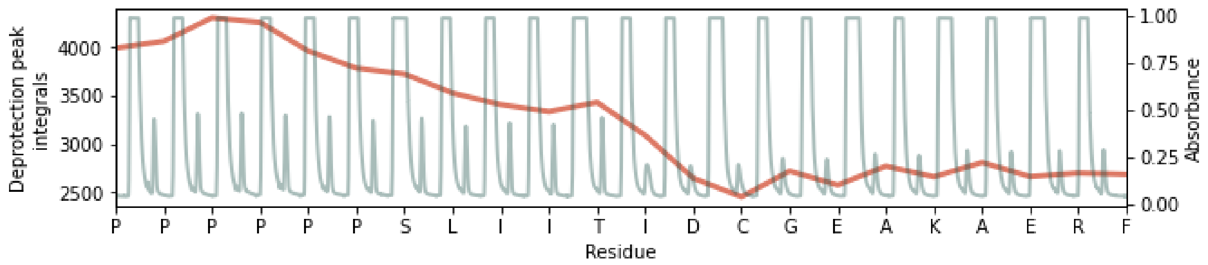

SI Figure 24. UV trace (λ = 310 nm) from AFPS of Barstar[75–90]-(Pro)$_6$ (green) and deprotection peak integrals (red).

UHPLC of crude Barstar[75–90]-(Pro)$_6$

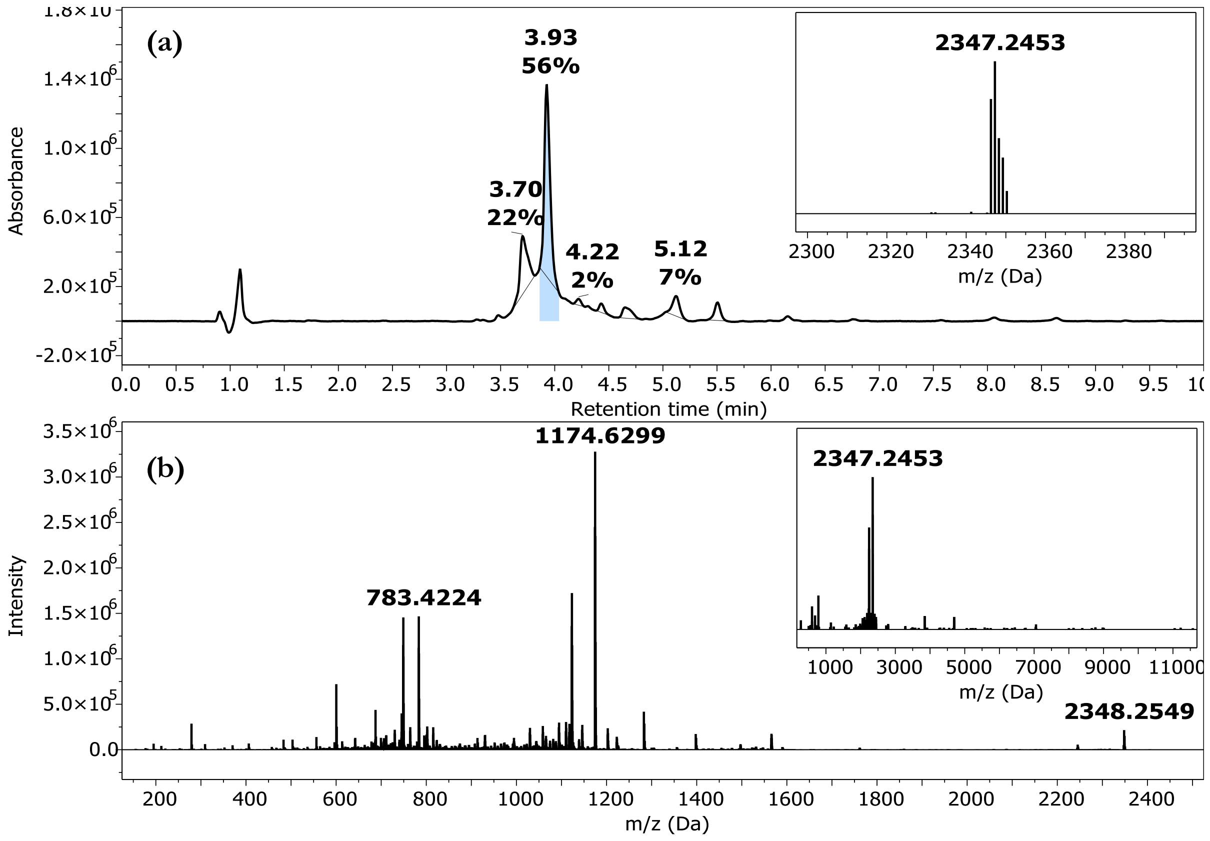

SI Figure 25. LCMS Profile of crude Barstar[75–90] bearing (Pro)$_6$. (**a**) Absorbance chromatogram (λ = 214 nm) of Barstar[75–90]-(Pro)$_6$; Rt 3.93 min, 56% purity. (**b**) ESI-TOF spectrum found within Rt 2–9 min (insert: deconvoluted masses). Monoisotopic mass (ESI+) calcd. for $C_{107}H_{171}N_{27}O_{30}S$ 2346.2406, found 2346.2428. LCMS Gradient A (**Section 2.7**).

UHPLC of crude Barstar[75–90]-(Pro)$_6$

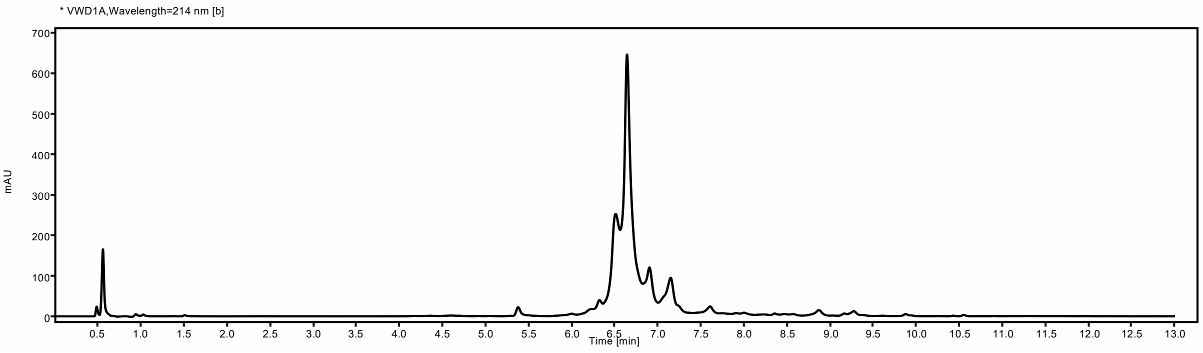

SI Figure 26. UHPLC profile of crude Barstar[75–90] bearing (Pro)$_6$. Rt 6.64 min (Agilent Zorbax 300SB-C18 column, 5 µm, 2.1 × 150 mm, 5–95% MeCN over 10 min, ca. 9%B/min), 58% purity based on Area Under Curve (AUC) at λ = 214 nm.

3.1.7 Barstar[75–90]: [Arg(Pbf)]$_6$-Tag

```
                     10          20
H2N-FREAKAEGCD  ITIILSRRRR RR-CONH2        (22 AA)
```

The peptide Barstar[75–90] bearing the [Arg(Pbf)]$_6$ tag was synthesized on commercially available Novabiochem® NovaPEG Rink Amide resin (0.41 mmol/g, 51 mg, 21 µmol) using the standard AFPS protocol (**Section 2.2.1**, 40 mL/min flowrate) (**SI Figure 27**). Total synthesis time to afford resin-bound Barstar[75–90]-[Arg(Pbf)]$_6$ was approximately 1 h. Cleavage of the peptidyl-resin (18 mg, approx. 2.7 µmol) according to Cleavage Protocol A (**Section 2.5.1**) afforded the crude peptide (3.2 mg, 81% purity by LCMS [**SI Figure 28**], 70% purity by UHPLC [**SI Figure 29**]).

UV-Vis synthesis trace

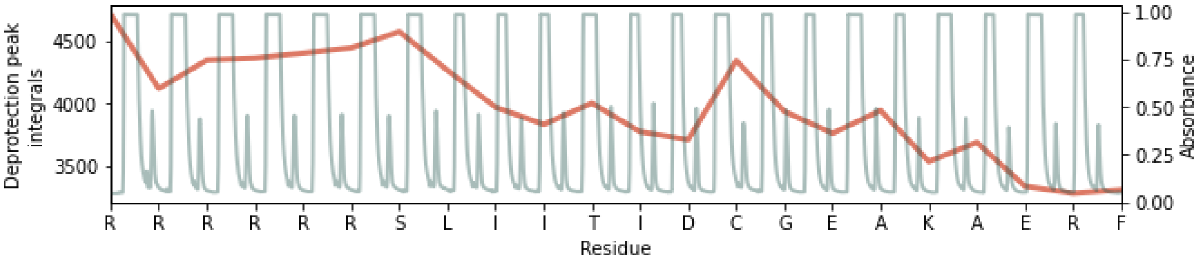

SI Figure 27. UV trace (λ = 310 nm) from AFPS of Barstar[75–90]-[Arg(Pbf)]$_6$ (green) and deprotection peak integrals (red).

LC-MS of crude Barstar[75–90]-(Arg)$_6$

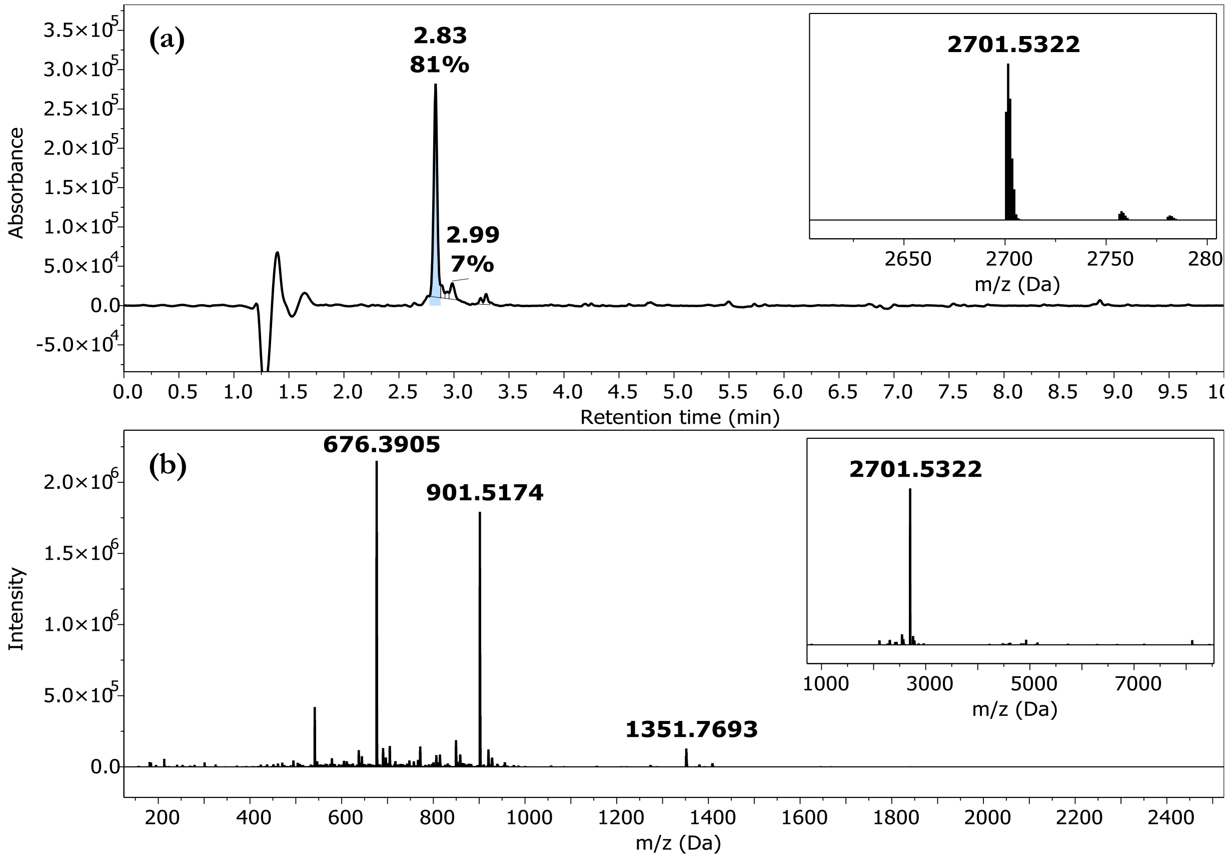

SI Figure 28. LCMS Profile of Barstar[75–90] bearing (Arg)$_6$. (**a**) Absorbance chromatogram (λ = 214 nm) of Barstar[75–90]-(Arg)$_6$; Rt 2.83 min, 81% purity. (**b**) ESI-TOF spectrum found within Rt 2–9min (insert: deconvoluted masses). Monoisotopic mass (ESI+) calcd. for $C_{113}H_{200}N_{44}O_{31}S$ 2700.5307, found 2700.5290. LCMS Gradient A (**Section 2.7**).

UHPLC of crude Barstar[75–90]-(Arg)$_6$

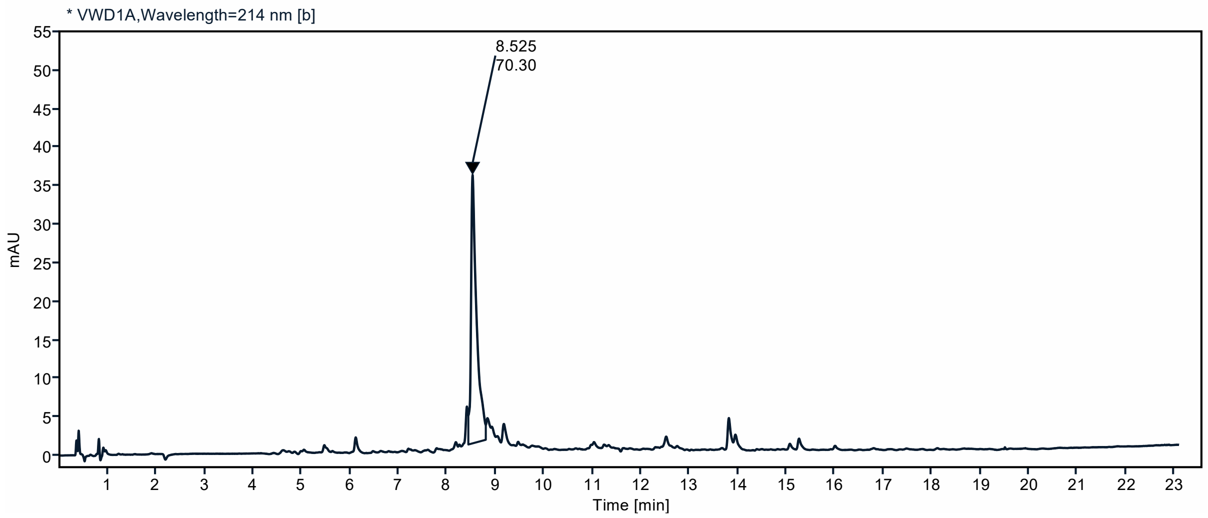

SI Figure 29. UHPLC profile of crude Barstar[75–90] bearing (Arg)$_6$. Rt 8.53 min (Agilent Zorbax 300SB-C18 column, 5 µm, 2.1 × 150 mm, 5–95% MeCN over 20 min, ca. 4.5%B/min), 70% purity based on Area Under Curve (AUC) at λ = 214 nm.

3.1.8 Barstar[75–90]: [Gln(Trt)]$_6$-Tag

```
              10          20
H2N-FREAKAEGCD  ITIILSQQQQ  QQ-CONH2        (22 AA)
```

The peptide Barstar[75–90] bearing the [Gln(Trt)]$_6$ tag was synthesized on commercially available Novabiochem® NovaPEG Rink Amide resin (0.41 mmol/g, 50 mg, 21 µmol) using the standard AFPS protocol (**Section 2.2.1**, 20 mL/min flowrate) (**SI Figure 30**). Total synthesis time to afford resin-bound Barstar[75–90]-[Gln(Trt)]$_6$ was approximately 1 h. Cleavage of the peptidyl-resin (19.5 mg, approx. 3.1 µmol) according to Cleavage Protocol A (**Section 2.5.1**) afforded the crude peptide (5.7 mg, LCMS [**SI Figure 31**], 50% purity by UHPLC [**SI Figure 32**]).

UV-Vis synthesis trace

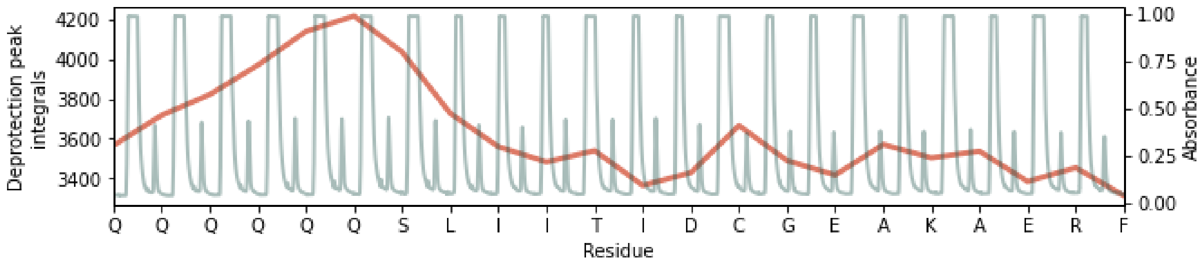

SI Figure 30. UV trace (λ = 310 nm) from AFPS of Barstar[75–90]-[Gln(Trt)]$_6$ (green) and deprotection peak integrals (red).

LC-QTOF of crude Barstar[75–90]-(Gln)$_6$

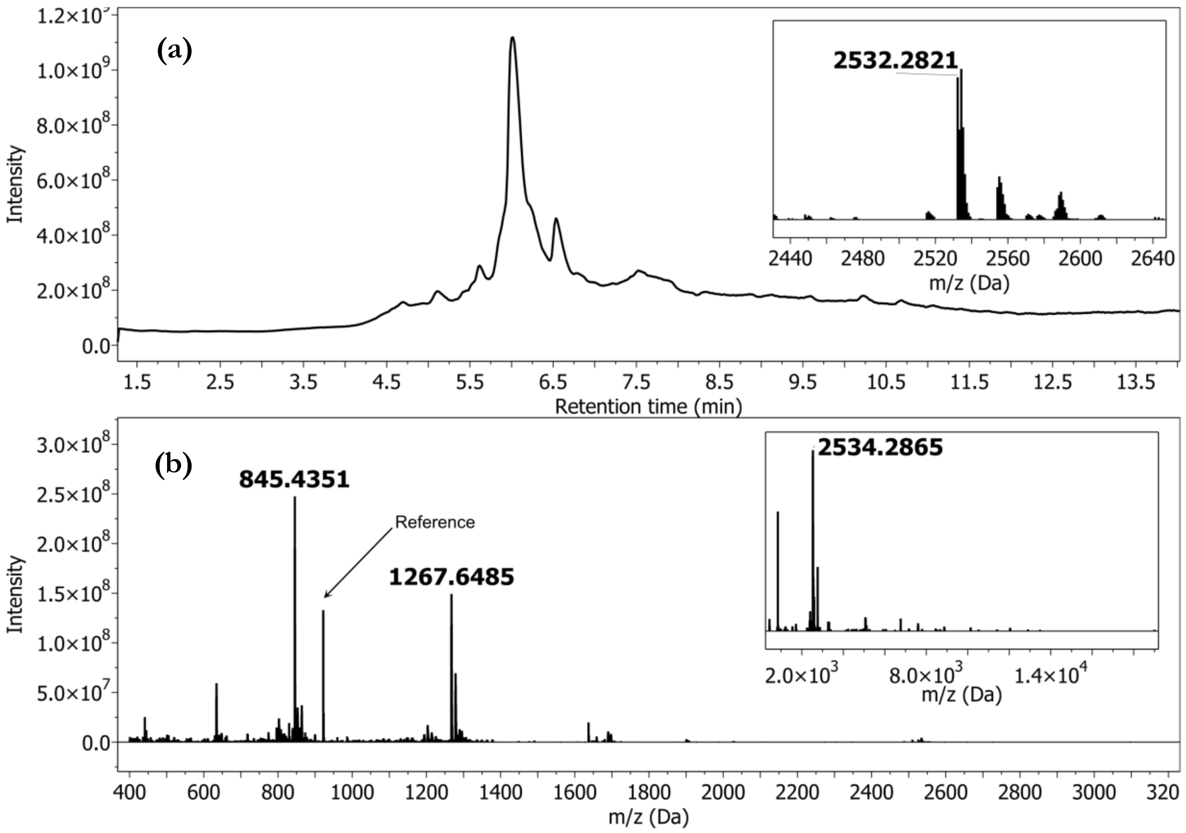

SI Figure 31. LC-QTOF Profile of Barstar[75–90] bearing (Gln)$_6$. (**a**) HRMS (ESI-qTOF) total ion count (TIC) of Barstar[75–90]-(Asn)$_6$; Rt 3.40 min, 50% purity. (**b**) ESI-qTOF spectrum found within Rt 2–9min (insert: deconvoluted masses). Monoisotopic mass (ESI+) calcd. for $C_{107}H_{176}N_{32}O_{37}S$ 2532.2755, found 22532.2821.The samples was analyzed on an Agilent 1290 Infinity II Series connected to an Agilent LC/Q-TOF.

UHPLC of crude Barstar[75–90]-(Gln)$_6$

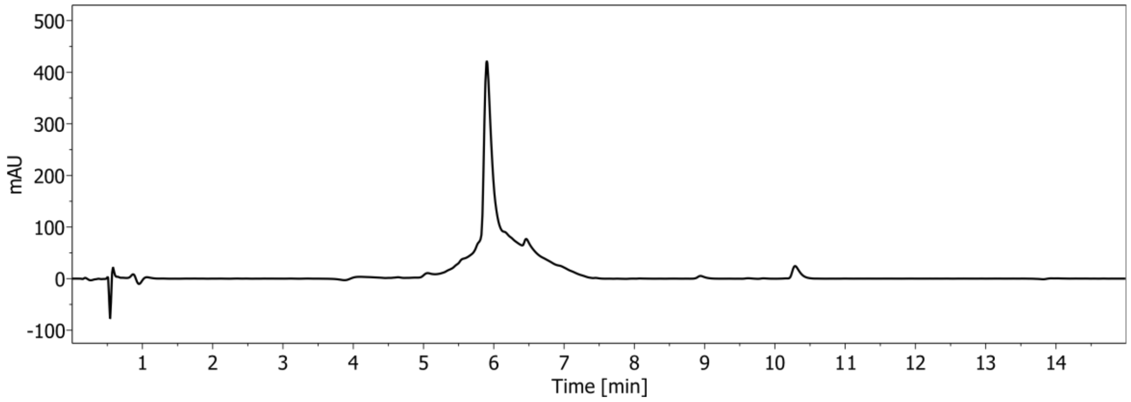

SI Figure 32. UHPLC profile of crude Barstar[75–90] bearing (Gln)$_6$. Rt 5.90 min (Agilent Zorbax 300SB-C18 column, 5 µm, 2.1 × 150 mm, 5–95% MeCN over 10 min, ca. 9%B/min), 47% purity based on Area Under Curve (AUC) at λ = 214 nm. *1% Formic acid was used as a counter ion instead of TFA.

3.1.9 Cumulative synthesis traces of Barstar[75–90]

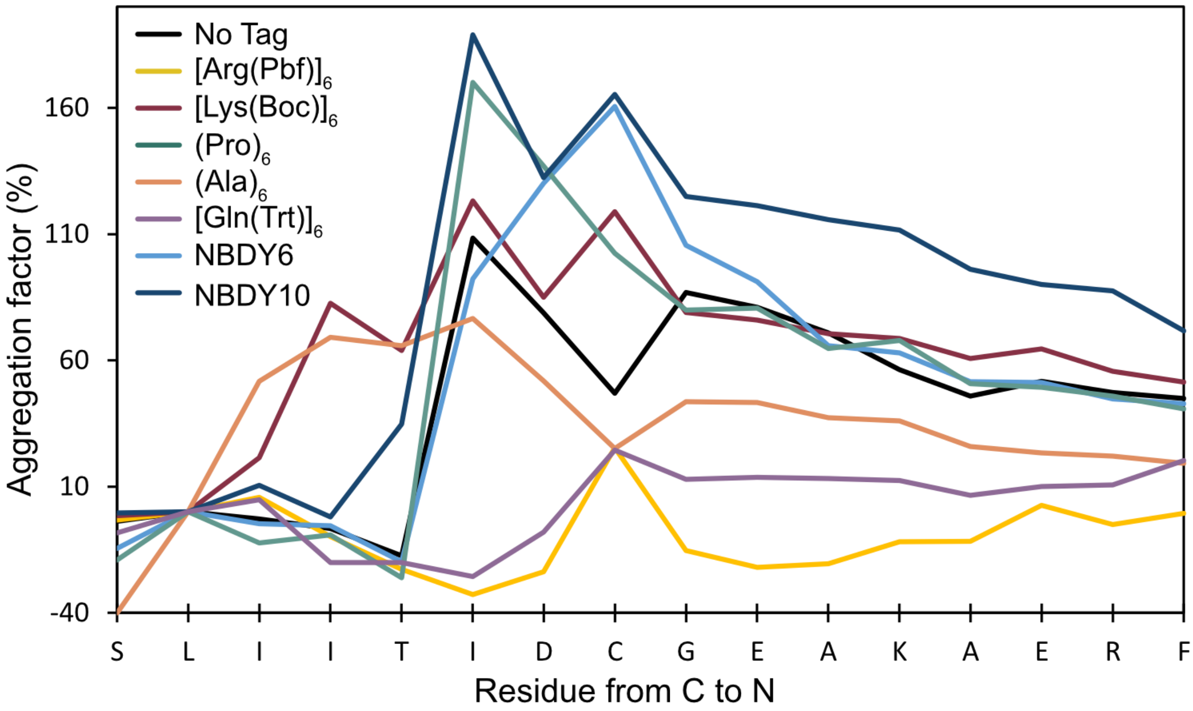

SI Figure 33. Aggregation as a function of Fmoc-deprotection peak broadening by in-line UV-Vis (310 nm) in flow-SPPS for various amino acid tags, normalized at Leu[89].

3.2 Evaluation of [Arg(Pbf)]$_6$-Tag: GLP-1

3.2.1 GLP-1: No tag (reference)

```
             10                20                30
H2N-HAEGTFTSDV   SSYLEGQAAK  EFIAWLVKGR-CONH2    (30 AA)
```

The peptide GLP-1 was synthesized on commercially available Novabiochem® NovaPEG Rink Amide resin (0.41 mmol/g, 53 mg, 22 µmol) using the standard AFPS protocol (**Section 2.2.1**, 40 mL/min flowrate) (**SI Figure 34**). Total synthesis time to afford resin-bound GLP-1 was approximately 1.5 h. Cleavage of the peptidyl-resin (59 mg, approx. 24 µmol), according to Cleavage

Protocol A (**Section 2.5.1**) afforded the crude peptide (16 mg, 70% purity by LCMS [**SI Figure 35**], 73% purity by UHPLC [**SI Figure 36**]).

UV-Vis synthesis trace

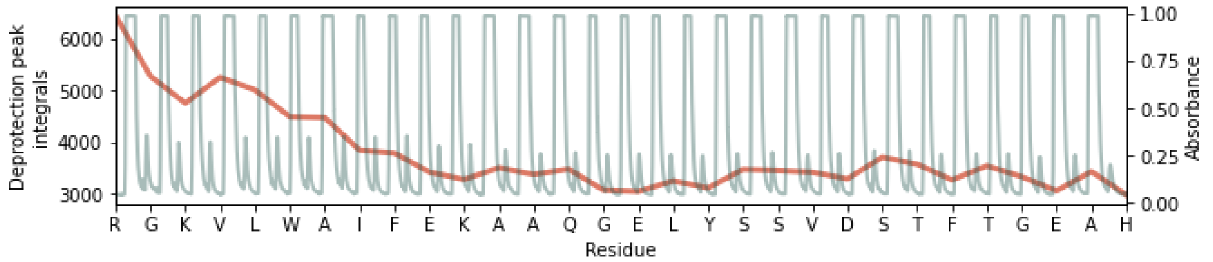

SI Figure 34: UV trace (λ = 310 nm) from AFPS of GLP-1 synthesized on Rink Amide resin (green) and deprotection peak integrals (red).

LC-MS of crude GLP-1

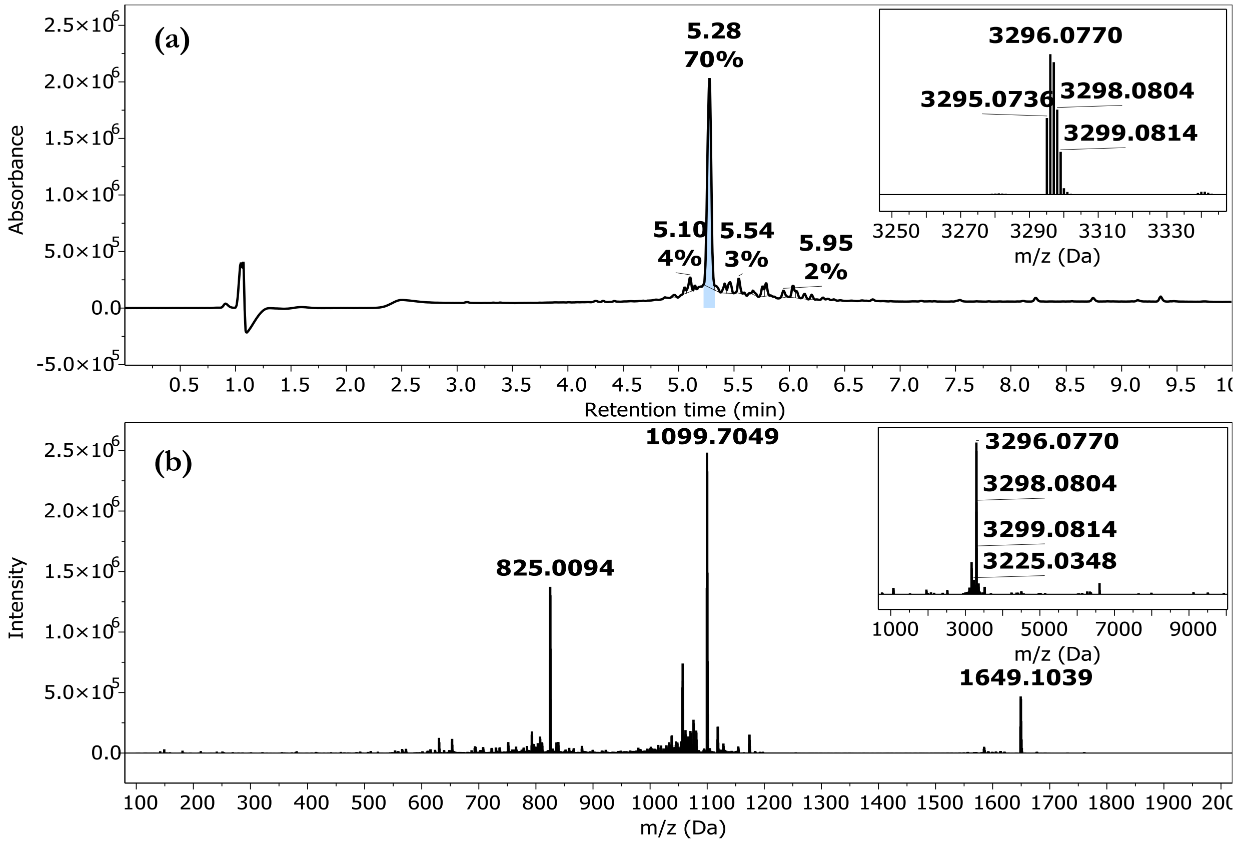

SI Figure 35: LC-MS Profile of crude GLP-1. (**a**) Absorbance chromatogram (λ = 214 nm) of peptide GLP-1; Rt 5.28 min, 70% purity. (**b**) ESI-TOF spectrum found within Rt 2–9 min (insert: deconvoluted masses); Monoisotopic mass (ESI+) calcd. for $C_{149}H_{226}N_{40}O_{45}$ 3295.6626, found 3295.0736. LCMS Gradient A (**Section 2.7**).

UHPLC of crude GLP-1

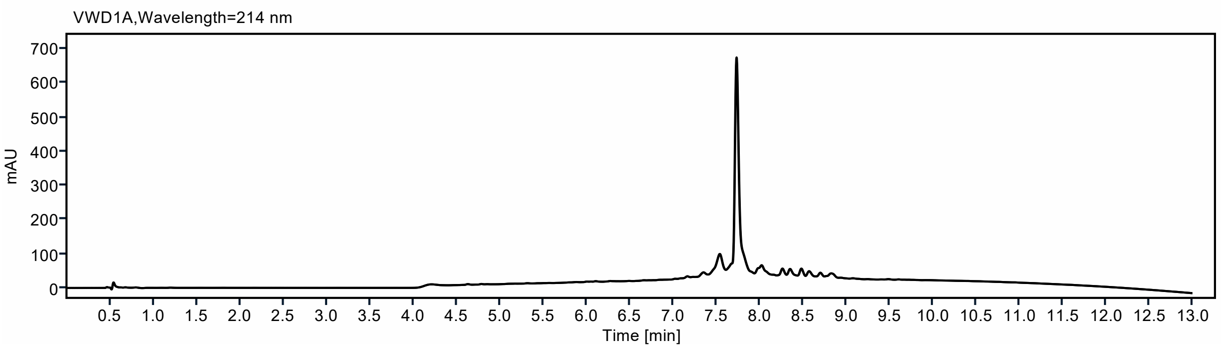

SI Figure 36: UHPLC profile of crude GLP-1. Rt 7.74 min (Agilent Zorbax 300SB-C18 column, 5 µm, 2.1 × 150 mm, 5–95% MeCN over 10 min, ca. 9%B/min), 73% purity based on Area Under Curve (AUC) at λ = 214 nm.

3.2.2 GLP-1: [Arg(Pbf)]$_6$- Tag

```
                 10                20                   30          36
H2N-HAEGTFTSDV   SSYLEGQAAK   EFIAWLVKGR RRRRRR-CONH2        (36 AA)
```

The peptide GLP-1-[Arg(Pbf)]$_6$ was synthesized on commercially available Novabiochem® NovaPEG Rink Amide resin (0.41 mmol/g, 52 mg, 21 µmol) using the standard AFPS protocol (**Section 2.2.1**, 40 mL/min flowrate) (**SI Figure 37**). Total synthesis time to afford resin-bound GLP-1-[Arg(Pbf)]$_6$ was approximately 2 h. Cleavage of the peptidyl-resin (99 mg, approx. 41 µmol), according to Cleavage Protocol A (**Section 2.5.1**) afforded the crude peptide (18 mg, 78% purity by LCMS [**SI Figure 38**], 84% purity by UHPLC [**SI Figure 39**]).

UV-Vis synthesis trace

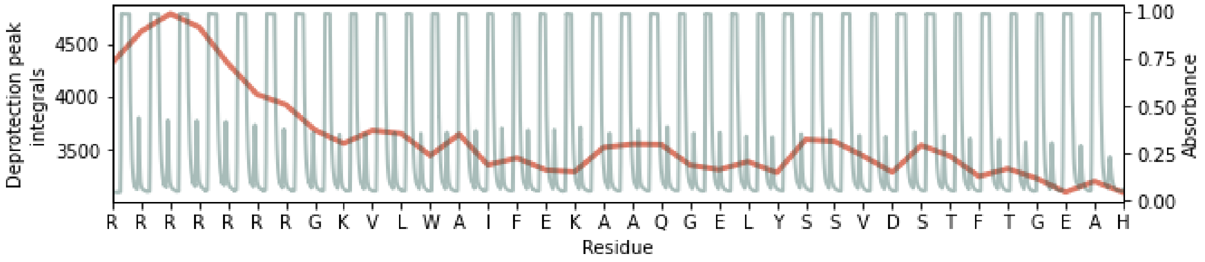

SI Figure 37. UV trace (λ = 310 nm) from AFPS of GLP-1 bearing the [Arg(Pbf)]$_6$ at the C-terminus, synthesized on Rink Amide resin (green) and deprotection peak integrals (red).

LC-MS of crude GLP-1-(Arg)$_6$

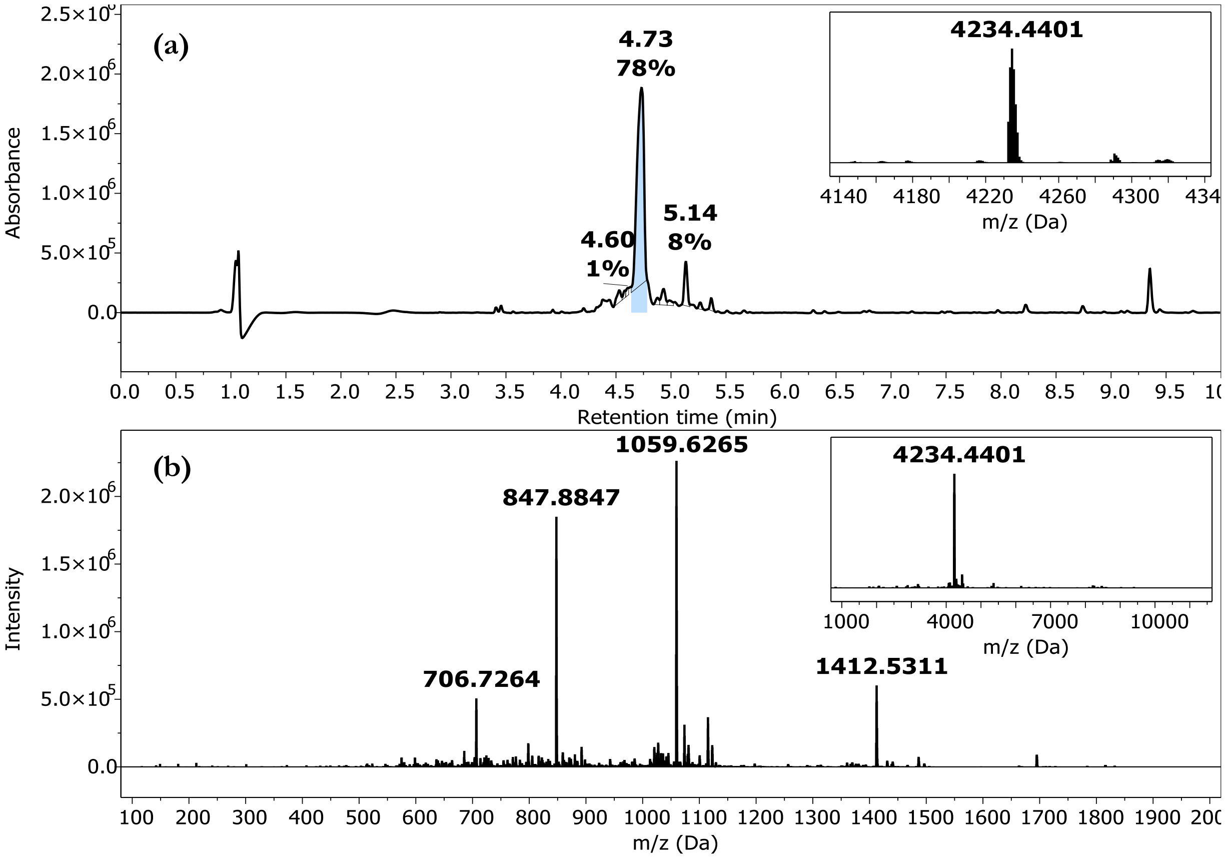

SI Figure 38: LCMS Profile of crude GLP-1 bearing (Arg)$_6$. (**a**) Absorbance chromatogram (λ = 214 nm) of peptide GLP-1-(Arg)$_6$; Rt 4.73 min, 78% purity. (**b**) ESI-TOF spectrum found within Rt 2–9 min (insert: deconvoluted masses); Monoisotopic mass (ESI+) calcd. for $C_{185}H_{298}N_{64}O_{51}$ 4232.2692, found 4232.4347. LCMS Gradient A (**Section 2.7**).

UHPLC of crude GLP-1-(Arg)$_6$

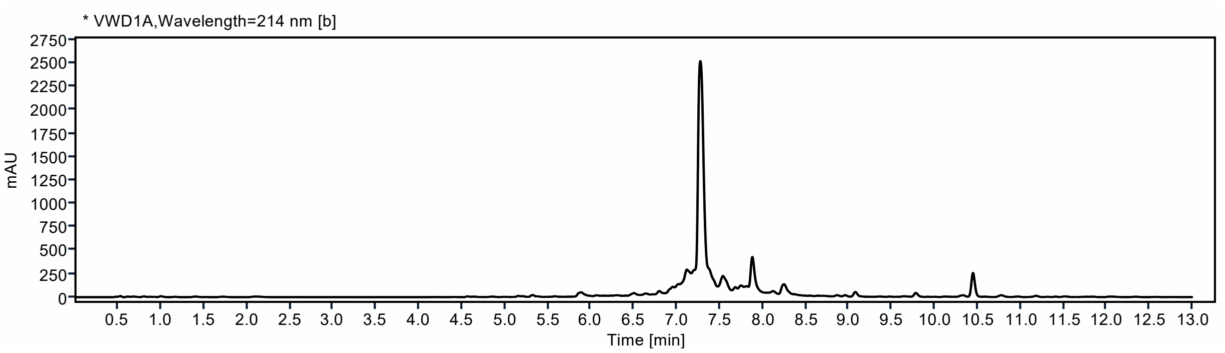

SI Figure 39: UHPLC profile of crude GLP-1 bearing (Arg)$_6$ at the C-terminus. Rt 7.272 min (Agilent Zorbax 300SB-C18 column, 5 µm, 2.1 × 150 mm, 5–95% MeCN over 10 min, ca. 9%B/min), 84% purity based on Area Under Curve (AUC) at λ = 214 nm.

3.2.3 Cumulative synthesis traces of GLP-1

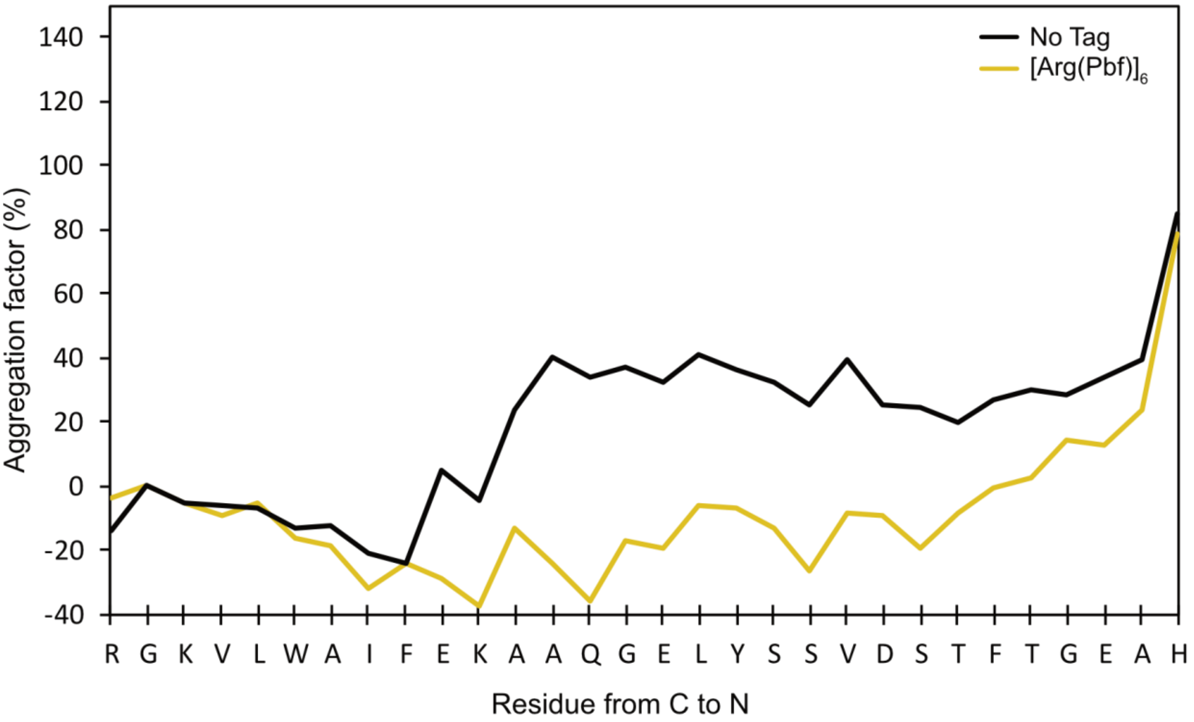

SI Figure 40. Aggregation as a function of Fmoc-deprotection peak broadening by in-line UV-Vis (310 nm) in flow-SPPS for GLP-1 with and without [Arg(Pbf)]$_6$ tag, normalized at Gly[29].

3.3 Evaluation of [Arg(Pbf)]$_6$-Tag: hGH[176–191]

3.3.1 hGH[176–191](F176Y): No tag (reference)

```
            10        16
H2N-YLRIVQCRSV EGSCGF -CONH2     (16 AA)
```

The peptide hGH[176–191](F176Y) was synthesized on commercially available Novabiochem® NovaPEG Rink Amide resin (0.41 mmol/g, 50 mg, 21 µmol) using the standard AFPS protocol (**Section 2.2.1**, 20 mL/min flowrate) (**SI Figure 41**). Total synthesis time to afford resin-bound hGH[176–191](F176Y) was approximately 0.6 h. Cleavage of the peptidyl-resin (12 mg, approx. 2.8 µmol), according to Cleavage Protocol A (**Section 2.5.1**) afforded the crude peptide (2.1 mg, 44% purity by LCMS [**SI Figure 42**], 32% purity by UHPLC [**SI Figure 43**]).

UV-Vis synthesis trace

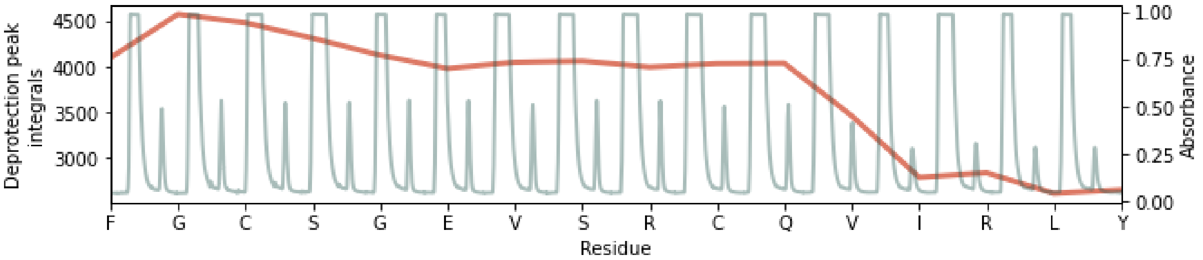

SI Figure 41. UV trace (λ = 310 nm) from AFPS of hGH[176–191](F176Y) (green) and deprotection peak integrals (red).

LC-MS of crude hGH[176–191](F176Y)

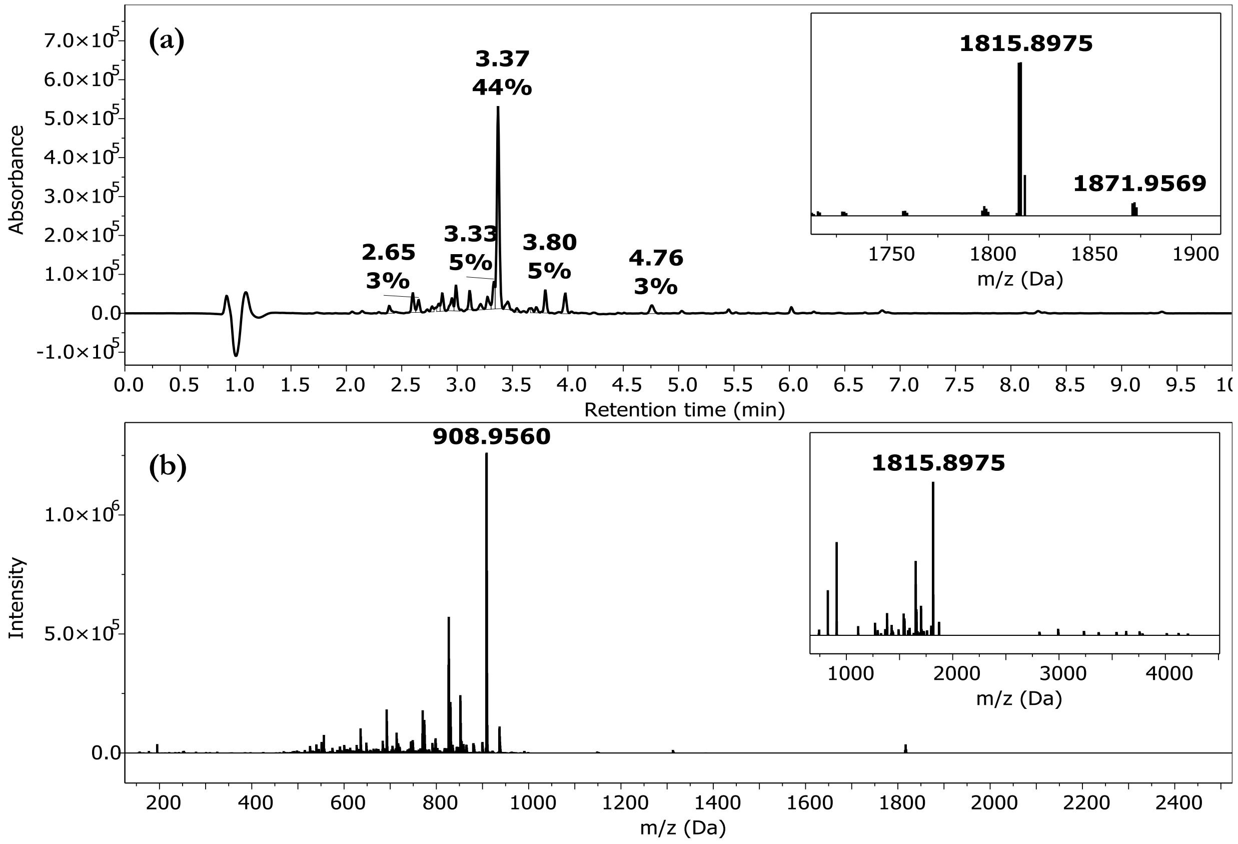

SI Figure 42. LCMS Profile of crude hGH[176–191](F176Y). (**a**) Absorbance chromatogram (λ = 214 nm); Rt 3.37 min, 44% purity. (**b**) ESI-TOF spectrum found within Rt 2–9 min (insert: deconvoluted masses); Monoisotopic mass (ESI+) calcd. for $C_{78}H_{126}N_{24}O_{22}S_{28}$ 1814.8920, found 1814.8954. LCMS Gradient A (**Section 2.7**).

UHPLC of crude hGH[176–191](F176Y)

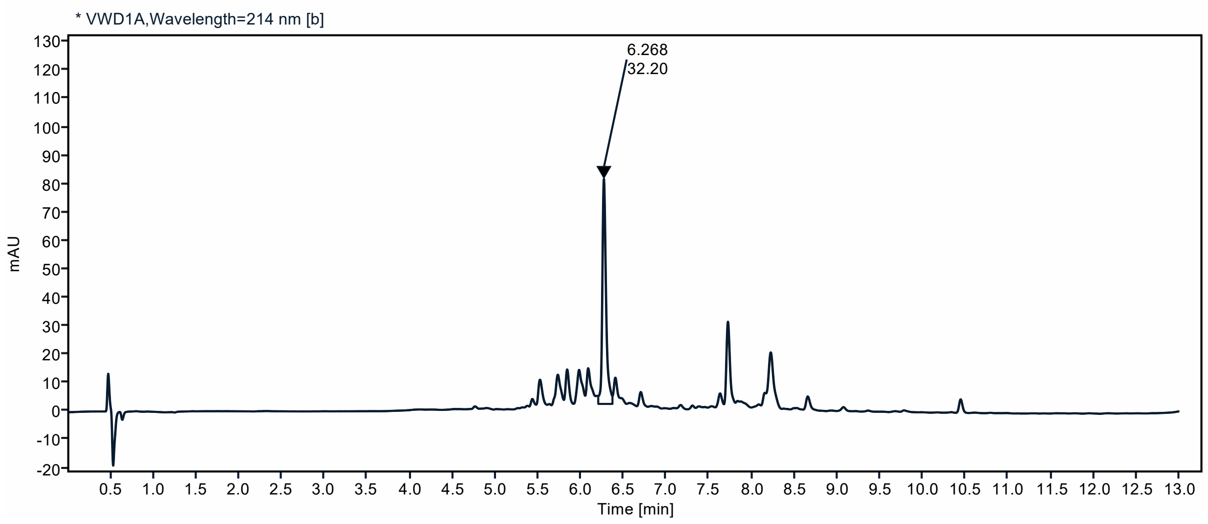

SI Figure 43. UHPLC profile of crude hGH[176–191](F176Y). Rt 6.27 min (Agilent Zorbax 300SB-C18 column, 5 µm, 2.1 × 150 mm, 5–95% MeCN over 10 min, ca. 9%B/min), 32% purity based on Area Under Curve (AUC) at λ = 214 nm

3.3.2 hGH[176–191](F176Y): [Arg(Pbf)]$_6$-Tag

```
                10          20  22
H2N-YLRIVQCRSV EGSCGFRRRR RR-CONH2   (22 AA)
```

The peptide hGH[176–191](F176Y) bearing the [Arg(Pbf)]$_6$-Tag was synthesized on commercially available Novabiochem® NovaPEG Rink Amide resin (0.41 mmol/g, 50 mg, 21 µmol) using the standard AFPS protocol (**Section 2.2.1**, 20 mL/min flowrate) (**SI Figure 44**). Total synthesis time to afford resin-bound hGH[176–191](F176Y)-[Arg(Pbf)]$_6$ was approximately 1 h. Cleavage of the peptidyl-resin (25 mg, approx. 3.7 µmol), according to Cleavage Protocol A (**Section 2.5.1**) afforded the crude peptide (3.1 mg, 80% purity by LCMS [**SI Figure 45**], 73% purity by UHPLC [**SI Figure 46**]).

UV-Vis synthesis trace

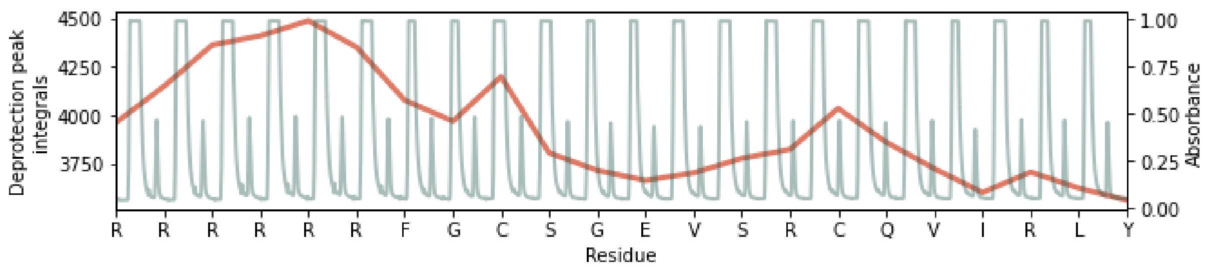

SI Figure 44. UV trace (λ = 310 nm) from AFPS of hGH[176–191](F176Y)-[Arg(Pbf)]$_6$ (green) and deprotection peak integrals (red).

LC-MS of crude hGH[176–191](F176Y)-(Arg)$_6$

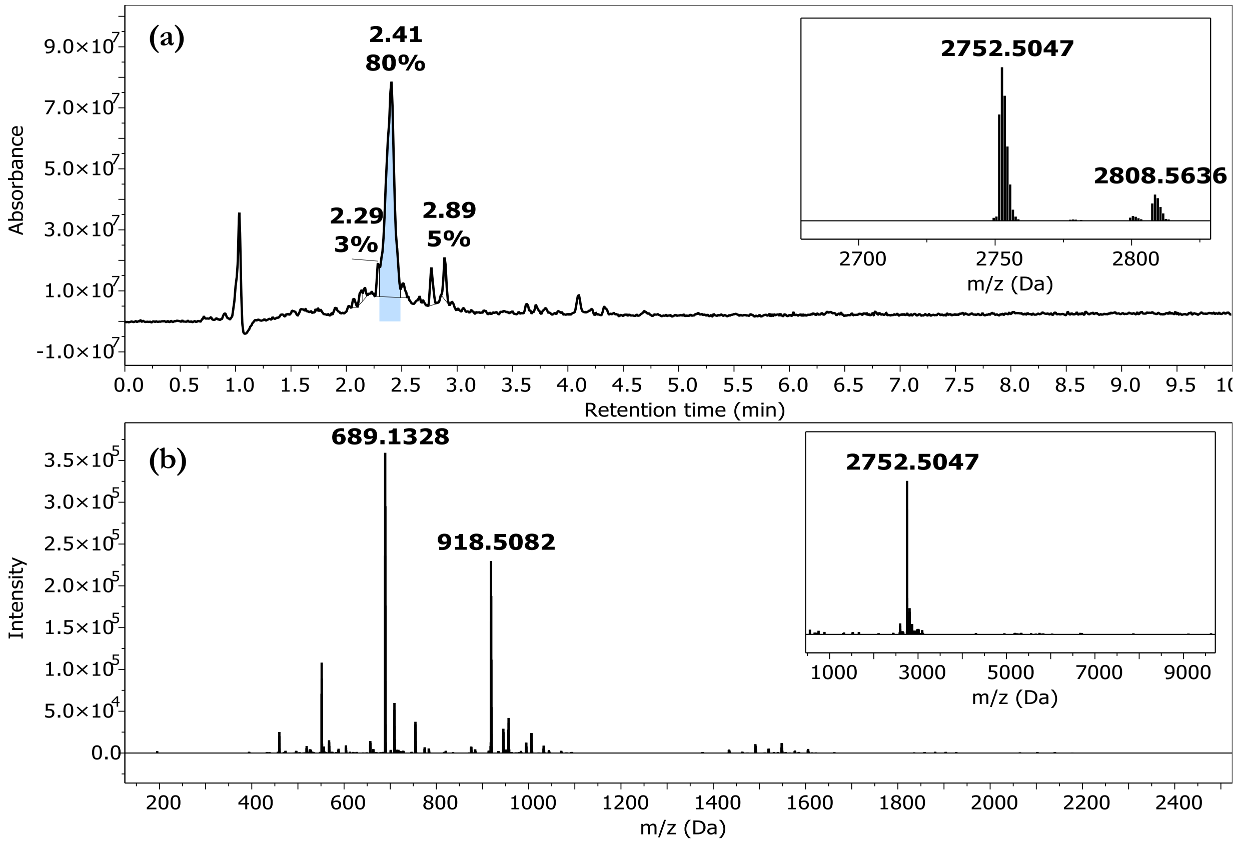

SI Figure 45. LCMS Profile of crude hGH[176–191](F176Y) bearing (Arg)$_6$. (**a**) Absorbance chromatogram (λ = 214 nm); Rt 2.41min, 80% purity. (**b**) ESI-TOF spectrum found within Rt 2–9 min (insert: deconvoluted masses); Monoisotopic mass (ESI+) calcd. for $C_{114}H_{198}N_{48}O_{28}S_2$ 2751.4987, found 2752.5047. LCMS Gradient A (**Section 2.7**).

UHPLC of crude hGH[176–191](F176Y)-(Arg)$_6$

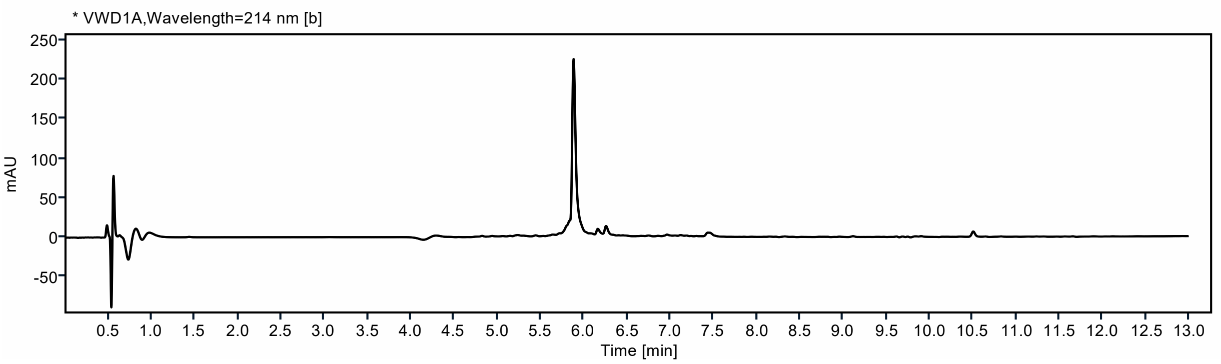

SI Figure 46. UHPLC profile of crude hGH[176–191](F176Y) bearing (Arg)$_6$. Rt 5.88 min (Agilent Zorbax 300SB-C18 column, 5 µm, 2.1 × 150 mm, 5–95% MeCN over 10 min, ca. 9%B/min), 73% purity based on Area Under Curve (AUC) at λ = 214 nm

3.3.3 hGH[176–191](F176Y): [Lys(Boc)]$_6$-Tag

```
               10          20  22
H2N-YLRIVQCRSV EGSCGFKKKK KK-CONH2    (22 AA)
```

The peptide hGH[176–191](F176Y) bearing the [Lys(Boc)]$_6$-Tag was synthesized on commercially available Novabiochem® NovaPEG Rink Amide resin (0.41 mmol/g, 51 mg, 21 µmol) using the standard AFPS protocol (**Section 2.2.1**, 20 mL/min flowrate) (**SI Figure 47**). Total synthesis time to afford resin-bound hGH[176–191](F176Y)-(Lys)$_6$ was approximately 1 h. Cleavage of the peptidyl-resin (16 mg, approx. 3.2 µmol), according to Cleavage Protocol A (**Section 2.5.1**) afforded the crude peptide (4.4 mg, 49% purity by LCMS [**SI Figure 48**], 33% purity by UHPLC [**SI Figure 49**]).

UV-Vis synthesis trace

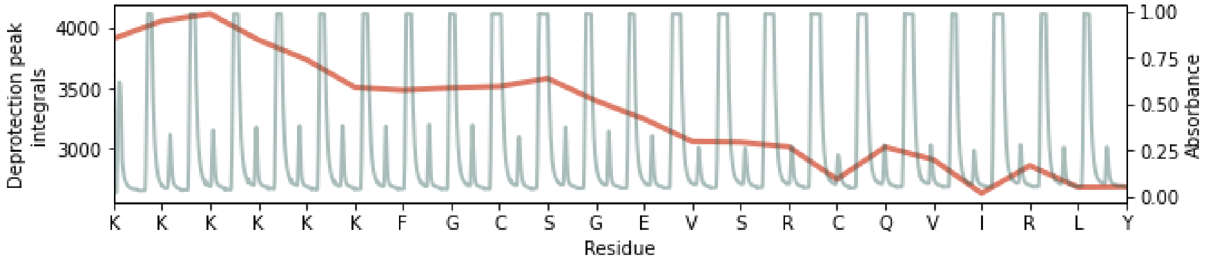

SI Figure 47. UV trace (λ = 310 nm) from AFPS of hGH[176–191](F176Y)-[Lys(Boc)]$_6$ (green) and deprotection peak integrals (red).

LC-MS of crude hGH[176–191](F176Y)-(Lys)$_6$

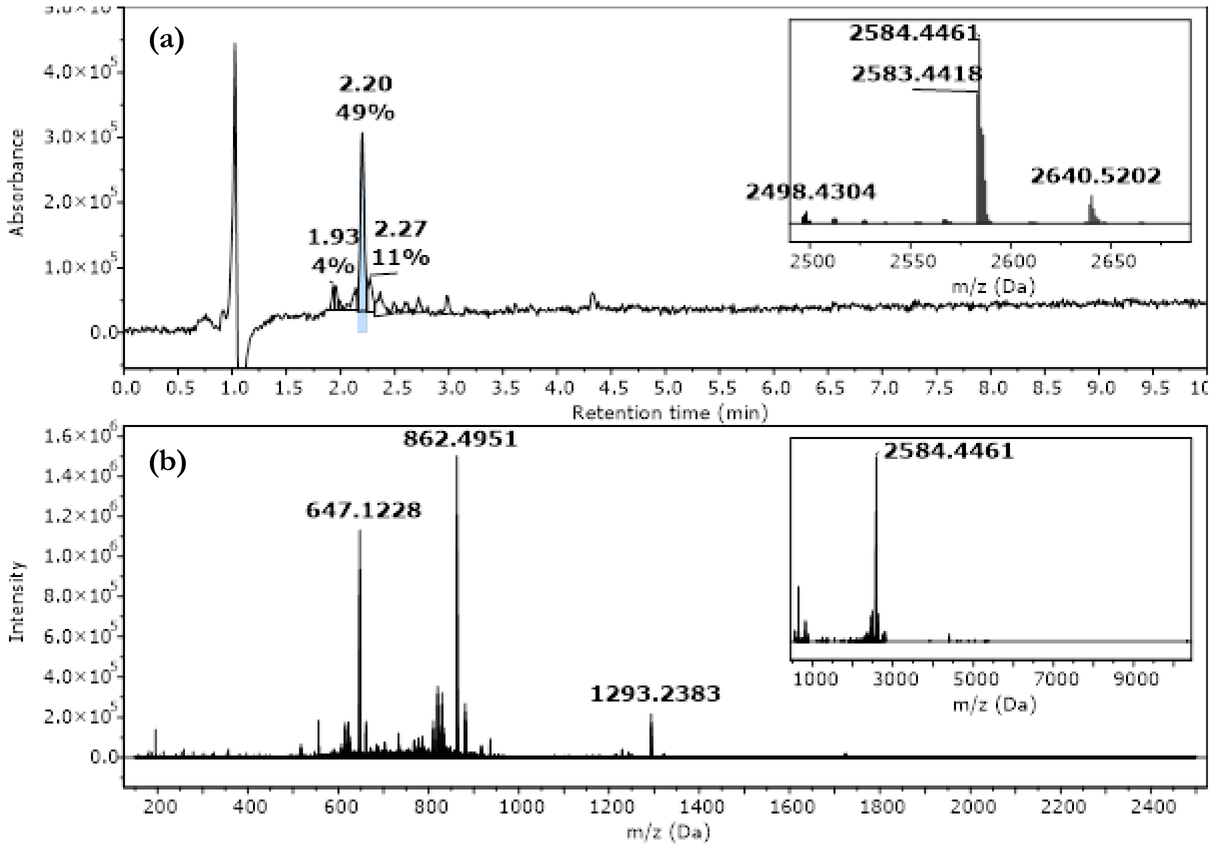

SI Figure 48. LCMS Profile of crude hGH[176–191](F176Y) bearing (Lys)$_6$. (**a**) Absorbance chromatogram (λ = 214 nm); Rt 2.20 min, 49% purity. (**b**) ESI-TOF spectrum found within Rt 1.5–9 min (insert: deconvoluted masses); Monoisotopic mass (ESI+) calcd. for $C_{114}H_{198}N_{36}O_{28}S_2$ 2583.4618, found 2583.4418. LCMS Gradient A (**Section 2.7**).

UHPLC of crude hGH[176–191](F176Y)-(Lys)$_6$

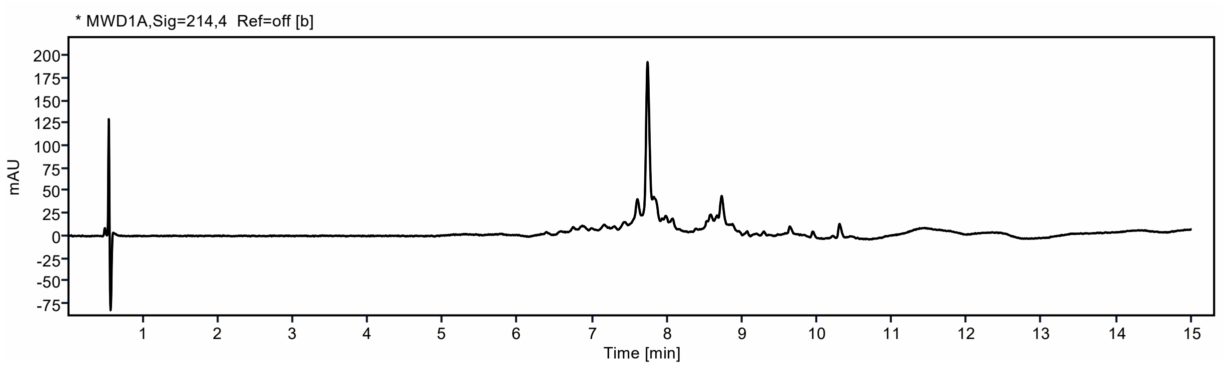

SI Figure 49. UHPLC profile of crude hGH[176–191](F176Y) bearing (Lys)$_6$. Rt 7.74 min (Agilent Zorbax 300SB-C18 column, 5 µm, 2.1 × 150 mm, 5–95% MeCN over 10 min, ca. 9%B/min), 33% purity based on Area Under Curve (AUC) at λ = 214 nm

3.3.4 hGH[176–191](F176Y): (Ala)$_6$-Tag

```
                     10              20   22
H2N-YLRIVQCRSV EGSCGFAAAA AA-CONH2    (22 AA)
```

The peptide hGH[176–191](F176Y) bearing the (Ala)$_6$-Tag was synthesized on commercially available Novabiochem® NovaPEG Rink Amide resin (0.41 mmol/g, 50 mg, 21 µmol) using the standard AFPS protocol (**Section 2.2.1**, 20 mL/min flowrate) (**SI Figure 50**). Total synthesis time

to afford resin-bound hGH[176–191](F176Y)-(Ala)$_6$ was approximately 1 h. Cleavage of the peptidyl-resin (19 mg, approx. 4.7 µmol), according to Cleavage Protocol A (**Section 2.5.1**) afforded the crude peptide (6.6 mg, 49% purity by LCMS [**SI Figure 51**], 23% purity by UHPLC [**SI Figure 52**]).

UV-Vis synthesis trace

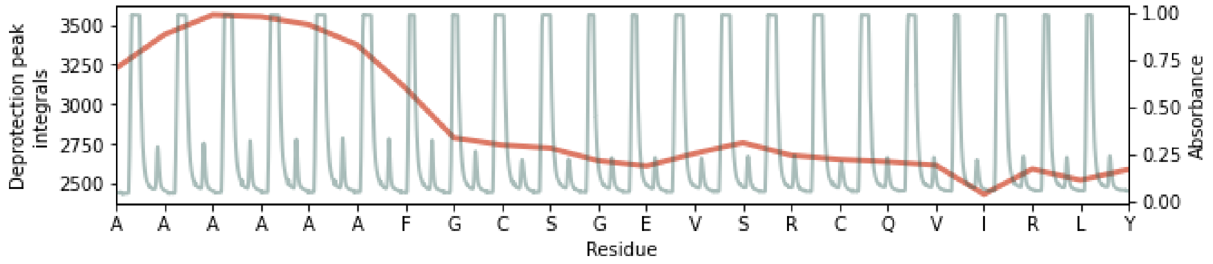

SI Figure 50. UV trace (λ = 310 nm) from AFPS of hGH[176–191](F176Y)-(Ala)$_6$ (green) and deprotection peak integrals (red).

LC-MS of crude hGH[176–191](F176Y)-(Ala)$_6$

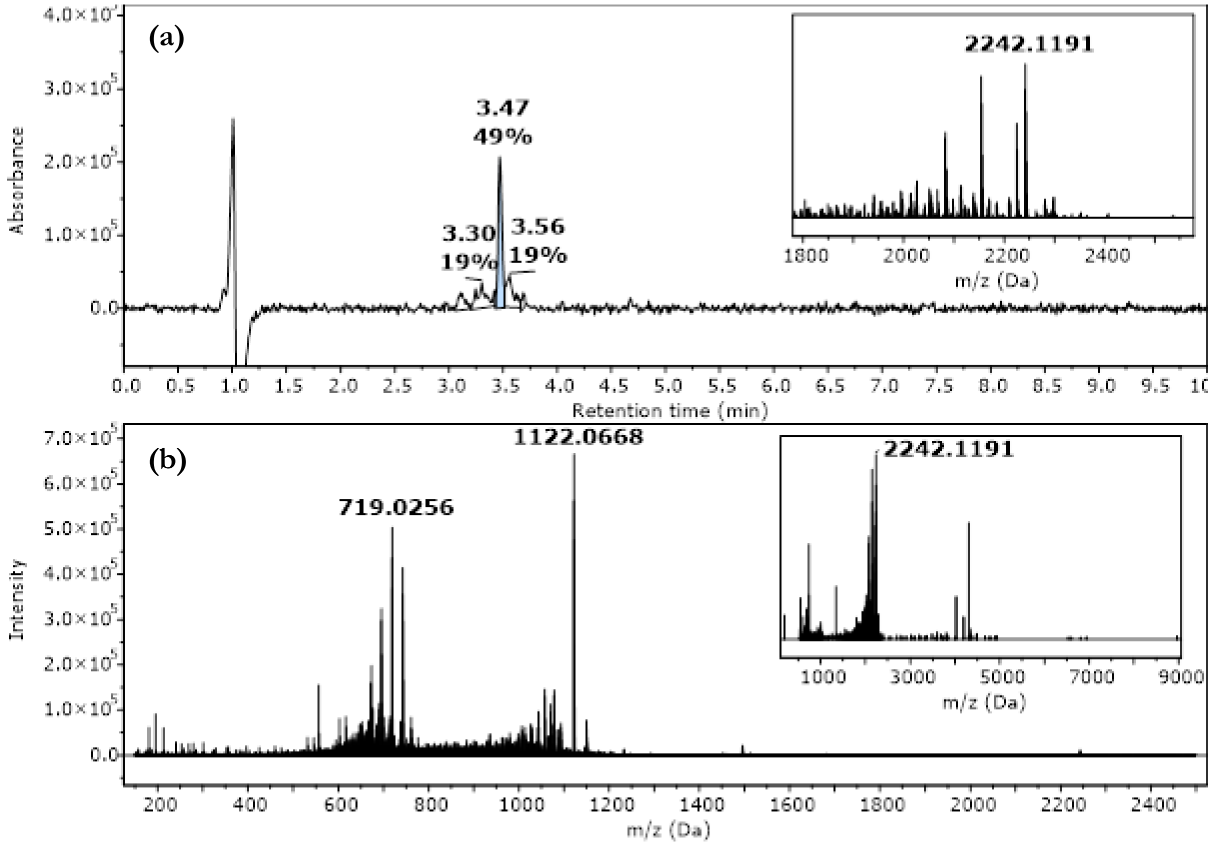

SI Figure 51. LCMS Profile of crude hGH[176–191](F176Y) bearing (Ala)$_6$. (**a**) Absorbance chromatogram (λ = 214 nm); Rt 3.47 min, 49% purity. (**b**) ESI-TOF spectrum found within Rt 2–9 min (insert: deconvoluted masses); Monoisotopic mass (ESI+) calcd. for $C_{96}H_{196}N_{30}O_{28}S_2$ 2241.1147, found 2241.1161. LCMS Gradient A (**Section 2.7**).

UHPLC of crude hGH[176–191](F176Y)-(Ala)$_6$

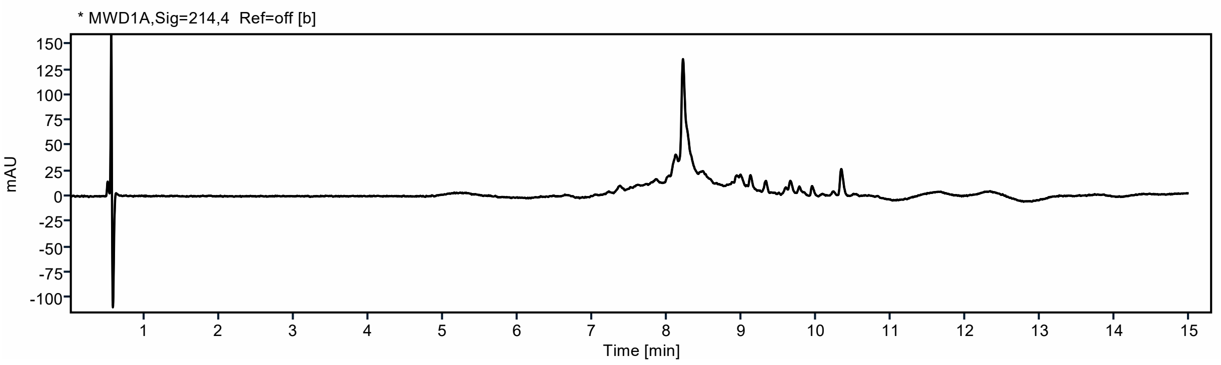

SI Figure 52. UHPLC profile of crude hGH[176–191](F176Y) bearing (Ala)$_6$. Rt 8.21 min (Agilent Zorbax 300SB-C18 column, 5 µm, 2.1 × 150 mm, 5–95% MeCN over 10 min, ca. 9%B/min), 23% purity based on Area Under Curve (AUC) at λ = 214 nm

3.3.5 hGH[176–191](F176Y): (Pro)$_6$-Tag

```
                 10            20  22
H2N-YLRIVQCRSV EGSCGFPPPP PP-CONH2    (22 AA)
```

The peptide hGH[176–191](F176Y) bearing the (Pro)$_6$-Tag was synthesized on commercially available Novabiochem® NovaPEG Rink Amide resin (0.41 mmol/g, 50 mg, 21 µmol) using the standard AFPS protocol (**Section 2.2.1**, 20 mL/min flowrate) (**SI Figure 53**). Total synthesis time to afford resin-bound hGH[176–191](F176Y)-(Pro)$_6$ was approximately 1 h. Cleavage of the peptidyl-resin (17 mg, approx. 3.7 µmol), according to Cleavage Protocol A (**Section 2.5.1**) afforded the crude peptide (6.6 mg, 33% purity by LCMS [**SI Figure 54**], 28% purity by UHPLC [**SI Figure 55**]).

UV-Vis synthesis trace

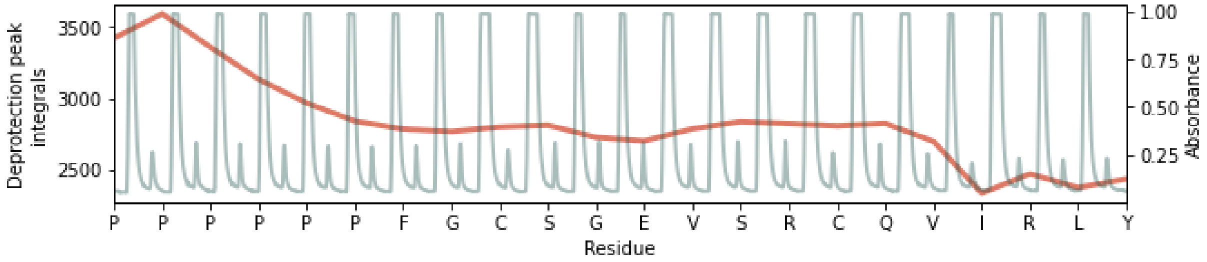

SI Figure 53. UV trace (λ = 310 nm) from AFPS of hGH[176–191](F176Y)-(Pro)$_6$ (green) and deprotection peak integrals (red).

LC-MS of crude hGH[176–191](F176Y)-(Pro)$_6$

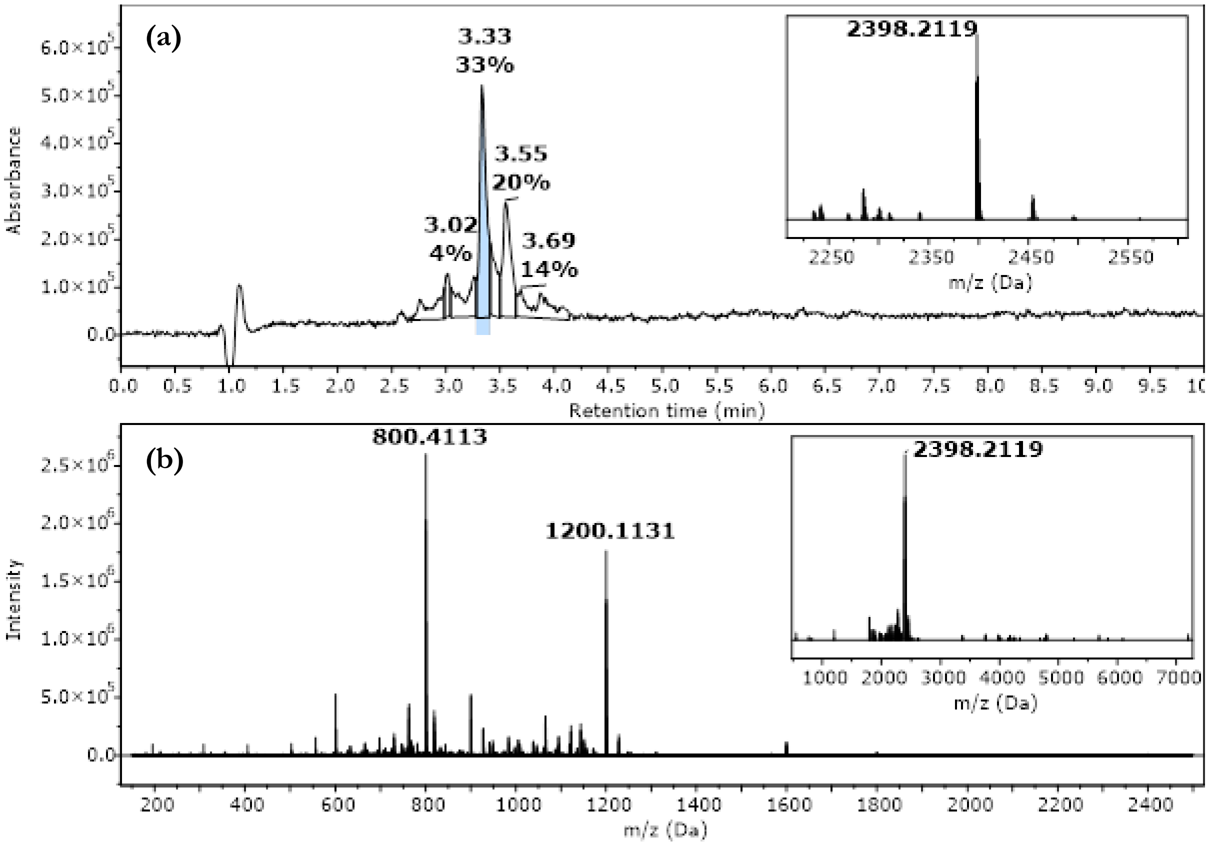

SI Figure 54. LCMS Profile of crude hGH[176–191](F176Y) bearing (Pro)$_6$. (**a**) Absorbance chromatogram (λ = 214 nm); Rt 3.33 min, 33% purity. (**b**) ESI-TOF spectrum found within Rt 2–9 min (insert: deconvoluted masses); Monoisotopic mass (ESI+) calcd. for $C_{108}H_{168}N_{30}O_{28}S_2$ 2397.2086, found 2397.2088. LCMS Gradient A (**Section 2.7**).

UHPLC of crude hGH[176–191](F176Y)-(Pro)$_6$

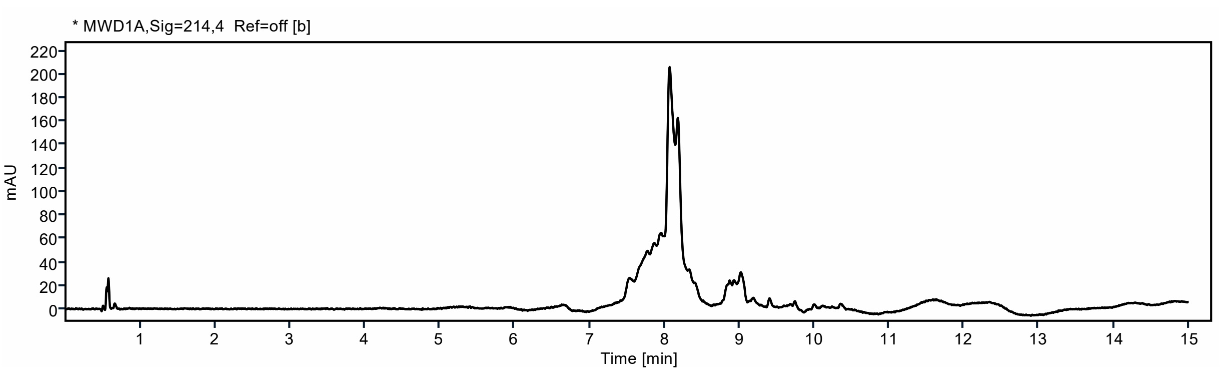

SI Figure 55. UHPLC profile of crude hGH[176–191](F176Y) bearing (Pro)$_6$. Rt 8.07 min (Agilent Zorbax 300SB-C18 column, 5 µm, 2.1 × 150 mm, 5–95% MeCN over 10 min, ca. 9%B/min), 28% purity based on Area Under Curve (AUC) at λ = 214 nm

3.3.6 hGH[176–191](F176Y): [Gln(Trt)]$_6$-Tag

```
                  10                  20    22
H2N-YLRIVQCRSV EGSCGFQQQQ QQ-CONH2    (22 AA)
```

The peptide hGH[176–191](F176Y) bearing the [Gln(Trt)]$_6$-Tag was synthesized on commercially available Novabiochem® NovaPEG Rink Amide resin (0.41 mmol/g, 50 mg, 21 µmol) using the standard AFPS protocol (**Section 2.2.1**, 20 mL/min flowrate) (**SI Figure 56**). Total synthesis time

to afford resin-bound hGH[176–191](F176Y)-(Gln)$_6$ was approximately 1 h. Cleavage of the peptidyl-resin (26.1 mg, approx. 3.8 μmol), according to Cleavage Protocol A (**Section 2.5.1**) afforded the crude peptide (5.9 mg, 69% purity by LCMS [**SI Figure 57**], 77% purity by UHPLC [**SI Figure 58**]).

UV-Vis synthesis trace

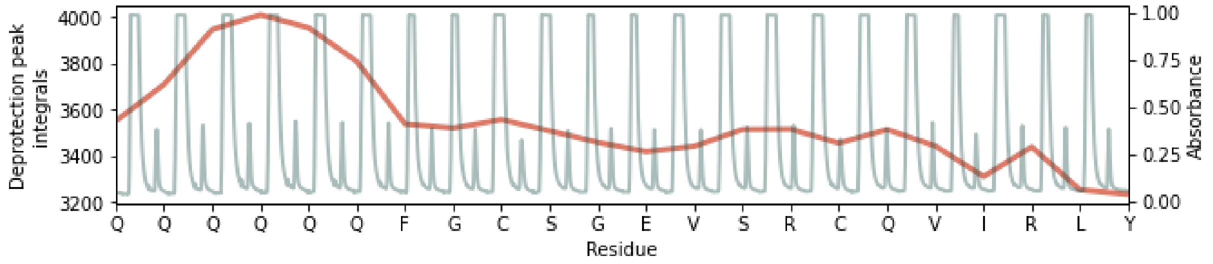

SI Figure 56. UV trace (λ = 310 nm) from AFPS of hGH[176–191](F176Y)- [Gln(Trt)]$_6$ (green) and deprotection peak integrals (red).

LC-MS of crude hGH[176–191](F176Y)-(Gln)$_6$

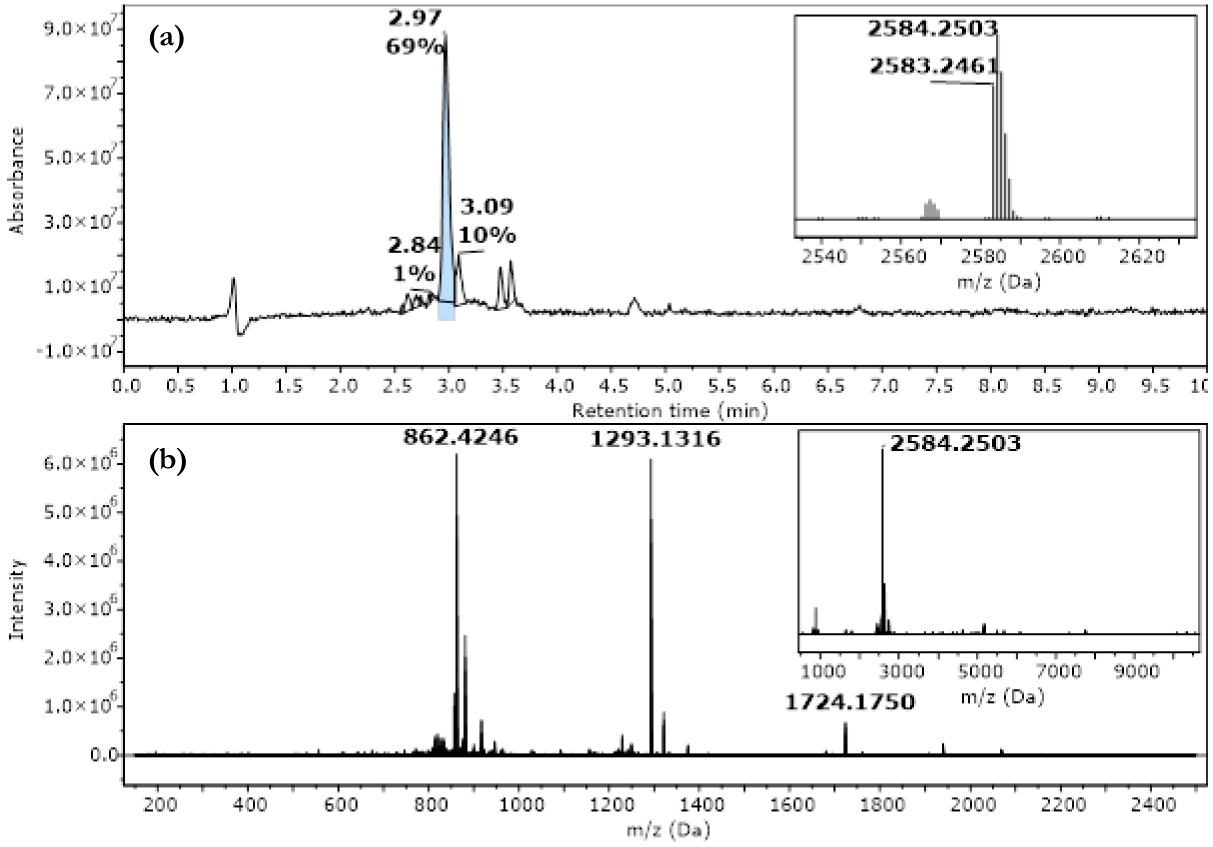

SI Figure 57. LCMS Profile of crude hGH[176–191](F176Y) bearing (Gln)$_6$. (**a**) Absorbance chromatogram (λ = 214 nm); Rt 2.97 min, 69% purity. (**b**) ESI-TOF spectrum found within Rt 2–9 min (insert: deconvoluted masses); Monoisotopic mass (ESI+) calcd. for $C_{108}H_{174}N_{36}O_{34}S_2$ 2583.2435, found 2583.2461. LCMS Gradient A (**Section 2.7**).

UHPLC of crude hGH[176–191](F176Y)-(Gln)$_6$

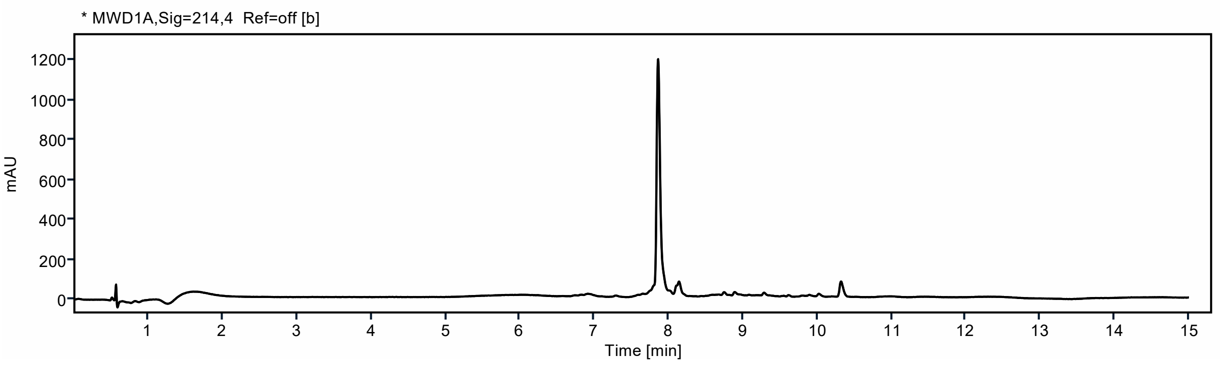

SI Figure 58. UHPLC profile of crude hGH[176–191] bearing (Gln)$_6$. Rt 7.86min (Agilent Zorbax 300SB-C18 column, 5 µm, 2.1 × 150 mm, 5–95% MeCN over 10 min, ca. 9%B/min), 77% purity based on Area Under Curve (AUC) at λ = 214 nm.

3.3.7 Cumulative synthesis traces of hGH[176–191](F176Y)

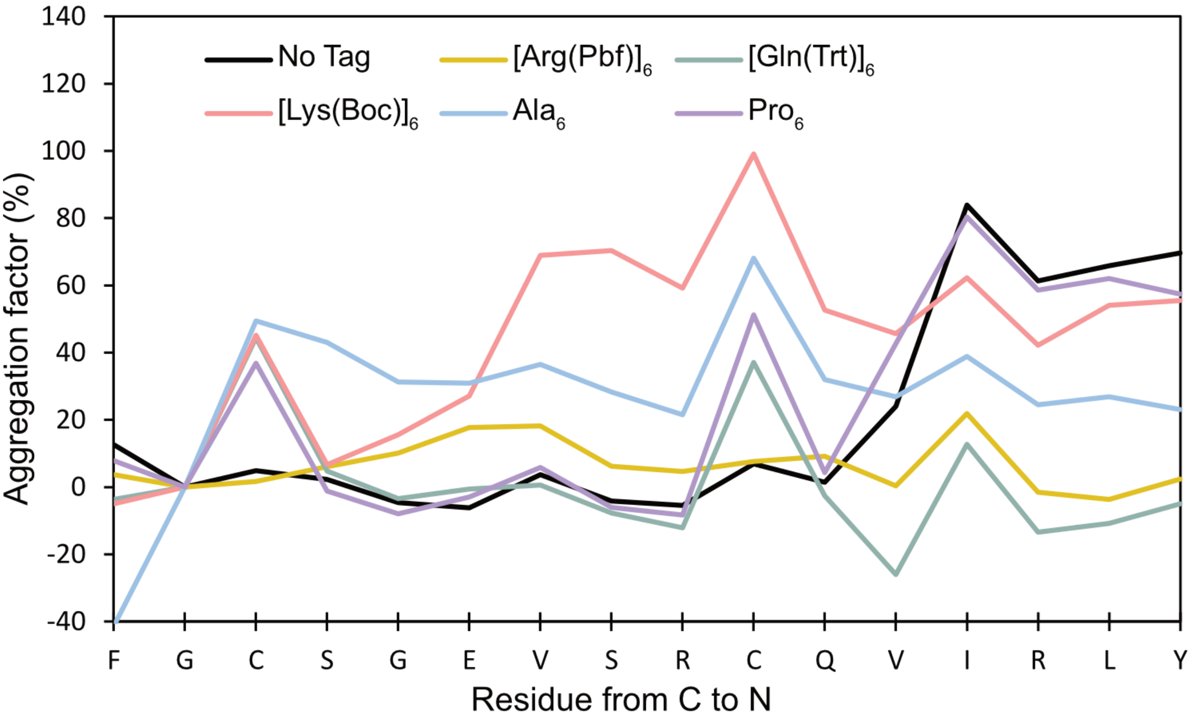

SI Figure 59. Aggregation as a function of Fmoc-deprotection peak broadening by in-line UV-Vis (310 nm) in flow-SPPS for hGH[176–191](F176Y) with various amino acid tag, normalized at Gly[15].

3.4 Evaluation of [Arg(Pbf)]$_6$-Tag: JR-10

3.4.1 JR-10: No tag (reference)

```
            10
H2N- WFFTLISTIM-CONH2      (10 AA)
```

The peptide JR-10 was synthesized on commercially available Novabiochem® NovaPEG Rink Amide resin (0.20 mmol/g, 0.15 g, 30 µmol) using the standard AFPS protocol (**Section 2.2.1**, 40 mL/min flowrate) (**SI Figure 60**). Total synthesis time to afford resin-bound JR-10 was approximately 0.5 h. Cleavage of the peptidyl-resin (37 mg, approx. 6.6 µmol), according to Cleavage

Protocol A (**Section 2.5.1**) afforded the crude peptide (1.1 mg, 21% purity by LCMS [**SI Figure 61**], 28% purity by UHPLC [**SI Figure 62**]).

UV-Vis synthesis trace

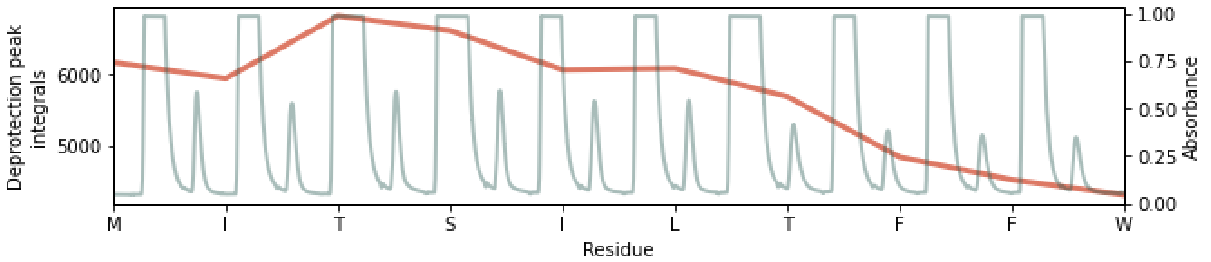

SI Figure 60. UV trace (λ = 310 nm) from AFPS of JR-10 (green) and deprotection peak integrals (red).

LC-MS of crude JR-10

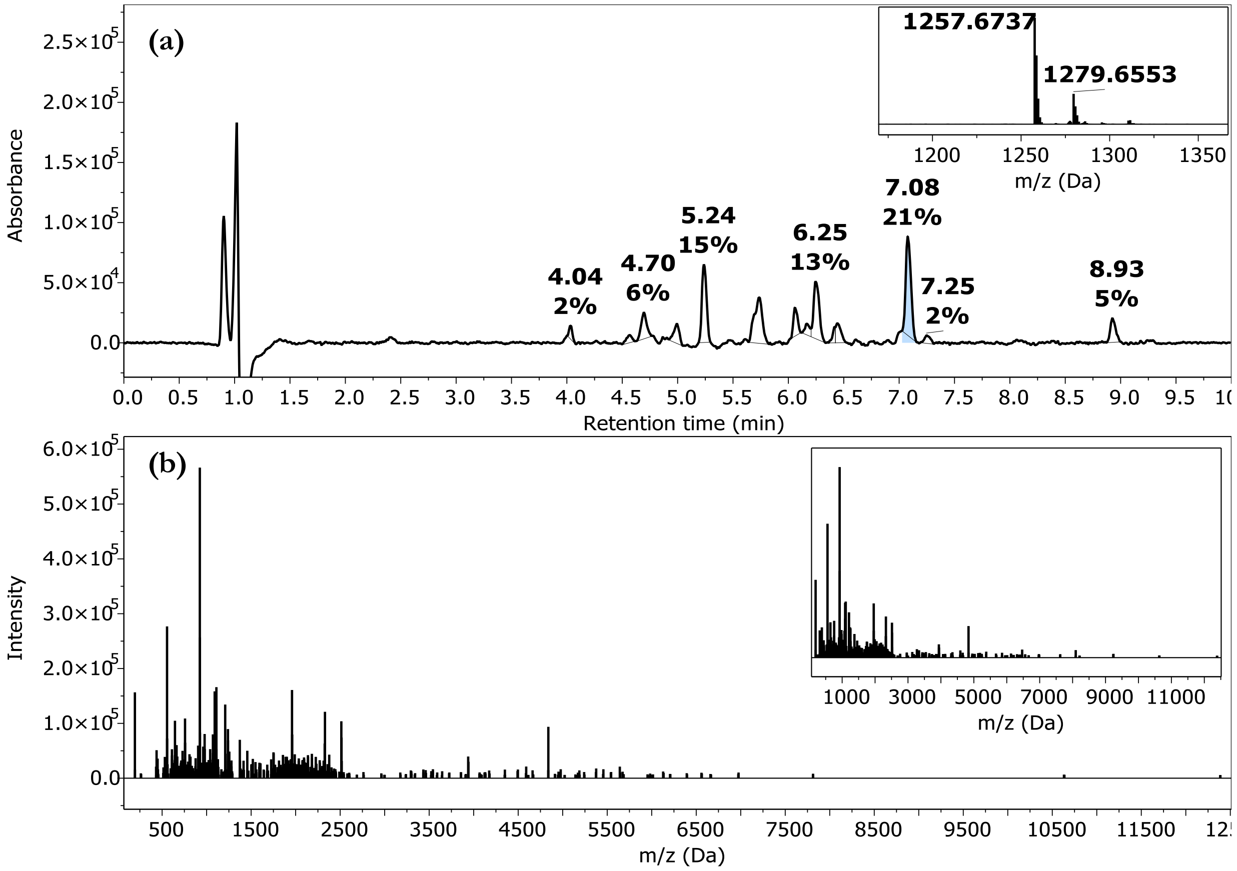

SI Figure 61. LCMS Profile of JR-10. (**a**) Absorbance chromatogram (λ = 214 nm) of JR-10; Rt 7.08 min, 21% purity. (**b**) ESI-TOF spectrum found within Rt 2–9 min (insert: deconvoluted masses); Monoisotopic mass (ESI+) calcd. for $C_{63}H_{91}N_{11}O_{14}S$ 1256.6628, found 1257.6737. LCMS Gradient A (**Section 2.7**).

UHPLC of crude JR-10

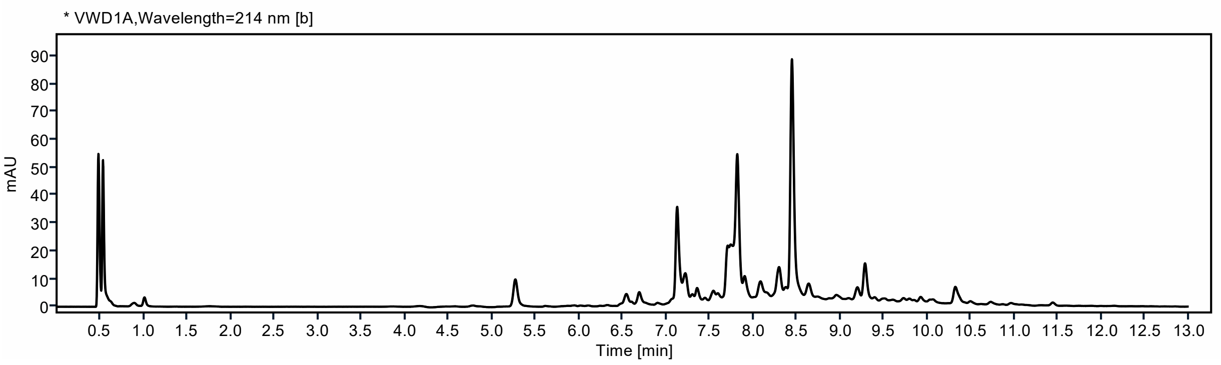

SI Figure 62. UHPLC profile of crude JR-10. Rt 8.44 min (Agilent Zorbax 300SB-C18 column, 5 µm, 2.1 × 150 mm, 5–95% MeCN over 10 min, ca. 9%B/min), 28% purity based on Area Under Curve (AUC) at λ = 214 nm.

3.4.2 JR-10: [Arg(Pbf)]$_6$- Tag

```
          10         16
H2N- WFFTLISTIM RRRRRR-CONH2        (16 AA)
```

The peptide JR-10 bearing the [Arg(Pbf)]$_6$ tag was synthesized on commercially available Novabiochem® NovaPEG Rink Amide resin (0.41 mmol/g, 51 mg, 21 µmol) using the standard AFPS protocol (**Section 2.2.1**, 40 mL/min flowrate) (**SI Figure 63**). Total synthesis time to afford resin-bound JR-10-[Arg(Pbf)]$_6$ was approximately 0.5 h. Cleavage of the peptidyl-resin (17 mg, approx. 3.5 µmol), according to Cleavage Protocol A (**Section 2.5.1**) afforded the crude peptide (2.6 mg, 61% purity by LCMS [**SI Figure 64**], 77% purity by UHPLC [**SI Figure 65**]).

UV-Vis synthesis trace

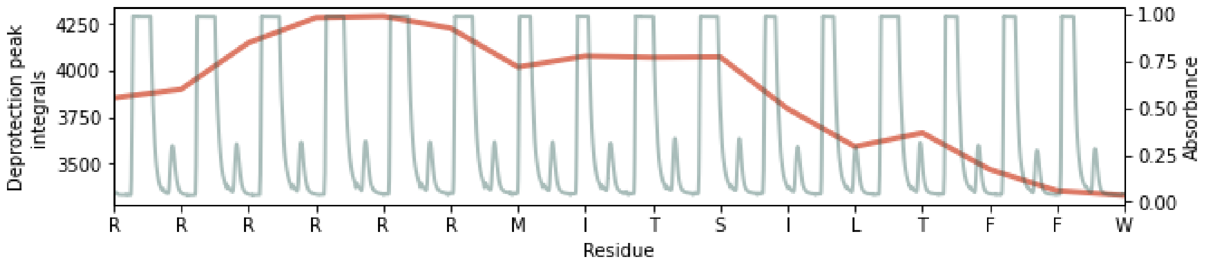

SI Figure 63. UV trace (λ = 310 nm) from AFPS of JR-10 bearing the [Arg(Pbf)]$_6$ tag (green) and deprotection peak integrals (red).

LC-MS of crude JR-10-(Arg)$_6$

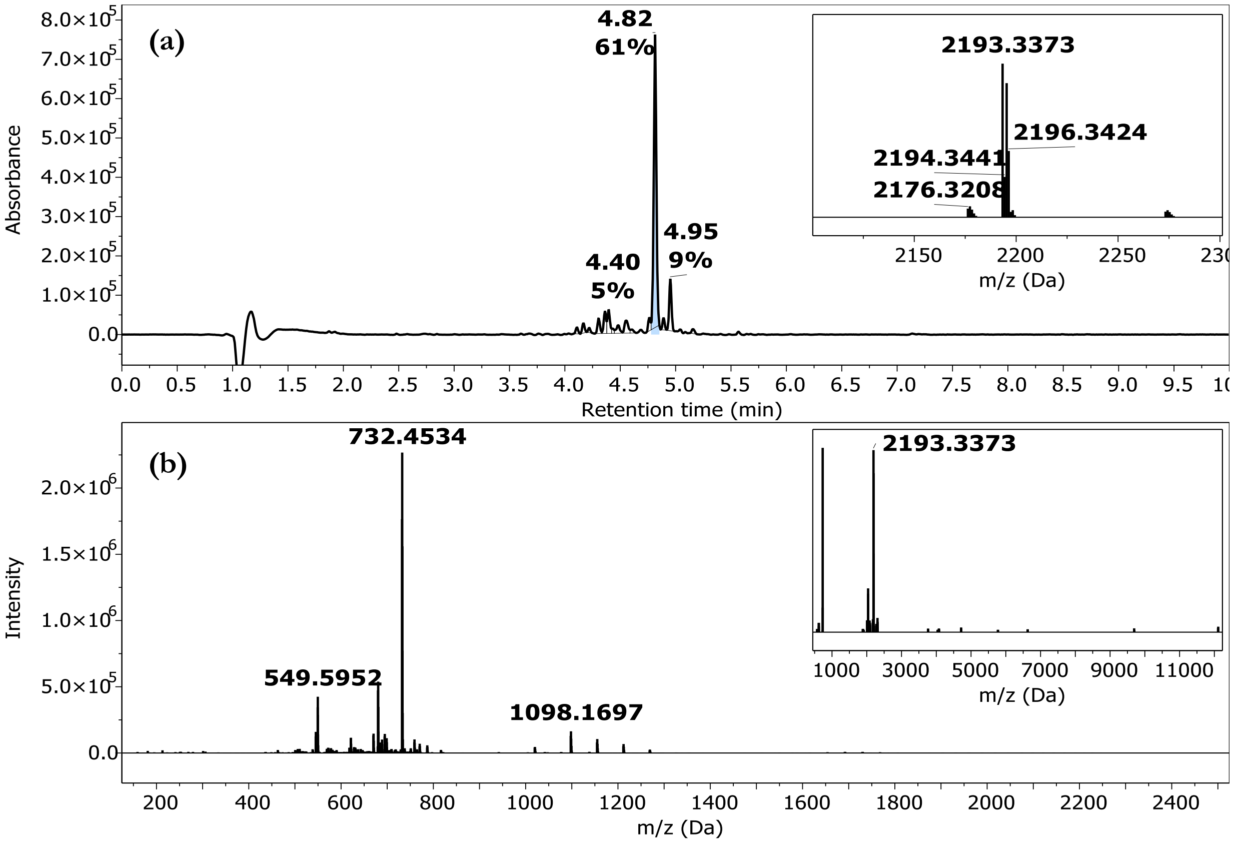

SI Figure 64. LCMS Profile of JR-10 bearing (Arg)$_6$. (**a**) Absorbance chromatogram (λ = 214 nm) of JR-10-(Arg)$_6$; Rt 4.82 min, 61% purity. (**b**) ESI-TOF spectrum found within Rt 2–9 min (insert: deconvoluted masses); Monoisotopic mass (ESI+) calcd. for $C_{99}H_{164}N_{36}O_{19}S$ 2193.2694, found 2193.3373. LCMS Gradient A (**Section 2.7**).

UHPLC of crude JR-10-(Arg)$_6$

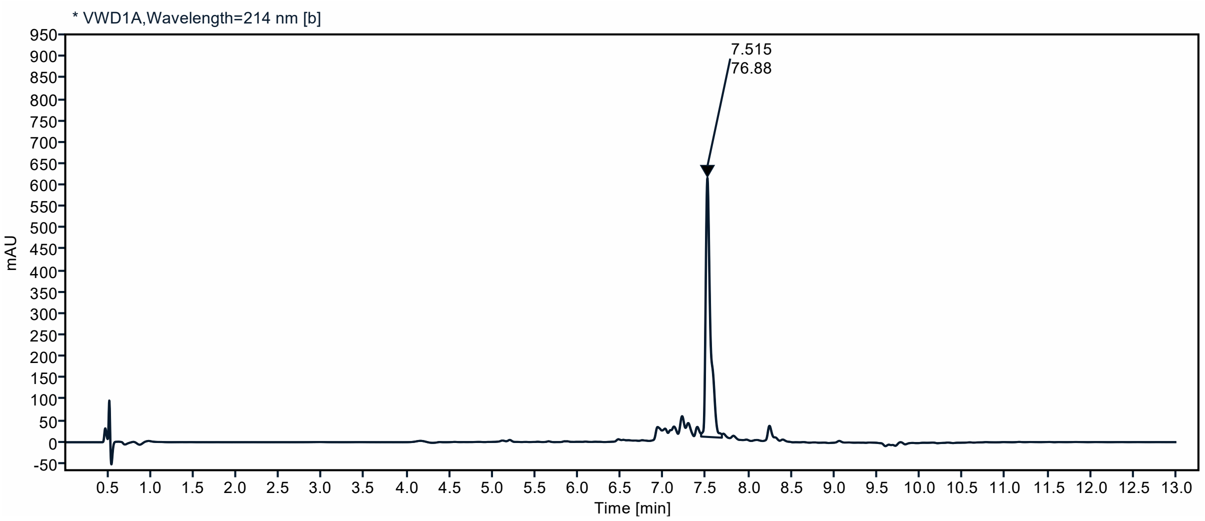

SI Figure 65. UHPLC profile of crude JR-10 bearing (Arg)$_6$ tag. Rt 7.52 min (Agilent Zorbax 300SB-C18 column, 5 µm, 2.1 × 150 mm, 5–95% MeCN over 10 min, ca. 9%B/min), 77% purity based on Area Under Curve (AUC) at λ = 214 nm.

3.4.3 Cumulative synthesis traces of JR-10

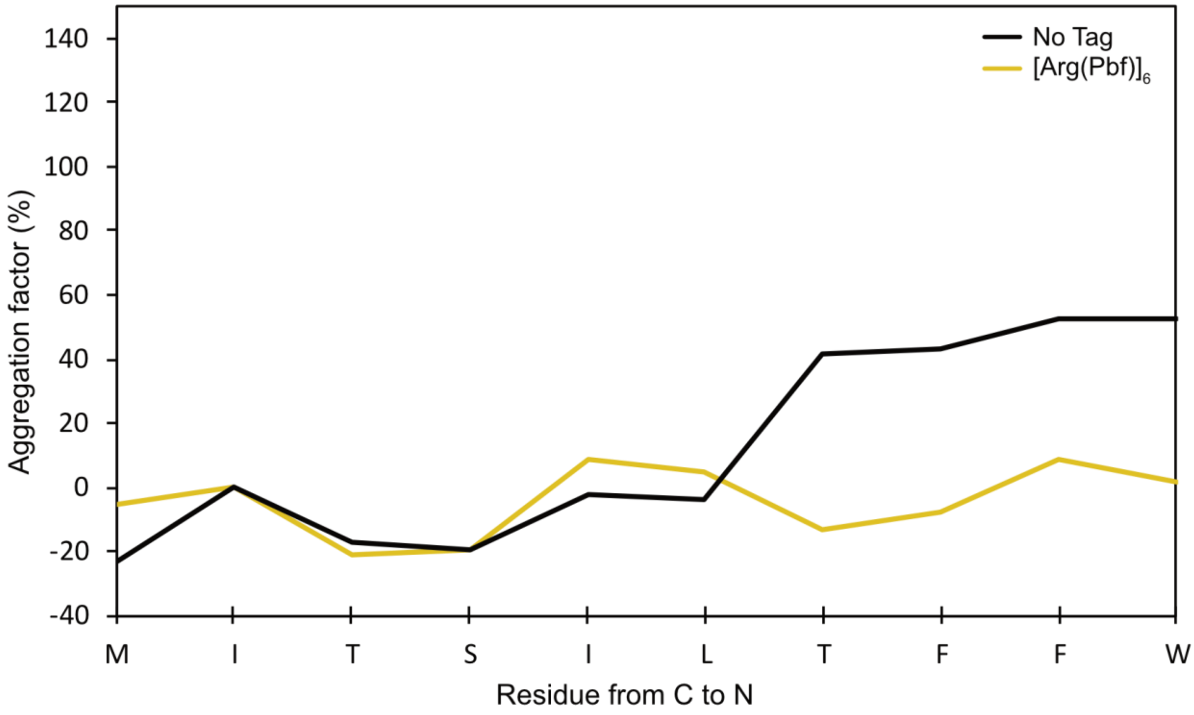

SI Figure 66. Aggregation as a function of Fmoc-deprotection peak broadening by in-line UV-Vis (310 nm) in flow-SPPS for JR-10 with and without [Arg(Pbf)]$_6$ tag, normalized at Ile[9].

3.5 Evaluation of [Arg(Pbf)]$_6$-Tag: MYC[123–143]

3.5.1 MYC[123–143]: No tag (reference)

```
            10                   20
H2N-FIKNIIIQDC   MWSGFSAAAK -CONH2        (20 AA)
```

The peptide MYC[123–143] was synthesized on commercially available Novabiochem® NovaPEG Rink Amide resin (0.41 mmol/g, 50 mg, 20 µmol) using the standard AFPS protocol (**Section 2.2.1**, 40 mL/min flowrate) (**SI Figure 67**). Total synthesis time to afford resin-bound MYC[123–143] was approximately 1 h. Cleavage of the peptidyl-resin (17 mg, approx. 3.8 µmol), according to Cleavage Protocol A (**Section 2.5.1**) afforded the crude peptide (7.6 mg crude product, 16% purity by LCMS [**SI Figure 68**], 27% purity by UHPLC [**SI Figure 69**]).

UV-Vis synthesis trace

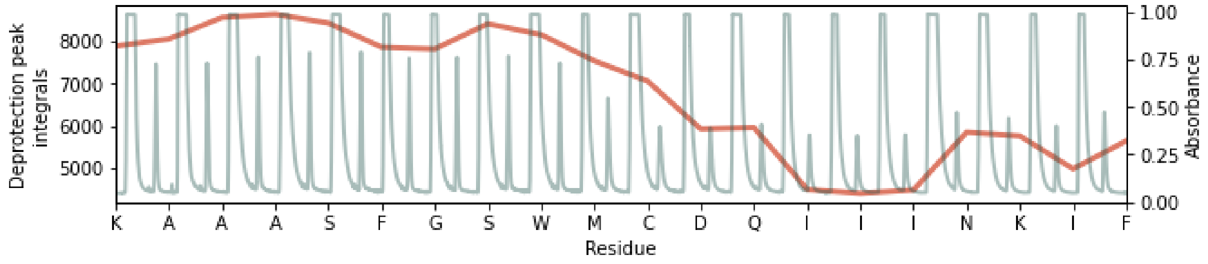

SI Figure 67. UV trace (λ = 310 nm) from AFPS of MYC[123–143] (green) and deprotection peak integrals (red).

LC-MS of crude MYC[123–143]

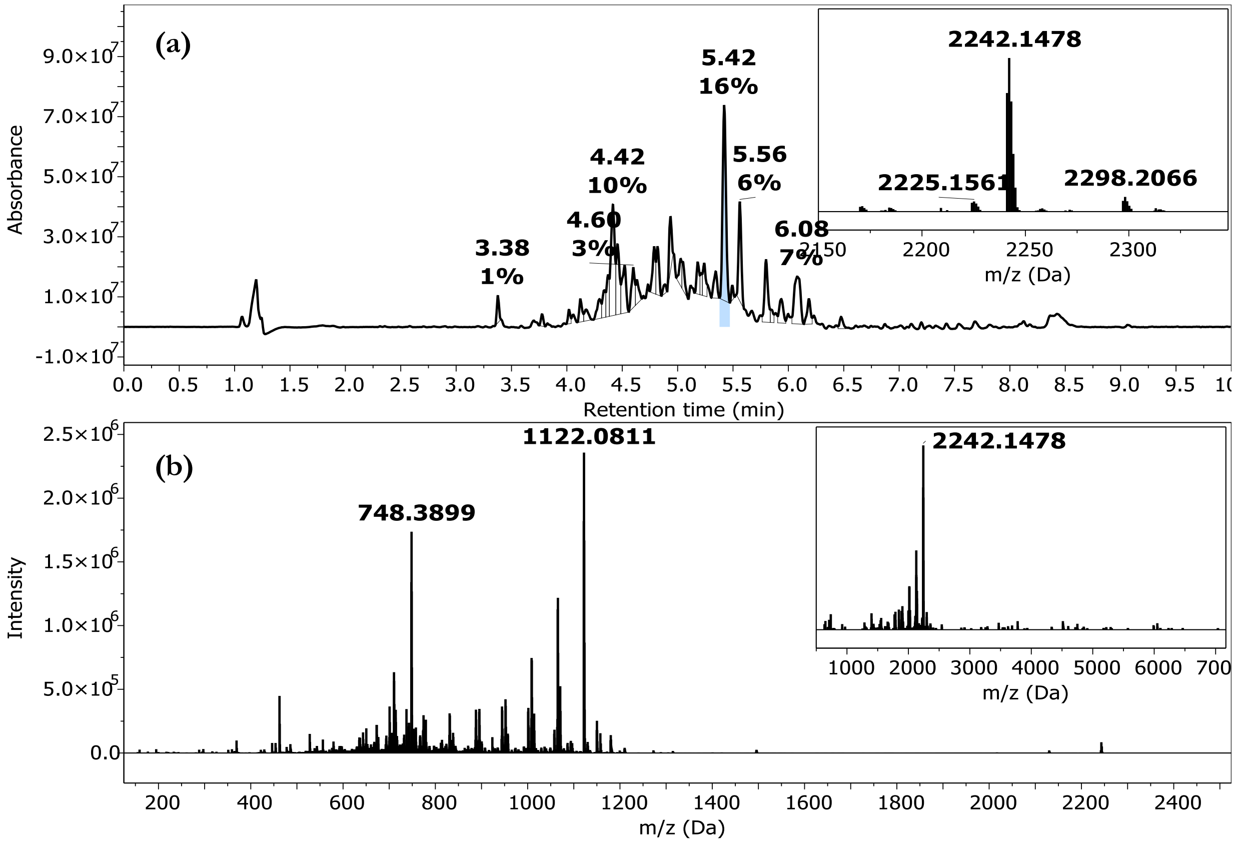

SI Figure 68. LCMS Profile of crude MYC[123–143]. (a) Absorbance chromatogram (λ = 214 nm) of MYC[123–143]; Rt 5.42 min, 16% purity. (**b**) ESI-TOF spectrum found within Rt 2–9 min (insert: deconvoluted masses); Monoisotopic mass (ESI+) calcd. for $C_{64}H_{117}N_{17}O_{15}S$ 2241.1439, found 2241.1452. LCMS Gradient A (**Section 2.7**).

UHPLC of crude MYC[123–143]

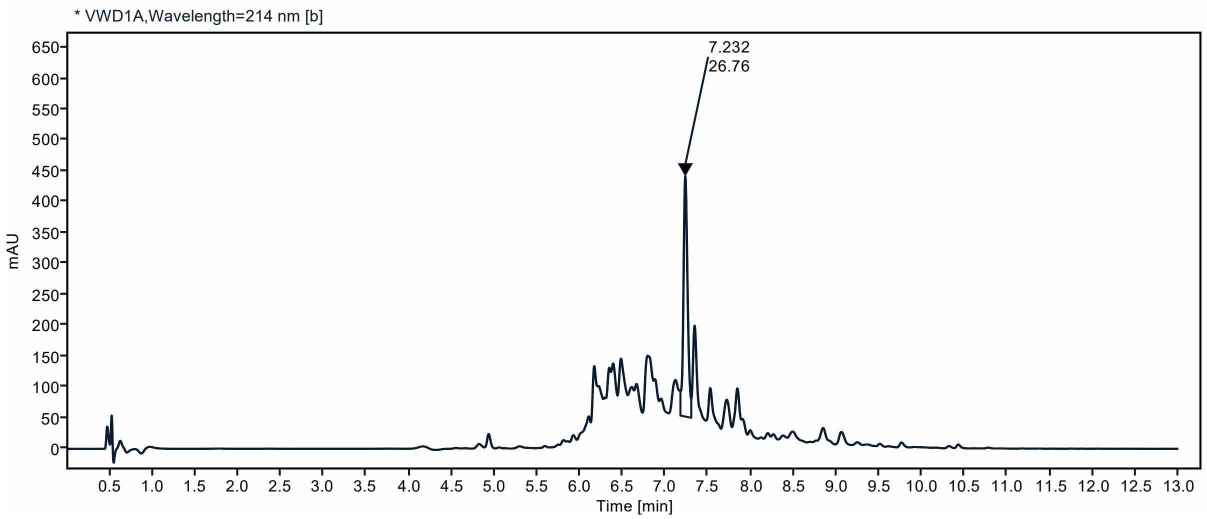

SI Figure 69. UHPLC profile of crude MYC[123–143]. Rt 7.23 min (Agilent Zorbax 300SB-C18 column, 5 µm, 2.1 × 150 mm, 5–95% MeCN over 10 min, ca. 9%B/min), 27% purity based on Area Under Curve (AUC) at λ = 214 nm.

3.5.2 MYC[123–143]: [Arg(Pbf)]$_6$-Tag

```
                10                     20          26
H2N-FIKNIIIQDC    MWSGFSAAAK RRRRRR-CONH2      (26 AA)
```

The peptide MYC[123–143] bearing the [Arg(Pbf)]$_6$ tag was prepared via Automated Fast-Flow Peptide Synthesis (AFPS) using Novabiochem® NovaPEG Rink Amide resin (0.41 mmol/g, 50.1 mg, 21 µmol) using the standard AFPS protocol (**Section 2.2.1**, 40 mL/min flowrate) (**SI Figure 70**). Total synthesis time to afford resin-bound MYC[123–143]-[Arg(Pbf)]$_6$ was approximately 1.2 h. Cleavage of the peptidyl-resin (18.5 mg, approx. 2.5 µmol), according to Cleavage Protocol A (**Section 2.5.1**) afforded the crude peptide (2.6 mg crude product, 74% purity by LCMS [**SI Figure 71**], 76% purity by UHPLC [**SI Figure 72**]).

UV-Vis synthesis trace

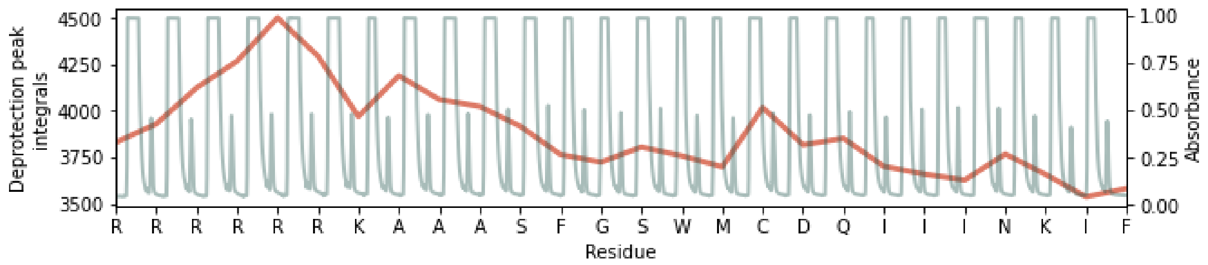

SI Figure 70. UV trace (λ = 310 nm) from AFPS of MYC[123–143]-[Arg(Pbf)]$_6$ (green) and deprotection peak integrals (red).

LC-MS of crude MYC[123–143]-(Arg)$_6$

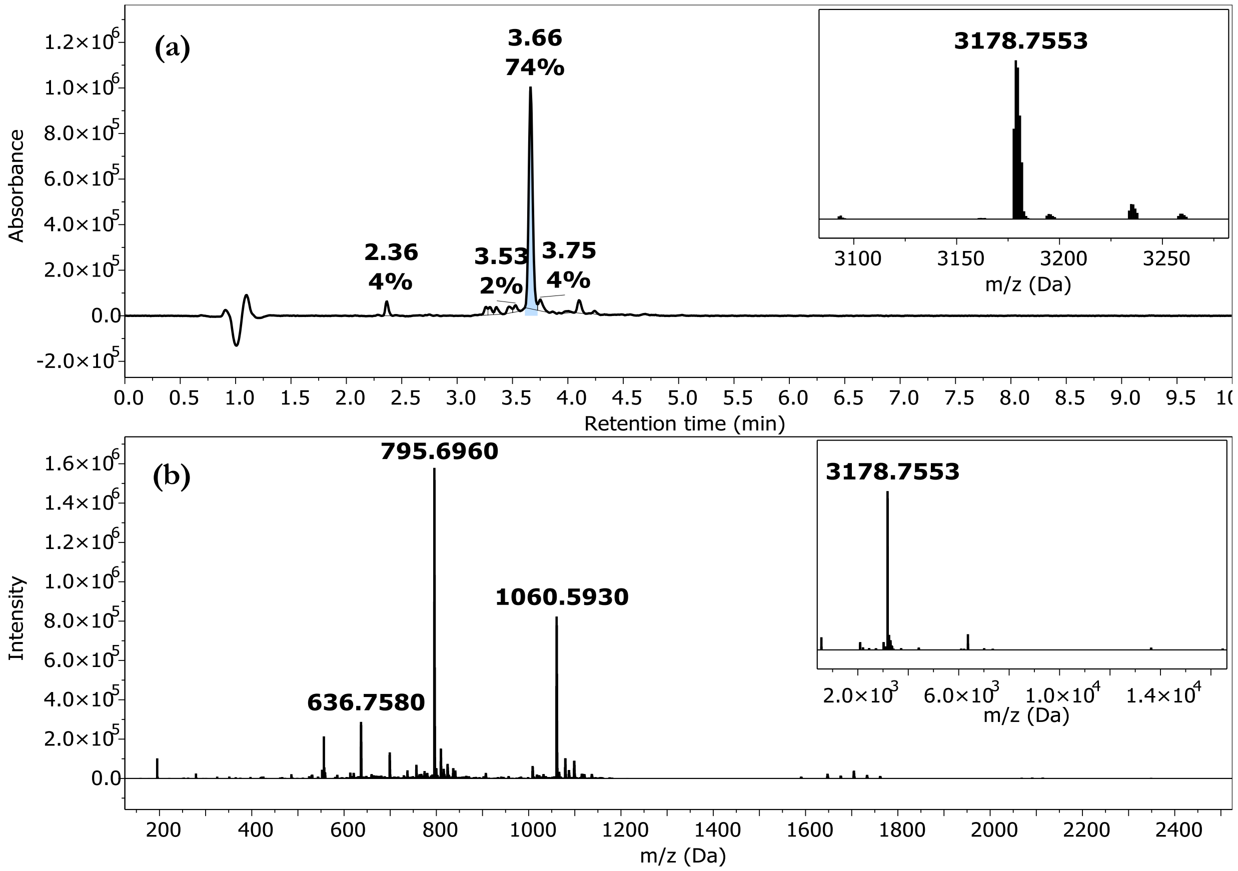

SI Figure 71. LCMS Profile of crude MYC[123–143] bearing (Arg)$_6$. (**a**) Absorbance chromatogram (λ = 214 nm) of MYC[123–143]-(Arg)$_6$; Rt 3.66 min, 74% purity. (**b**) ESI-TOF spectrum found within Rt 2–9 min (insert: deconvoluted masses); Monoisotopic mass (ESI+) calcd. for $C_{139}H_{232}N_{50}O_{32}S_2$ 3177.7505, found 3177.7537. LCMS Gradient A (**Section 2.7**).

UHPLC of crude MYC[123–143]-(Arg)$_6$

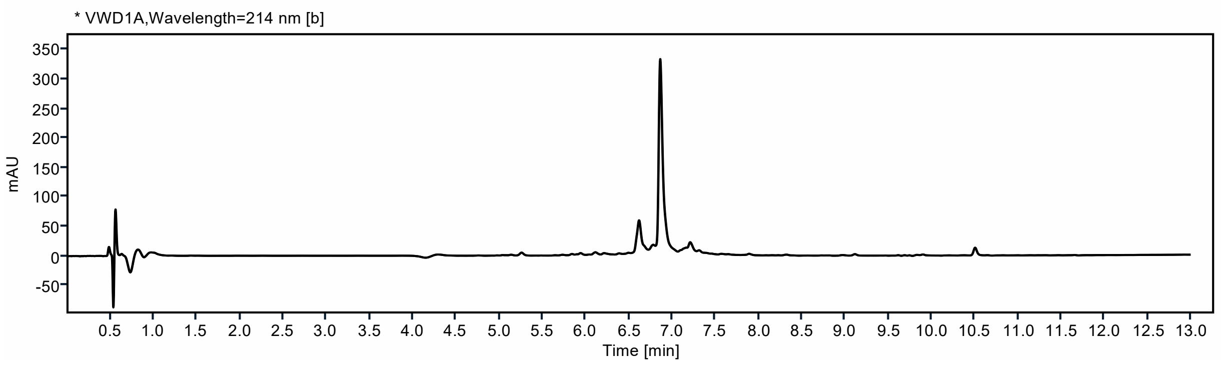

SI Figure 72. UHPLC profile of crude MYC[123–143] bearing (Arg)$_6$. Rt 6.86 min (Agilent Zorbax 300SB-C18 column, 5 µm, 2.1 × 150 mm, 5–95% MeCN over 10 min, ca. 9%B/min), 76% purity based on Area Under Curve (AUC) at λ = 214 nm

3.5.3 Cumulative synthesis traces of MYC[123–143]

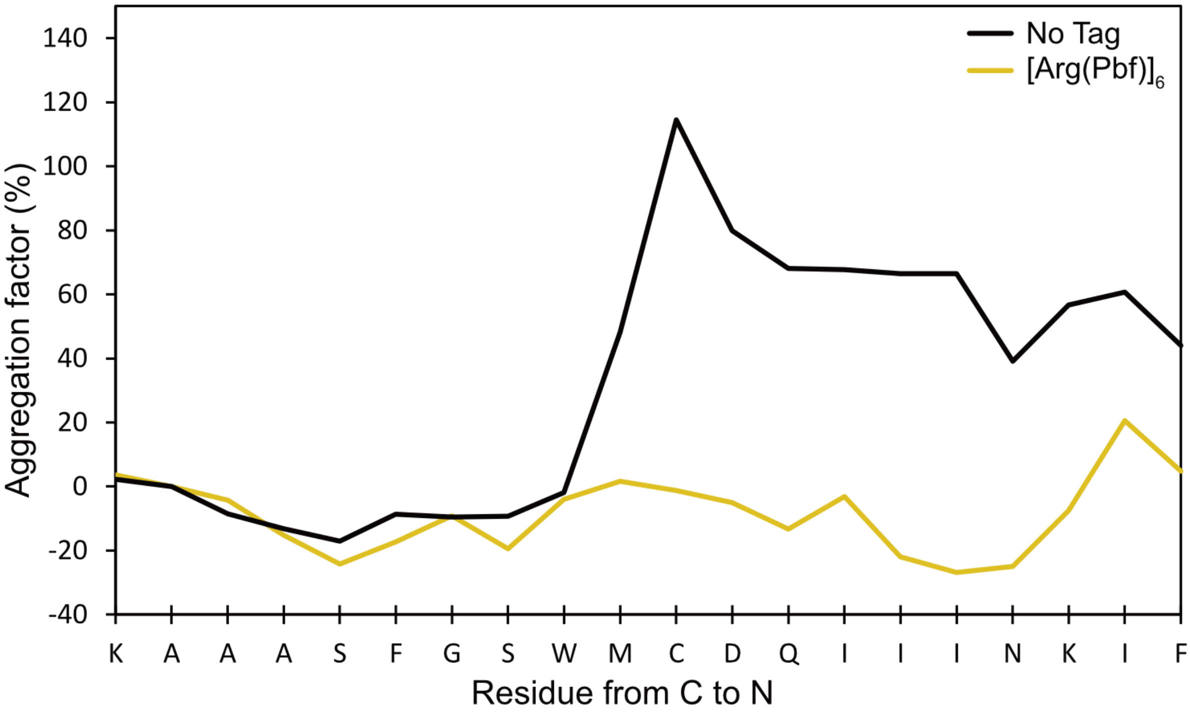

SI Figure 73. Aggregation as a function of Fmoc-deprotection peak broadening by in-line UV-Vis (310 nm) in flow-SPPS for MYC[123–143] with and without [Arg(Pbf)]$_6$ tag, and with [Arg(Pbf)]$_6$ tag on "Low loading" Rink amide resin, normalized at Ala[142].

3.6 Evaluation of [Arg(Pbf)]$_6$-Tag: MYC[85–143]

3.6.1 MYC[85–143]: No tag (reference)

```
                  10                   20                  30                  40
H2N-DNDGGGGSFS    TADQLEMVTE    LLGGDMVNQS    FICDPDDETF
                  50                   59
    IKNIIIQDCM    WSGFSAAAK -CONH2                                  (59 AA)
```

The peptide MYC[86–143] was synthesized on commercially available Novabiochem® NovaPEG Rink Amide resin (0.20 mmol/g, 0.15 mg, 30 µmol) using the standard AFPS protocol (**Section 2.2.1**, 20 mL/min flowrate) (**SI Figure 74**). Total synthesis time to afford resin-bound MYC[86–

143] was approximately 3 h. Cleavage of the peptidyl-resin (97 mg, approx. 8.5 µmol), according to Cleavage Protocol A (**Section 2.5.1**) afforded crude material (14 mg, desired mass not detected by LCMS [**SI Figure 75**], purity by UHPLC N/A [**SI Figure 76**]).

UV-Vis synthesis trace

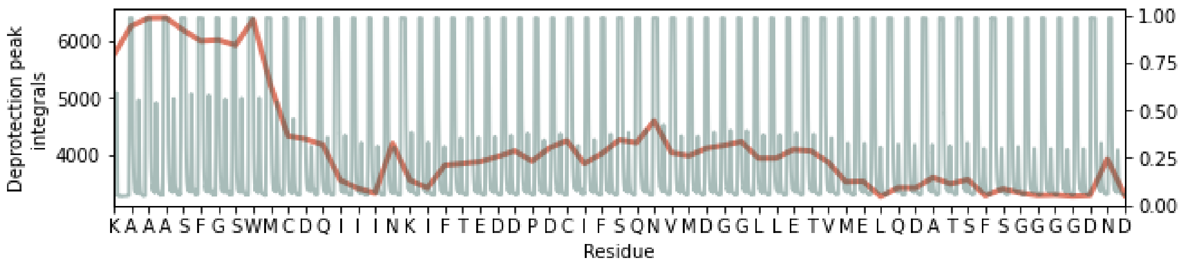

SI Figure 74. UV trace (λ = 310 nm) from AFPS of MYC[85–143] (green) and deprotection peak integrals (red).

LC-MS of crude MYC[85–143]

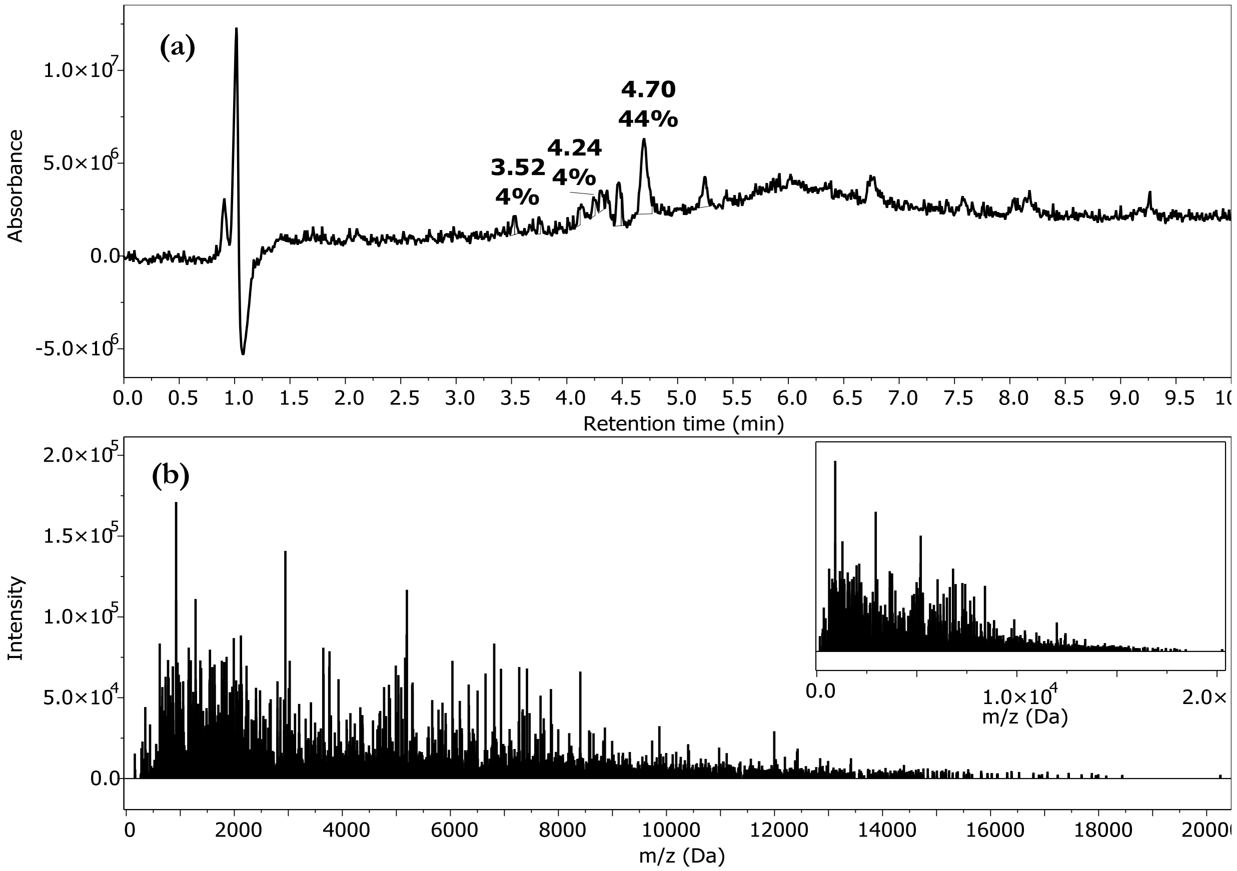

SI Figure 75. LCMS Profile of MYC[85–143]. (**a**) Absorbance chromatogram (λ = 214 nm) of MYC[85–143]; Rt N/A. (**b**) ESI-TOF spectrum found within Rt 3–9 min (insert: deconvoluted masses); Monoisotopic mass (ESI+) calcd. for $C_{270}H_{413}N_{69}O_{95}S_5$ 6301.8211, not found. LCMS Gradient A (**Section 2.7**).

UHPLC of crude MYC[85–143]

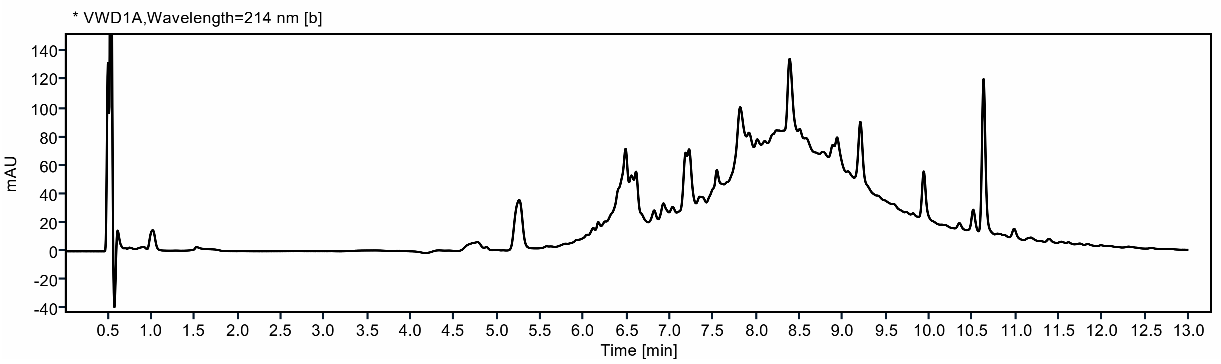

SI Figure 76. UHPLC profile of crude MYC[85–143]. Purity N/A (Agilent Zorbax 300SB-C18 column, 5 µm, 2.1 × 150 mm, 5–95% MeCN over 10 min, ca. 9%B/min).

3.6.2 MYC[86–143]: [Arg(Pbf)]$_6$-Tag

```
                 10                 20                 30                 40
H2N-NDGGGGSFST     ADQLEMVTEL     LGGDMVNQSF     ICDPDDETFI

                 50                 60
     KNIIIQDCMW     SGFSAAAKRR RRRR-CONH2                              (64 AA)
```

The peptide MYC[86–143] bearing the [Arg(Pbf)]$_6$ tag was synthesized on commercially available Novabiochem® NovaPEG Rink Amide resin (0.20 mmol/g, 0.15 mg, 30 µmol) using the standard AFPS protocol (**Section 2.2.1**, 20 mL/min flowrate) (**SI Figure 77**). Total synthesis time to afford resin-bound MYC[86–143] was approximately 3 h. Cleavage of the peptidyl-resin (39 mg, approx. 8.6 µmol), according to Cleavage Protocol A (**Section 2.5.1**) afforded the crude peptide (14 mg crude product, 39% purity by LCMS [**SI Figure 78**], 27% purity by UHPLC [**SI Figure 79**]).

UV-Vis synthesis trace

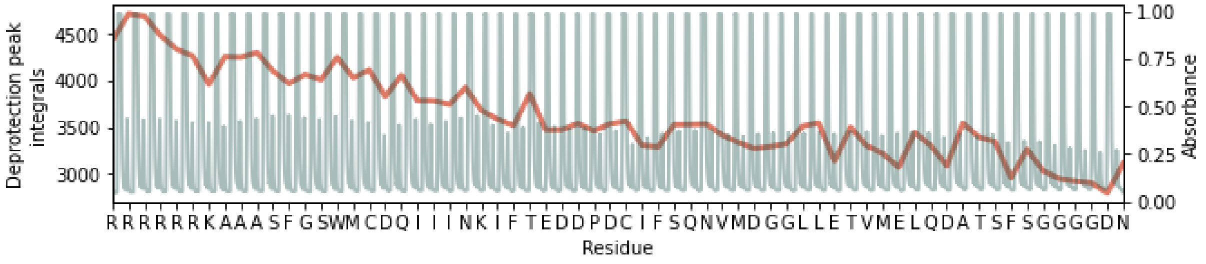

SI Figure 77. UV trace (λ = 310 nm) from AFPS of MYC[86–143]-[Arg(Pbf)]$_6$ (green) and deprotection peak integrals (red).

LC-MS of crude MYC[86–143]-(Arg)$_6$

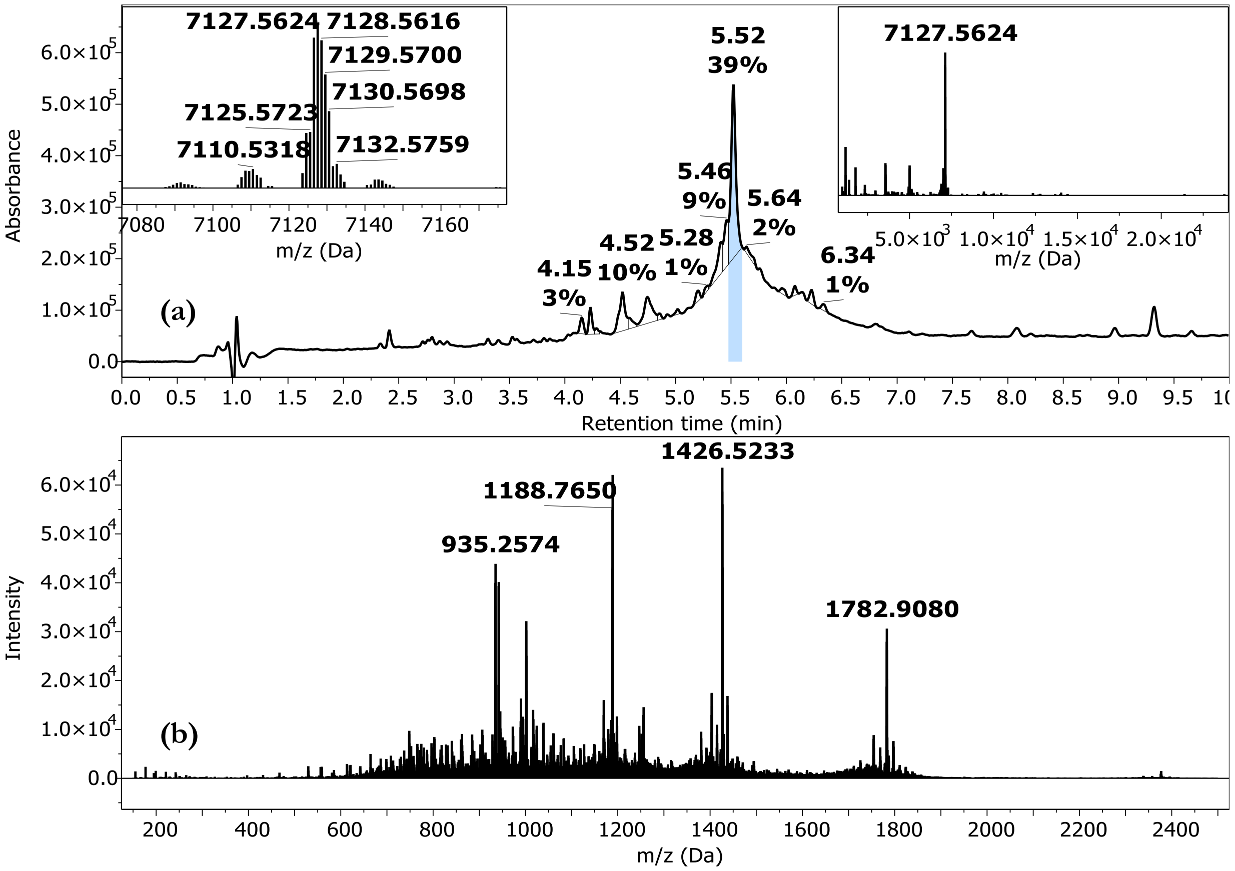

SI Figure 78. LCMS Profile of crude MYC[86–143] bearing (Arg)$_6$. (**a**) Absorbance chromatogram (λ = 214 nm) of MYC[86–143]-(Arg)$_6$; Rt 5.52 min, 39% purity. (**b)** ESI-TOF spectrum found within Rt 4–7 min (insert: deconvoluted masses); Monoisotopic mass (ESI+) calcd. for $C_{302}H_{485}N_{93}O_{101}S_5$ 7123.4008, found 7123.5559 LCMS Gradient A (**Section 2.7**).

UHPLC of crude MYC[86–143]-(Arg)$_6$

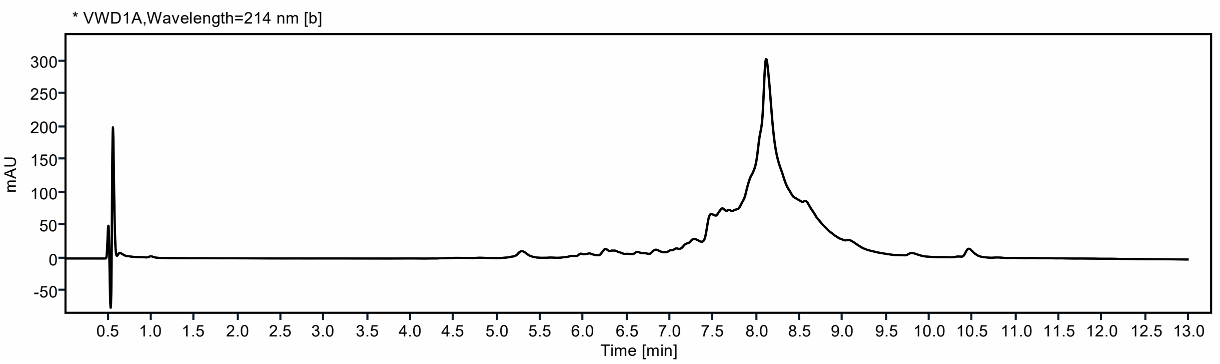

SI Figure 79. UHPLC profile of crude MYC[86–143] bearing (Arg)$_6$. Rt 8.11 min (Agilent Zorbax 300SB-C18 column, 5 µm, 2.1 × 150 mm, 5–95% MeCN over 10 min, ca. 9%B/min), 27% purity based on Area Under Curve (AUC) at λ = 214 nm

3.6.3 Cumulative synthesis traces of MYC[86–143]

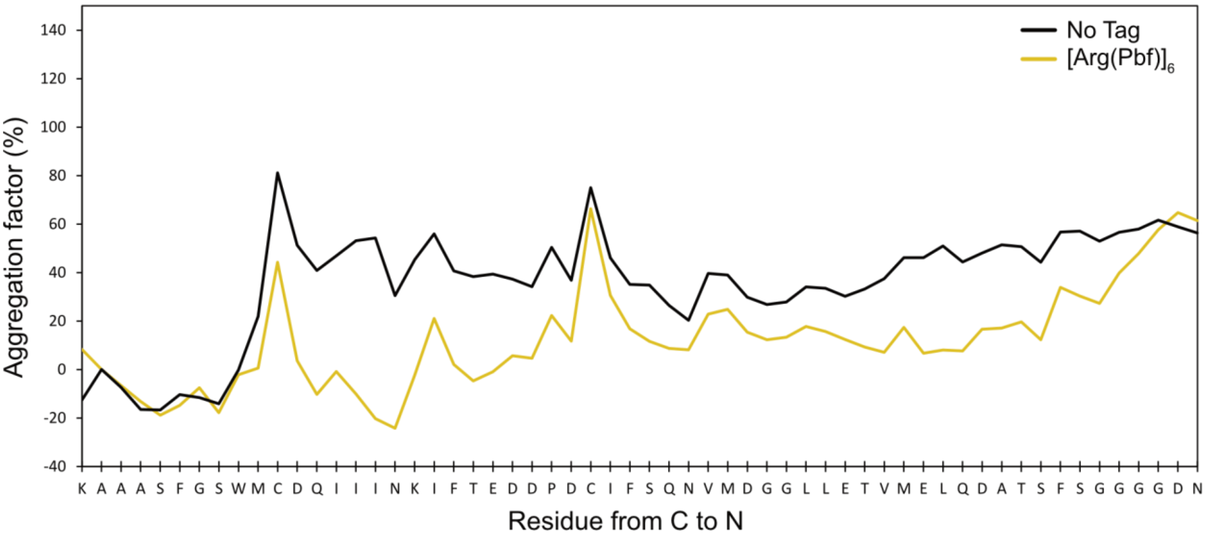

SI Figure 80. Aggregation as a function of Fmoc-deprotection peak broadening by in-line UV-Vis (310 nm) in flow-SPPS for MYC[86–143] with and without [Arg(Pbf)]$_6$ tag, normalized at Ala[142].

3.7 Evaluation of [Arg(Pbf)]$_6$-Tag: Amyloid-β42[27–42]

3.7.1 Amyloid-β42[27–42]: No tag (reference)

```
                      10        15
H2N- KGAIIGLMVG GVVIA-CONH2        (15 AA)
```

The peptide Amyloid-β42[27–42] was synthesized on commercially available Novabiochem® NovaPEG Rink Amide resin (0.41 mmol/g, 50 mg, 20 μmol) using the standard AFPS protocol (**Section 2.2.1**, 40 mL/min flowrate) (**SI Figure 81**). Total synthesis time to afford resin-bound Amyloid-β42[27–42] was approximately 0.6 h. Cleavage of the peptidyl-resin (17 mg, approx. 5.5 μmol) according to Cleavage Protocol A (**Section 2.5.1**) afforded the crude peptide (4.7 mg, 43% purity by LCMS [**SI Figure 82**], 33% purity by UHPLC [**SI Figure 83**]).

UV-Vis synthesis trace

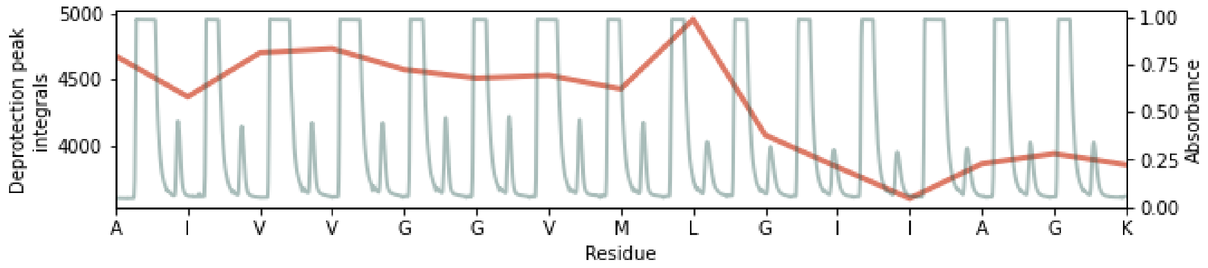

SI Figure 81. UV trace (λ = 310 nm) from AFPS of Amyloid-β42[27–42] (green) and deprotection peak integrals (red).

LC-MS of crude Amyloid-β42[27–42]

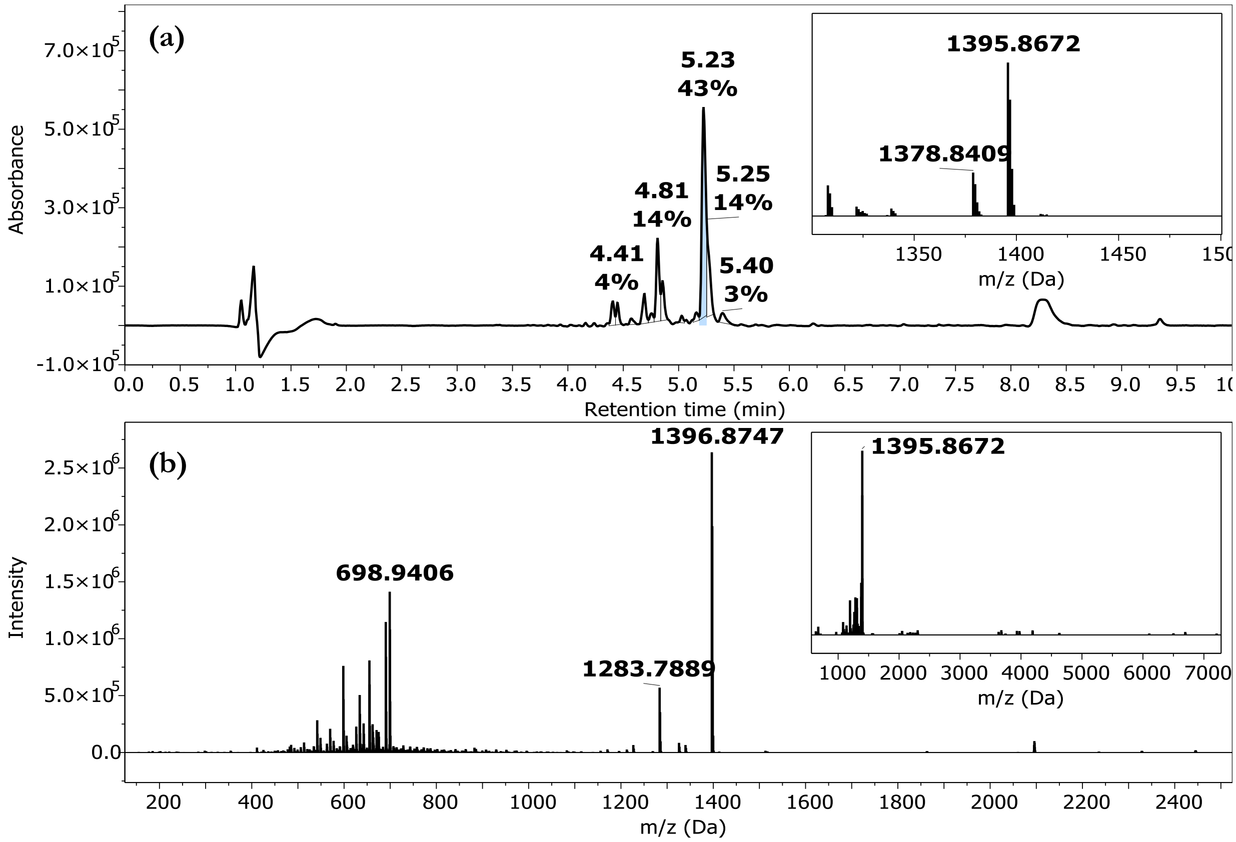

SI Figure 82. LCMS Profile of crude Amyloid-β42[27–42]. (**a**) Absorbance chromatogram (λ = 214 nm) of Aβ42[27–42]; Rt 5.23 min, 43% purity. (**b**) ESI-TOF spectrum found within Rt 2–9 min (insert: deconvoluted masses); Monoisotopic mass (ESI+) calcd. for $C_{64}H_{117}N_{17}O_{15}S$ 1395.8636, found 1395.8672. LCMS Gradient A (**Section 2.7**).

UHPLC of crude Amyloid-β42[27–42]

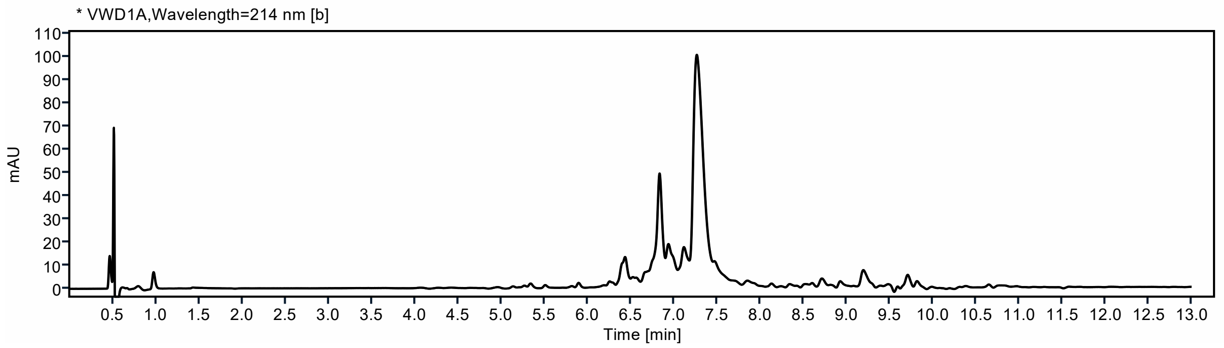

SI Figure 83. UHPLC profile of crude Amyloid-β42[27–42]. Rt 7.27 min (Agilent Zorbax 300SB-C18 column, 5 µm, 2.1 × 150 mm, 5–95% MeCN over 10 min, ca. 9%B/min), 33% purity based on Area Under Curve (AUC) at λ = 214 nm.*

*The peptide was aggregating on the column, leading to a broadening of the peak at Rt 7.27 min.

3.7.2 Amyloid-β42[27–42]: [Arg(Pbf)]$_6$-Tag

```
                10                   20
H2N-KGAIIGLMVG   GVVIARRRRR R-CONH2         (21 AA)
```

The peptide Amyloid-β42[27–42] bearing the [Arg(Pbf)]$_6$ tag was synthesized on commercially available Novabiochem® NovaPEG Rink Amide resin (0.41 mmol/g, 51 mg, 21 µmol) using the standard AFPS protocol (**Section 2.2.1**, 40 mL/min flowrate) (**SI Figure 84**). Total synthesis time

to afford resin-bound Amyloid-β42[27–42] was approximately 0.6 h. Cleavage of the peptidyl-resin (22 mg, approx. 4.1 μmol) according to Cleavage Protocol A (**Section 2.5.1**) afforded the crude peptide (4.4 mg, 62% purity by LCMS [**SI Figure 85**], 48% purity by UHPLC [**SI Figure 86**]).

UV-Vis synthesis trace

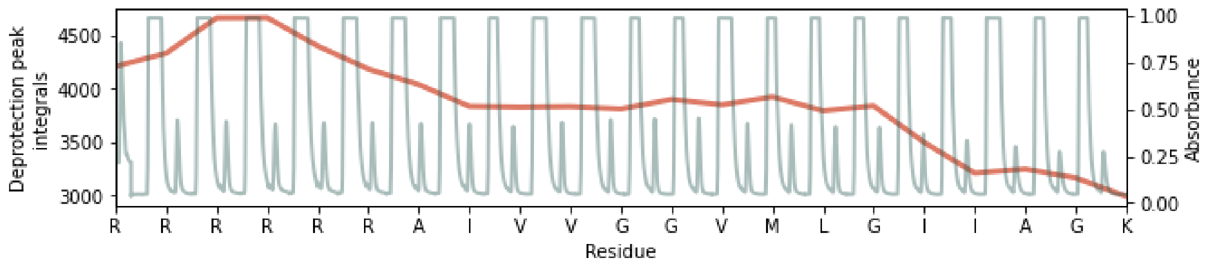

SI Figure 84. UV trace (λ = 310 nm) from AFPS of Amyloid-β42[27–42]-[Arg(Pbf)]$_6$-Tag (green) and deprotection peak integrals (red).

LC-MS of crude Amyloid-β42[27–42]-(Arg)$_6$

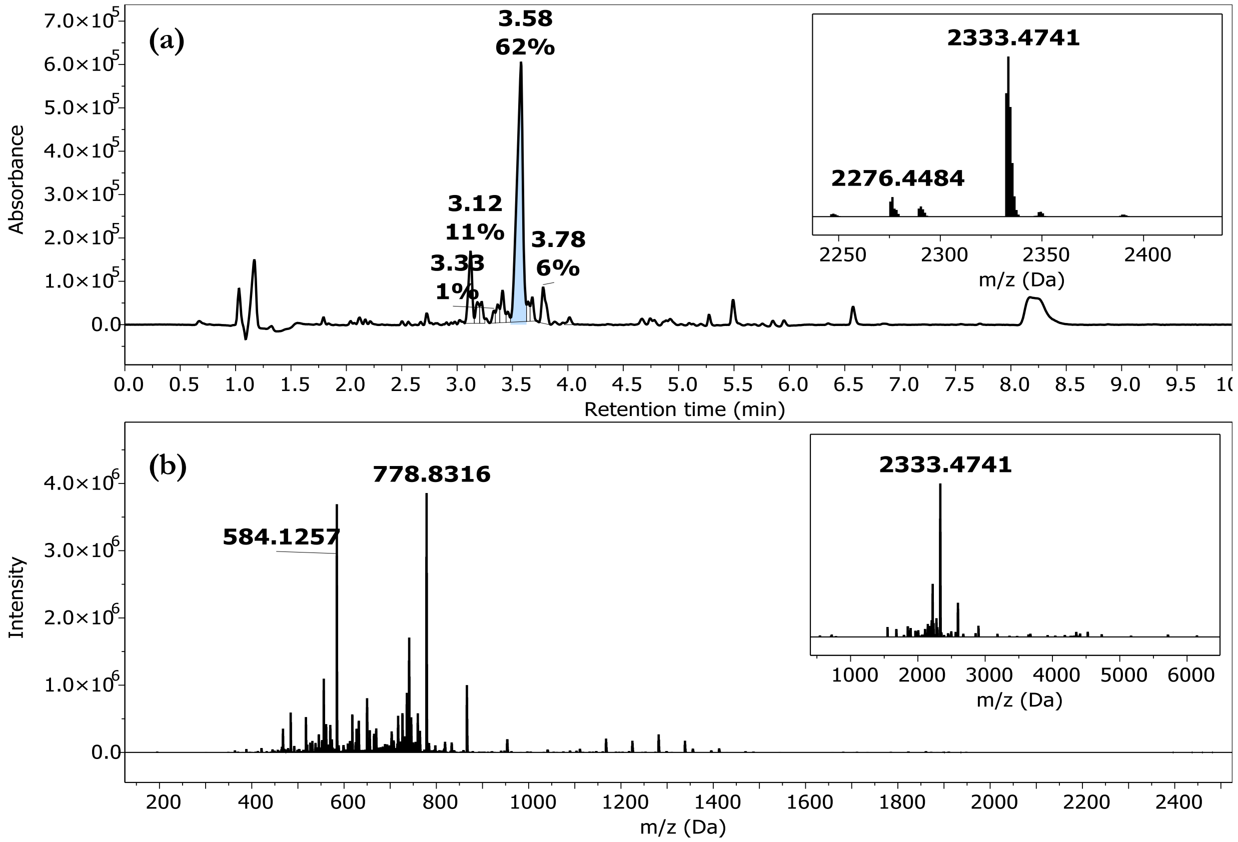

SI Figure 85. LCMS Profile of crude Amyloid-β42[27–42] bearing (Arg)$_6$. (**a**) Absorbance chromatogram (λ = 214 nm) of Aβ42[27–42]-(Arg)$_6$; Rt 3.58 min, 62% purity. (**b**) ESI-TOF spectrum found within Rt 2–9 min (insert: deconvoluted masses); Monoisotopic mass (ESI+) calcd. for $C_{100}H_{189}N_{41}O_{21}S$ 2332.4702, found 2332.4756. LCMS Gradient A (**Section 2.7**).

UHPLC of crude Amyloid-β42[27–42]-(Arg)$_6$

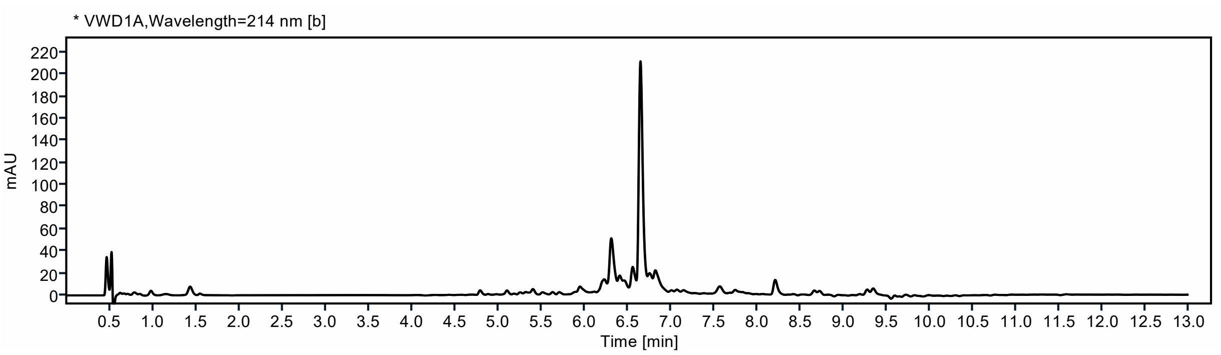

SI Figure 86. UHPLC profile of crude Amyloid-β42[27–42] bearing (Arg)$_6$. Rt 6.65 min (Agilent Zorbax 300SB-C18 column, 5 µm, 2.1 × 150 mm, 5–95% MeCN over 10 min, ca. 9%B/min), 48% purity based on Area Under Curve (AUC) at λ = 214 nm.

3.7.3 Cumulative synthesis traces of Amyloid-β42

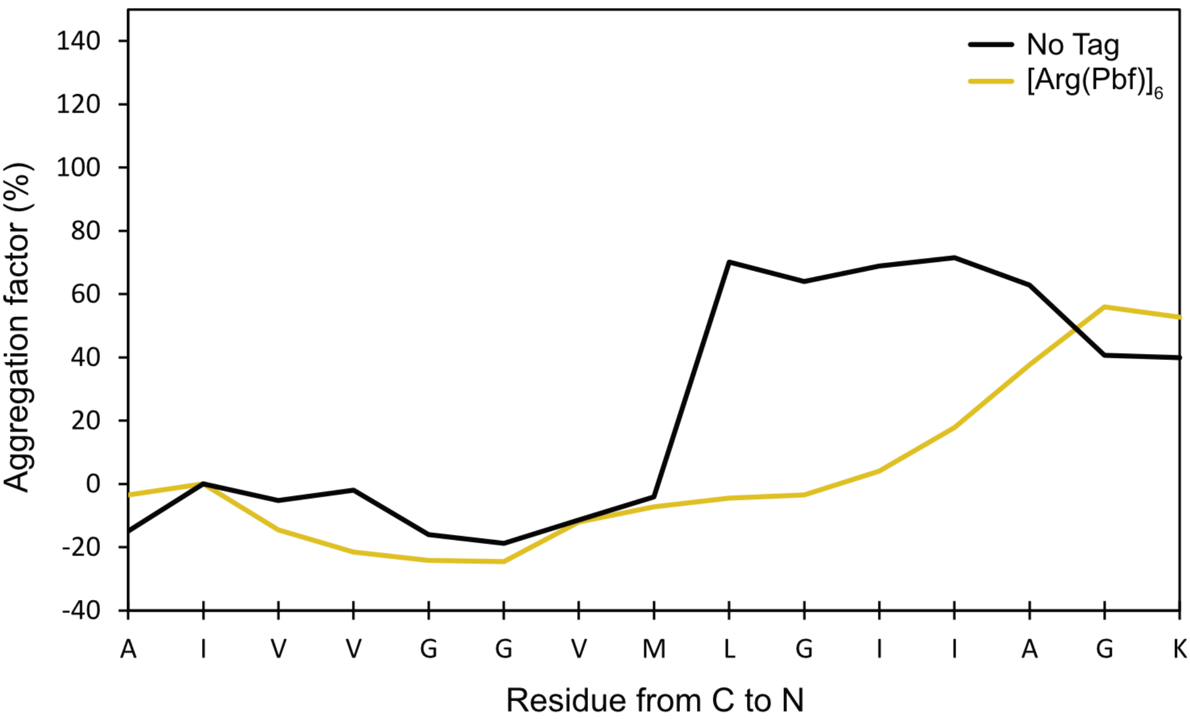

SI Figure 87. Aggregation as a function of Fmoc-deprotection peak broadening by in-line UV-Vis (310 nm) in flow-SPPS for Amyloid-β42[27–42] with and without [Arg(Pbf)]$_6$ tag, normalized at Ile[42].

3.8 Evaluation of [Arg(Pbf)]$_6$-Tag: α-Synuclein[66–82]

3.8.1 α-Synuclein[66–82]: No tag (reference)

```
                       10         17
   H2N-VGGAVVTGVT  AVAQKTV-CONH2    (17 AA)
```

The peptide α-Synuclein[66–82] was synthesized on commercially available Novabiochem® NovaPEG Rink Amide resin (0.41 mmol/g, 50 mg, 20 µmol) using the standard AFPS protocol (**Section 2.2.1**, 40 mL/min flowrate) (**SI Figure 88**). Total synthesis time to afford resin-bound α-synuclein[66–82] was approximately 0.6 h. Cleavage of the peptidyl-resin (15 mg, approx.

4.3 μmol), according to Cleavage Protocol A (**Section 2.5.1**) afforded the crude peptide (7.3 mg, 37% purity by LCMS [**SI Figure 89**], 38% purity by UHPLC [**SI Figure 90**]).

UV-Vis synthesis trace

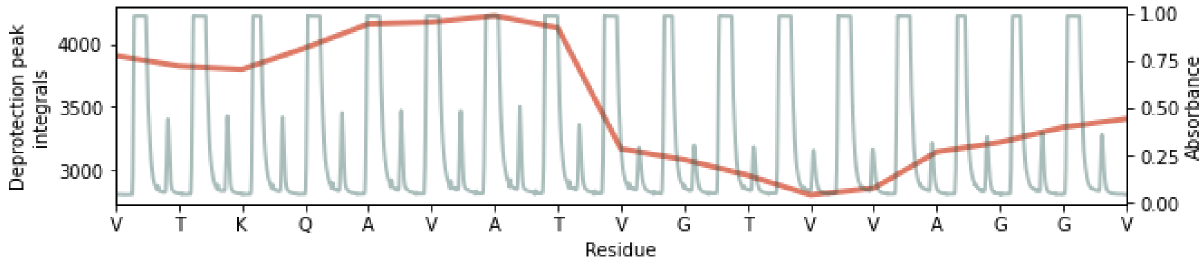

SI Figure 88. UV trace (λ = 310 nm) from AFPS of α-synuclein[66–82] (green) and deprotection peak integrals (red).

LC-MS of crude α-synuclein[66–82]

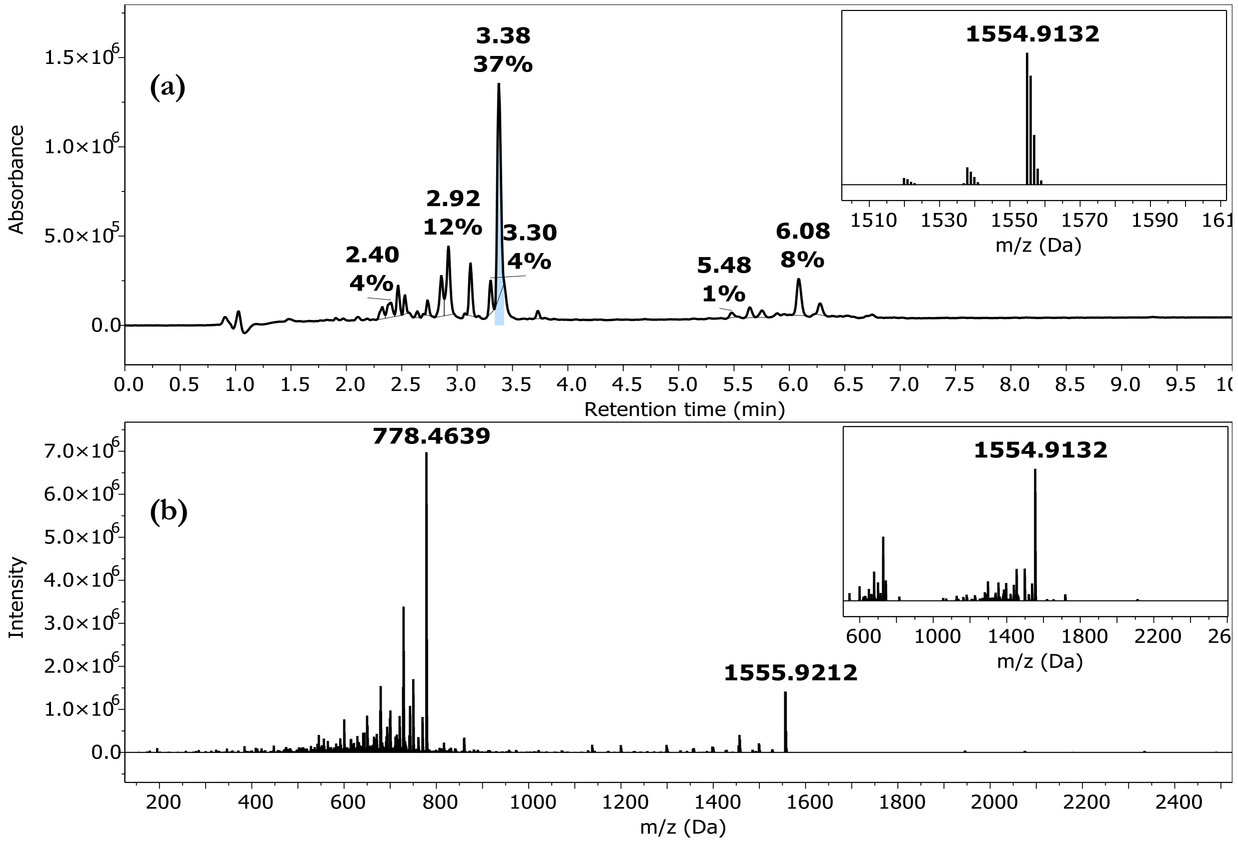

SI Figure 89. LCMS Profile of crude α-synuclein[66–82]. (**a**) Absorbance chromatogram (λ = 214 nm) of α-synuclein[66–82]; Rt 3.38 min, 37% purity. (**b**) ESI-TOF spectrum found within Rt 2–9 min (insert: deconvoluted masses); Monoisotopic mass (ESI+) calcd. for $C_{68}H_{122}N_{20}O_{21}$ 1554.9093, found 1554.9132. LCMS Gradient A (**Section 2.7**).

UHPLC of crude α-synuclein[66–82]

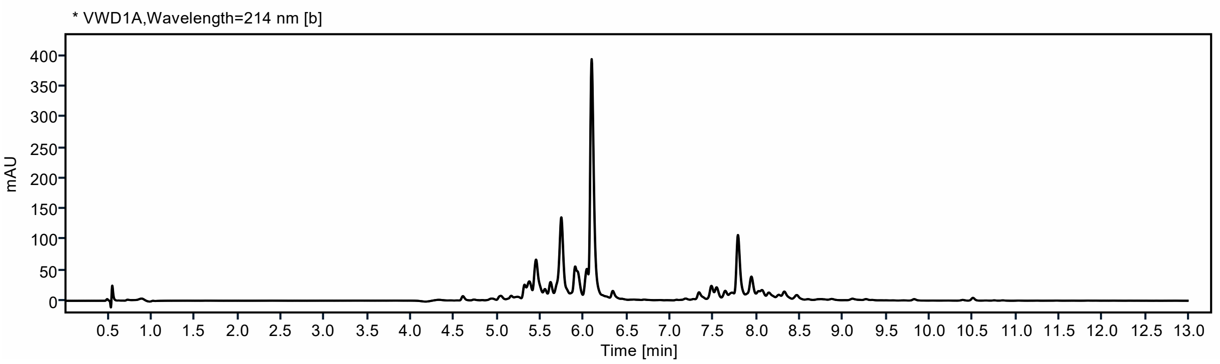

SI Figure 90. UHPLC profile crude α-synuclein[66–82]. Rt 6.09 min (Agilent Zorbax 300SB-C18 column, 5 µm, 2.1 × 150 mm, 5–95% MeCN over 10 min, ca. 9%B/min), 38% purity based on Area Under Curve (AUC) at λ = 214 nm.

3.8.2 α-Synuclein[66–82]: [Arg(Pbf)]$_6$-Tag

```
                 10              20
     H2N-VGGAVVTGVT   AVAQKTVRRR RRR-CONH2      (23 AA)
```

The peptide α-synuclein[66–82] bearing the [Arg(Pbf)]$_6$ tag was synthesized on commercially available Novabiochem® NovaPEG Rink Amide resin (0.41 mmol/g, 49.5 mg, 20 µmol) using the standard AFPS protocol (**Section 2.2.1**, 40 mL/min flowrate) (**SI Figure 91**). Total synthesis time to afford resin-bound α-synuclein[66–82]-[Arg(Pbf)]$_6$ was approximately 1 h. Cleavage of the peptidyl-resin (22 mg, approx. 3.8 µmol), according to Cleavage Protocol A (**Section 2.5.1**) afforded the crude peptide (2.4 mg, 28% purity by LCMS [**SI Figure 92**], 30% purity by UHPLC [**SI Figure 93**]).

UV-Vis synthesis trace

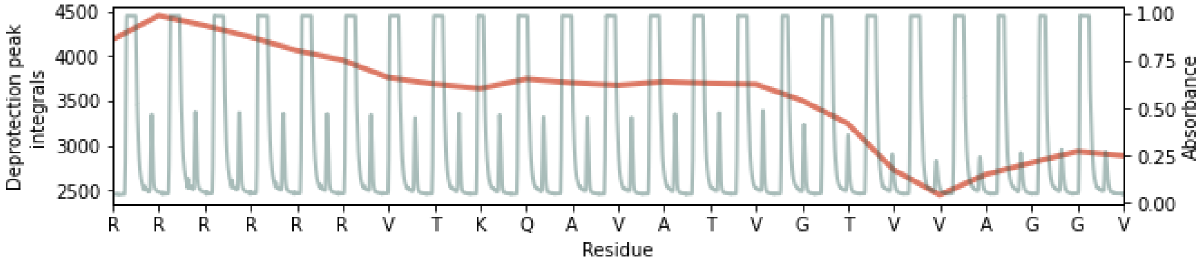

SI Figure 91. UV trace (λ = 310 nm) from AFPS of α-synuclein[66–82] bearing the [Arg(Pbf)]$_6$ tag.

LC-MS of crude α-synuclein[66–82]-(Arg)$_6$

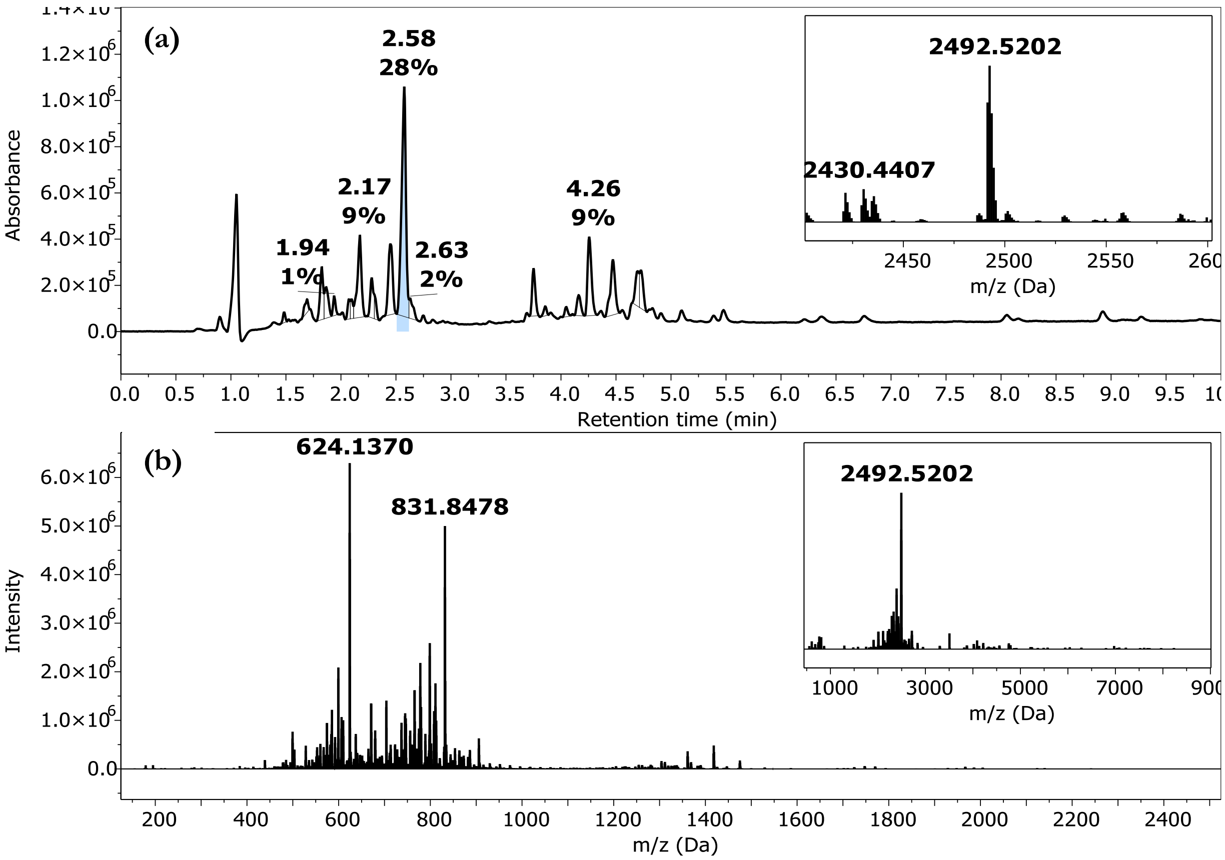

SI Figure 92. LCMS Profile of α-synuclein[66–82] bearing (Arg)$_6$. (**a**) Total absorbance chromatogram (λ = 214 nm) of α-synuclein[66–82]-(Arg)$_6$; Rt 2.58 min, 28% purity. (**b**) ESI-TOF spectrum found within Rt 2–9 min (insert: deconvoluted masses); Monoisotopic mass (ESI+) calcd. for $C_{104}H_{194}N_{44}O_{27}$ 2491.5160, found 2491.5178. LCMS Gradient A (**Section 2.7**).

UHPLC of crude α-synuclein[66–82]-(Arg)$_6$

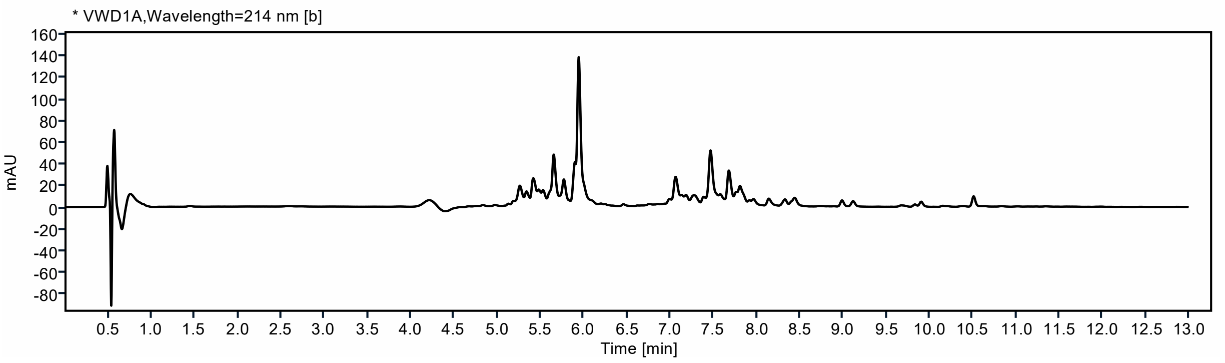

SI Figure 93. UHPLC profile of crude α-synuclein[66–82] bearing (Arg)$_6$. Rt 5.94 min (Agilent Zorbax 300SB-C18 column, 5 µm, 2.1 × 150 mm, 5–95% MeCN over 10 min, ca. 9%B/min), 30% purity based on Area Under Curve (AUC) at λ = 214 nm.

3.8.3 Cumulative synthesis traces of α-synuclein[66–82]

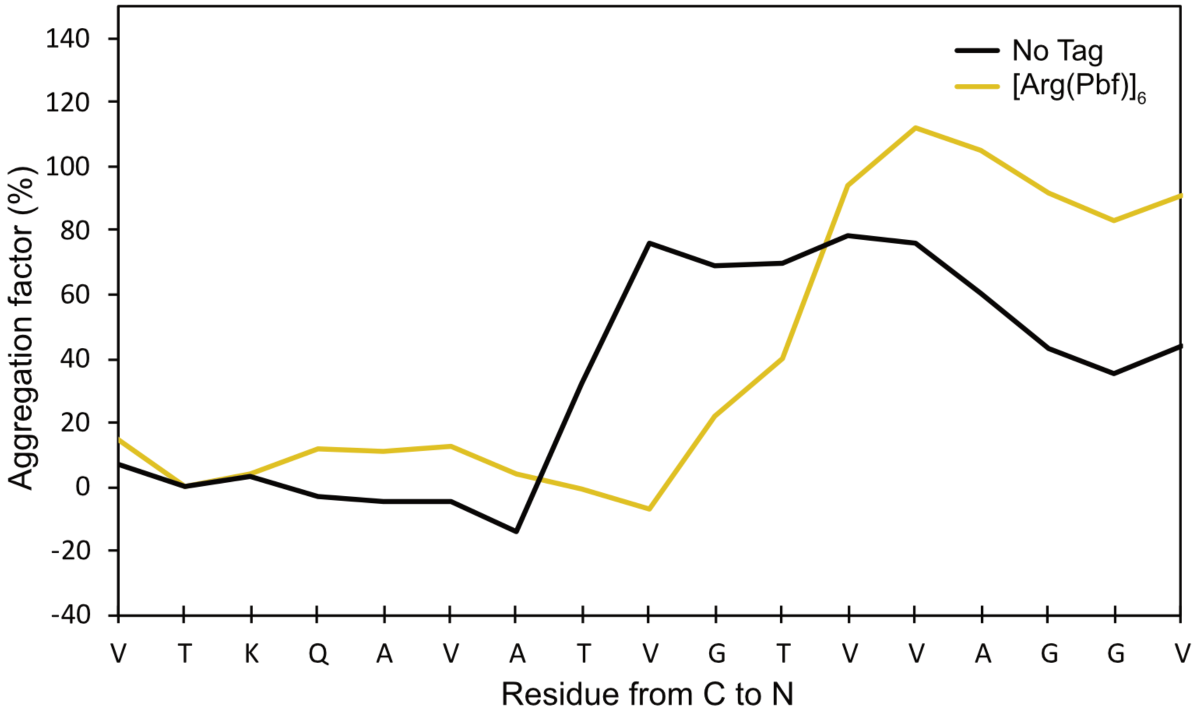

SI Figure 94. Aggregation as a function of Fmoc-deprotection peak broadening by in-line UV-Vis (310 nm) in flow-SPPS for α-synuclein[66–82] with and without $[Arg(Pbf)]_6$ tag, normalized at Thr[81].

3.9 Effect of $[Arg(Pbf)]_6$ Tag position, temperature, and resin loading: Barstar[75–90]

3.9.1 Barstar[75–90]: AFPS synthesis at 70°C – no tag

```
           10        16
H2N-FREAKAEGCD ITIILS-CONH2      (16 AA)
```

The peptide Barstar[75–90] was synthesized on commercially available Novabiochem® NovaPEG Rink Amide resin (0.41 mmol/g, 50 mg, 20 µmol) using the standard AFPS protocol (**Section 2.2.1**, 40 mL/min flowrate), except at 70 °C synthesis temperature (**SI Figure 95**). Total synthesis time to afford resin-bound Barstar[75–90] was approximately 0.8 h. Cleavage of the peptidyl-resin (14 mg, approx. 3.5 µmol) according to Cleavage Protocol A (**Section 2.5.1**) afforded the crude peptide (3.7 mg, 39% purity by LCMS [**SI Figure 96**], 38% purity by UHPLC [**SI Figure 97**]).

UV-Vis synthesis trace

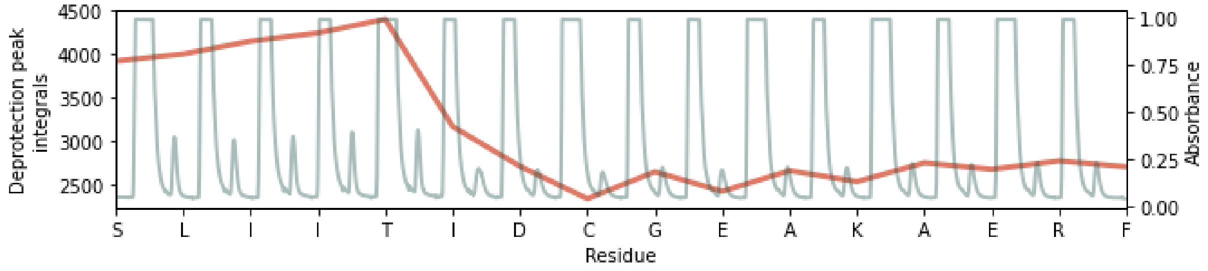

SI Figure 95. UV trace (λ = 310 nm) of Barstar[75–90] synthesized at 70 °C (green) and deprotection peak integrals (red).

LC-MS of crude Barstar[75–90]

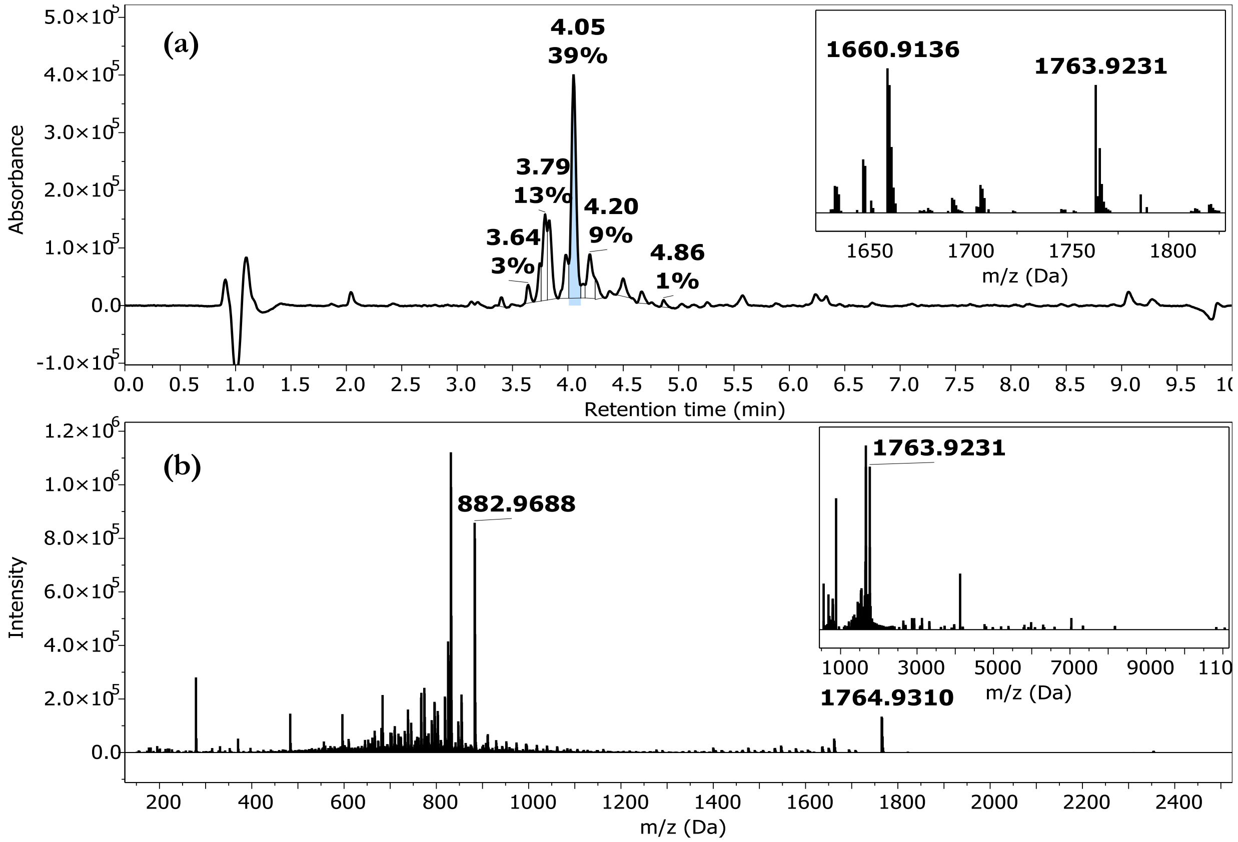

SI Figure 96. LCMS Profile of crude Barstar[75–90] synthesized at 70 °C. (**a**) Absorbance chromatogram (λ = 214 nm) of Barstar[75–90]; Rt 4.05 min, 39% purity. (**b**) ESI-TOF spectrum found within Rt 2–9 min (insert: deconvoluted masses); Monoisotopic mass (ESI+) calcd. for $C_{77}H_{129}N_{21}O_{24}S$ 1763.9240, found 1763.9231. LCMS Gradient A (**Section 2.7**).

UHPLC of crude Barstar[75–90]

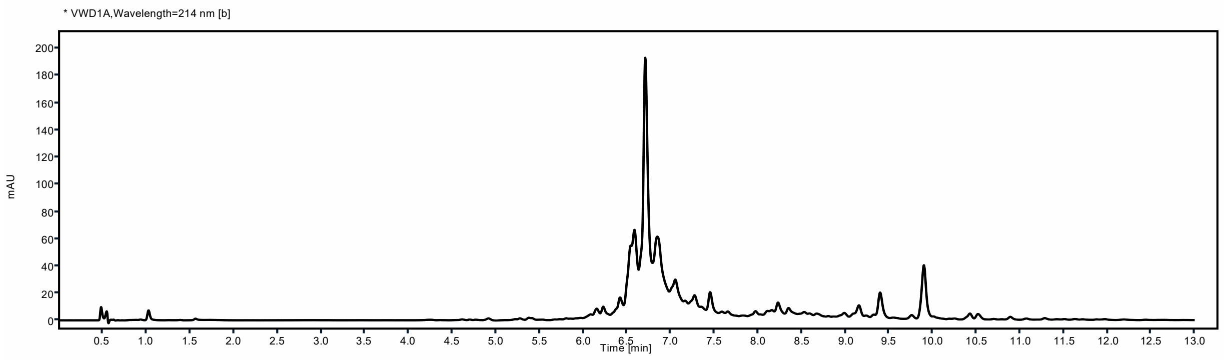

SI Figure 97. UHPLC profile of crude Barstar[75–90] synthesized at 70 °C. Rt 6.71 min (Agilent Zorbax 300SB-C18 column, 5 µm, 2.1 × 150 mm, 5–95% MeCN over 10 min, ca. 9%B/min), 38% purity based on Area Under Curve (AUC) at λ = 214 nm.

3.9.2 Barstar[75–90]: AFPS synthesis at 70 °C – [Arg(Pbf)$_6$]-tag C-terminus

```
          10         20
H2N-FREAKAEGCD  ITIILSRRRR RR-CONH2        (22 AA)
```

The peptide Barstar[75–90] bearing the [Arg(Pbf)$_6$] tag was synthesized on commercially available Novabiochem® NovaPEG Rink Amide resin (0.41 mmol/g, 51 mg, 21 µmol) using the standard AFPS protocol (**Section 2.2.1**, 40 mL/min flow-rate), except at 70 °C synthesis temperature (**SI Figure 98**). Total synthesis time to afford resin-bound Barstar[75–90]-[Arg(Pbf)$_6$] was

approximately 0.8 h. Cleavage of the peptidyl-resin (18 mg, approx. 3.3 μmol) according to Cleavage Protocol A (**Section 2.5.1**) afforded the crude peptide (3.4 mg, 63% purity by LCMS [**SI Figure 99**], 61% purity by UHPLC [**SI Figure 100**]).

UV-Vis synthesis trace

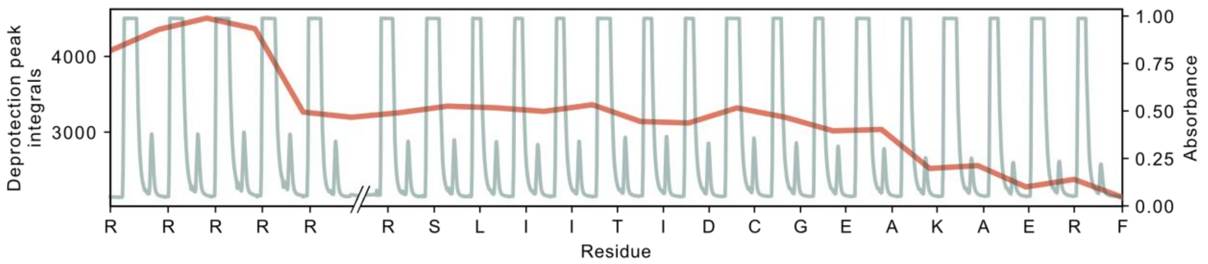

SI Figure 98. UV trace (λ = 310 nm) from AFPS Barstar[75–90]-[Arg(Pbf)]$_6$ synthesized at 70 °C (green) and deprotection peak integrals (red). The synthesis was briefly paused for approx. 5 min after the 5th coupling [Arg].

LC-MS of crude Barstar[75–90]-(Arg)$_6$

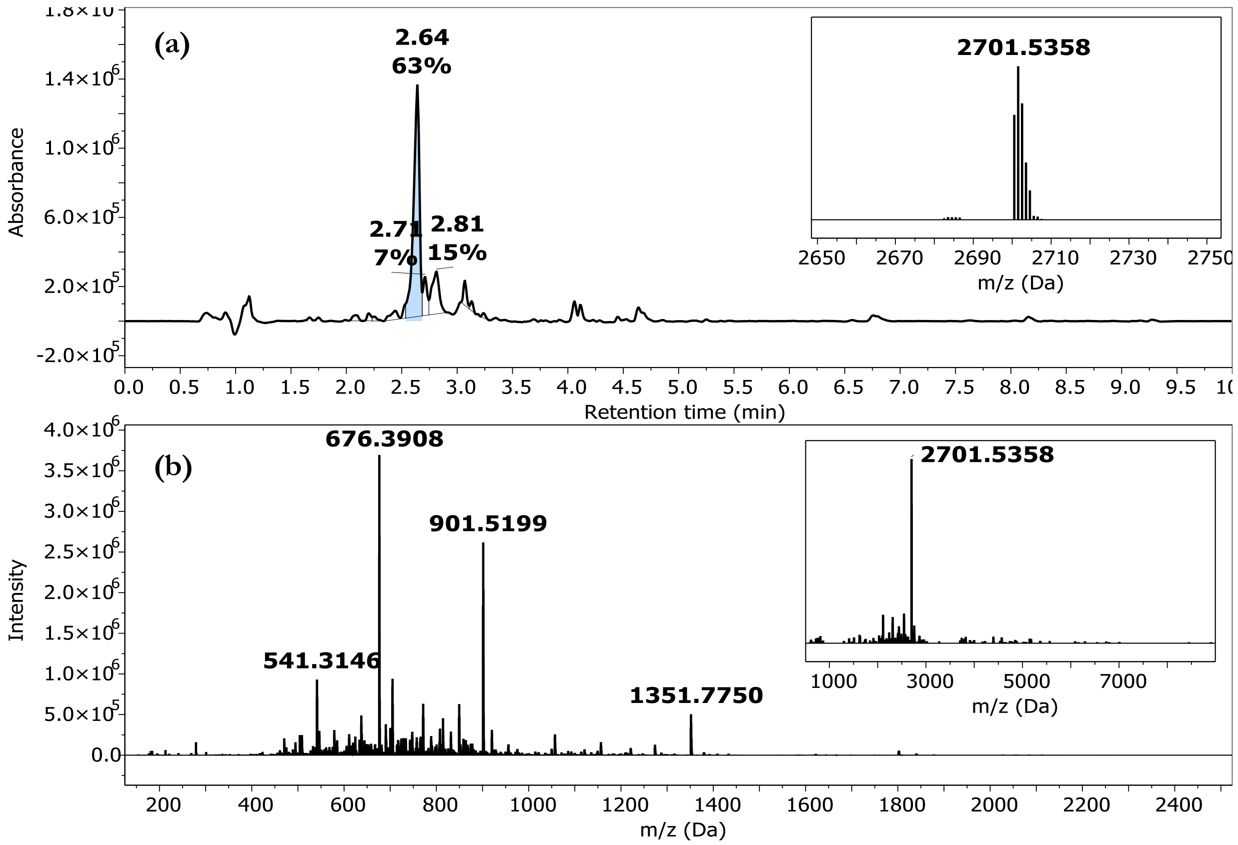

SI Figure 99. LCMS Profile of crude Barstar[75–90] bearing (Arg)$_6$ synthesized at 70 °C. (**a**) Absorbance chromatogram (λ = 214 nm) of Barstar[75–90]-(Arg)$_6$; Rt 2.64 min, 63% purity. (**b**) ESI-TOF spectrum found within Rt 2–9 min (insert: deconvoluted masses); Monoisotopic mass (ESI+) calcd. for $C_{113}H_{200}N_{44}O_{31}S$ 2700.5307, found 2700.5330. LCMS Gradient A (**Section 2.7**).

UHPLC of crude Barstar[75–90]-(Arg)$_6$

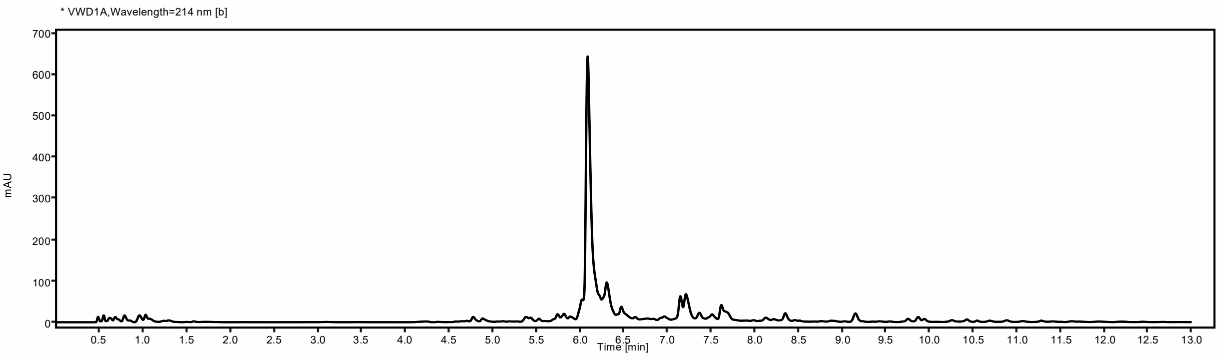

SI Figure 100. UHPLC profile of crude Barstar[75–90] bearing (Arg)$_6$ synthesized at 70 °C. Rt 6.09 min (Agilent Zorbax 300SB-C18 column, 5 µm, 2.1 × 150 mm, 5–95% MeCN over 10 min, ca. 9%B/min), 61% purity based on Area Under Curve (AUC) at λ = 214 nm.

3.9.3 Cumulative synthesis traces of Barstar[75–90] synthesized at 90 °C and 70 °C.

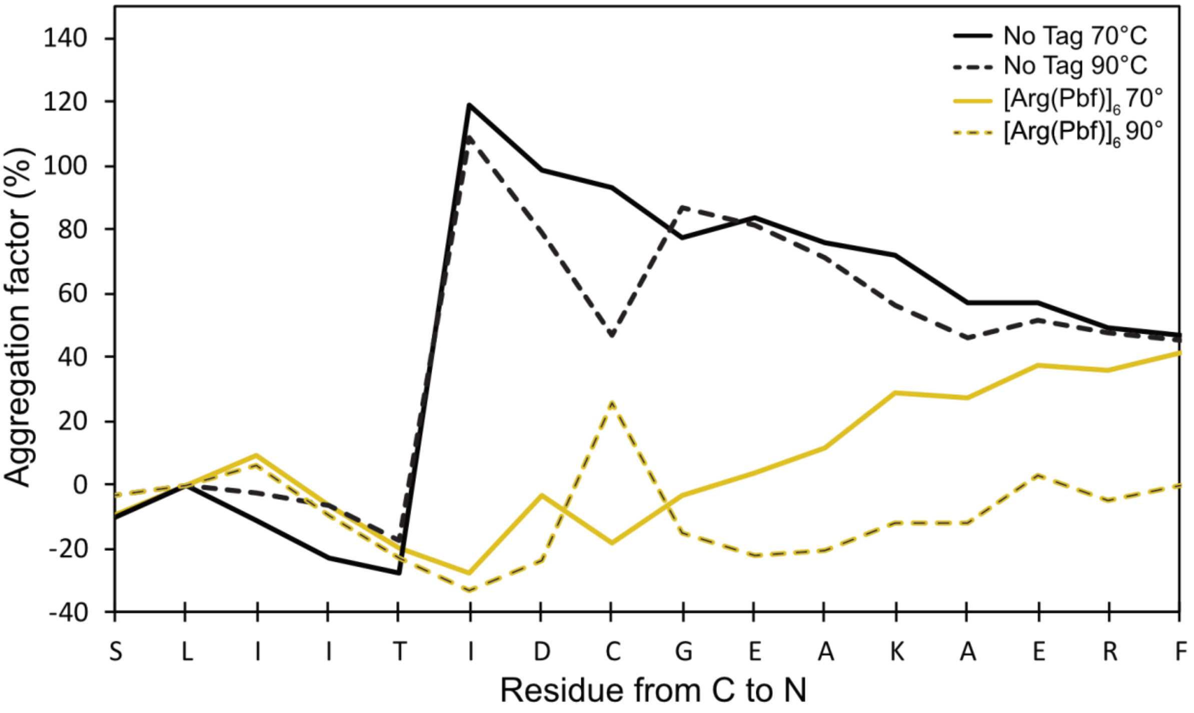

SI Figure 101. Aggregation as a function of Fmoc-deprotection peak broadening by in-line UV-Vis (310 nm) in flow-SPPS for Barstar[75–90] with and without amino acid tags, synthesized at 70 °C and 90 °C, normalized at Leu[89].

3.9.4 Barstar[75–90]: AFPS synthesis on low-loading resin: no tag

```
           10         16
H2N-FREAKAEGCD ITIILS-CONH2      (16 AA)
```

The peptide Barstar[75–90] was synthesized on commercially available Novabiochem® NovaPEG Rink Amide resin (0.20 mmol/g, 0.15 g, 30 µmol) using the standard AFPS protocol (**Section 2.2.1**, 40 mL/min flowrate) (**SI Figure 102**). Total synthesis time to afford resin-bound Barstar[75–90] was approximately 0.8 h. Cleavage of the peptidyl-resin (24 mg, approx. 3.7 µmol) according to Cleavage Protocol A (**Section 2.5.1**) afforded the crude peptide (4.5 mg, 79% purity by LCMS [**SI Figure 103**], 77% purity by UHPLC [**SI Figure 104**]).

UV-Vis synthesis trace

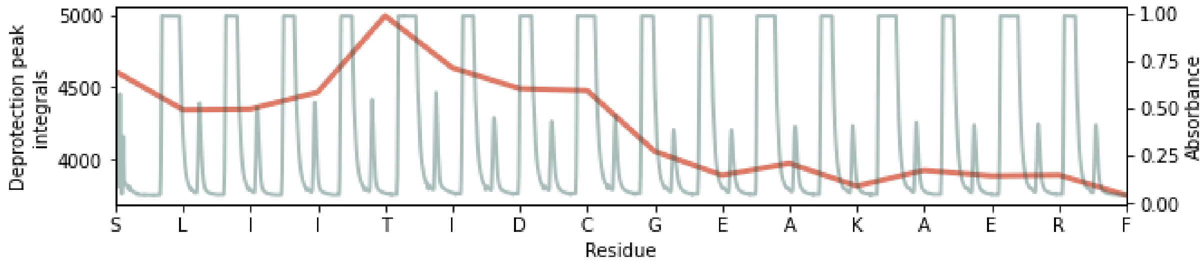

SI Figure 102. UV trace (λ = 310 nm) from AFPS of Barstar[75–90] on low-loading resin (0.20 mmol/g) (green) and deprotection peak integrals (red).

LC-MS of crude Barstar[75–90]

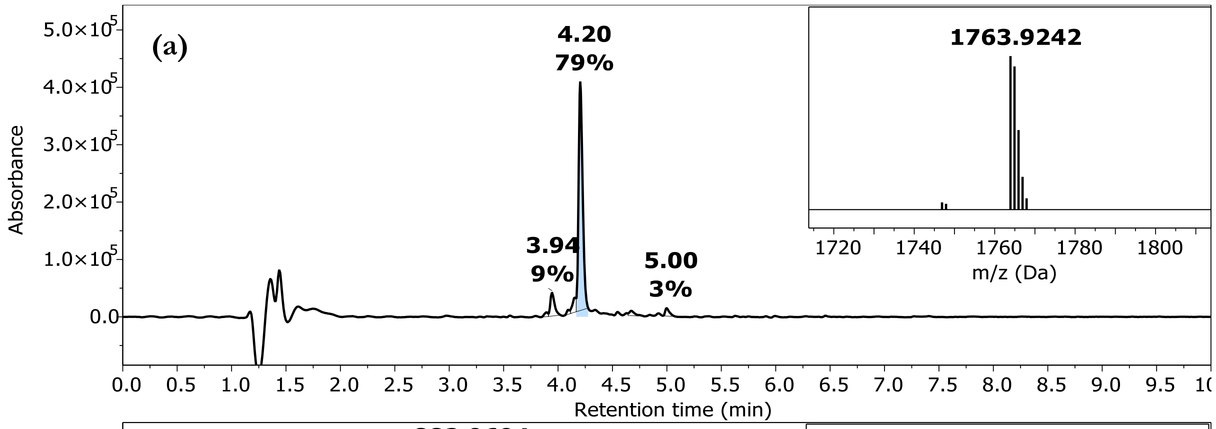

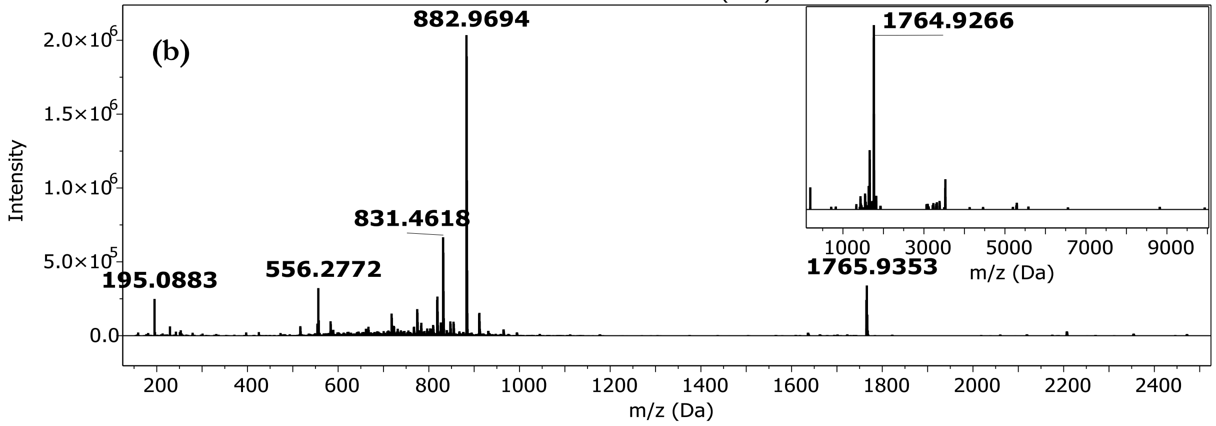

SI Figure 103. LCMS Profile of crude Barstar[75–90] synthesized on low-loading resin (0.20 mmol/g). (**a**) Absorbance chromatogram (λ = 214 nm) of Barstar[75–90]; Rt 4.20 min, 79% purity. (**b** ESI-TOF spectrum found within Rt 2–9 min (insert: deconvoluted masses); Monoisotopic mass (ESI+) calcd. for $C_{77}H_{129}N_{21}O_{24}S$ 1763.9240, found 1763.9242 LCMS Gradient A (**Section 2.7**).

UHPLC of crude Barstar[75–90]

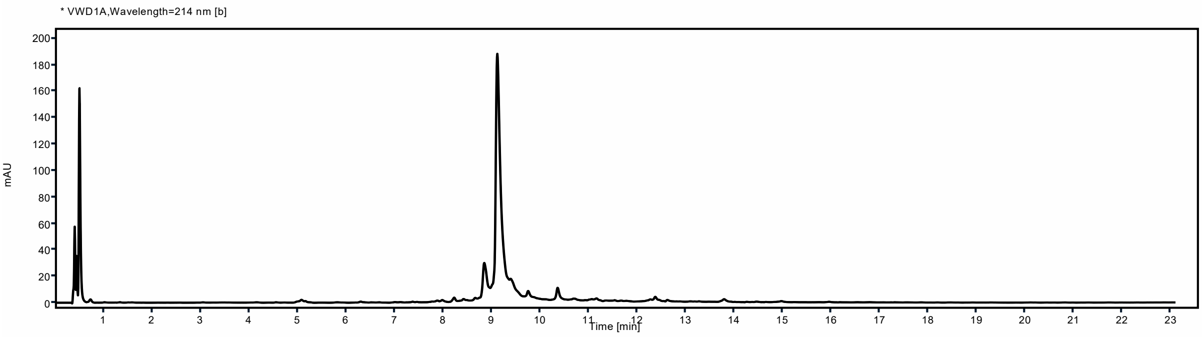

SI Figure 104. UHPLC profile of crude Barstar[75–90] synthesized on low-loading resin (0.20 mmol/g). Rt 9.107 min (Agilent Zorbax 300SB-C18 column, 5 µm, 2.1 × 150 mm, 5–95% MeCN over 20 min, ca. 4.5%B/min), 77% purity based on Area Under Curve (AUC) at λ = 214 nm.

3.9.5 Barstar[75–90]: AFPS synthesis on low-loading resin: [Arg(Pbf)]$_6$-Tag

```
              10                 20
H2N-FREAKAEGCD  ITIILSRRRR RR-CONH2        (22 AA)
```

The peptide Barstar[75–90] bearing the [Arg(Pbf)]$_6$ tag was synthesized on commercially available Novabiochem® NovaPEG Rink Amide resin (0.20 mmol/g, 150 mg, 30 µmol) using the standard AFPS protocol (**Section 2.2.1**, 40 mL/min flowrate) (**SI Figure 105**). Total synthesis time to afford resin-bound Barstar[75–90]-[Arg(Pbf)]$_6$ was approximately 1 h. Cleavage of the peptidyl-resin (38 mg, approx. 4.5 µmol) according to Cleavage Protocol A (**Section 2.5.1**) afforded the crude peptide (3.4 mg, 73% purity by LCMS [**SI Figure 106**], 80% purity by UHPLC [**SI Figure 107**]).

UV-Vis synthesis trace

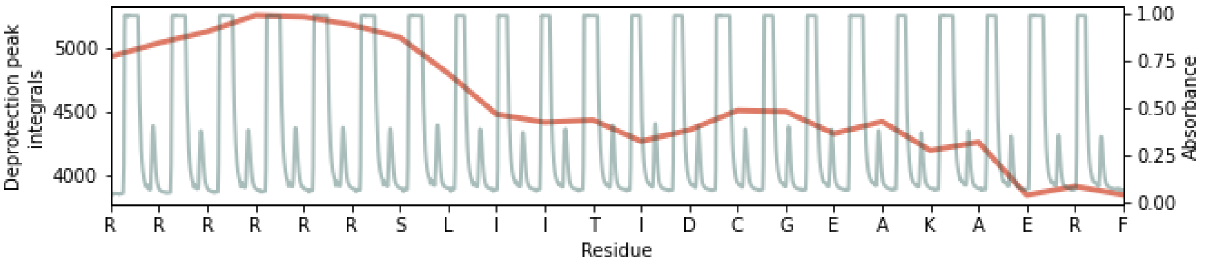

SI Figure 105. UV trace (λ = 310 nm) of Barstar[75–90] synthesized at 70°C on [Arg(Pbgf)]$_6$ (green) and deprotection peak integrals (red).

LC-MS of crude Barstar[75–90]-(Arg)$_6$

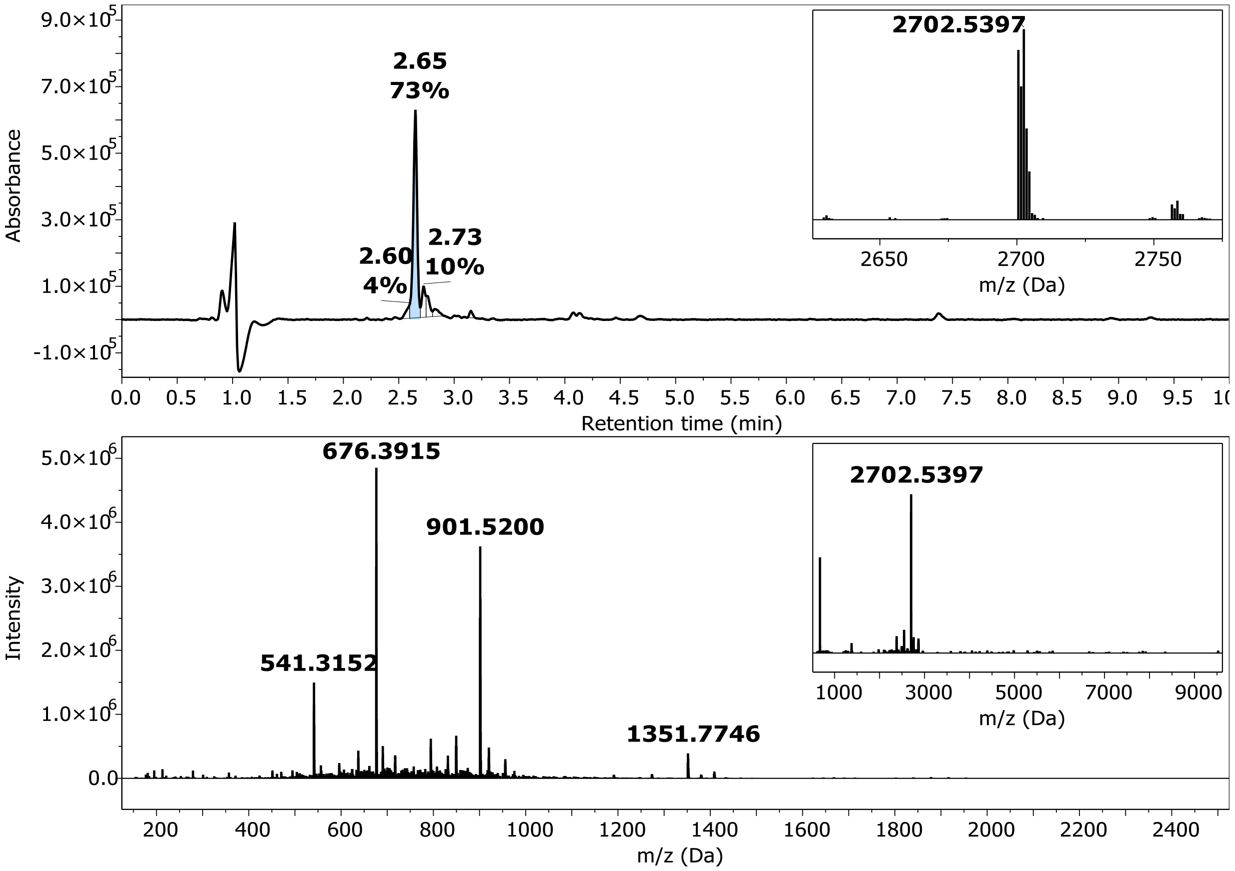

SI Figure 106. LCMS Profile of crude Barstar[75–90] bearing (Arg)$_6$. (**a**) Absorbance chromatogram (λ = 214 nm) of Barstar[75–90]-(Arg)$_6$; Rt 2.65 min, 73% purity. (**b**) ESI-TOF spectrum found within Rt 2–9 min (insert: deconvoluted masses); Monoisotopic mass (ESI+) calcd. for $C_{113}H_{200}N_{44}O_{31}S$ 2700.5307, found 2700.5345. LCMS Gradient A (**Section 2.7**).

UHPLC of crude Barstar[75–90]-(Arg)$_6$

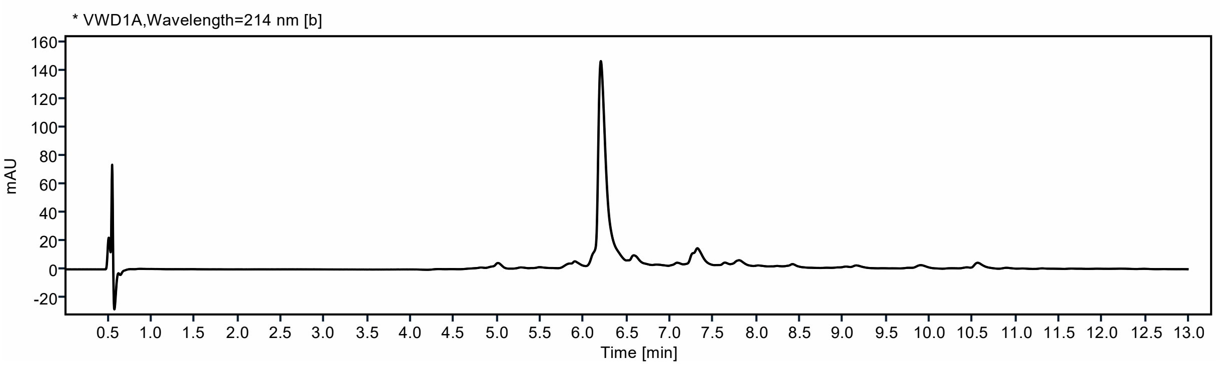

SI Figure 107. UHPLC profile of crude Barstar[75–90] bearing (Arg)$_6$ synthesized on low-loading resin. Rt 6.20 min (Agilent Zorbax 300SB-C18 column, 5 µm, 2.1 × 150 mm, 5–95% MeCN over 10 min, ca. 9%B/min), 80% purity based on Area Under Curve (AUC) at λ = 214 nm.

3.9.6 Cumulative synthesis traces of Barstar[75–90] on high and low loading resin with and without [Arg(Pbf)]$_6$-Tag.

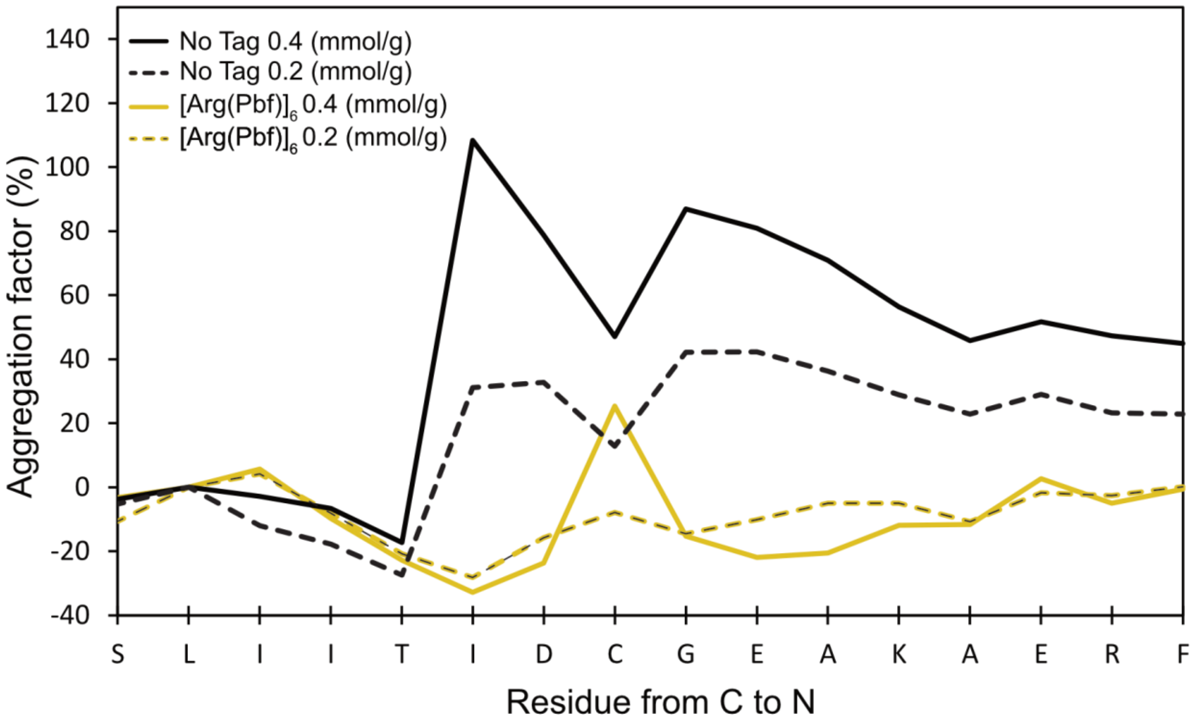

SI Figure 108. Aggregation as a function of Fmoc-deprotection peak broadening by in-line UV-Vis (310 nm) in flow-SPPS for Barstar[75–90] with and without amino acid tags, on two different resin loadings, normalized at Leu[89].

3.9.7 Batch synthesis at room temperature (23 °C): no tag

```
          10         16
H2N-FREAKAEGCD ITIILS-CONH2        (16 AA)
```

The peptide Barstar[75–90] was synthesized on commercially available Novabiochem® NovaPEG Rink Amide resin (0.41 mmol/g, 50 mg, 21 µmol) using the batch SPPS standard protocol (**Section 2.2.4**) at room temperature. Cleavage of the peptidyl-resin (16 mg, approx. 4.1 µmol) according to Cleavage Protocol A (**Section 2.5.1**) afforded the crude peptide (3.8 mg, 55% purity by LCMS [**SI Figure 109**], 55% purity by UHPLC [**SI Figure 110**]).

LC-MS of crude Barstar[75–90]

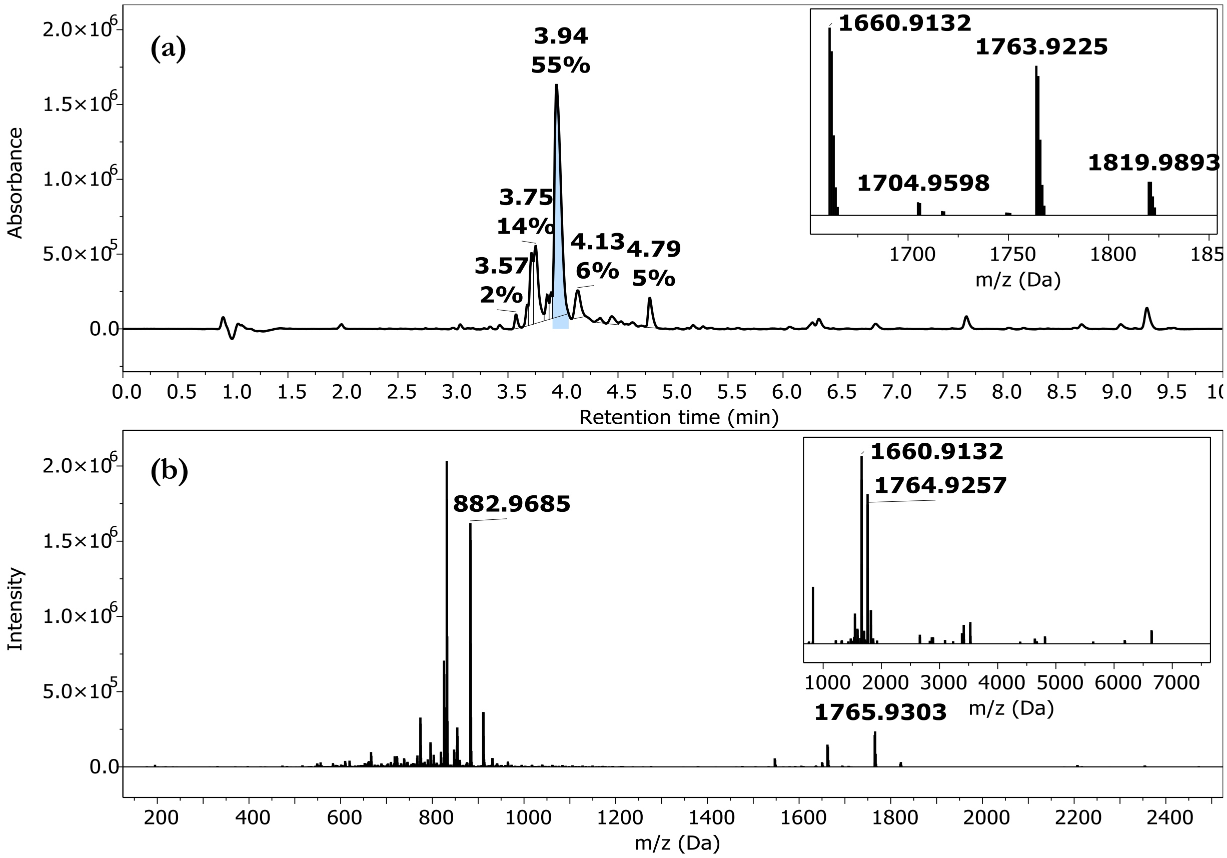

SI Figure 109. LCMS Profile of crude Barstar[75–90] synthesized at room temperature (23 °C) by batch SPPS. (**a**) Absorbance chromatogram (λ = 214 nm) of Barstar[75–90]; Rt 3.94 min, 55% purity. (**b**) ESI-TOF spectrum found within Rt 2–9 min (insert: deconvoluted masses); Monoisotopic mass (ESI+) calcd. for $C_{77}H_{129}N_{21}O_{24}S$ 1763.9240, found 1763.9225. LCMS Gradient A (**Section 2.7**).

UHPLC of crude Barstar[75–90]

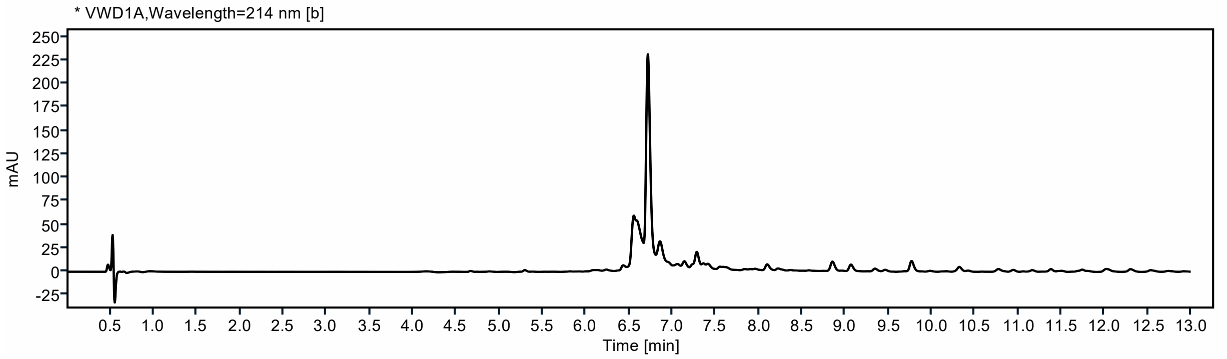

SI Figure 110. UHPLC profile of crude Barstar[75–90] synthesized at room temperature (23 °C) by batch SPPS. Rt 6.71 min (Agilent Zorbax 300SB-C18 column, 5 µm, 2.1 × 150 mm, 5–95% MeCN over 10 min, ca. 9%B/min), 55% purity based on Area Under Curve (AUC) at λ = 214 nm.

3.9.8 Barstar[75–90]: Batch synthesis at room temperature (23 °C) – [Arg(Pbf)$_6$]-tag at C-terminus

```
          10          20
H2N-FREAKAEGCD ITIILSRRRR RR-CONH2        (22 AA)
```

The peptide Barstar[75–90] bearing the [Arg(Pbf)$_6$]-tag was synthesized on commercially available Novabiochem® NovaPEG Rink Amide resin (0.41 mmol/g, 50 mg, 21 µmol) using the batch SPPS standard protocol (**Section 2.2.4**) at 23 °C. Cleavage of the peptidyl-resin (11 mg, approx. 4.5 µmol) according to Cleavage Protocol A (**Section 2.5.1**) afforded the crude peptide (1.5 mg, 59% purity by LCMS [**SI Figure 111**], 69% purity by UHPLC [**SI Figure 112**]).

LC-MS of crude Barstar[75–90]-(Arg)$_6$

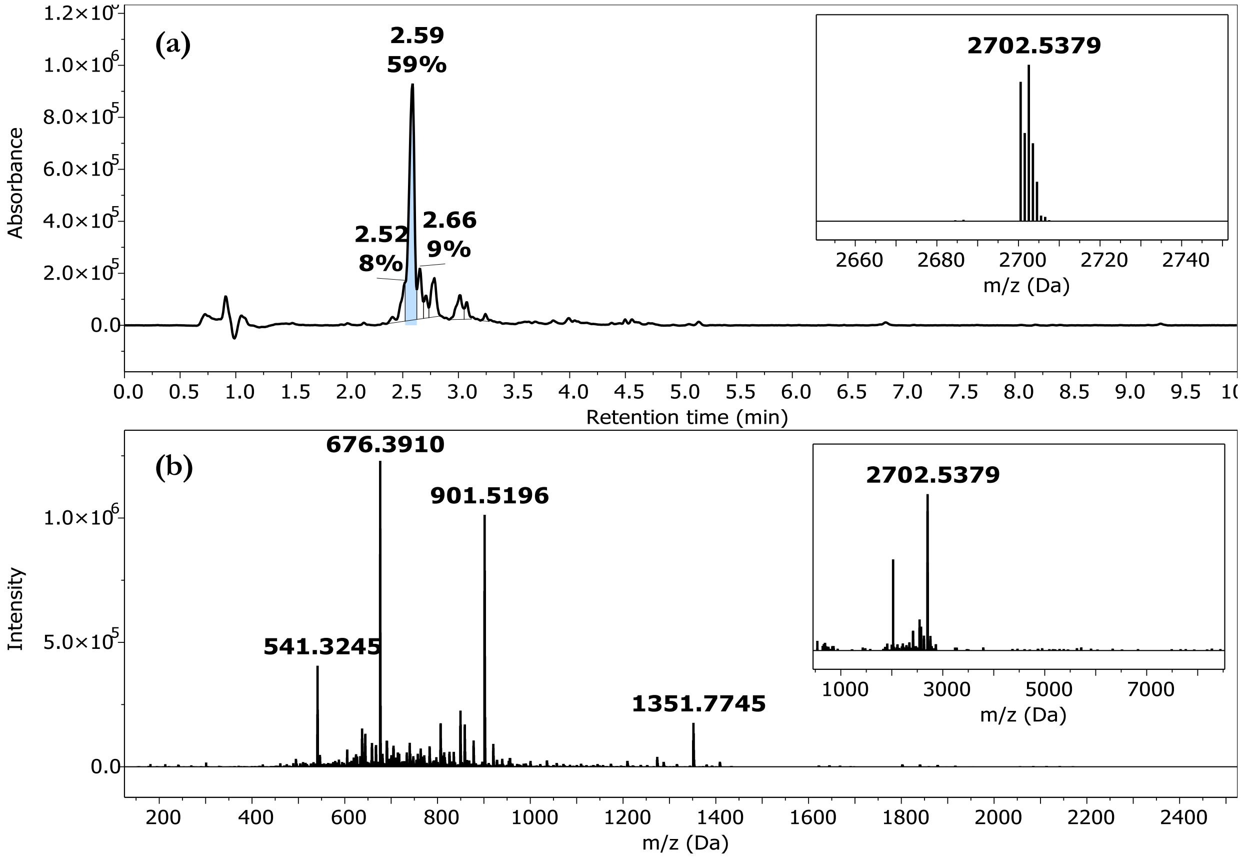

SI Figure 111. LCMS Profile of crude Barstar[75–90] bearing (Arg)$_6$ at the C-terminus, synthesized at room temperature (23 °C) using batch SPPS. (**a**) Absorbance chromatogram (λ = 214 nm) of Barstar[75–90]-(Arg)$_6$; Rt 2.59 min, 59% purity. (**b**) ESI-TOF spectrum found within Rt 2–9 min (insert: deconvoluted masses); Monoisotopic mass (ESI+) calcd. for $C_{113}H_{200}N_{44}O_{31}S$ 2700.5307, found 2700.5327. LCMS Gradient A (**Section 2.7**).

UHPLC of crude Barstar[75–90]-(Arg)$_6$

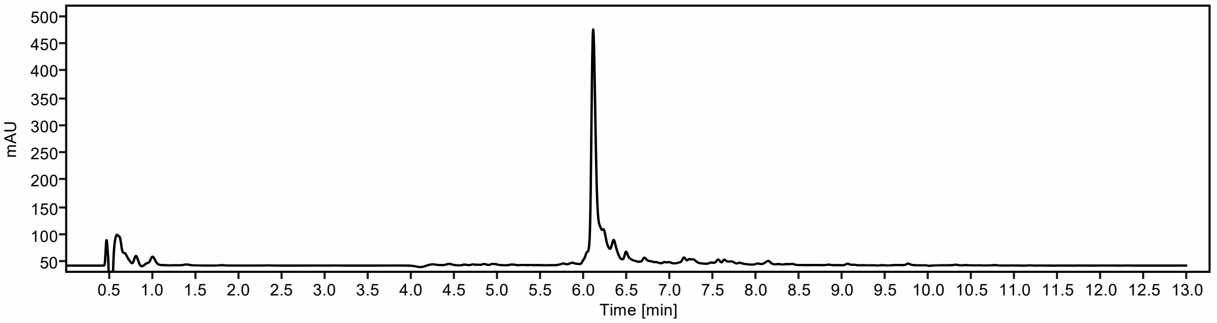

SI Figure 112. UHPLC profile of crude Barstar[75–90] bearing (Arg)$_6$ at the C-terminus, synthesized at room temperature (23 °C) using batch SPPS. Rt 6.11 min (Agilent Zorbax 300SB-C18 column, 5 µm, 2.1 × 150 mm, 5–95% MeCN over 10 min, ca. 9%B/min), 69% purity based on Area Under Curve (AUC) at λ = 214 nm.

3.9.9 Barstar[75–90]: Batch synthesis at room temperature (23 °C) – [Arg(Pbf)$_6$]-tag at N-terminus

```
             10                  20
H2N-RRRRRRFREA  KAEGCDITII LS-CONH2        (22 AA)
```

Upon resin-bound Barstar[75–90] (18.5 mg, approx. 36 µmol) prepared by batch SPPS (see **Section 3.9.7**), [Arg(Pbf)]$_6$ was added to the N-terminus using the batch SPPS standard protocol (**Section 2.2.4**) at 23 °C. Cleavage of the peptidyl-resin (9.5 mg, approx. 11 µmol) according to Cleavage Protocol A (**Section 2.5.1**) afforded the crude peptide (2.0 mg, 21% purity by LCMS [**SI Figure 113**], 33% purity by UHPLC [**SI Figure 114**]).

LC-MS of crude (Arg)$_6$-Barstar[75–90]

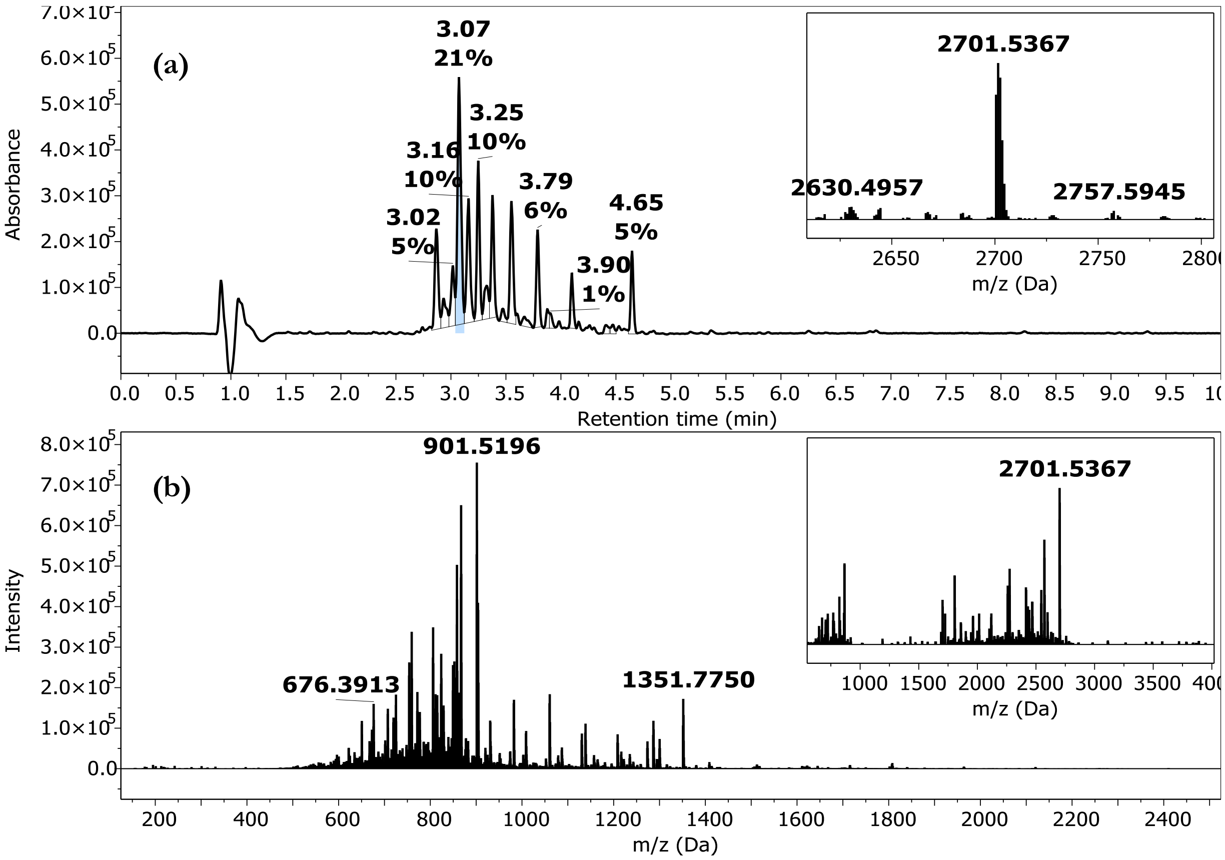

SI Figure 113. LCMS Profile of Barstar[75–90] bearing (Arg)$_6$ at the N-terminus, synthesized at room temperature (23 °C) using batch SPPS. (**a**) Absorbance chromatogram (λ = 214 nm) of (Arg)$_6$-Barstar[75–90]; Rt 3.07 min, 21% purity. (**b**) ESI-TOF spectrum found within Rt 2–9 min (insert: deconvoluted masses); Monoisotopic mass (ESI+) calcd. for $C_{113}H_{200}N_{44}O_{31}S$ 2700.5307, found 2700.5334. LCMS Gradient A (**Section 2.7**).

UHPLC of crude $(Arg)_6$-Barstar[75–90]

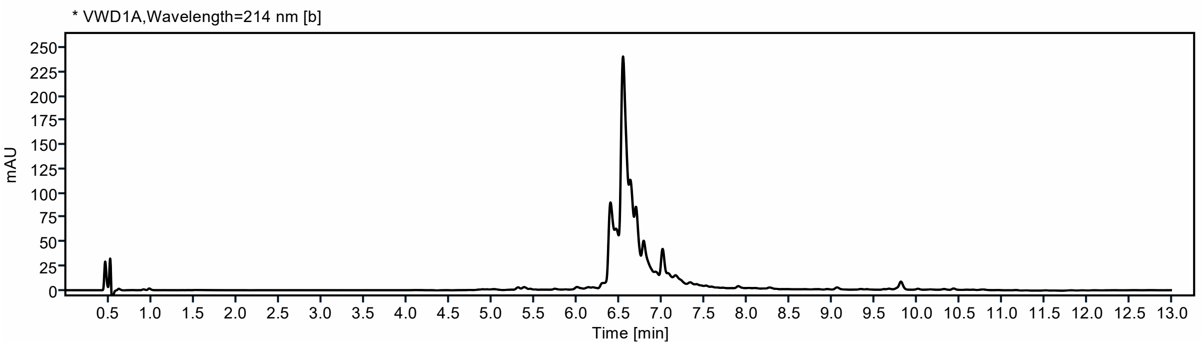

SI Figure 114. UHPLC profile of Barstar[75–90] bearing $(Arg)_6$ at the N-terminus, synthesized at room temperature (23 °C) by batch SPPS. Rt 6.70 min (Agilent Zorbax 300SB-C18 column, 5 µm, 2.1 × 150 mm, 5–95% MeCN over 10 min, ca. 9%B/min), 33% purity based on Area Under Curve (AUC) at λ = 214 nm.

3.10 Effect of $[Arg(Pbf)]_6$ Tag position and temperature: hGH[176–191](F176Y)

3.10.1 Batch synthesis at room temperature (23 °C) – no tag

```
                10         16
$H_2N$-YLRIVQCRSV EGSCGF -$CONH_2$     (16 AA)
```

The peptide hGH[176–191](F176Y) was synthesized on commercially available Novabiochem® NovaPEG Rink Amide resin (0.41 mmol/g, 104 mg, 49 µmol) using the batch SPPS standard protocol (**Section 2.2.4**) at room temperature. Cleavage of the peptidyl-resin (16 mg, approx. 3.1 µmol) according to Cleavage Protocol A (**Section 2.5.1**) afforded the crude peptide (3.7 mg, 35% purity by LCMS [**SI Figure 115**], 16% purity by UHPLC [**SI Figure 116**]).

LC-MS of crude hGH[176–191](F176Y)

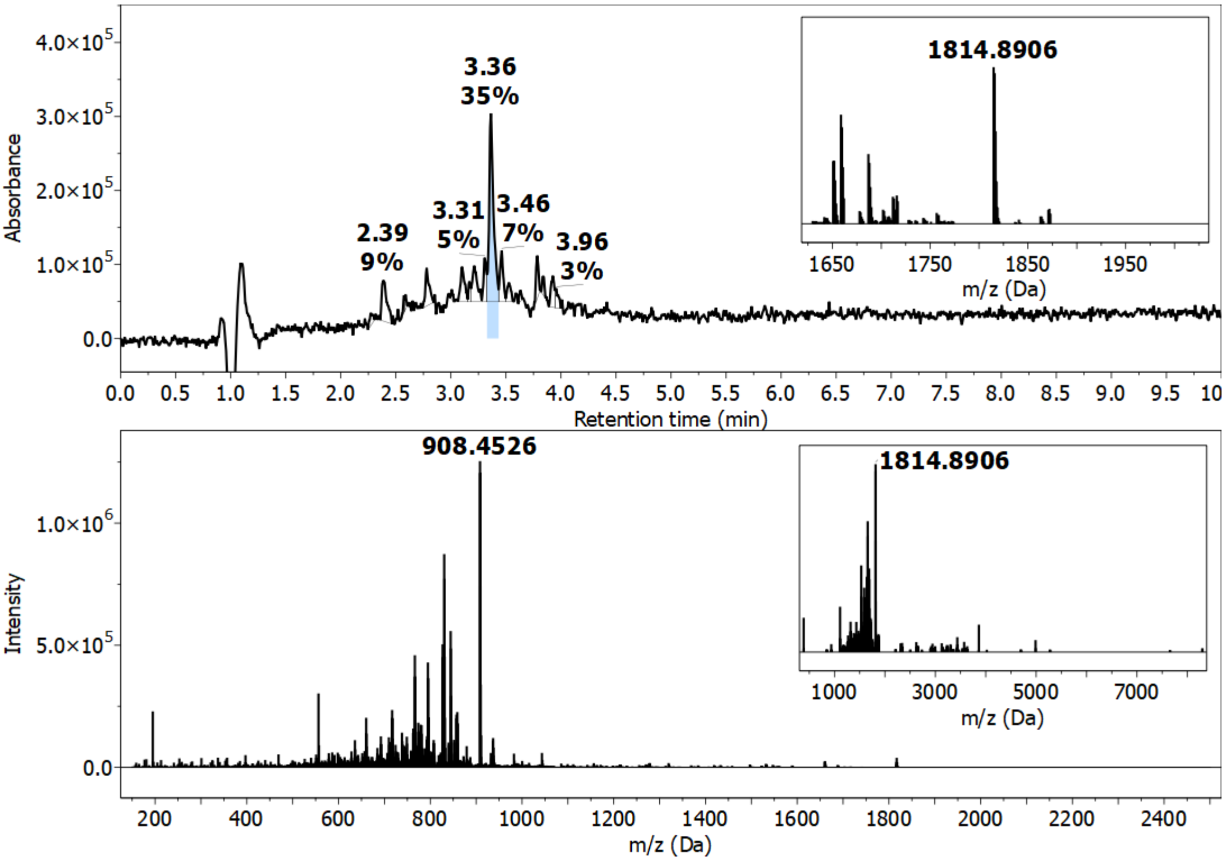

SI Figure 115. LCMS Profile of crude hGH[176–191](F176Y). (**a**) Absorbance chromatogram (λ = 214 nm); Rt 3.36 min, 35% purity. (**b**) ESI-TOF spectrum found within Rt 2–9 min (insert: deconvoluted masses); Monoisotopic mass (ESI+) calcd. for $C_{78}H_{126}N_{24}O_{22}S_{28}$ 1814.8920, found 1814.8906. LCMS Gradient A (**Section 2.7**).

UHPLC of crude hGH[176–191]

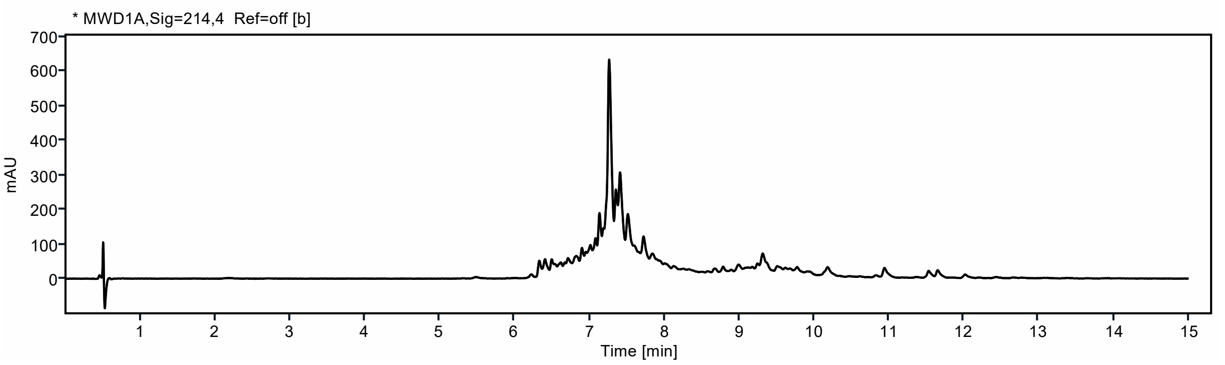

SI Figure 116. UHPLC profile of crude hGH[176–191](F176Y). Rt 7.63 min (Agilent Zorbax 300SB-C18 column, 5 µm, 2.1 × 150 mm, 5–95% MeCN over 10 min, ca. 9%B/min), 16% purity based on Area Under Curve (AUC) at λ = 214 nm.

3.10.2 hGH[176–191](F176Y): Batch synthesis at room temperature (23 °C) – [Arg(Pbf)$_6$]-tag at C-terminus

```
          10                    20    22
H2N-YLRIVQCRSV EGSCGFRRRR RR-CONH2    (22 AA)
```

The peptide hGH[176–191](F176Y)-[Arg(Pbf)]$_6$ was synthesized on commercially available Novabiochem® NovaPEG Rink Amide resin (0.41 mmol/g, 100 mg, 4.1 µmol) using the batch

SPPS standard protocol (**Section 2.2.4**) at room temperature. Cleavage of the peptidyl-resin (12 mg, approx. 2.0 μmol) according to Cleavage Protocol A (**Section 2.5.1**) afforded the crude peptide (3.5 mg, 57% purity by LCMS [**SI Figure 117**], 32% purity by UHPLC [**SI Figure 118**]).

LC-MS of crude hGH[176–191](F176Y)-(Arg)$_6$

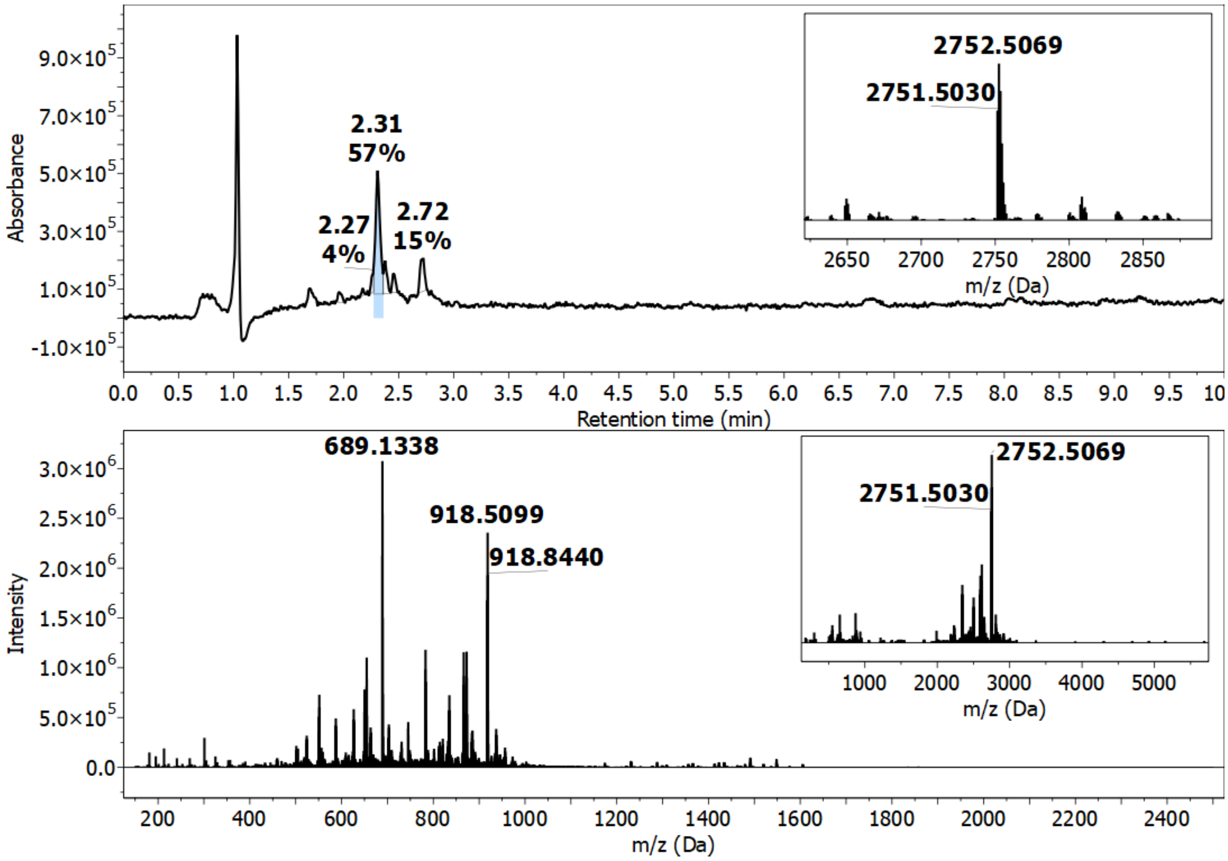

SI Figure 117. LCMS Profile of crude hGH[176–191](F176Y) bearing (Arg)$_6$ at the C-terminus. (**a**) Absorbance chromatogram (λ = 214 nm); Rt 2.31 min, 57% purity. (**b**) ESI-TOF spectrum found within Rt 2–9 min (insert: deconvoluted masses); Monoisotopic mass (ESI+) calcd. for $C_{114}H_{198}N_{48}O_{28}S_2$ 2751.4987, found 2751.5030. LCMS Gradient A (**Section 2.7**).

UHPLC of crude hGH[176–191](F176Y)-(Arg)$_6$

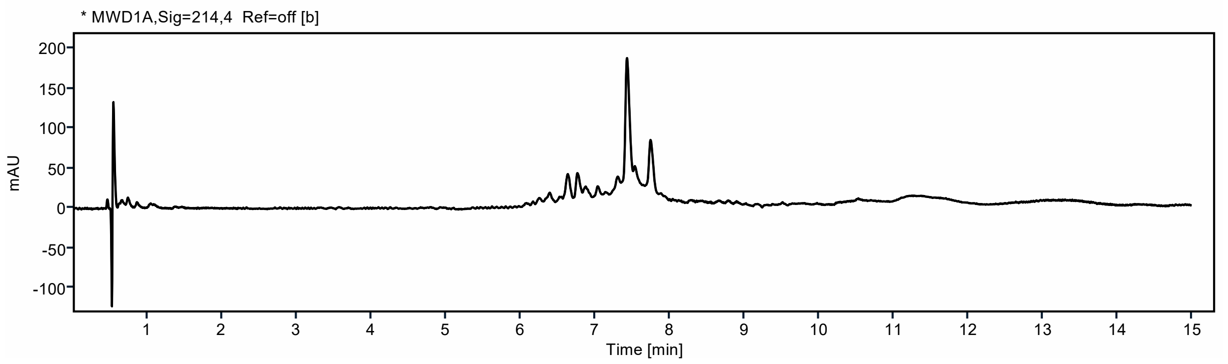

SI Figure 118. UHPLC profile of crude hGH[176–191](F176Y) bearing (Arg)$_6$ at the C-terminus. Rt 7.42 min (Agilent Zorbax 300SB-C18 column, 5 μm, 2.1 × 150 mm, 5–95% MeCN over 10 min, ca. 9%B/min), 32% purity based on Area Under Curve (AUC) at λ = 214 nm

3.10.3 hGH[176–191](F176Y): Batch synthesis at room temperature (23 °C) – [Arg(Pbf)$_6$]-tag at N-terminus

```
                   10                  20   22
H2N-RRRRRRYLRI VQCRSVEGSCGF GF-CONH2        (22 AA)
```

Upon resin-bound hGH[176–191](F176Y) (97.3 mg, approx. 20 µmol) prepared by batch SPPS (see **Section 3.10.1**), [Arg(Pbf)]$_6$ was added to the N-terminus using the batch SPPS standard protocol (**Section 2.2.4**) at 23 °C. Cleavage of the peptidyl-resin (25.5 mg, approx. 4.0 µmol) according to Cleavage Protocol A (**Section 2.5.1**) afforded the crude peptide (5.8 mg, 32% purity by LCMS [**SI Figure 119**], 13% purity by UHPLC [**SI Figure 120**]).

LC-MS of crude (Arg)$_6$-hGH[176–191](F176Y)

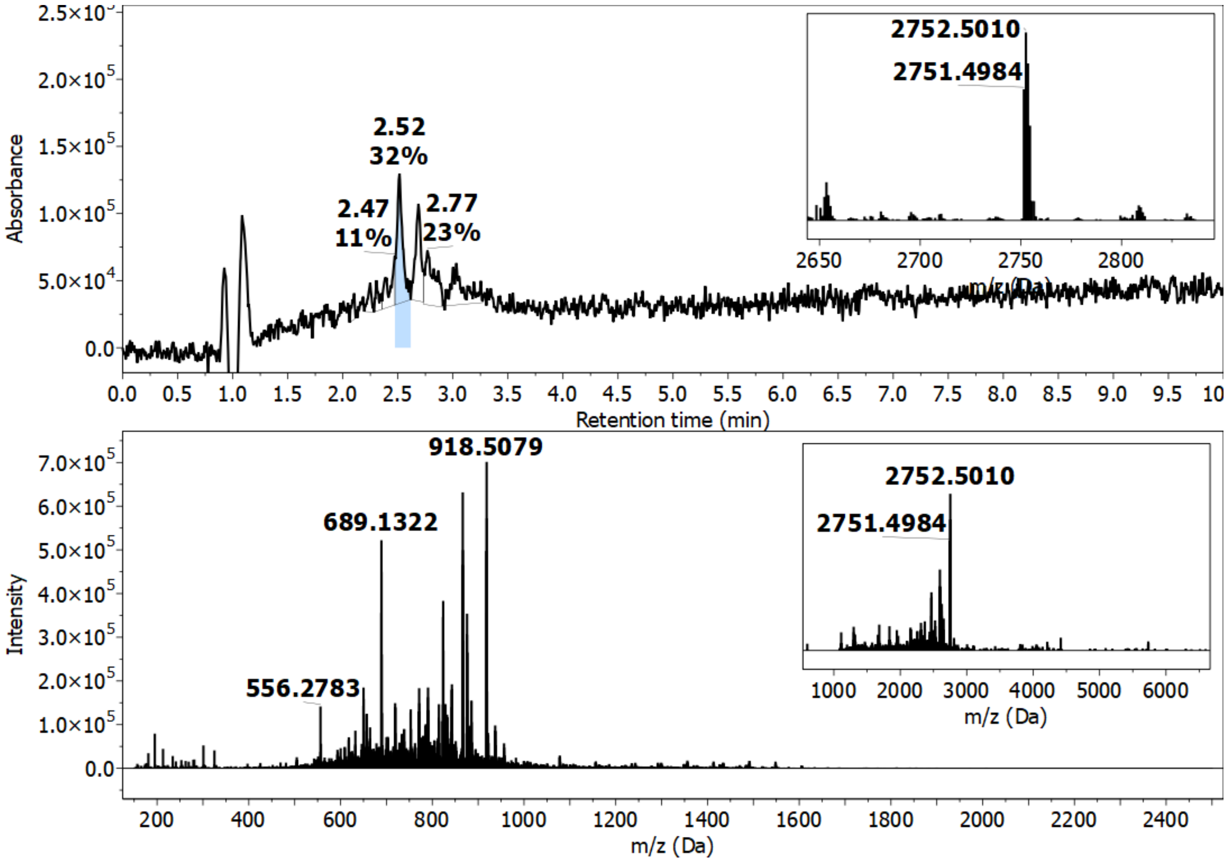

SI Figure 119. LCMS Profile of crude hGH[176–191](F176Y) bearing (Arg)$_6$ at the N-terminus. (**a**) Absorbance chromatogram (λ = 214 nm); Rt 2.52min, 32% purity. (**b**) ESI-TOF spectrum found within Rt 2–9 min (insert: deconvoluted masses); Monoisotopic mass (ESI+) calcd. for $C_{114}H_{198}N_{48}O_{28}S_2$ 2751.4987, found 2751.4984. LCMS Gradient A (**Section 2.7**).

UHPLC of crude $(Arg)_6$-hGH[176–191](F176Y)

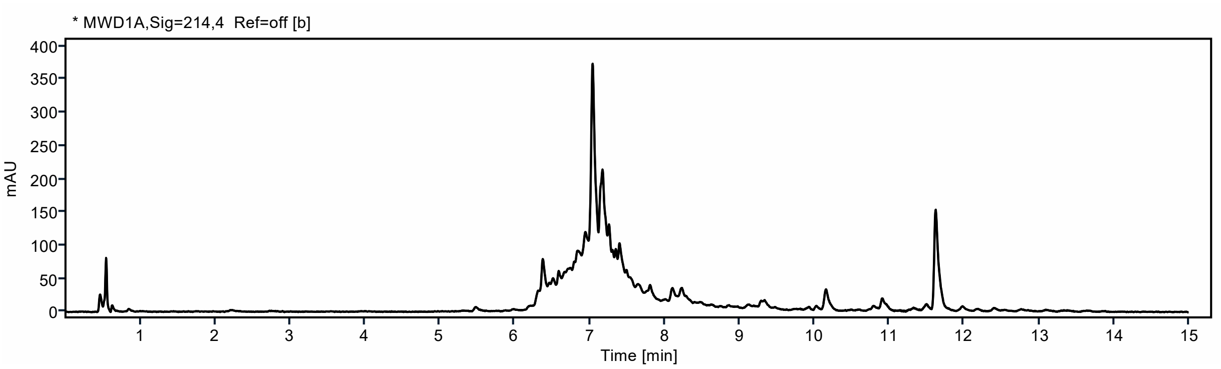

SI Figure 120. UHPLC profile of crude hGH[176–191](F176Y) bearing $(Arg)_6$ on the N-terminus. Rt 7.40 min (Agilent Zorbax 300SB-C18 column, 5 µm, 2.1 × 150 mm, 5–95% MeCN over 10 min, ca. 9%B/min), 13% purity based on Area Under Curve (AUC) at λ = 214 nm

3.10.4 Cumulative HPLC traves of hGH[176–191](F176Y)

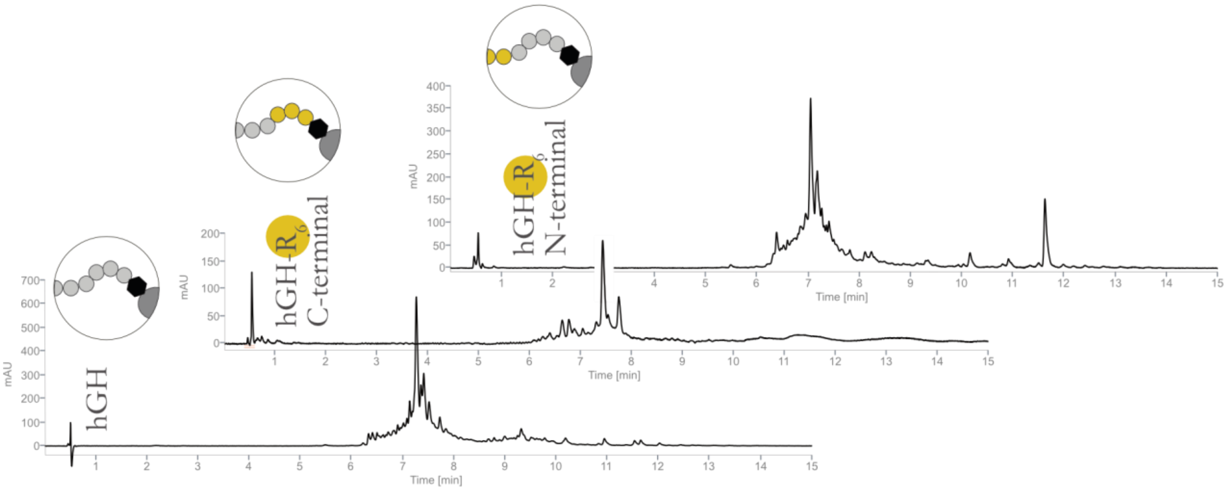

SI Figure 121. Comparison of the crude purities of hGH[176–191](F176Y) synthesized at room temperature in batch-SPPS depending on the position of the $[Arg(Pbf)]_6$ tag.

3.11 Effect of Arg(Pbf) tag length and position: GLP-1

3.11.1 GLP-1: $[Arg(Pbf)]_6$-Tag, N-terminus

```
                  10          20          30
H2N-RRRRRRHAEG  TFTSDVSSYL  EGQAAKEFIA WLVKGR-CONH2     (36 AA)
```

The peptide $[Arg(Pbf)]_6$-GLP-1 was synthesized on commercially available Novabiochem® NovaPEG Rink Amide resin (0.41 mmol/g, 50 mg, 21 µmol) using the standard AFPS protocol (**Section 2.2.1**, 40 mL/min flowrate) (**SI Figure 122**). Total synthesis time to afford resin-bound $[Arg(Pbf)]_6$-GLP-1 was approximately 2 h. Cleavage of the peptidyl-resin (54 mg, approx. 22 µmol), according to Cleavage Protocol A (**Section 2.5.1**) afforded the crude peptide (18 mg, 67% purity by LCMS [**SI Figure 123**], 65% purity by UHPLC [**SI Figure 124**]).

UV-Vis synthesis trace

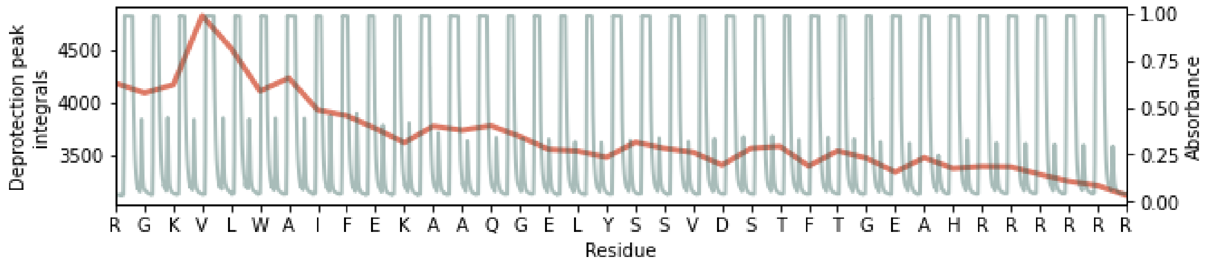

SI Figure 122. UV trace (λ = 310 nm) from AFPS of GLP-1 bearing the [Arg(Pbf)]$_6$ tag on the N-terminus (green) and deprotection peak integrals (red).

LC-MS of crude (Arg)$_6$-GLP-1

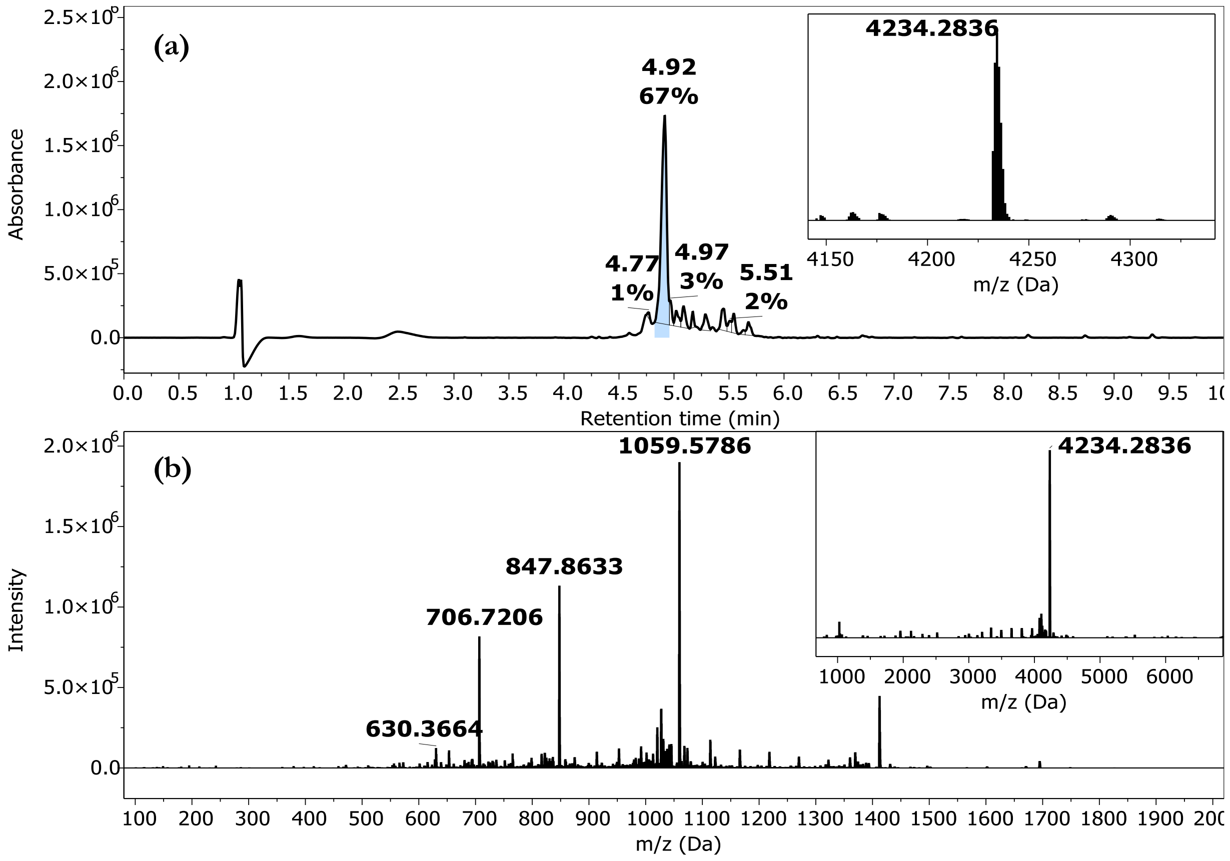

SI Figure 123. LC-MS Profile of crude GLP-1 bearing (Arg)$_6$ at the N-terminus. (**a**) Absorbance chromatogram (λ = 214 nm) of [Arg]$_6$-GLP-1; Rt 4.92 min, 67% purity. (**b**) ESI-TOF spectrum found within Rt 2–9 min (insert: deconvoluted masses); Monoisotopic mass (ESI+) calcd. for $C_{185}H_{298}N_{64}O_{51}$ 4232.2692, found 4232.2790. LCMS Gradient A (**Section 2.7**).

UHPLC of crude (Arg)$_6$-GLP-1

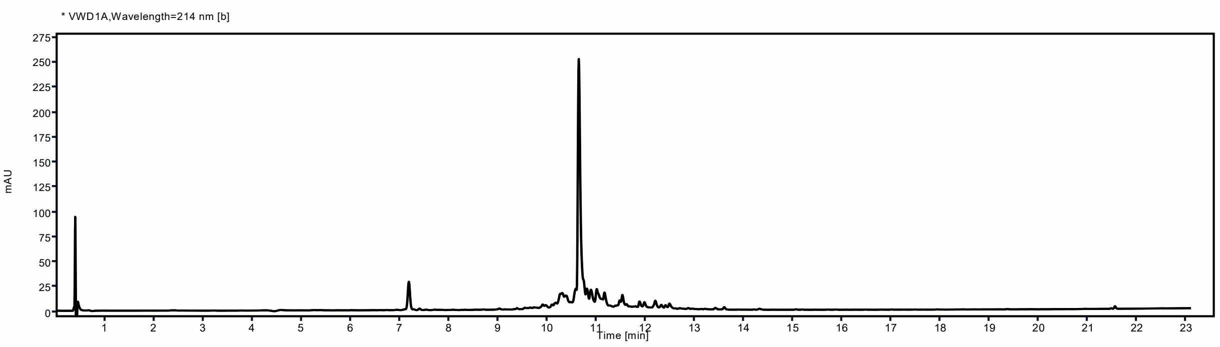

SI Figure 124. UHPLC profile of crude GLP-1 bearing (Arg)$_6$ at the N-terminus. Rt 10.63 min (Agilent Zorbax 300SB-C18 column, 5 µm, 2.1 × 150 mm, 5–95% MeCN over 20 min, ca. 4.5%B/min), 65% purity based on Area Under Curve (AUC) at λ = 214 nm.

3.11.2 GLP-1: [Arg(Pbf)]$_3$-Tag, C-terminus

```
                 10          20          30
H2N-HAEGTFTSDV  SSYLEGQAAK  EFIAWLVKGR RRR-CONH2      (33 AA)
```

The peptide GLP-1-[Arg(Pbf)]$_3$ was synthesized on commercially available Novabiochem® NovaPEG Rink Amide resin (0.41 mmol/g, 53 mg, 22 µmol) using the standard AFPS protocol (**Section 2.2.1**, 40 mL/min flowrate) (**SI Figure 125**). Total synthesis time to afford resin-bound GLP-1-[Arg(Pbf)]$_3$ was approximately 2 h. Cleavage of the peptidyl-resin (56 mg, approx. 23 µmol), according to Cleavage Protocol A (**Section 2.5.1**) afforded the crude peptide (17 mg, 62% purity by LCMS [**SI Figure 126**], 60% purity by UHPLC [**SI Figure 127**]).

UV-Vis synthesis trace

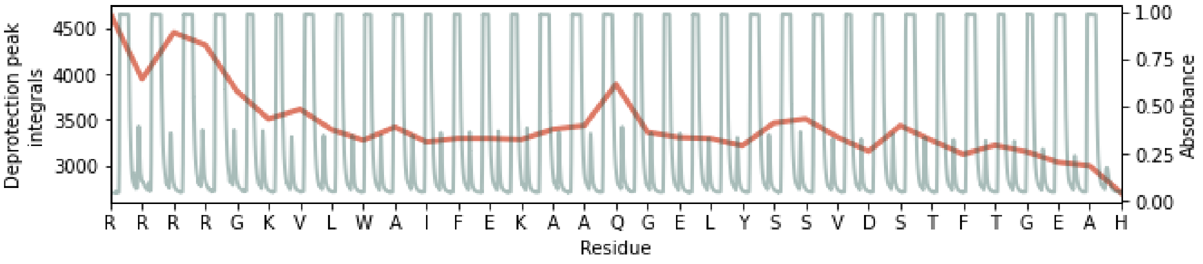

SI Figure 125. UV trace (λ = 310 nm) of GLP-1 bearing the [Arg(Pbf)]$_3$ tag on the C-terminus (green) and deprotection peak integrals (red).

LC-MS of crude GLP-1-(Arg)₃

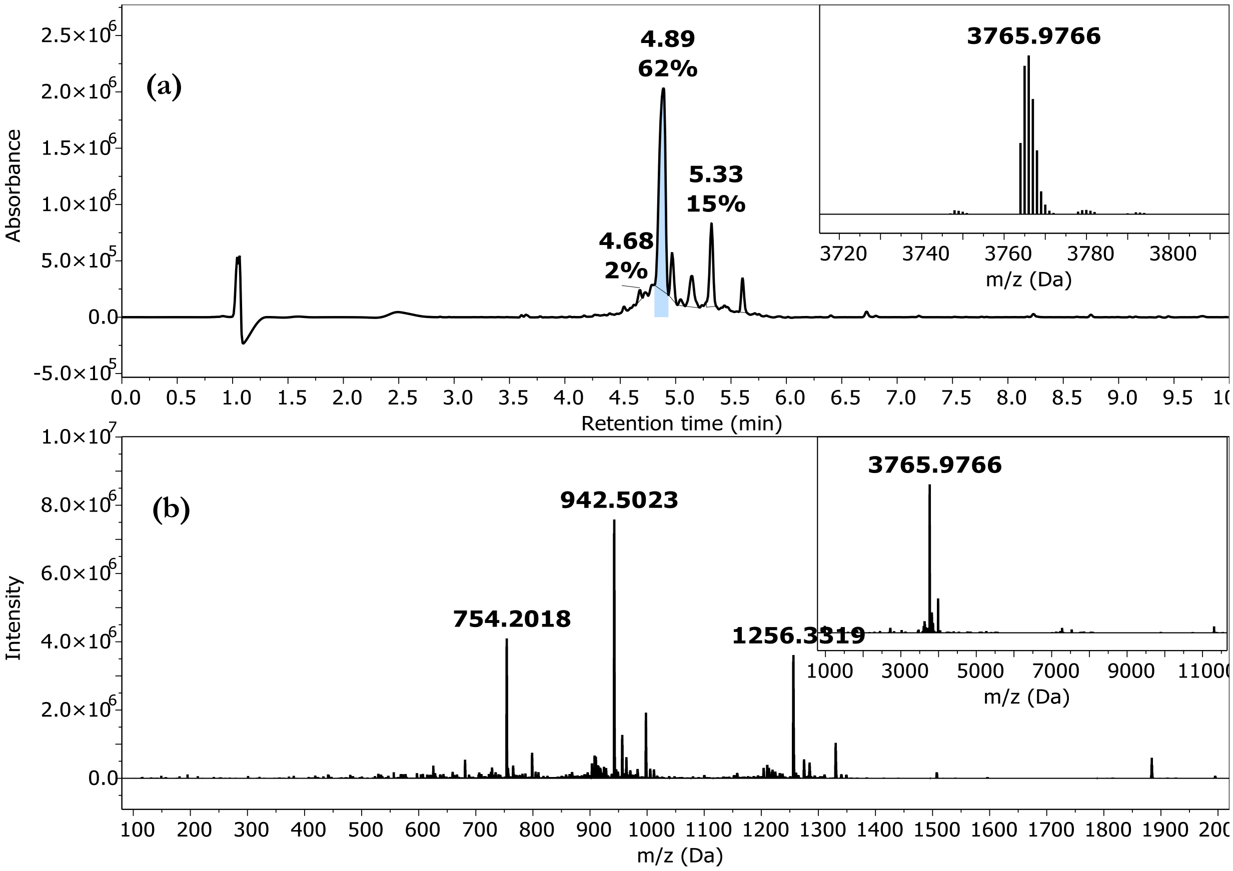

SI Figure 126. LC-MS Profile of crude GLP-1-[Arg]$_3$. (**a**) Absorbance chromatogram (λ = 214 nm) of peptide GLP-1-[Arg]$_3$; Rt 4.89 min, 62% purity. (**b**) ESI-TOF spectrum found within Rt 2–9 min (insert: deconvoluted masses); Monoisotopic mass (ESI+) calcd. for $C_{167}H_{262}N_{52}O_{48}$ 3763.9659, found 3763.9713. LCMS Gradient A (**Section 2.7**).

UHPLC of crude GLP-1-(Arg)₃

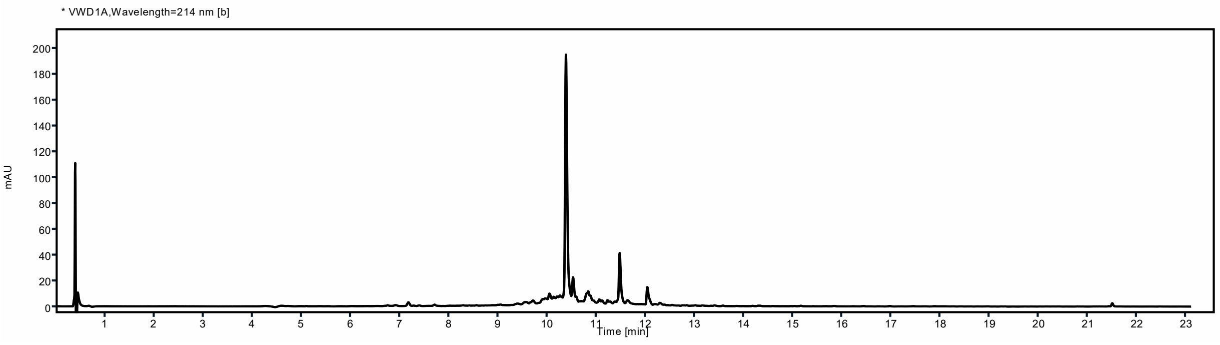

SI Figure 127. UHPLC profile of crude GLP-1-[Arg]$_3$. Rt 10.37 min (Agilent Zorbax 300SB-C18 column, 5 µm, 2.1 × 150 mm, 5–95% MeCN over 23 min, ca. 4.5%B/min), 60% purity based on Area Under Curve (AUC) at λ = 214 nm

3.11.3 GLP-1: [Arg(Pbf)]₉-Tag, C-terminus

```
                  10                 20                  30
H2N-HAEGTFTSDV   SSYLEGQAAK   EFIAWLVKGR RRRRRRRRR-CONH2    (39 AA)
```

The peptide GLP-1-[Arg(Pbf)]$_9$ was synthesized on commercially available Novabiochem® NovaPEG Rink Amide resin (0.41 mmol/g, 49 mg, 20 µmol) using the standard AFPS protocol (**Section 2.2.1**, 40 mL/min flowrate) (**SI Figure 128**). Total synthesis time to afford resin-bound

GLP-1-[Arg(Pbf)]₉ was approximately 2 h. Cleavage of the peptidyl-resin (44 mg, approx. 18 µmol), according to Cleavage Protocol A (**Section 2.5.1**) afforded the crude peptide (6.7 mg, 85% purity by LCMS [**SI Figure 129**], 66% purity by UHPLC [**SI Figure 130**]).

UV-Vis synthesis trace

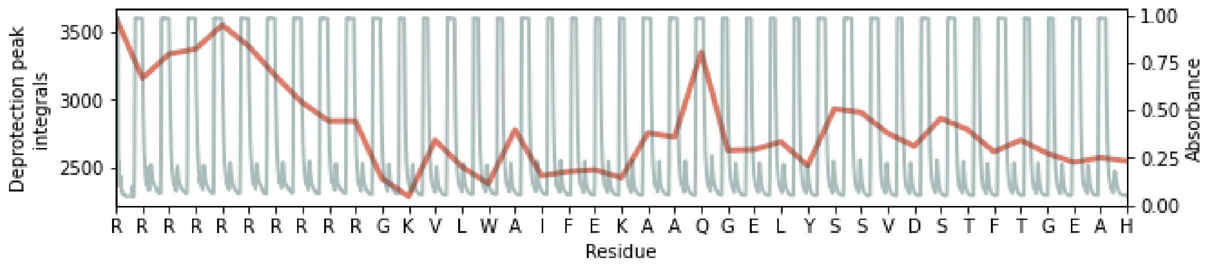

SI Figure 128. UV trace (λ = 310 nm) from AFPS of GLP-1 bearing the [Arg(Pbf)]$_9$ tag on the C-terminus (green) and deprotection peak integrals (red).

LC-MS of crude GLP-1-(Arg)$_9$

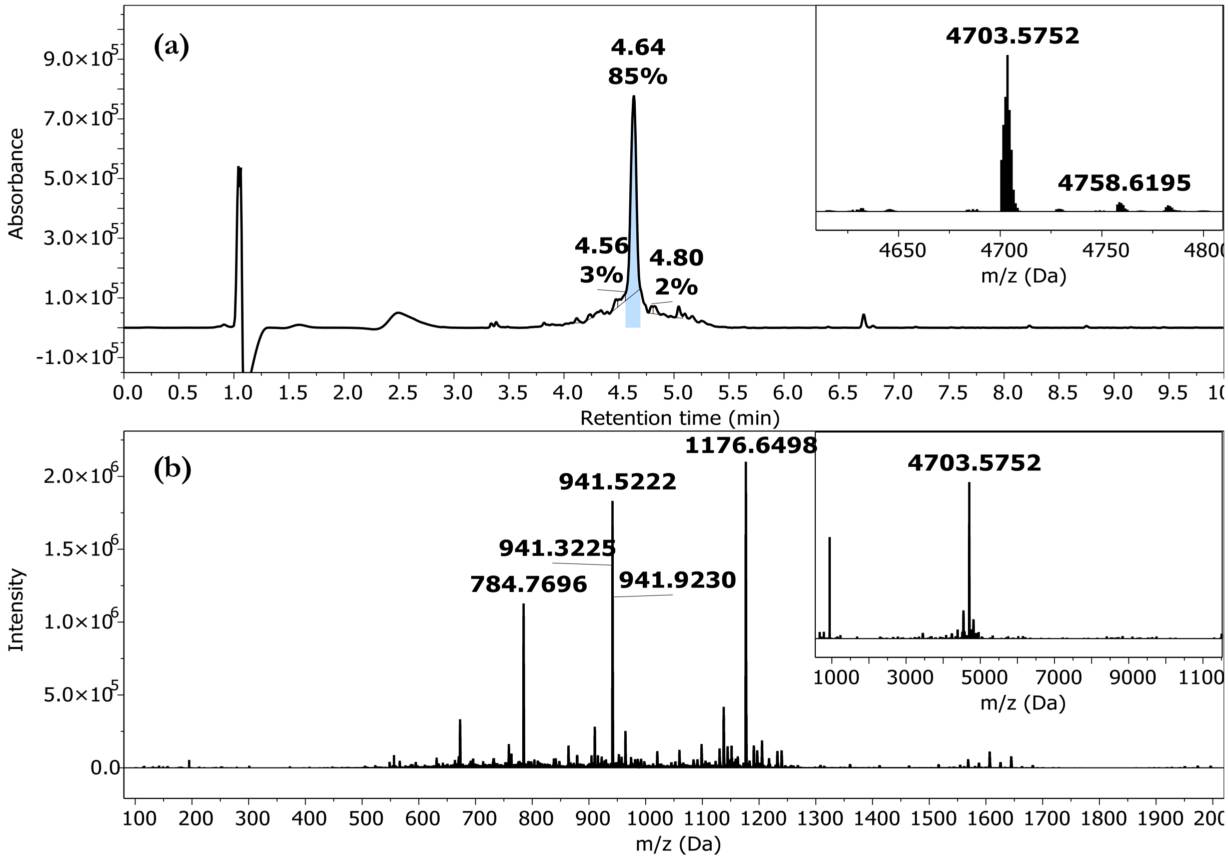

SI Figure 129. LC-MS Profile of crude peptide GLP-1-(Arg)$_9$. (**a**) Absorbance chromatogram (λ = 214 nm) of peptide GLP-1-(Arg)$_6$; Rt 4.64 min, 85% purity. (**b**) ESI-TOF spectrum found within Rt 2–9 min (insert: deconvoluted masses); Monoisotopic mass (ESI+) calcd. for $C_{203}H_{334}N_{76}O_{54}$ 4700.5726, found 4700.5701. LCMS Gradient A (**Section 2.7**).

UHPLC of crude GLP-1-(Arg)$_9$

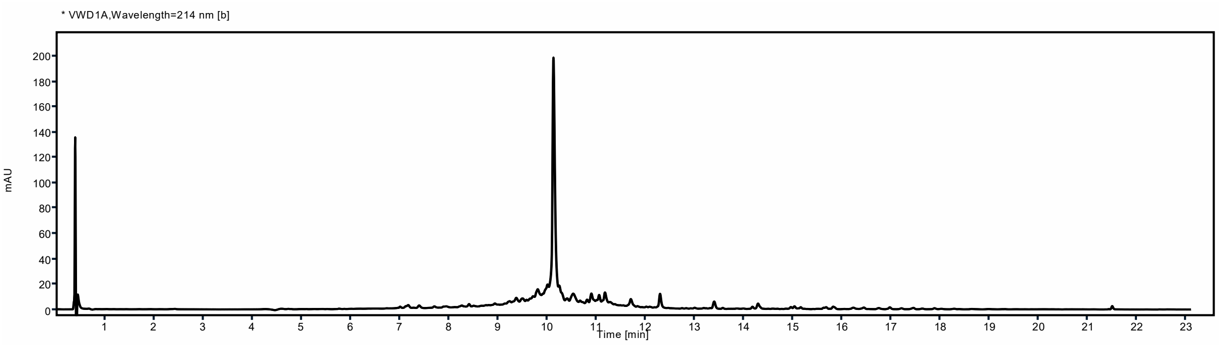

SI Figure 130. UHPLC profile of crude GLP-1 bearing (Arg)$_9$ on the C-terminus. Rt 10.12 min (Agilent Zorbax 300SB-C18 column, 5 µm, 2.1 × 150 mm, 5–95% MeCN over 23 min, ca. 4.5%B/min), 66% purity based on Area Under Curve (AUC) at λ = 214 nm.

3.12 Recapitulative table of crude purities for amino acid tags

	No Tag	(Arg)$_6$
**GLP-1**	73%	84%
**hGH**	32%	73%
**JR-10**	28%	77%
**MYC[123–143]**	27%	76%
**MYC[86–143]**	-	27%
**Aβ42[27–42]**	33%	48%
**α-synuclein[66–82]**	38%	30%

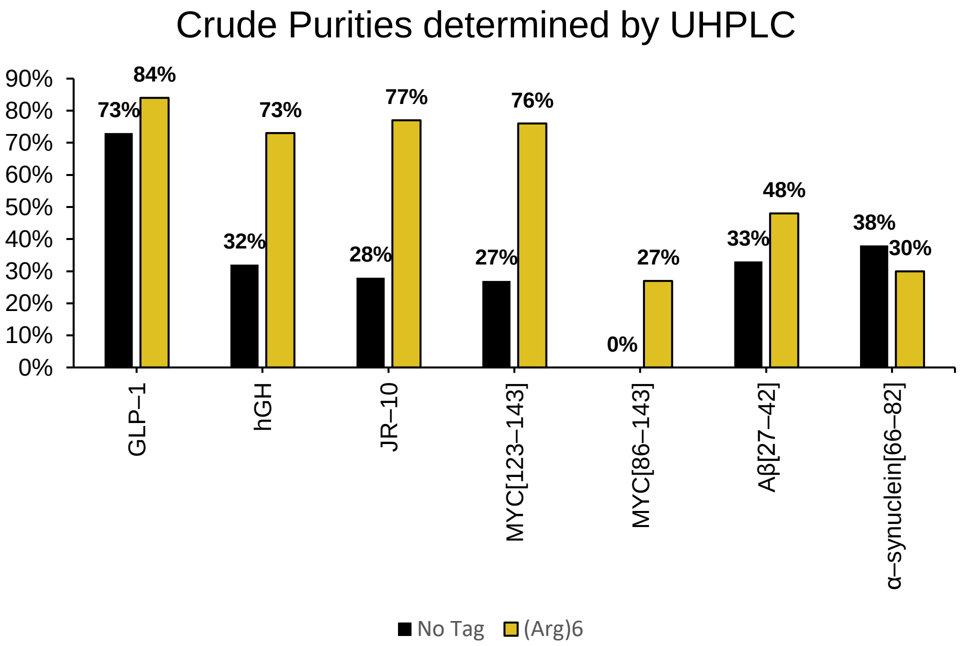

SI Figure 131. Crude purities determined by UHPLC of GLP-1, hGH, JR-10, MYC[123–143], MYC[86–143], Aβ[27–42] and α-synuclein[66–82] with (yellow) and without (black) (Arg)$_6$.

	No Tag	$(Arg)_6$
**Barstar[75–90] 90 °C**	63%	70%
**Barstar[75–90] 70 °C**	38%	61%
**Barstar[75–90] Batch SPPS**	55%	69%
**Barstar[75–90] Low Loading Resin**	77%	80%

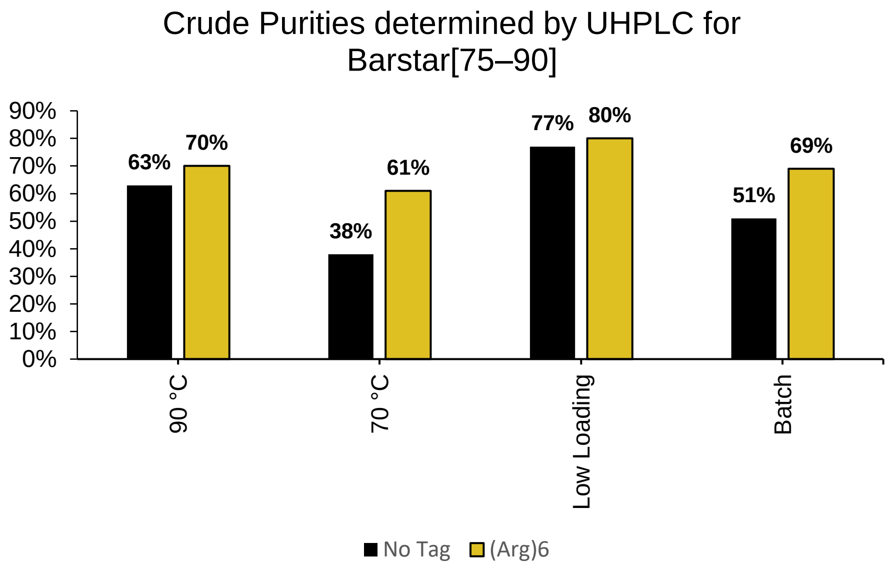

SI Figure 132. Crude purities determined by UHPLC of Barstar[75–90] with (yellow) and without (black) $(Arg)_6$, synthesized on the AFPS on high loading resin (0.41 mmol/g) at 90°C and 70°C, and at 90°C on low loading (0.20 mmol/g), and in batch SPPS at 23 °C.

	GLP-1	GLP-1 -$(Arg)_6$	$(Arg)_6$-GLP-1	GLP-1-$(Arg)_3$	GLP-1-$(Arg)_9$
**Crude Purity**	73%	84%	65%	60%	66%

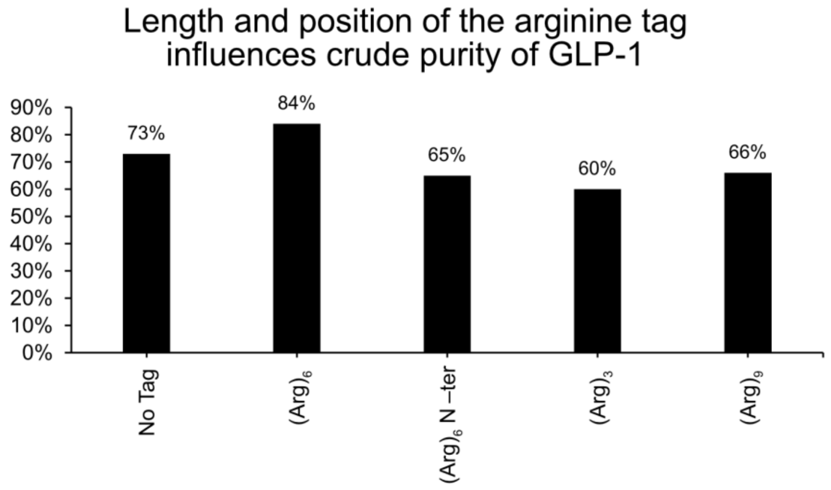

SI Figure 133. The length and position of the Arginine tags impact the overall crude purities of GLP-1. Crude purities were determined by UHPLC for GLP-1, GLP-1-$(Arg)_6$, $(Arg)_6$-GLP-1, GLP-1-$(Arg)_3$, and GLP-1-$(Arg)_9$ that were synthesized by AFPS on high loading resin (0.41 mmol/g) at 90 °C.

3.13 Impact of the protecting groups

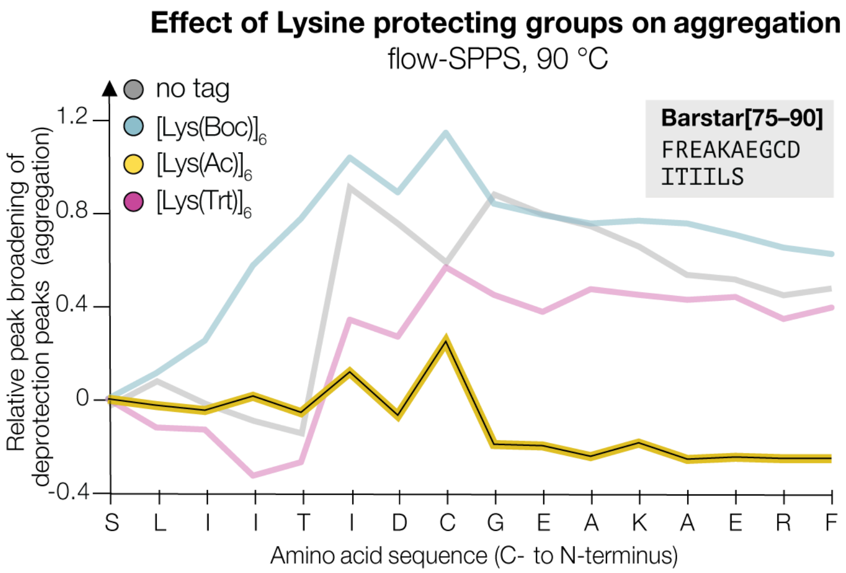

SI Figure 134. Side-chain protecting groups of Lys residues affected aggregation of Barstar[75–90], with [Lys(Boc)]$_6$ impacting both the onset and severity. Aggregation is plotted as a function of Fmoc-deprotection peak broadening by in-line UV-Vis (310 nm) in flow-SPPS for hGH[176–191](F176Y) with various amino acid tag, normalized at Ser[90].

3.13.1 Barstar[75–90]: [Lys(Ac)]$_6$-Tag

```
          10          20
H2N-FREAKAEGCD ITIILSKKKK KK-CONH2        (22 AA)
```

The six tag amino acids were coupled manually as follows: pre-functionalized NovaPEG Rink Amide resin (0.41 mmol/g loading, 103.4 mg, 42 µmol, 1.0 eq.) was swelled with DCM (1 × 5 mL) for 1 min, drained, and washed with DMF (1 × 5 mL). For each coupling, a solution of Fmoc-Lys(Ac)-OH (513 µL, 0.40 M in DMF, 5.0 eq.) and HATU (505 µL, 0.38 M in DMF, 4.8 eq.) was prepared. To this solution, DIPEA (36 µL, 0.20 mmol, 5 eq.) was added, and the solution was gently agitated at 23 °C for 1 min. The solution was then added to the resin, and the reaction was gently stirred for 20 s, then left at 23 °C for 45 min. The resin was then drained, washed with DMF (3 × 5 mL) and DCM (3 × 5 mL). For each deprotection step, 20% piperidine in DMF (*v*/*v*) (3.0 mL) was added to the resin, and the reaction was gently stirred for 20 s then left at 23 °C for 10 min. The resin was then drained, then washed with DMF (3 × 5 mL) and DCM (3 × 5 mL).

The peptide Barstar[75–90]-[Lys(Ac)]$_6$ tag was synthesized on the resulting resin using the standard AFPS protocol (**Section 2.2.1**, 20 mL/min flowrate) (**SI Figure 135**). Total synthesis time to afford resin-bound Barstar[75–90]-[Lys(Ac)]$_6$ was approximately 0.75 h. Cleavage of the peptidyl-resin (12.0 mg, 4.9 µmol) according to Cleavage Protocol A (**Section 2.5.1**) afforded the crude peptide (3.0 mg, 43% purity by LCMS [**SI Figure 136**], 46% purity by UHPLC [**SI Figure 137**]).

UV-Vis synthesis trace

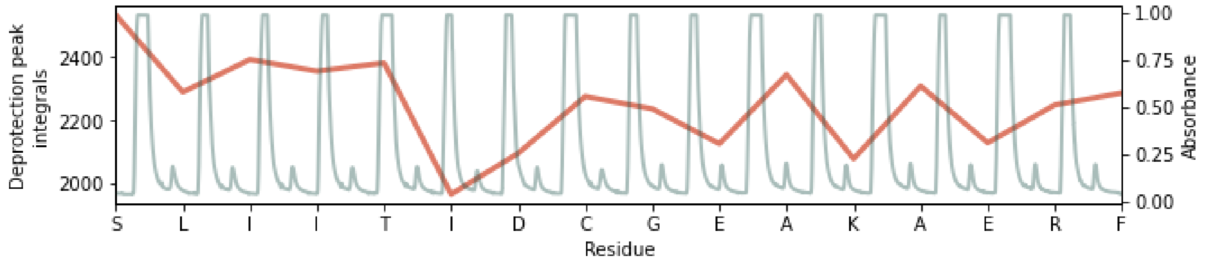

SI Figure 135. UV trace (λ = 310 nm) from AFPS of Barstar[75–90]-[Lys(Ac)]$_6$ (green) and deprotection peak integrals (red).

LC-MS of crude Barstar[75–90]-[Lys(Ac)]$_6$

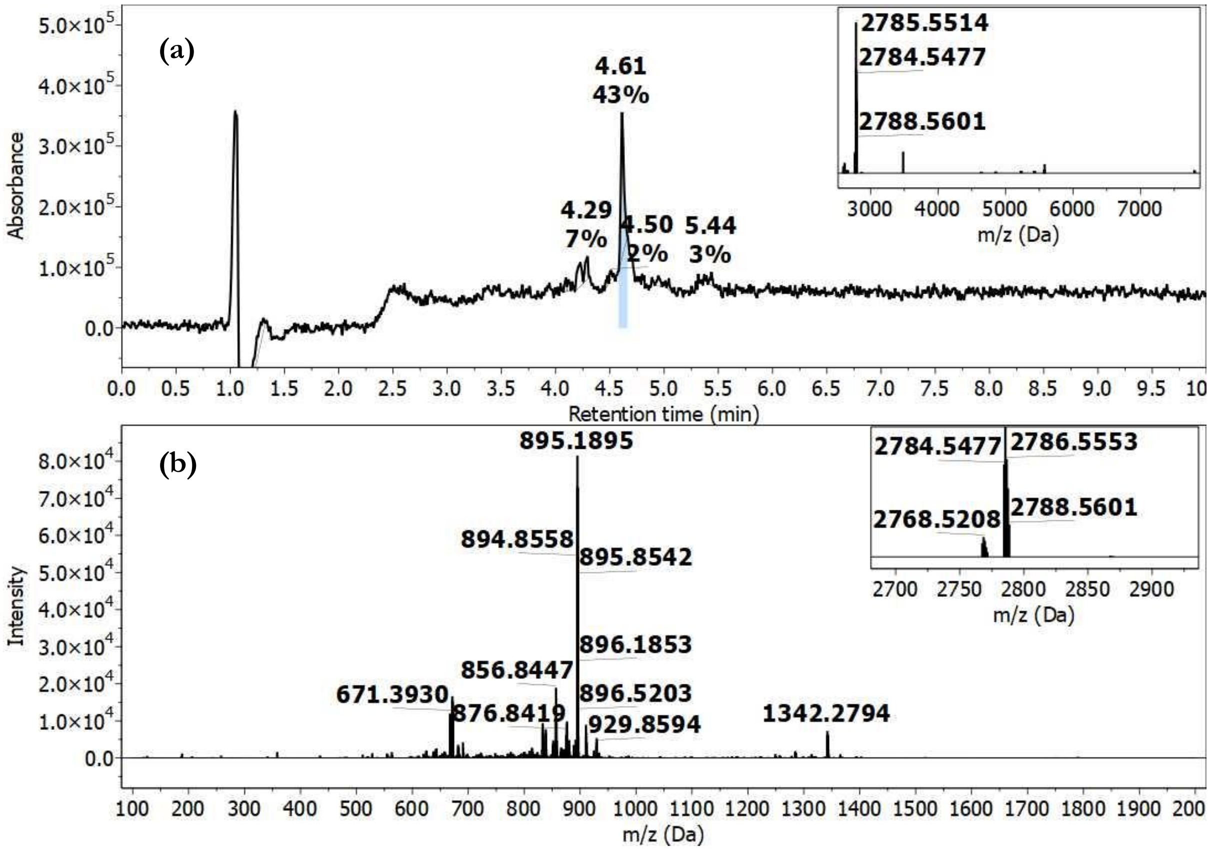

SI Figure 136. LCMS Profile of crude Barstar[75–90] bearing [Lys(Ac)]$_6$. (**a**) Absorbance chromatogram (λ = 214 nm) of Barstar[75–90]-(Lys)$_6$ tag; Rt 4.61 min, 43% purity. (**b**) ESI-TOF spectrum found within Rt 2–9 min (insert: deconvoluted masses). Monoisotopic mass (ESI+) calcd. for $C_{125}H_{213}N_{33}O_{36}S$ 2784.5572, found 2784.5477. LCMS Gradient B (**Section 2.7**).

UHPLC of crude Barstar[75–90]-[Lys(Ac)]$_6$

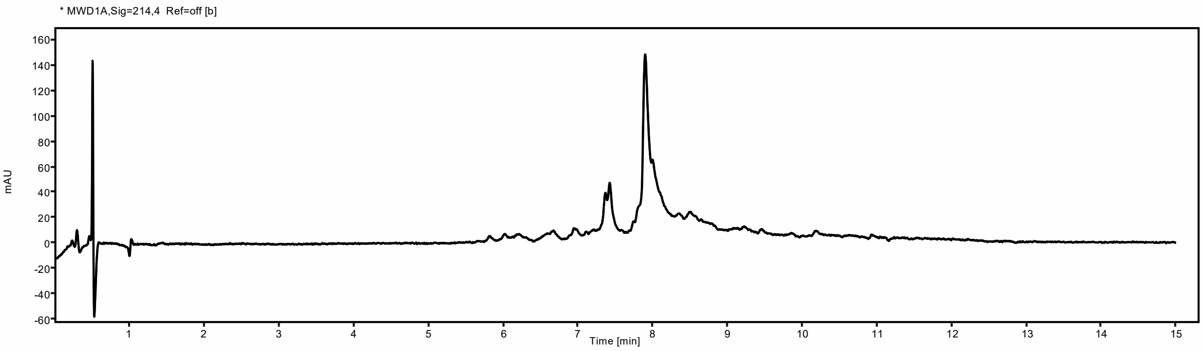

SI Figure 137. UHPLC profile of crude Barstar[75–90]-[Lys(Ac)]$_6$. Rt 7.90 min (Agilent Zorbax 300SB-C18 column, 5 µm, 2.1 × 150 mm, 5–95% MeCN over 10 min, ca. 9%B/min), 46% purity based on Area Under Curve (AUC) at λ = 214 nm.

3.13.2 Barstar[75–90]: [Lys(Boc)]$_6$-Tag

10 20
H_2N-FREAKAEGCD ITIILS**KKKK KK**-CONH_2 (22 AA)

The peptide Barstar[75–90]-[Lys(Boc)]$_6$ tag was synthesized on commercially available Novabiochem® NovaPEG Rink Amide resin (0.41 mmol/g, 50.7 mg, 21 µmol) using the standard AFPS protocol (**Section 2.2.1**, 20 mL/min flowrate) (**SI Figure 138**). Total synthesis time to afford resin-bound Barstar[75–90]-[Lys(Boc)]$_6$ was approximately 1 h. Cleavage of the peptidyl-resin (18.9 mg, 7.7 µmol) according to Cleavage Protocol A (**Section 2.5.1**) afforded the crude peptide (4.0 mg, 33% purity by LCMS **SI Figure 139**], 33% purity by UHPLC **SI Figure 140**]).

UV-Vis synthesis trace

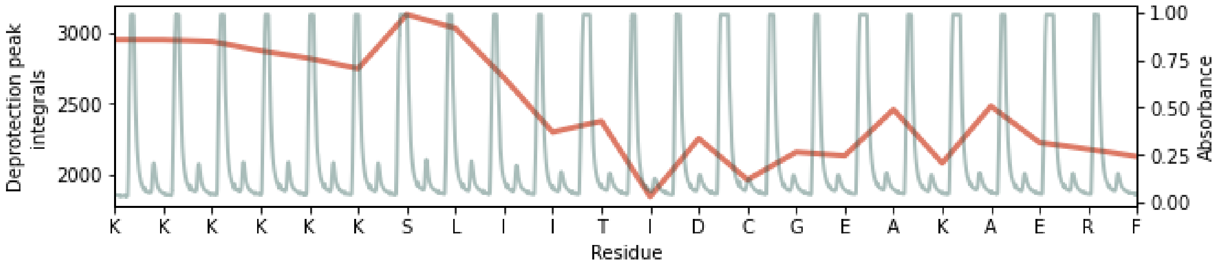

SI Figure 138. UV trace (λ = 310 nm) from AFPS of Barstar[75–90]-[Lys(Boc)]$_6$ (green) and deprotection peak integrals (red).

LC-MS of crude Barstar[75–90]-(Lys)$_6$

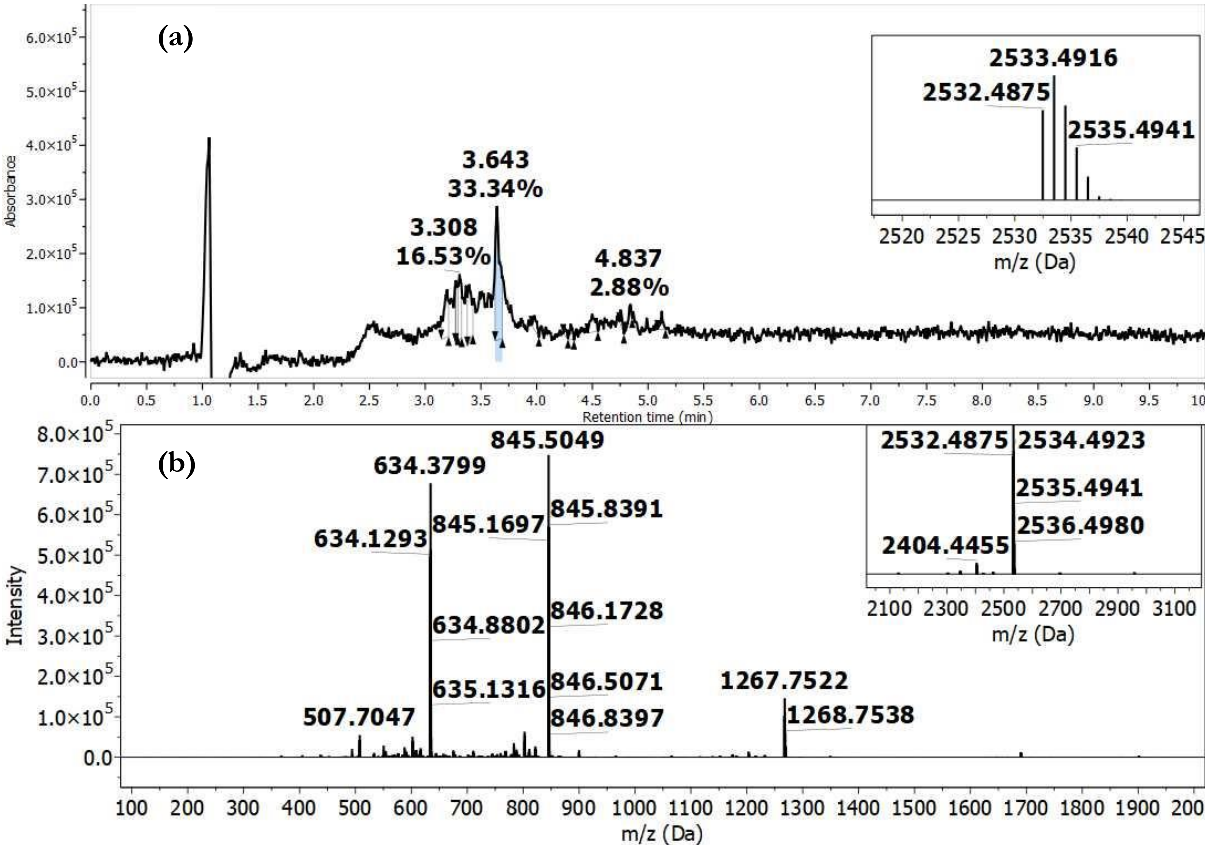

SI Figure 139. LCMS Profile of crude Barstar[75–90] bearing (Lys)$_6$. (**a**) Absorbance chromatogram (λ = 214 nm) of Barstar[75–90]-(Lys)$_6$ tag; Rt 3.64 min, 33% purity. (**b**) ESI-TOF spectrum found within Rt 2–9 min (insert: deconvoluted masses). Monoisotopic mass (ESI+) calcd. for $C_{113}H_{201}N_{33}O_{30}S$ 2532.4938, found 2532.4875. LCMS Gradient B (**Section 2.7**).

UHPLC of crude Barstar[75–90]-(Lys)$_6$

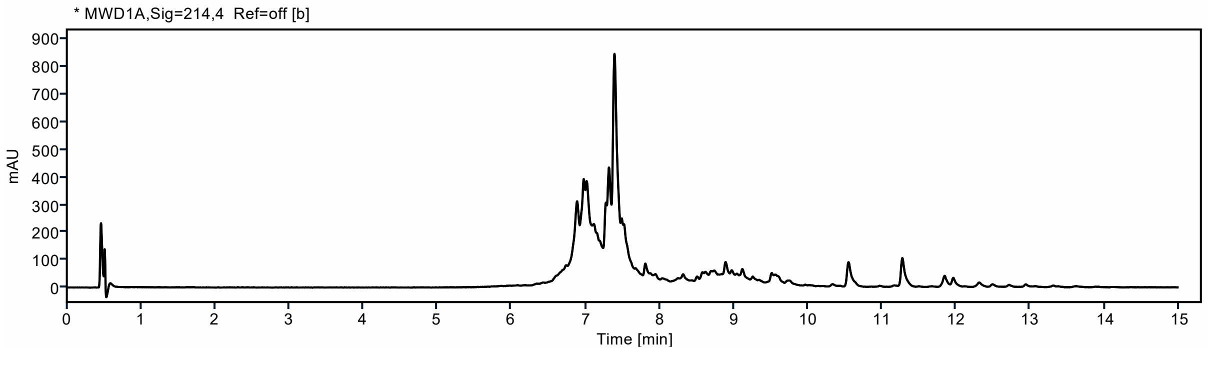

SI Figure 140. UHPLC profile of crude Barstar[75–90] bearing (Lys)$_6$. Rt 7.39 min (Agilent Zorbax 300SB-C18 column, 5 µm, 2.1 × 150 mm, 5–95% MeCN over 10 min, ca. 9.0%B/min), 21% purity based on Area Under Curve (AUC) at λ = 214 nm.

3.13.3 Barstar[75–90]: [Lys(Trt)]$_6$-Tag

```
           10         20
H2N-FREAKAEGCD ITIILSKKKK KK-CONH2      (22 AA)
```

The six tag amino acids were coupled manually as follows: pre-functionalized NovaPEG Rink amide resin (0.41 mmol/g loading) resin (100.4 mg, 41 µmol, 1.0 eq.) was swelled with DCM (1 × 5 mL) for 1 min, drained, and washed with DMF (1 × 5 mL). For each coupling, a solution of Fmoc-Lys(Trt)-OH (513 µL, 0.40 M in DMF, 5.0 eq.) and HATU (505 µL, 0.38 M in DMF, 4.8 eq.) was prepared. To this solution, DIPEA (36 µL, 0.20 mmol, 5 eq.) was added, and the solution was gently agitated at 23 °C for 1 min. The solution was then added to the resin, and the reaction was gently stirred for 20 s, then left at 23 °C for 45 min. The resin was then drained, washed with DMF (3 × 5 mL) and DCM (3 × 5 mL). For each deprotection step, 20% piperidine in DMF (*v*/*v*) (3.0 mL) was added to the resin, and the reaction was gently stirred for 20 s then left at 23 °C for 10 min. The resin was then drained, then washed with DMF (3 × 5 mL) and DCM (3 × 5 mL).

The peptide Barstar[75–90]-[Lys(Trt)]$_6$ was synthesized on the resulting resin using the standard AFPS protocol (**Section 2.2.1**, 20 mL/min flowrate) **SI Figure 141**

Total synthesis time to afford resin-bound Barstar[75–90]-[Lys(Trt)]$_6$ was approximately 0.75 h. Cleavage of the peptidyl-resin (10.4 mg, 4.3 µmol) according to Cleavage Protocol A (**Section 2.5.1**) afforded the crude peptide (4.0 mg, 43% purity by LCMS [**SI Figure 142**] 47% purity by UHPLC [**SI Figure 143**]).

UV-Vis synthesis trace

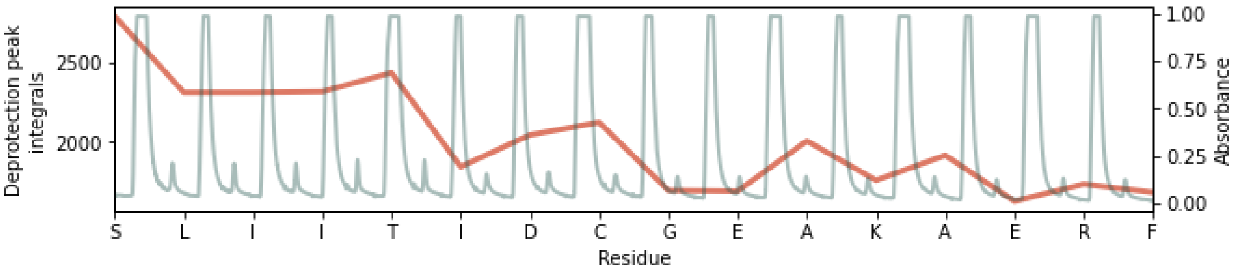

SI Figure 141. UV trace (λ = 310 nm) from AFPS of Barstar[75–90]-[Lys(Boc)]$_6$ (green) and deprotection peak integrals (red).

LC-MS of crude Barstar[75–90]-(Lys)$_6$

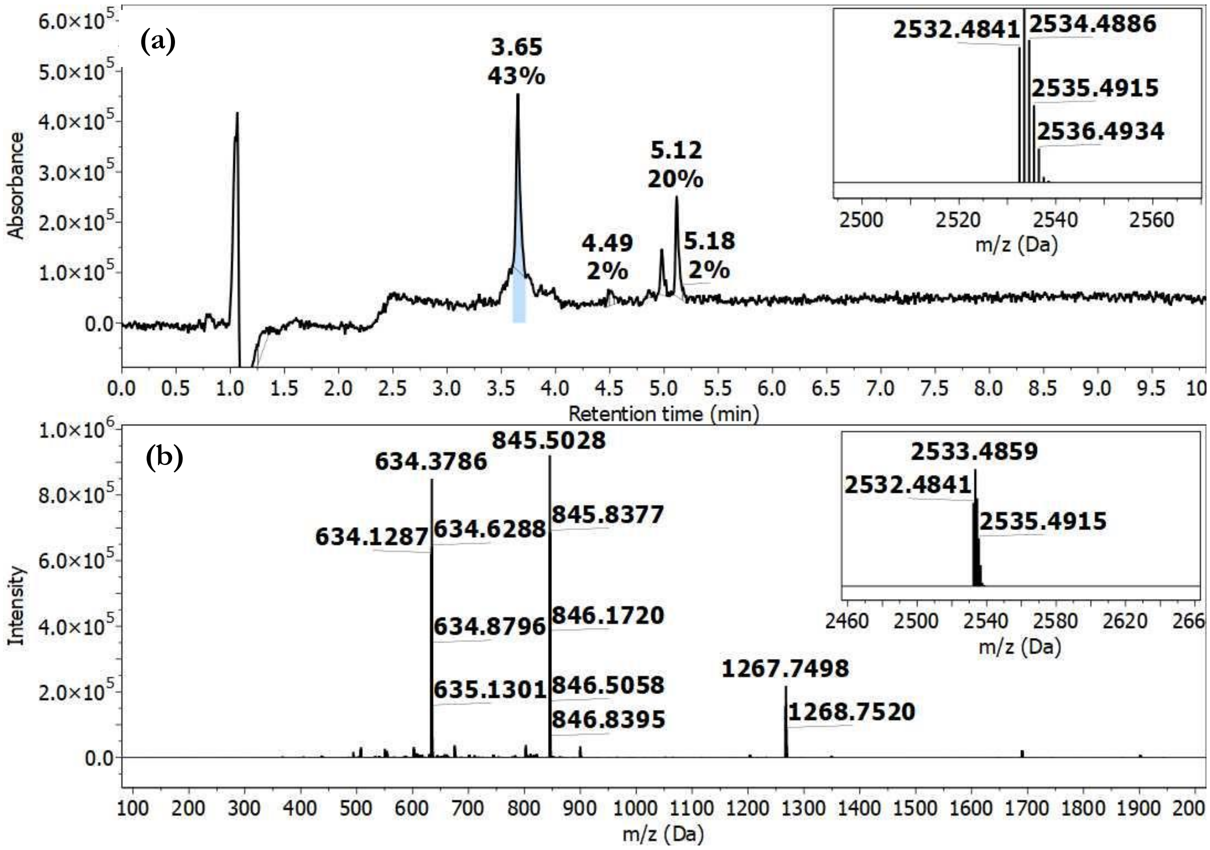

SI Figure 142. LCMS Profile of crude Barstar[75–90] bearing (Lys)$_6$. (**a**) Absorbance chromatogram (λ = 214 nm) of Barstar[75–90]-(Lys)$_6$ tag; Rt 3.65 min, 43% purity. (**b**) ESI-TOF spectrum found within Rt 2–9 min (insert: deconvoluted masses). Monoisotopic mass (ESI+) calcd. for $C_{113}H_{201}N_{33}O_{30}S$ 2532.4938, found 2532.4841. LCMS Gradient B (**Section 2.7**).

UHPLC of crude Barstar[75–90]-(Lys)$_6$

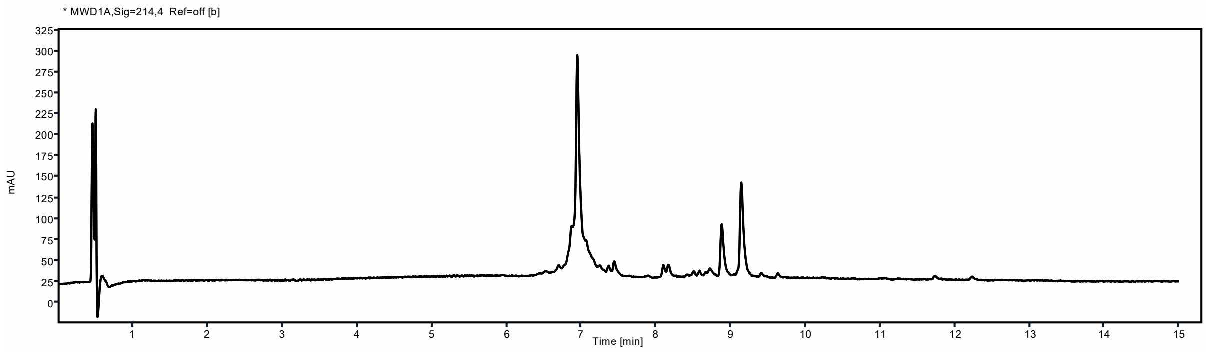

SI Figure 143. UHPLC profile of crude Barstar[75–90] bearing (Lys)$_6$. Rt 6.95 min (Agilent Zorbax 300SB-C18 column, 5 µm, 2.1 × 150 mm, 5–95% MeCN over 10 min, ca. 9.0%B/min), 47% purity based on Area Under Curve (AUC) at λ = 214 nm.

3.13.4 Barstar[75–90]: Without tag (reference)

```
             10        16
H2N-FREAKAEGCD ITIILS-CONH2      (16 AA)
```

The peptide Barstar[75–90] was synthesized on commercially available Novabiochem® NovaPEG Rink Amide resin (0.41 mmol/g, 55.1 mg, 22.5 µmol) using the standard AFPS protocol (**Section 2.2.1**, 20 mL/min flowrate) (**SI Figure 144**). Total synthesis time to afford resin-bound Barstar[75–90] was approximately 0.75 h. Cleavage of the peptidyl-resin (13.5 mg, 5.5 µmol) according to

Cleavage Protocol A (**Section 2.5.1**) afforded the crude peptide (3.0 mg, 46% purity by LCMS [**SI Figure 145**], 44% purity by UHPLC [**SI Figure 159**]).

UV-Vis synthesis trace

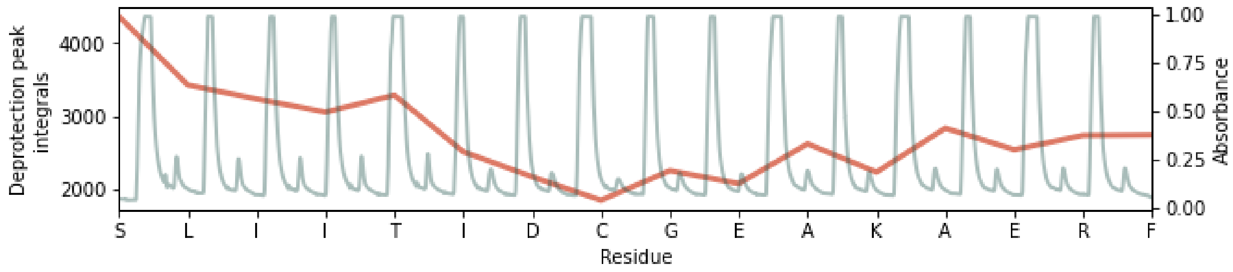

SI Figure 144. UV trace (λ = 310 nm) from AFPS of Barstar[75–90] without tag (green) and deprotection peak integrals (red).

LC-MS of crude Barstar[75–90]

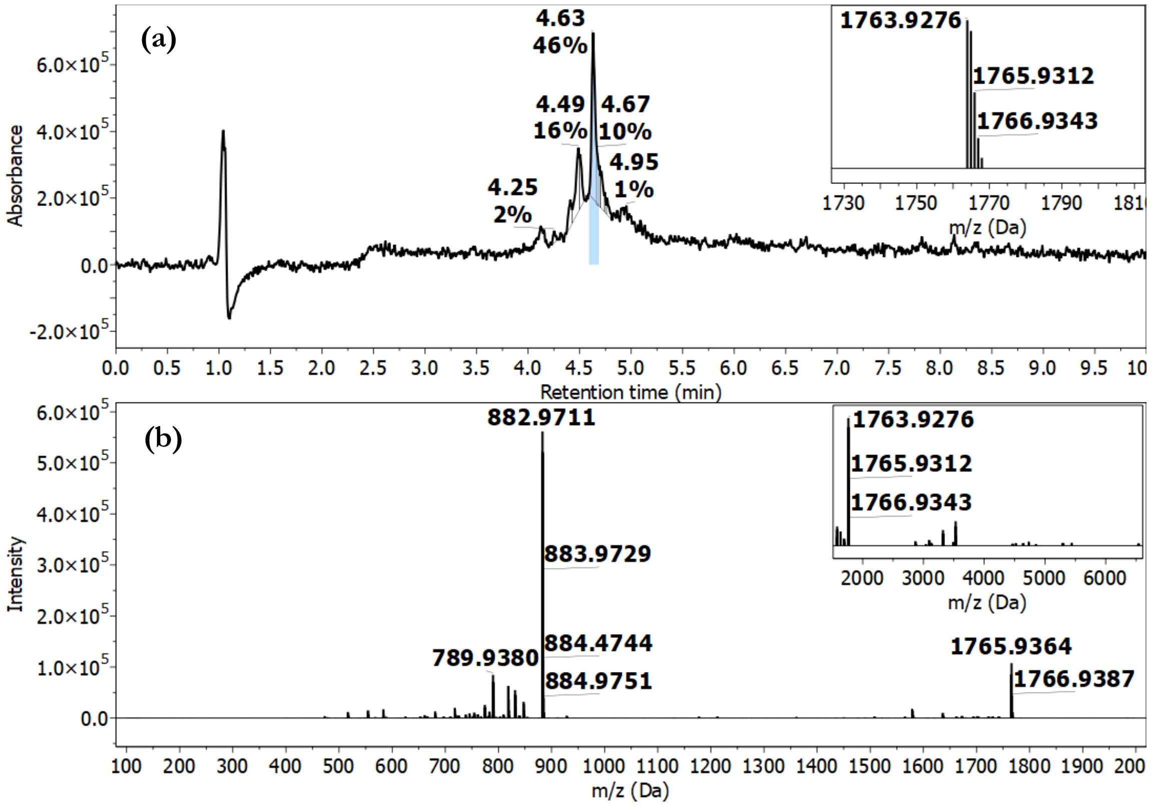

SI Figure 145. LCMS Profile of crude Barstar[75–90] without tag. (**a**) Absorbance chromatogram (λ = 214 nm) of Barstar[75–90]; Rt 4.63 min, 46% purity. (**b**) ESI-TOF spectrum found within Rt 2–9 min (insert: deconvoluted masses). Monoisotopic mass (ESI+) calcd. for $C_{77}H_{129}N_{21}O_{24}S$ 1763.9240, found 1763.9276. LCMS Gradient B (**Section 2.7**).

UHPLC of crude Barstar[75–90]

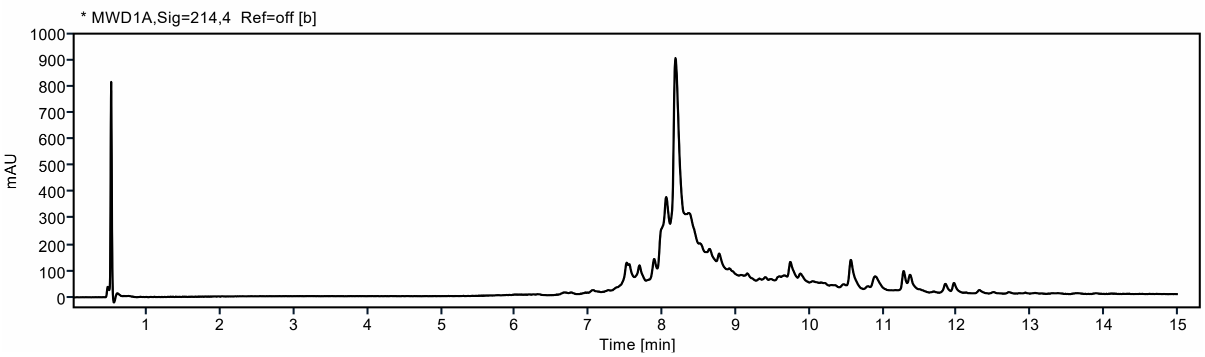

SI Figure 146. UHPLC profile of crude Barstar[75–90] without tag. Rt 8.18 min (Agilent Zorbax 300SB-C18 column, 5 µm, 2.1 × 150 mm, 5–95% MeCN over 10 min, ca. 9%B/min), 44% purity based on Area Under Curve (AUC) at λ = 214 nm.

4 IR evaluation

4.1 Summary of the IR constructs and measurements

SI Table 1 Summary of the constructs used for the IR studies. IR spectras are reported in **SI Section 9**.

Entry	Peptide	On resin?	Sequence	α-helix	β-sheet
1	$[Arg(Pbf)]_9$	No	RRRRRRRRR	1653, 1556	-
2	$[Arg(Pbf)]_{15}$	Yes	RRRRRRRRRRRRRRR	1655, 1542	-
3	Barstar [75–90]	Yes	FREAKAEGCDITIILS	-	1628, 1624
4	Barstar [75–90]-$[Arg(Pbf)]_6$	Yes	FREAKAEGCDITIILSRRRRRR	1651, 1545	1624

4.2 $[Arg(Pbf)]_9$ for IR measurement

The photolinker was grafted to the Novabiochem® NovaPEG Amino resin (0.48 mmol/g, 258 mg, 124 µmol) according to **SI Section 2.3.3**. Nine Fmoc-[Arg(Pbf)]-OH residues were coupled manually according to **SI Section 2.2.4**. Cleavage of the peptidyl resin was performed by suspending the resin-bound peptide (64 mg) in a vial with CH_3CN (3.0 mL) and placing it under irradiation at 365 nm for 3 h. After this time, the supernatant was separated from the resin, and milli-Q water (3.0 mL) was added. Subsequent lyophilization afforded the crude peptide (8.6 mg, 52% purity by LCMS [**SI Figure 160**], which was then measured by IR as outlined in **SI Section 2.9**.

IR ν max (film, cm^{-1}) 3445, 3320, 2970, 2932, 2158, 2016, 1970, 1653, 1556, 1456, 1246, 1093, 835, 667, 617, 567, 507, 445, 419.

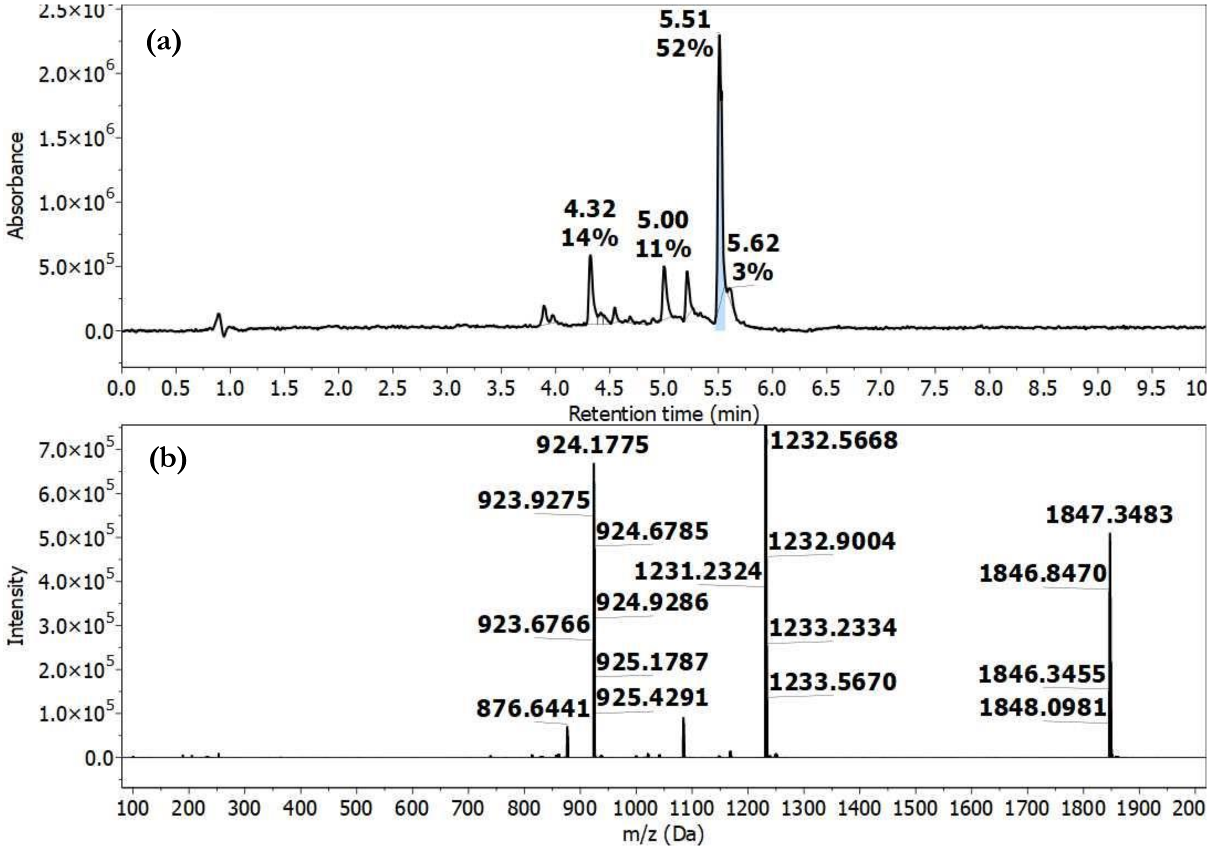

Figure 147. LCMS Profile of crude [Arg(Pbf)]$_9$. (**a**) Absorbance chromatogram (λ = 214 nm) of [Arg(Pbf)]$_9$; Rt 5.51 min, 52% purity. (**b**) ESI-TOF spectrum found within Rt 2–9 min (insert: deconvoluted masses). Monoisotopic mass (ESI+) calcd. for $C_{171}H_{255}N_{37}O_{36}S_9$ 3690.6747, found 3690.6747. LCMS Gradient: gradient of 30–98% Solvent B over 5 min, followed by isocratic at 98% Solvent B for 5 min.

4.3 Resin-Bound Barstar[75–90]

See **Section 5.1** for the synthesis of the construct.

IR ν max (film, cm^{-1}) 3281, 2925, 2871, 1628, 1624, 1537, 1457, 1247, 1088, 1031, 955.

4.4 Resin-Bound [Arg(Pbf)]$_{15}$

See **Section 5.3** for the synthesis of the construct.

IR ν max (film, cm^{-1}) 3325, 2931, 2868, 1724, 1655, 1617, 1542, 1452, 1248, 1089, 1089, 1033, 851, 812, 783, 660, 640, 619, 565, 418, 409.

4.5 Resin-Bound Barstar[75–90]-[Arg(Pbf)]$_6$

See **Section 5.2** for the synthesis of the construct.

IR ν max (film, cm^{-1}) 3293, 2928, 2874, 2361, 2156, 2015, 1974, 1651, 1624, 1545, 1453, 1244, 1092.

5 NMR evaluation

5.1 Barstar[75–90] ^{13}C-labeled

```
          10        16
H2N-FREAKAEGCD ITIILS-CONH2      (16 AA)
```

The peptide Barstar[75–90] was synthesized on commercially available Novabiochem® NovaPEG Rink Amide resin (0.41 mmol/g, 120 mg, 48 µmol) using the standard AFPS protocol (**Section 2.2.1**, 40 mL/min flowrate). Residues Ile[85], Gly[82] and Ala[78] were coupled manually using a 1:1 mixture of ^{13}C-labeled (**SI Figure 148**) and unlabeled (natural isotope abundance) Fmoc-protected building blocks using the standard batch SPPS protocol (**Section 2.2.4**) at 23 °C. Total synthesis time to afford resin-bound Barstar[75–90] was approximately 4 h. Cleavage of the peptidyl-resin (9 mg, approx. 4.5 µmol) according to Cleavage Protocol A (**Section 2.5.1**) afforded the crude peptide as a colorless solid (1.6 mg, 56% purity by LCMS [**SI Figure 149**], 38% purity by UHPLC [**SI Figure 150**]).

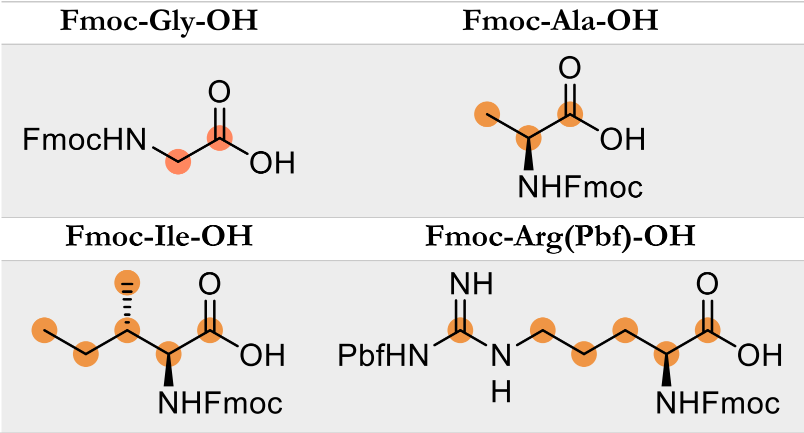

SI Figure 148. ^{13}C-labeled amino acid building blocks used for NMR experiments. ^{13}C-labeled atoms are highlighted in orange.

LC-MS of crude Barstar[75–90] ^{13}C-labeled

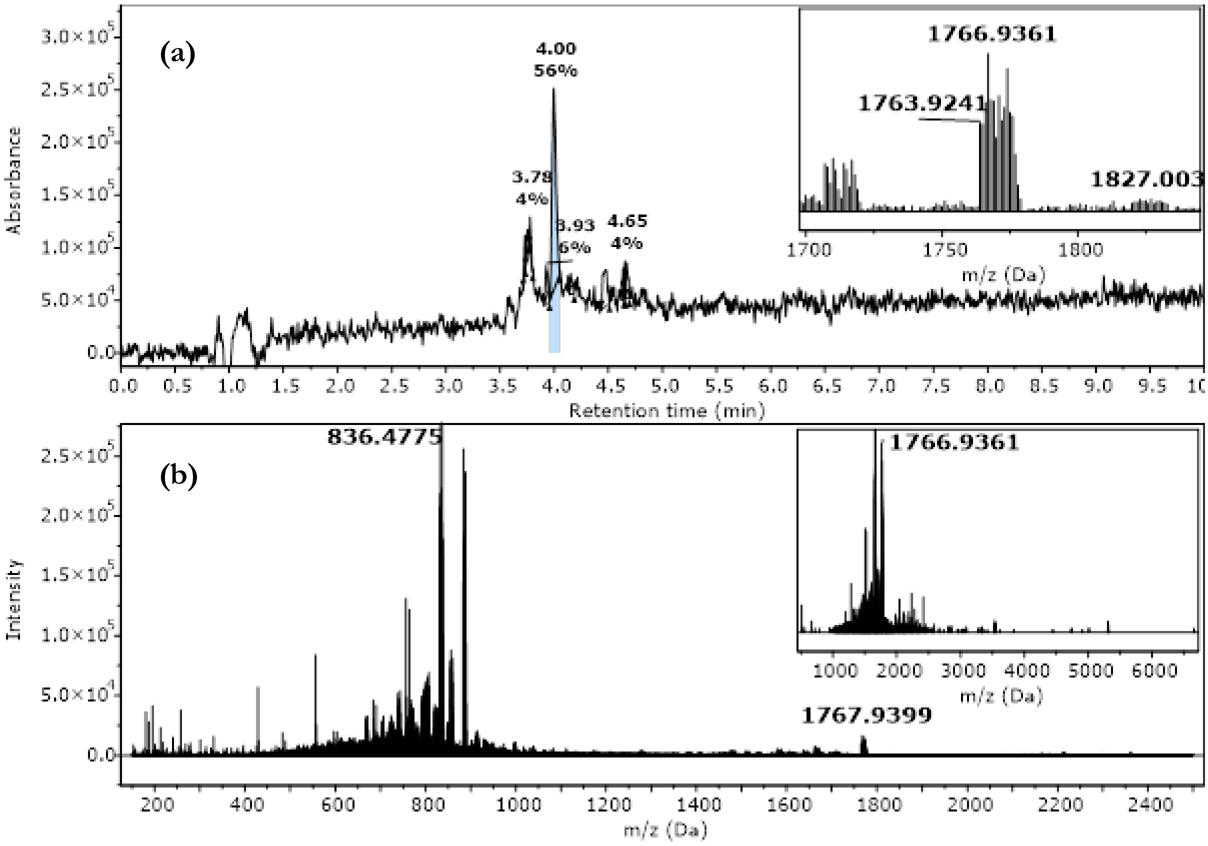

SI Figure 149. LCMS Profile of crude Barstar[75–90]. (**a**) Absorbance chromatogram (λ = 214 nm) of Barstar[75–90] ; Rt 4.00 min, 56% purity. (**b**) ESI-TOF spectrum found within Rt 2–9 min (insert: deconvoluted masses). Monoisotopic mass (ESI+) calcd. for $C_{77}H_{129}N_{21}O_{24}S$ 1763.9240, found 1763.8900. LCMS Gradient A (**Section 2.7**).

UHPLC of crude Barstar[75–90] ^{13}C-labeled

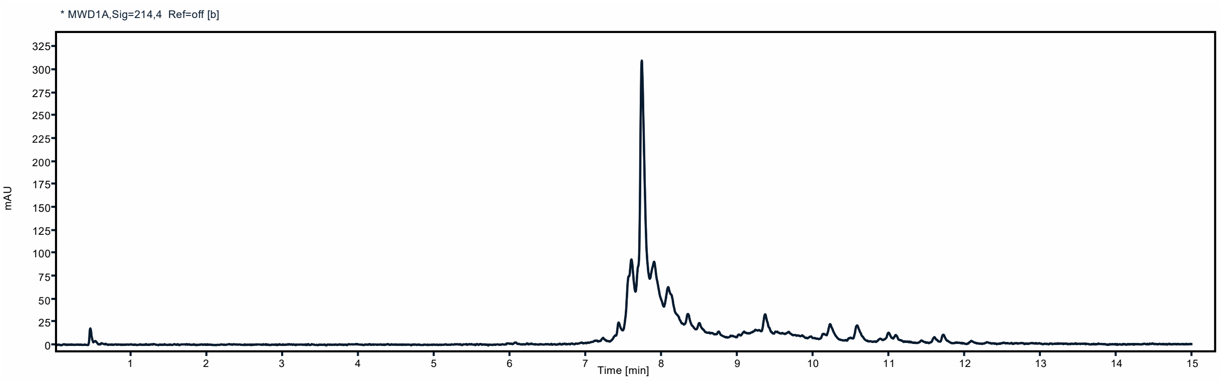

SI Figure 150. UHPLC profile of crude Barstar[75–90] ^{13}C-labeled, synthesized at room temperature (23 °C) using batch SPPS for ^{13}C-labeled amino acid and SPPS for standard amino acid coupling. Rt 7.734 min (Agilent Zorbax 300SB-C18 column, 5 µm, 2.1 × 150 mm, 5–95% MeCN over 10 min, ca. 9%B/min), 38% purity based on Area Under Curve (AUC) at λ = 214 nm.

5.2 Barstar[75–90]: [Arg(Pbf)]$_6$-Tag ^{13}C-labeled

```
                    10                  20
   H2N-FREAKAEGCD  ITIILSRRRR RR-CONH2         (22 AA)
```

The peptide Barstar[75–90] bearing the [Arg(Pbf)]$_6$ tag was synthesized on commercially available Novabiochem® NovaPEG Rink Amide resin (0.41 mmol/g, 120 mg, 49 µmol) using the standard

AFPS protocol (**Section 2.2.1**, 40 mL/min flowrate). Residues Ile[85], Gly[82], Ala[78], and Arg[92] were coupled manually using a 1:1 mixture of the ^{13}C-labeled (**SI Figure 148**) and unlabeled (natural isotope abundance) Fmoc-protected building blocks using the standard batch SPPS protocol (**Section 2.2.4**) at 23 °C. Total synthesis time to afford resin-bound Barstar[75–90]-[Arg(Pbf)]$_6$ was approximately 5 h. Cleavage of the peptidyl-resin (12 mg, approx. 5.4 µmol) according to Cleavage Protocol A (**Section 2.5.1**) afforded the crude peptide (1.7 mg, 55% purity by LCMS [**SI Figure 151**], 82% purity by UHPLC [**SI Figure 152**]).

LC-MS of crude Barstar[75–90]-(Arg)$_6$ ^{13}C-labeled

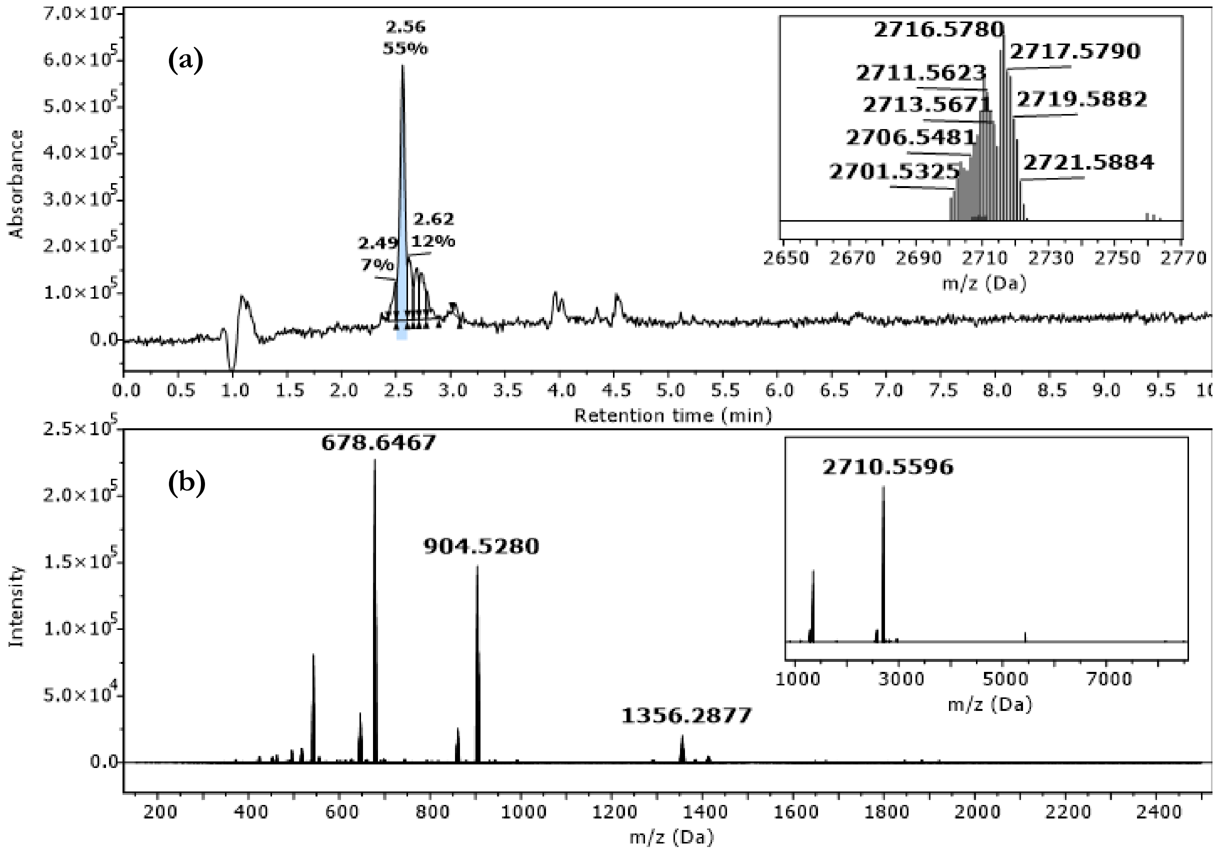

SI Figure 151. LCMS Profile of Barstar[75–90] bearing (Arg)$_6$. (**a**) Absorbance chromatogram (λ = 214 nm) of Barstar[75–90]-(Arg)$_6$; Rt 2.56 min, 55% purity. (**b**) ESI-TOF spectrum found within Rt 2–9 min (insert: deconvoluted masses). Monoisotopic mass (ESI+) calcd. for $C_{113}H_{200}N_{44}O_{31}S$ 2700.5307, found 2700.5233. LCMS Gradient A (**Section 2.7**).

UHPLC of crude Barstar[75–90]-(Arg)$_6$ ^{13}C-labeled

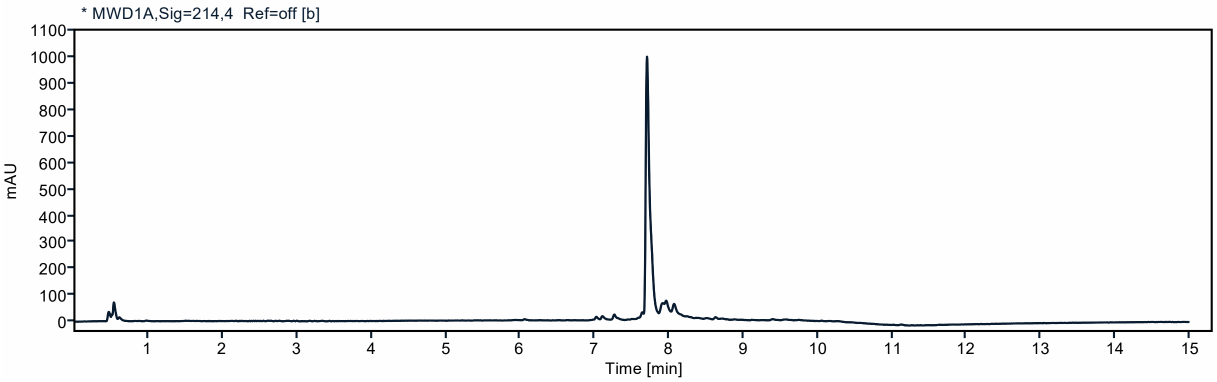

SI Figure 152. UHPLC profile of crude Barstar[75–90] bearing (Arg)$_6$. Rt 7.70 min (Agilent Zorbax 300SB-C18 column, 5 µm, 2.1 × 150 mm, 5–95% MeCN over 20 min, ca. 4.5%B/min), 82% purity based on Area Under Curve (AUC) at λ = 214 nm.

5.3 [Arg(Pbf)]$_{15}$ ^{13}C-labeled

```
               10        15
H2N-RRRRRRRRRR RRRRR-CONH2        (15 AA)
```

The peptide [Arg(Pbf)]$_{15}$ tag was synthesized on commercially available Novabiochem® NovaPEG Rink Amide resin (0.41 mmol/g, 120 mg, 49 µmol) using the standard AFPS protocol (**Section 2.2.1**, 40 mL/min flowrate). Residue Arg[7] was coupled manually using a 1:1 mixture of the ^{13}C-labeled (**SI Figure 148**) and unlabeled (natural isotope abundance) Fmoc-protected building blocks using the standard batch SPPS protocol (**Section 2.2.4**) at 23 °C. Total synthesis time to afford resin-bound [Arg(Pbf)]$_{15}$ was approximately 2 h. Cleavage of the peptidyl-resin (13 mg, approx. 1.8 µmol) according to Cleavage Protocol A (**Section 2.5.1**) afforded the crude peptide (3.0 mg, 69% purity by LCMS [**SI Figure 153**], 91% purity by UHPLC [**SI Figure 154**]).

LC-MS of crude (Arg)$_{15}$ ^{13}C-labeled

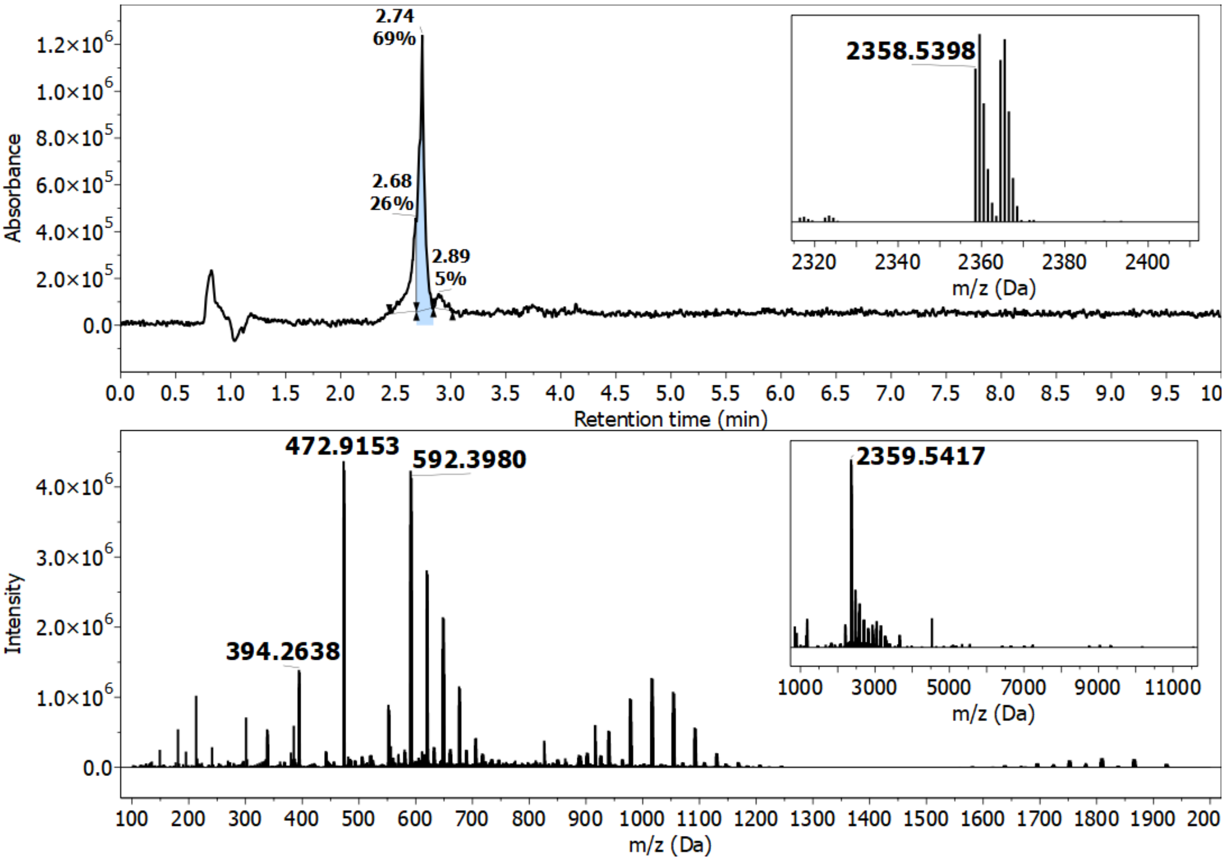

SI Figure 153. LCMS Profile of (Arg)$_{15}$. (**a**) Absorbance chromatogram (λ = 214 nm) of Arg)$_6$; Rt 2.74 min, 69% purity. (**b**) ESI-TOF spectrum found within Rt 2–9 min (insert: deconvoluted masses). Monoisotopic mass (ESI+) calcd. for $C_{90}H_{183}N_{61}O_{15}$ 2358.5432, found 2358.5398 LCMS Gradient A (**Section 2.7**).

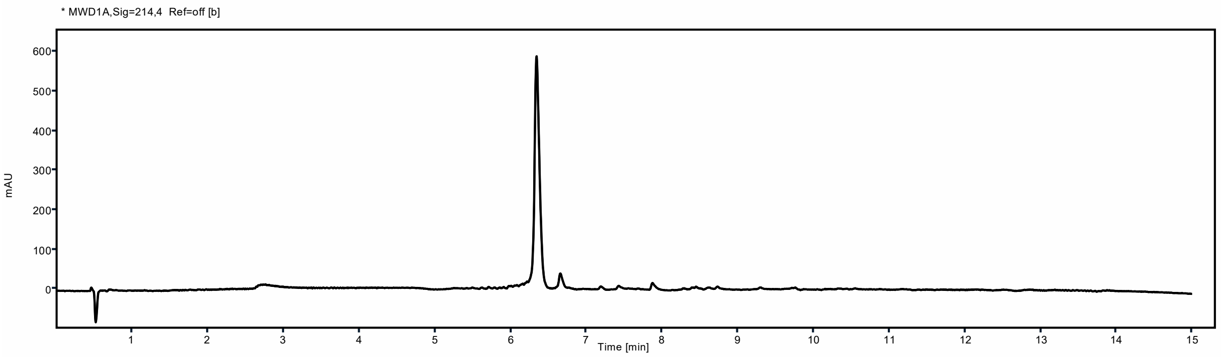

SI Figure 154. UHPLC profile of crude (Arg)$_{15}$. Rt 6.34 min (Agilent Zorbax 300SB-C18 column, 5 µm, 2.1 × 150 mm, 5–95% MeCN over 20 min, ca. 4.5%B/min), 91% purity based on Area Under Curve (AUC) at λ = 214 nm.

5.4 Solid-State NMR (SSNMR)

The dried, resin-bound peptides were packed into Varian 3.2 mm rotors without further treatment. For the samples solvated with DMF, 10 μL DMF was added to ~20 mg resin samples. The solvated samples were then transferred into Varian 3.2 mm rotors using centrifugation. The 3.2 mm rotors were sealed using Kel-F spacers with O-rings for the DMF re-solvated samples.

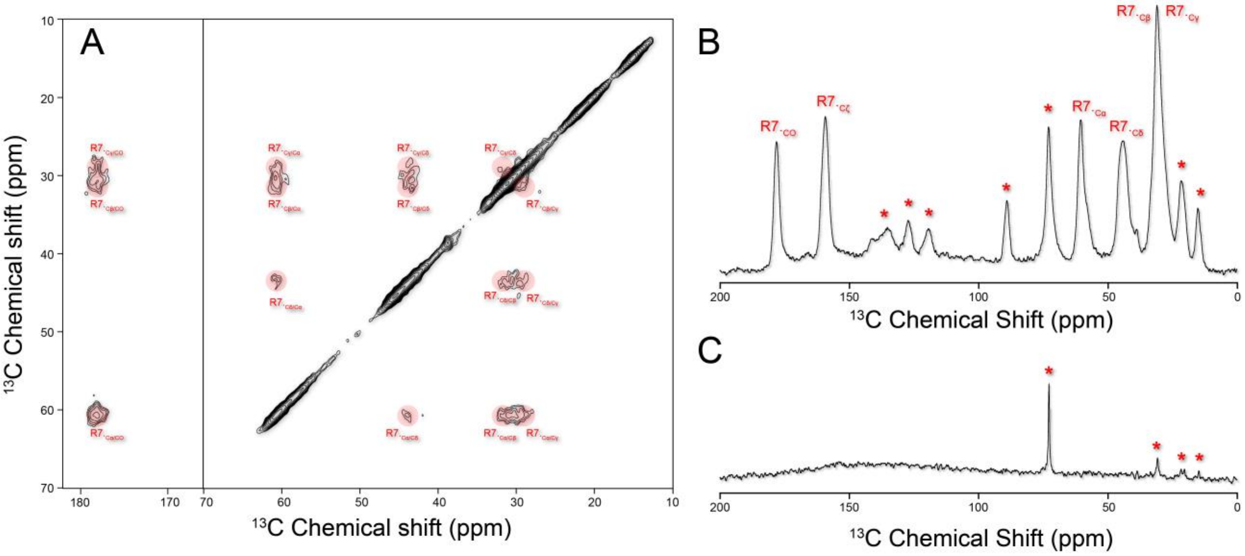

SI Figure 155. A) 2D ^{13}C/^{13}C correlation SSNMR spectrum of resin-bound $[Arg(Pbf)]_{15}$ under dry conditions. The red circles and assignments indicate a formation of α-helix structure. The secondary chemical shift analysis result is shown in **SI Table 4**. B) ^{13}C CP 1D spectrum of resin-bound $[Arg(Pbf)]_{15}$ under dry conditions. The * indicates the peaks from resin. C) ^{13}C CP 1D spectrum of resin-bound $[Arg(Pbf)]_{15}$ re-solvated using DMF. The resonance peaks from $[Arg(Pbf)]_{15}$ and most resonance peaks from resin disappeared from the spectrum due to increased flexibility.

SI Table 2 ^{13}C chemical shifts for ^{13}C-labeled residues in resin-bound Barstar[75–90] without arginine linker

Residues and sites		Experimental chemical shift (ppm)[a]	Chemical shift for random coil (ppm)*	Secondary chemical shift (ppm)	Dihedral angles (°)[b]
Ala-4	Cα	50.5	52.5	-2.0	Φ=-136±13 Ψ=148±17
	Cβ	23.4	19.1	4.3	
	CO	174.6	177.8	-3.2	
Gly-8	Cα	45.5	45.1	0.4	Φ=-159±8 Ψ=154±21
	CO	171.1	174.9	-3.8	
Ile-11	Cα	60.5	61.1	-0.6	Φ=-131±16 Ψ=146±22
	Cβ	42	38.8	3.2	
	Cγ1	27.8	27.2	0.6	
	Cγ2	17.5	17.4	0.1	
	Cδ	14.8	12.9	1.9	
	CO	173.5	176.4	-2.9	

a) All the ^{13}C chemical shifts shown above are referenced to DSS. b) Torsion angles were predicted using TALOS*.

* Wishart, D. S.; Bigam, C. G.; Holm, A.; Hodges, R. S.; Sykes, B. D. 1H, 13C and 15N Random Coil NMR Chemical Shifts of the Common Amino Acids. I. Investigations of Nearest-Neighbor Effects. *J. Biomol. NMR* **1995**, *5* (1), 67–81. https://doi.org/10.1007/BF00227471.

** Cornilescu, G.; Delaglio, F.; Bax, A. Protein Backbone Angle Restraints from Searching a Database for Chemical Shift and Sequence Homology. *J. Biomol. NMR* **1999**, *13* (3), 289–302. https://doi.org/10.1023/A:1008392405740.

SI Table 3 . [13]C chemical shifts for α-helix component of ^{13}C-labeled residues in resin-bound Barstar[75–90] with $[Arg(Pbf)]_6$ linker

Residues and sites		Experimental chemical shift (ppm)	Chemical shift for random coil (ppm)	Secondary chemical shift (ppm)	Dihedral angles (°)
Ala-4	Cα	55.1	52.5	2.6	Φ=-64±6<br>Ψ=-39±9
	Cβ	18.2	19.1	-0.9	
	CO	179.2	177.8	1.4	
Gly-8	Cα	47.7	45.1	2.6	Φ=-71±20<br>Ψ=-27±22
	CO	174.1	174.9	-0.8	
Ile-11	Cα	65.9	61.1	4.8	Φ=-61±6<br>Ψ=-44±3
	Cβ	37.8	38.8	-1.0	
	Cγ1	30.5	27.2	3.3	
	Cγ2	17.4	17.4	0.0	
	Cδ	14.1	12.9	1.2	
	CO	177.8	176.4	1.4	
Arg-linker	Cα	60.4	56.6	3.8	Φ=-62±9<br>Φ=-43±8
	Cβ	30.2	30.9	-0.7	
	CO	178.4	176.3	2.1	

SI Table 4 ^{13}C chemical shifts for β-sheet component of ^{13}C-labeled residues in resin-bound Barstar[75–90] with $[Arg(Pbf)]_6$ linker

Residues and sites		Experimental chemical shift (ppm)	Chemical shift for random coil (ppm)	Secondary chemical shift (ppm)	Dihedral angles (°)
Ala-4	Cα	50.7	52.5	-1.8	Φ=-143±14<br>Ψ=153±13
	Cβ	23.5	19.1	4.4	
	CO	175.0	177.8	-2.8	
Gly-8	Cα	45.9	45.1	0.8	Φ=-161±9<br>Ψ=147±12
	CO	170.5	174.9	-4.4	
Ile-11	Cα	60.8	61.1	-0.3	Φ=-127±13<br>Ψ=140±20
	Cβ	42.4	38.8	3.6	
	Cγ1	28.1	27.2	0.9	
	Cγ2	17.2	17.4	-0.2	
	Cδ	14.0	12.9	1.1	
	CO	173.6	176.4	-2.8	
Arg-linker	Cα	55.3	56.6	-1.3	Φ=-146±12<br>Φ=-145±14
	Cβ	34.6	30.9	3.7	
	CO	173.3	176.3	-3.0	

SI Table 5 ^{13}C chemical shifts of ^{13}C-labeled residues in resin-bound [Arg(Pbf)]$_{15}$.

Residues and sites		Experimental chemical shift (ppm)	Chemical shift for random coil (ppm)	Secondary chemical shift (ppm)	Dihedral angles (°)
Arg-7	Cα	60.7	56.6	4.1	Φ=-61±9<br>Ψ=-42±10
	Cβ	31.5	30.9	0.6	
	Cγ	29.7	27.1	2.6	
	Cδ	43.9	43.3	0.6	
	CO	178.2	176.3	1.9	

SI Table 6 The percentage of α-helix component and β-strand component of resin-bound Barstar[75–90] with [Arg(Pbf)]$_6$ linker.

Residues	α-helix*	β-strand*
Ala-4	34.0%	66.0%
Gly-8	32.5%	67.5%
Ile-11	31.0%	69.0%
Arg-linker	30.8%	69.2%

* The percentages are evaluated using the integral intensity of $^{13}C_{\alpha}/^{13}CO$ cross peak for each ^{13}C-labeled residue.

6 Linker screening

6.1 Evaluation of cleavable linkers: GLP-1

6.1.1 GLP-1: Novabiochem® NovaPEG Rink Amide resin

```
            10          20          30
H2N-HAEGTFTSDV  SSYLEGQAAK  EFIAWLVKGR-CONH2   (30 AA)
```

The peptide GLP-1 was synthesized on commercially available Novabiochem® NovaPEG Rink Amide resin (0.41 mmol/g, 54 mg, 22 µmol) using the standard AFPS protocol (**Section 2.2.1**, 20 mL/min flowrate) (**SI Figure 156**). Total synthesis time to afford resin-bound GLP-1 was approximately 1.5 h. Cleavage of the peptidyl-resin (18 mg, approx. 7.2 µmol) according to Cleavage Protocol A (**Section 2.5.1**) afforded the crude peptide (8.1 mg, 88% purity by LCMS [**SI Figure 157**], 80% purity by UHPLC [**SI Figure 158**]).

UV-Vis synthesis trace

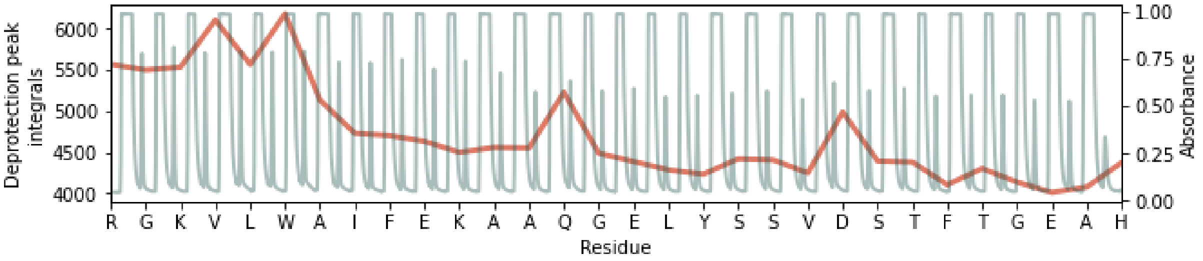

SI Figure 156. UV trace (λ = 310 nm) from AFPS of GLP-1 (green) and deprotection peak integrals (red), synthesized on Novabiochem® NovaPEG Rink Amide resin.

LC-MS of crude GLP-1

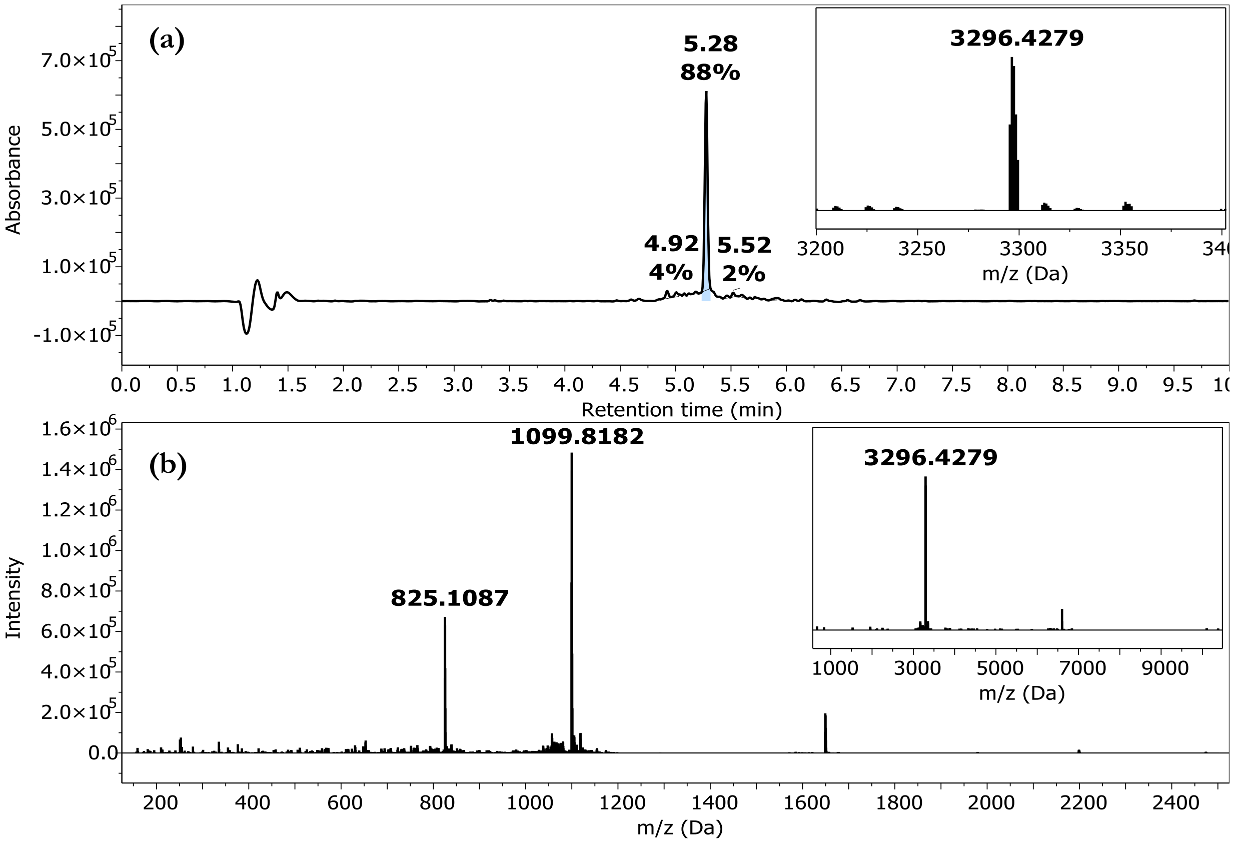

SI Figure 157. LC-MS Profile of crude GLP-1 synthesized on Rink amide resin. (a) Absorbance chromatogram (λ = 214 nm) of GLP-1; Rt 5.28 min, 88% purity. (b) ESI-TOF spectrum found within Rt 2–9 min (insert: deconvoluted masses); Monoisotopic mass (ESI+) calcd. For $C_{149}H_{226}N_{40}O_{45}$ 3295.6626, found 3295.4259. LCMS Gradient A (**Section 2.7**).

UHPLC of crude GLP-1

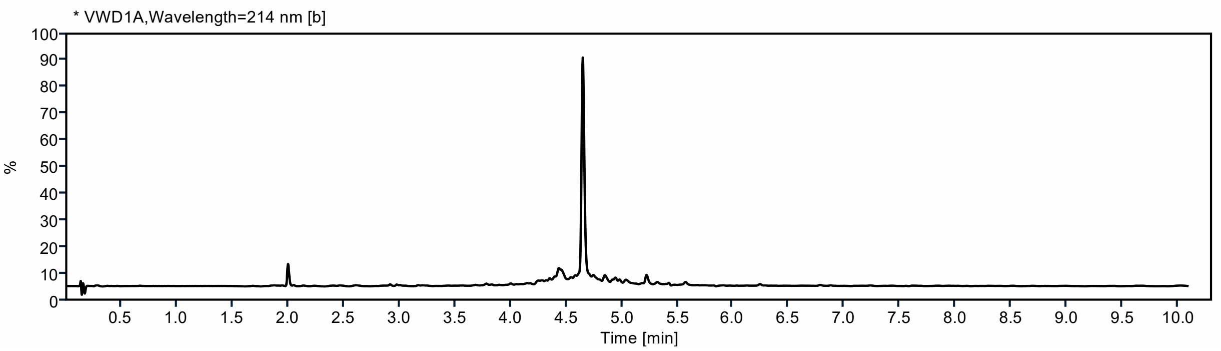

SI Figure 158. UHPLC profile of crude GLP-1 synthesized on Rink amide resin**.** Rt 4.65 min (Agilent Zorbax 300SB-C18 column, 5 µm, 2.1 × 150 mm, 5–95% MeCN over 10 min, ca. 9%B/min), 80% purity based on Area Under Curve (AUC) at λ = 214 nm.

6.1.2 GLP-1: HMPB ChemMatrix® resin

```
                10                  20                  30
H2N-HAEGTFTSDV SSYLEGQAAK   EFIAWLVKGR-COOH   (30 AA)
```

HMPB ChemMatrix® resin (0.62 mmol/g, 0.10 g, 66 µmol) was manually grafted with Fmoc-Arg(Pbf)-OH (0.51 g, 0.32 mmol) using the protocol outline in **Section 2.3.2**. The loading of the resin was determined using the Fmoc-loading test described in **Section 2.4**, which resulted in a loading of 0.62 mmol/g. The peptide GLP-1 was then prepared using the standard AFPS

protocol (**Section 2.2.1**, 20 mL/min flowrate) (**SI Figure 159**). Total synthesis time to afford resin-bound GLP-1 was approximately 1.5 h. Cleavage of the peptidyl-resin (42 mg, approx. 26 μmol) according to Cleavage Protocol A (**Section 2.5.1**) afforded the crude peptide (4.1 mg, 89% purity by LCMS [**SI Figure 160**], 66% purity by UHPLC [**SI Figure 161**]).

UV-Vis synthesis trace

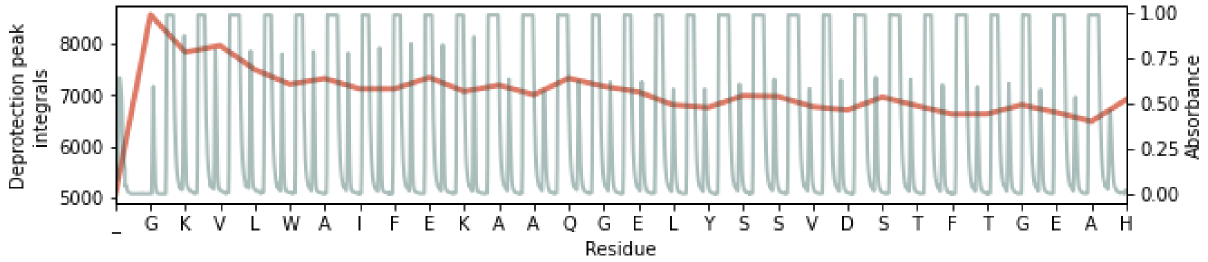

SI Figure 159. UV trace (λ = 310 nm) from AFPS of GLP-1 (green) and deprotection peak integrals (red), synthesized on HMPB ChemMatrix® resin.

LC-MS of crude GLP-1

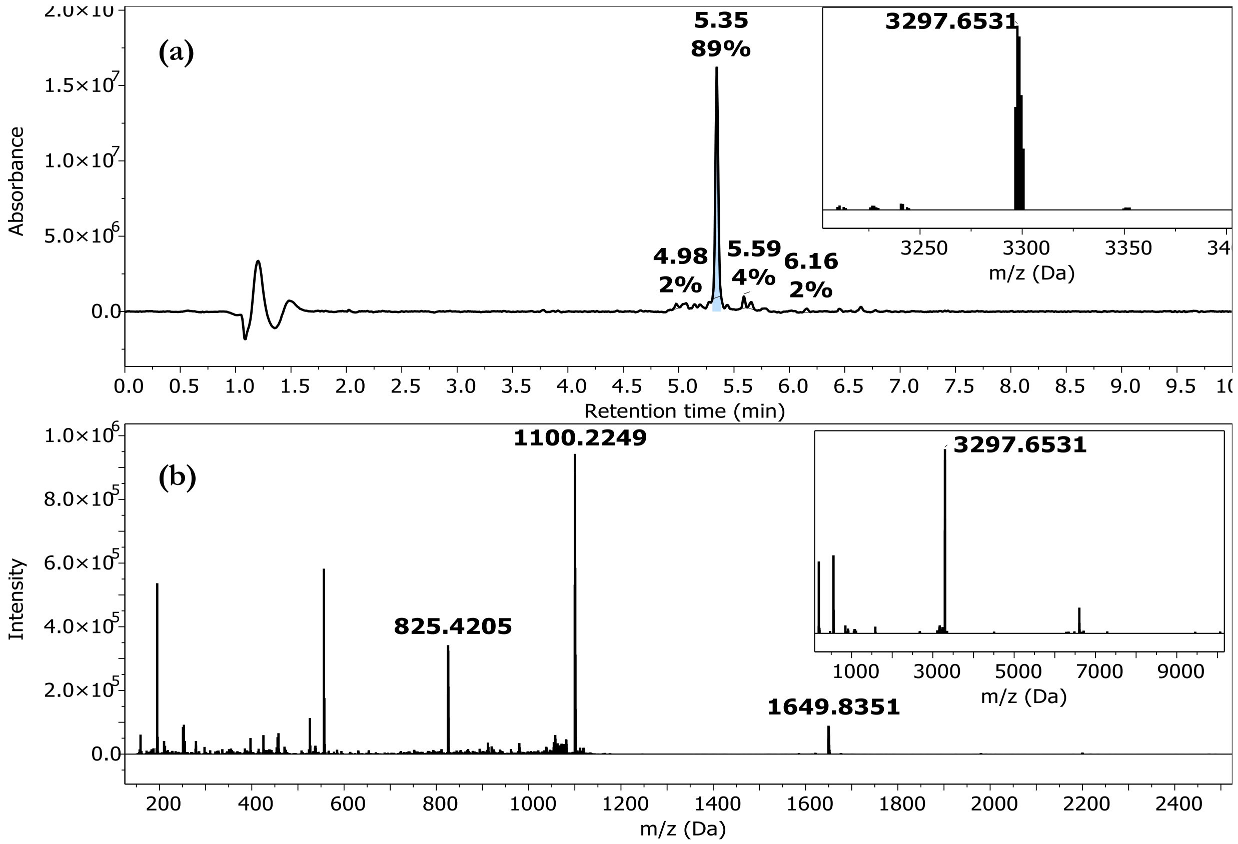

SI Figure 160. LC-MS Profile of crude GLP-1. (a) Absorbance chromatogram (λ = 214 nm) of peptide GLP-1; Rt 5.35 min, 89% purity. (b) ESI-TOF spectrum found within Rt 2–9 min (insert: deconvoluted masses); Monoisotopic mass (ESI+) calcd. for $C_{149}H_{226}N_{40}O_{45}$ 3296.6466, found 3296.6509. LCMS Gradient A (**Section 2.7**).

UHPLC of crude GLP-1

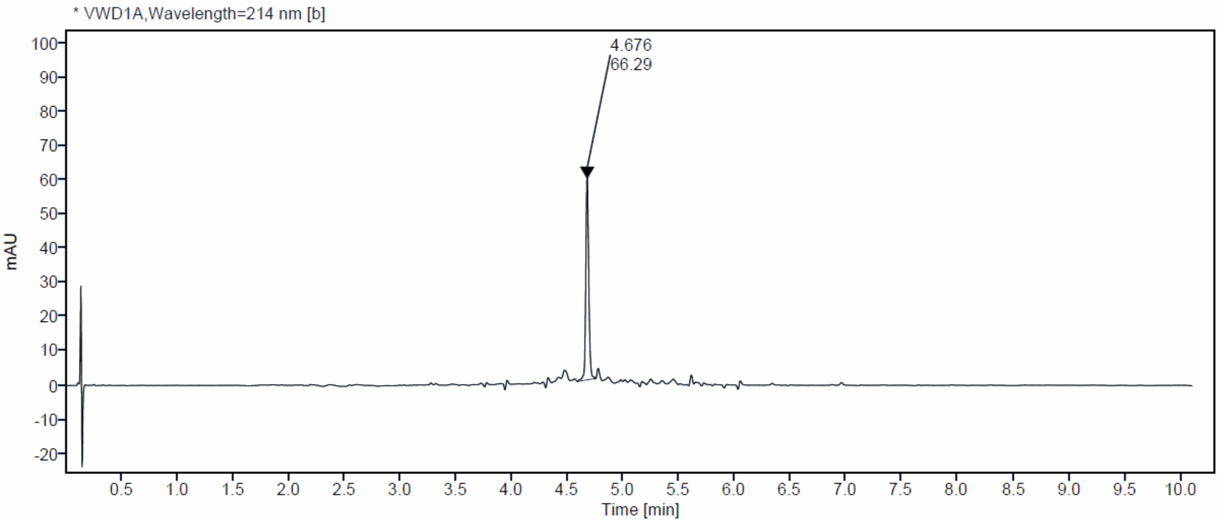

SI Figure 161. UHPLC profile of crude GLP-1. Rt 4.68 min (Agilent Zorbax 300SB-C18 column, 5 µm, 2.1 × 150 mm, 5–95% MeCN over 10 min, ca. 9%B/min), 66% purity based on Area Under Curve (AUC) at λ = 214 nm.

6.1.3 GLP-1: Photolinker on Novabiochem® NovaPEG resin

```
             10          20          30
H2N-HAEGTFTSDV  SSYLEGQAAK  EFIAWLVKGR-CONH2   (30 AA)
```

The peptide GLP-1 was synthesized on the manually Photolinker-grafted resin as described in **Section 2.3.3** (0.48 mmol/g, 54 mg, 26 µmol) using the standard AFPS protocol (**Section 2.2.1**, 20 mL/min flowrate). Total synthesis time to afford resin-bound GLP-1 was approximately 1.5 h. Cleavage of the peptidyl-resin (14 mg, approx. 6.8 µmol) according to Cleavage Protocol B (**Section 2.5.2**) afforded the crude peptide (60% purity by LCMS [**SI Figure 163**]).

UV-Vis synthesis trace

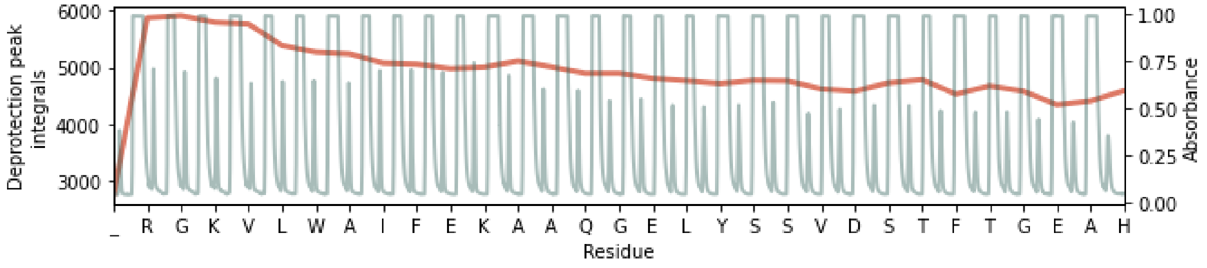

SI Figure 162. UV trace (λ = 310 nm) from AFPS of GLP-1 (green) and deprotection peak integrals (red), synthesized on Photolinker on Novabiochem® NovaPEG resin.

LC-MS of crude GLP-1

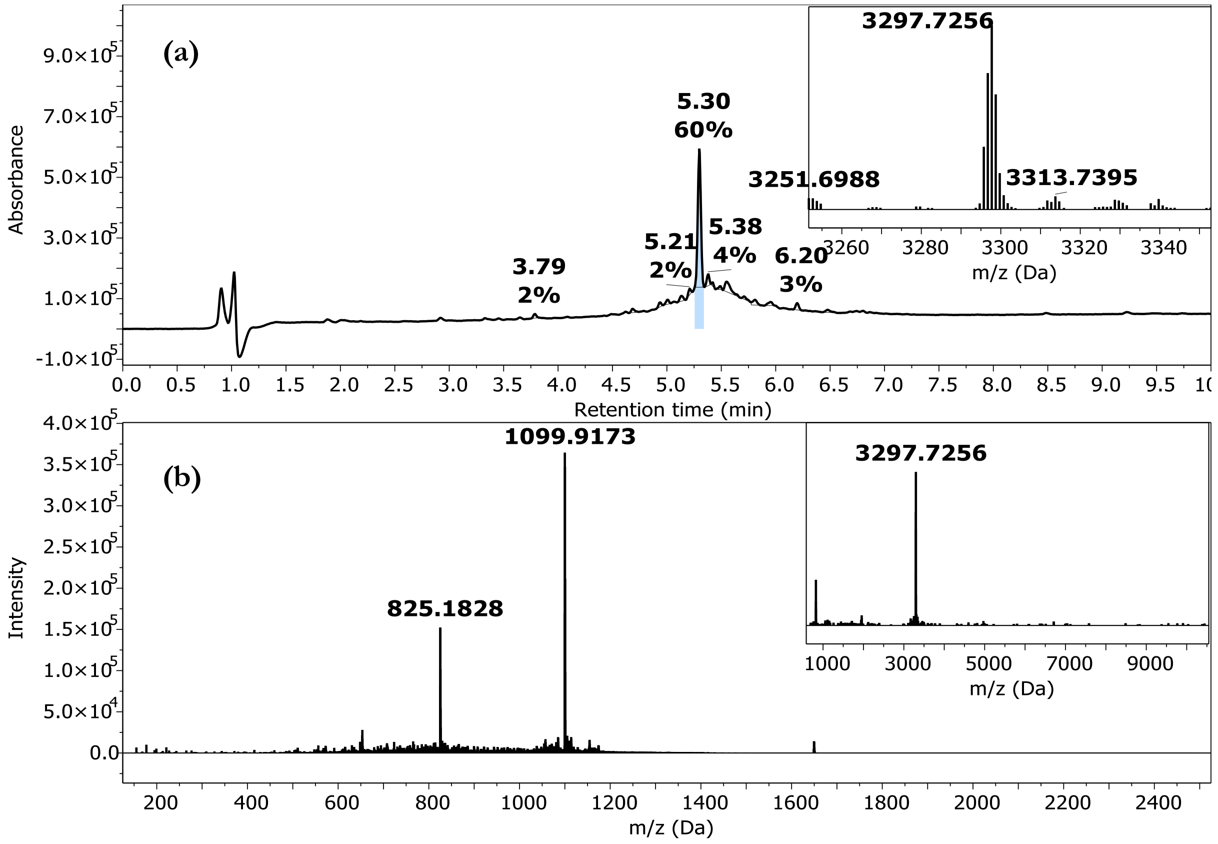

SI Figure 163. LC-MS Profile of crude GLP-1. (**a**) Absorbance chromatogram (λ = 214 nm) of peptide GLP-1; Rt 5.30 min, 60% purity. (**b**) ESI-TOF spectrum found within Rt 2–9 min (insert: deconvoluted masses); Monoisotopic mass (ESI+) calcd. for $C_{149}H_{226}N_{40}O_{45}$ 3295.6626, found 3295.0736. LCMS Gradient A (**Section 2.7**).

6.1.4 GLP-1: MeDbz linker on Novabiochem® NovaPEG resin

```
                   10            20             30
H2N-HAEGTFTSDV  SSYLEGQAAK   EFIAWLVKGR-COOH   (30 AA)
```

The peptide GLP-1 was synthesized on the manually MeDbz-grafted resin as described in **Section 2.3.4** (0.48 mmol/g, 0.10 g, 48 µmol) using the standard AFPS protocol (**Section 2.2.1**, 20 mL/min flowrate) (**SI Figure 164**). Total synthesis time to afford resin-bound GLP-1 was approximately 1.5 h. Cleavage of the peptidyl-resin (8.7 mg, 4.0 µmol) according to Cleavage Protocol C (**Section 2.5.3**) afforded the crude peptide (88% purity by LCMS [**SI Figure 165**]). Two other procedures inspired from the literature were also attempted, (*57*, *58*) but did not yield any of the expected product.

UV-Vis synthesis trace

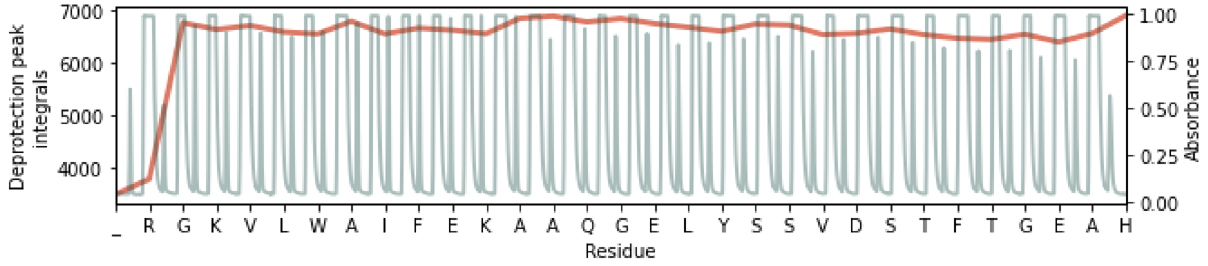

SI Figure 164. UV trace (λ = 310 nm) from AFPS of GLP-1 (green) and deprotection peak integrals (red), synthesized on MeDbz linker on Novabiochem® NovaPEG resin.

LCMS of crude GLP-1

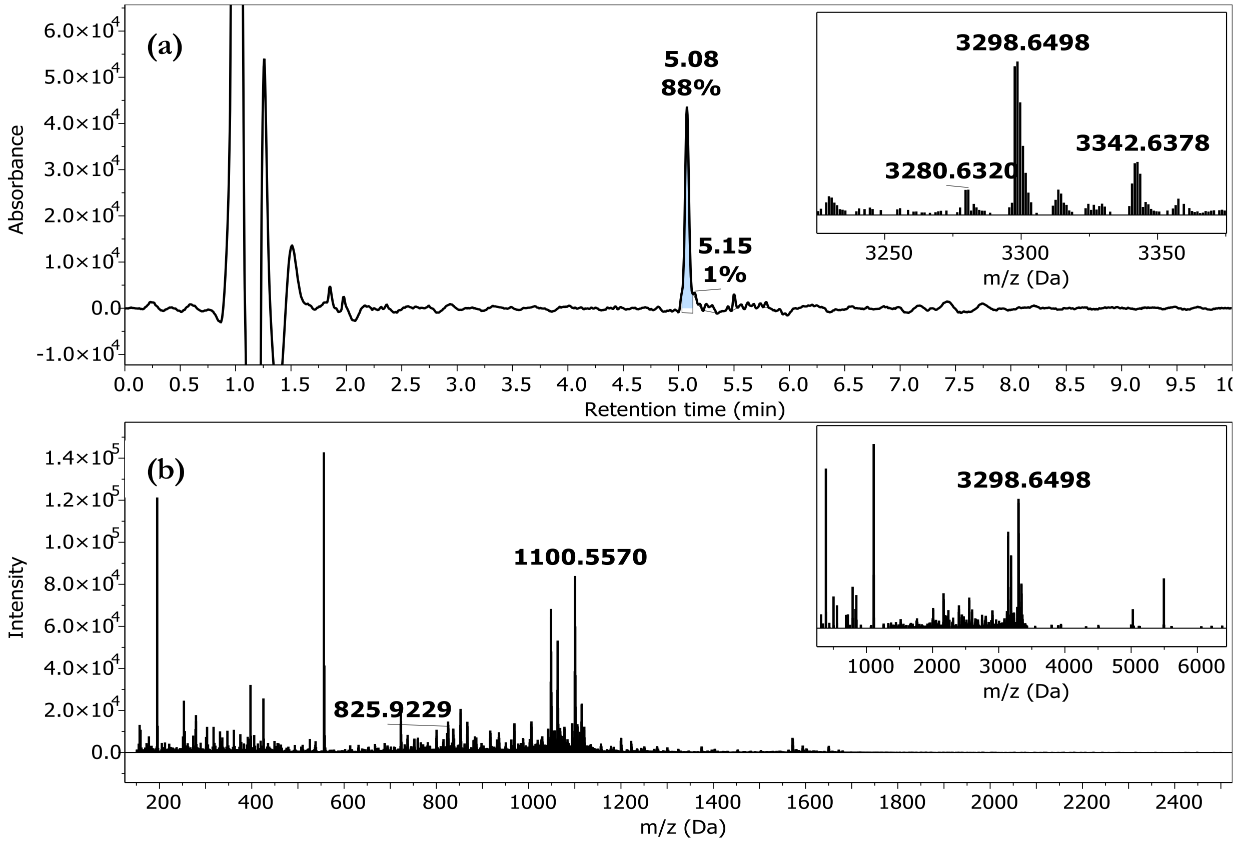

SI Figure 165. LC-MS Profile of crude GLP-1. (**a**) Absorbance chromatogram (λ = 214 nm) of peptide GLP-1; Rt 5.08 min, 88% purity. (**b**) ESI-TOF spectrum found within Rt 2.5–8 min (insert: deconvoluted masses); Monoisotopic mass (ESI+) calcd. for $C_{149}H_{226}N_{40}O_{45}$ 3295.6626, found 3295.6530. LCMS Gradient A (**Section 2.7**).

6.1.5 GLP-1: Novabiochem NovaPEG resin (no linker)

```
                   10                 20                     30
H2N-HAEGTFTSDV  SSYLEGQAAK   EFIAWLVKGR-CONH2   (30 AA)
```

The peptide GLP-1 was synthesized on commercially available Novabiochem NovaPEG resin (0.48 mmol/g, 52 mg, 25 μmol) using the standard AFPS protocol (**Section 2.2.1**, 20 mL/min flowrate) (**SI Figure 166**). Total synthesis time to afford resin-bound GLP-1 was approximately 1.5 h.

UV-Vis synthesis trace

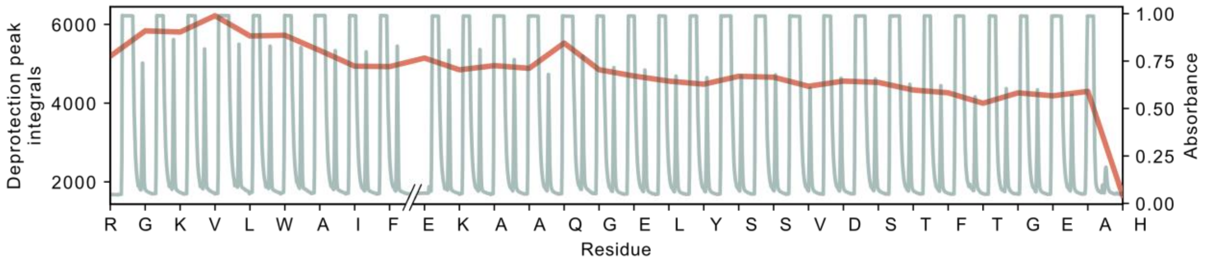

SI Figure 166. UV trace (λ = 310 nm) from AFPS of GLP-1 (green) and deprotection peak integrals (red), synthesized on Novabiochem® NovaPEG resin. The synthesis was paused after the 9th coupling [Phe] for approximately 10 min.

6.1.6 Comparison of impurities

Impurities detected and identified via LCMS for each synthesis are given in the following table. No data was obtained for the NovaPEG resin and for the MeDbz linker.

Linker	Guan. truncation before A2	W deletion	I/L deletion	V deletion	T deletion	Y deletion	H deletion	S deletion	Q/K deletion	A deletion	Q/K deletion	Formyl truncation before H1
Rink linker	4 %	4 %	1 %	1 %	0 %	<1%	2 %	1 %	0 %	0 %	0 %	2 %
HMPB linker	3 %	3 %	1 %	2 %	2 %	1 %	3 %	2 %	2 %	1 %	2 %	0 %
Photo-linker	2 %	2 %	2 %	2 %	0 %	4 %	1 %	2 %	0 %	0 %	0 %	3 %

6.2 Evaluation of cleavable linkers: Barstar[75–90]

6.2.1 Novabiochem® NovaPEG Rink Amide resin

See **Section 3.1.1**.

6.2.2 Barstar[75–90]: HMPB ChemMatrix® resin

```
                   10            16
H2N-FREAKAEGCD   ITIILS-COOH   (16 AA)
```

HMPB ChemMatrix® resin (0.62 mmol/g, 79 mg, 49 µmol) was manually coupled with Fmoc-Arg(Pbf)-OH using the protocol outline in **Section 2.3**. The loading of the resin was determined using the Fmoc-loading test described in **Section 2.4**, which resulted in a loading of 0.62 mmol/g. The peptide Barstar[75–90] was then prepared using the standard AFPS protocol (**Section 2.2.1**, 20 mL/min flowrate). Total synthesis time to afford resin-bound Barstar[75–90] was approximately 45 min. Cleavage of the peptidyl-resin (27 mg, approx. 17 µmol) according to Cleavage Protocol A (**Section 2.5.1**) afforded the crude peptide (1.4 mg, 27% purity by LCMS, 19% purity by UHPLC).

UV-Vis synthesis trace

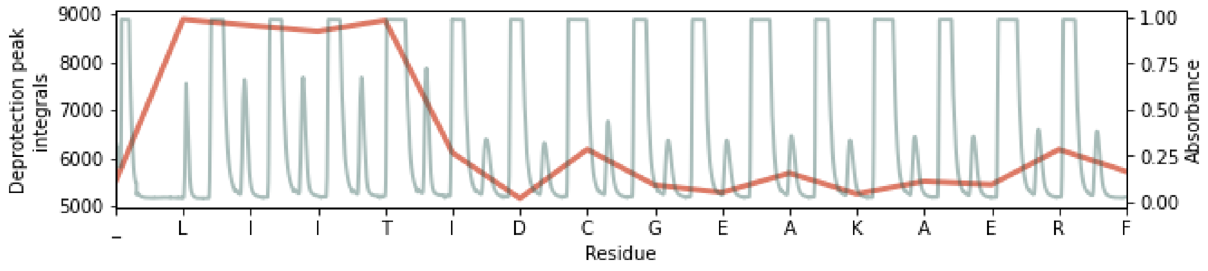

SI Figure 167. UV trace (λ = 310 nm) from AFPS of Barstar[75–90] (green) and deprotection peak integrals (red), synthesized on HMPB ChemMatrix® resin.

LC-MS of crude Barstar[75–90]

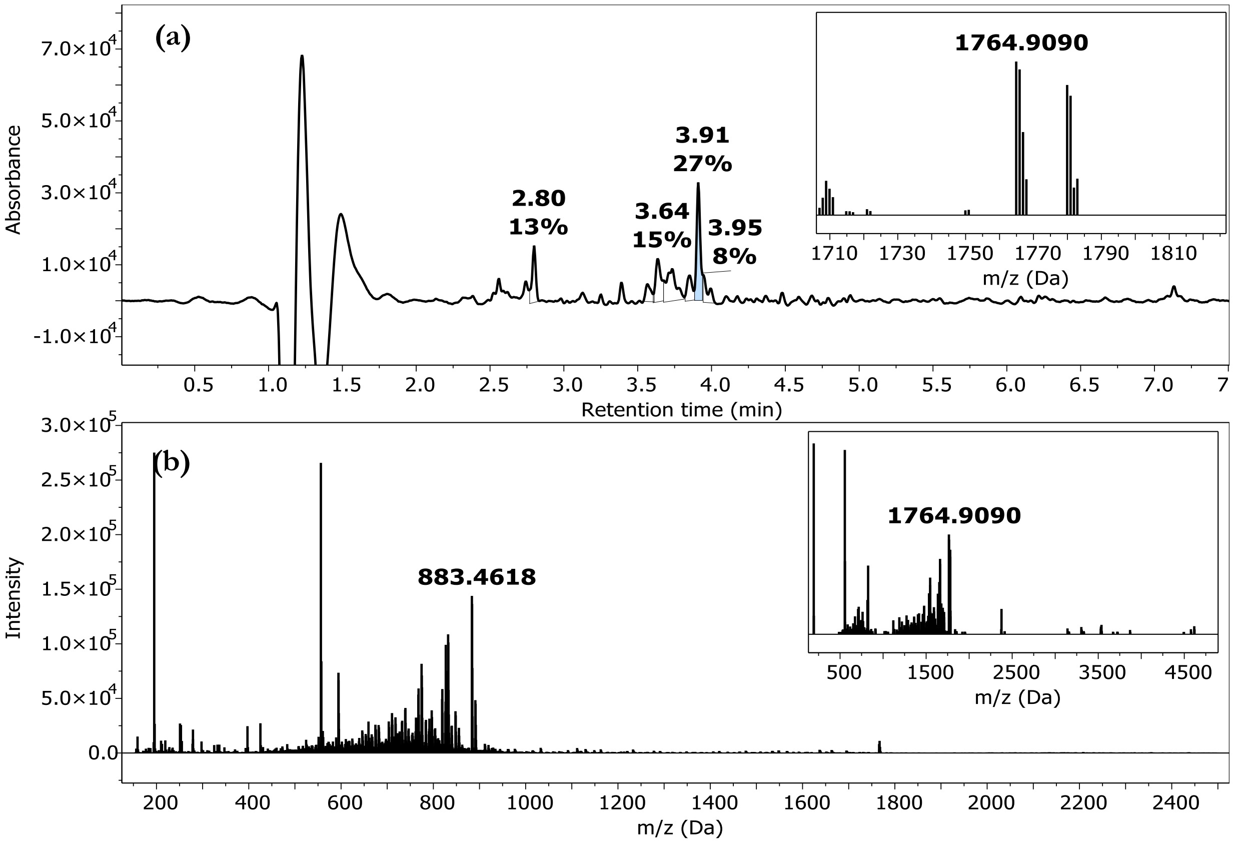

SI Figure 168. LC-MS Profile of crude peptide Barstar[75–90] synthesized on HMPB ChemMatrix® resin. (**a**) Absorbance chromatogram (λ = 214 nm) of Barstar[75–90]; Rt 3.91 min, 27% purity. (**b**) ESI-TOF spectrum found within Rt 2–9 min (insert: deconvoluted masses); Monoisotopic mass (ESI+) calcd. for $C_{77}H_{128}N_{20}O_{25}S$ 1764.9080, found 1764.9090. LCMS Gradient A (**Section 2.7**).

UHPLC of crude Barstar[75–90]

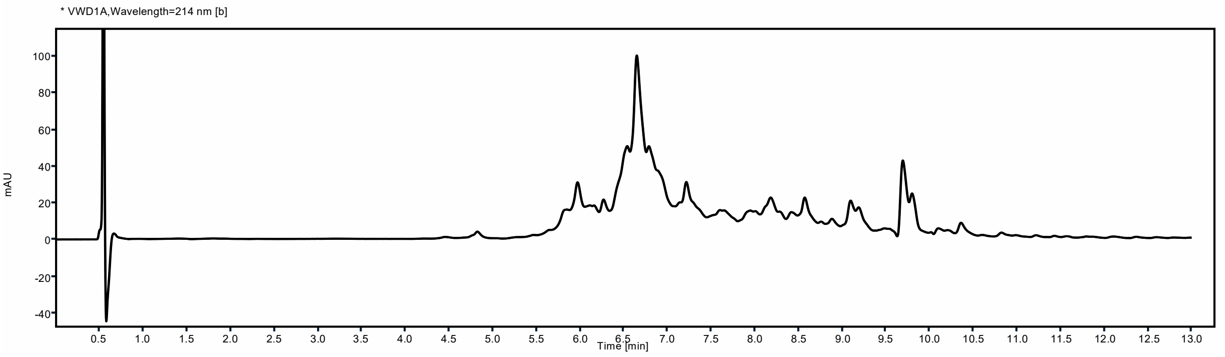

SI Figure 169. UHPLC profile of crude Barstar[75–90] synthesized on HMPB ChemMatrix® resin. Rt 6.65 min (Agilent Zorbax 300SB-C18 column, 5 µm, 2.1 × 150 mm, 5–95% MeCN over 10 min, ca. 9%B/min), 19% purity based on Area Under Curve (AUC) at λ = 214 nm.

6.2.3 Barstar[75–90]: Photolinker on Novabiochem® NovaPEG resin

```
                    10           16
H2N-FREAKAEGCD    ITIILS-CONH2    (16 AA)
```

The peptide Barstar[75–90] synthesized on the resin functionalized with the photolinker as described in **Section 2.3.3** (0.39 mmol/g, 61 mg, 24 µmol) using the standard AFPS protocol (**Section 2.2.1**, 20 mL/min flowrate) (**SI Figure 170**). Total synthesis time to afford resin-bound Barstar[75–90] was approximately 45 min. Cleavage of the peptidyl-resin (17 mg, 6.7 µmol) according to Cleavage Protocol B (**Section 2.5.2**) afforded the crude peptide (37% purity by LCMS [**SI Figure 171**]).

UV-Vis synthesis trace

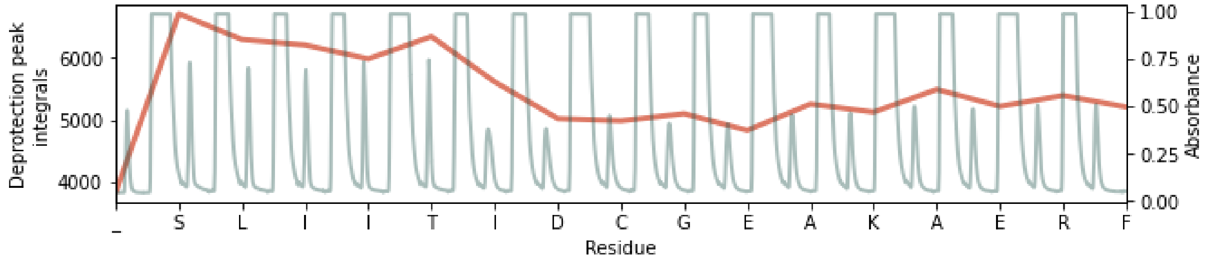

SI Figure 170. UV trace (λ = 310 nm) from AFPS of Barstar[75–90] (green) and deprotection peak integrals (red), synthesized on Photolinker on Novabiochem® NovaPEG resin.

LC-MS of crude Barstar[75–90]

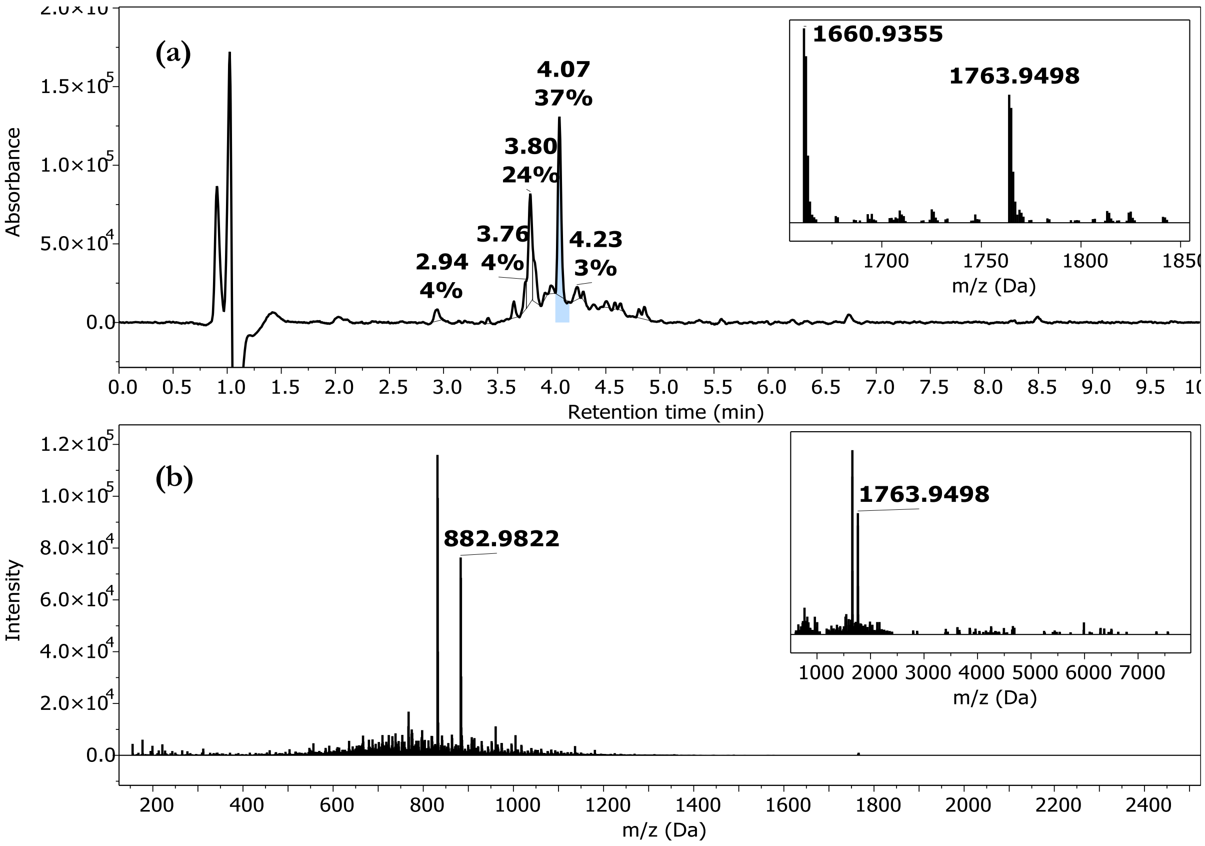

SI Figure 171. LCMS Profile of crude Barstar[75–90] synthesized on Novabiochem resin functionalized with the Photolinker. (**a**) Absorbance chromatogram (λ = 214 nm) of peptide Barstar[75–90]; Rt 4.07 min, 37% purity. (**b**) ESI-TOF spectrum found within

Rt 2–9 min (insert: deconvoluted masses); Monoisotopic mass (ESI+) calcd. for $C_{77}H_{129}N_{21}O_{24}S$ 1763.9240, found 1763.9496. LCMS Gradient A (**Section 2.7**).

6.2.4 Barstar[75–90]: MeDbz linker on Novabiochem® NovaPEG resin

```
               10         16
H2N-FREAKAEGCD   ITIILS-COOH   (16 AA)
```

The peptide Barstar[75–90] was synthesized on the manually MeDbz-grafted resin as described in **Section 2.3.4** (0.39 mmol/g, 0.10 g, 40 µmol) using the standard AFPS protocol (**Section 2.2.1**, 20 mL/min flowrate) (**SI Figure 172**). Total synthesis time to afford resin-bound Barstar[75–90] was approximately 45 minutes. Cleavage of the peptidyl-resin (8.7 mg, approx. 4.0 µmol) according to Cleavage Protocol C (**Section 2.5.3**) afforded the crude peptide (6.8 mg, 38% purity by LCMS [**SI Figure 173**]). Two other procedures inspired from the literature were also attempted,(*57*, *58*) but did not yield any of the expected product.

UV-Vis synthesis trace

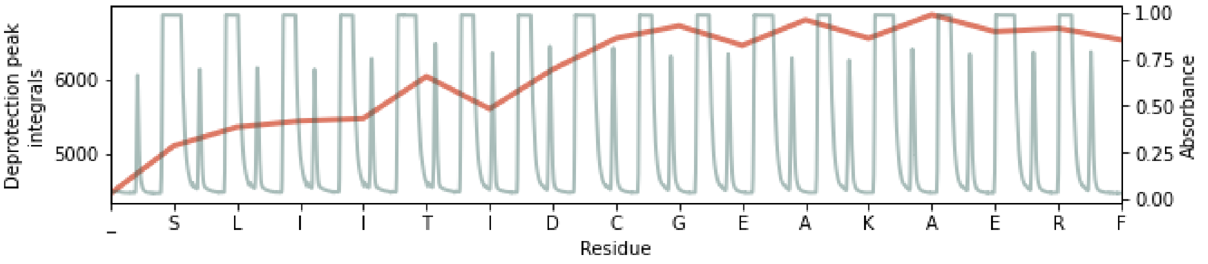

SI Figure 172. UV trace (λ = 310 nm) from AFPS of Barstar[75–90] (green) and deprotection peak integrals (red), synthesized on MeDbz linker on Novabiochem® NovaPEG resin.

LC-MS of crude Barstar[75–90]

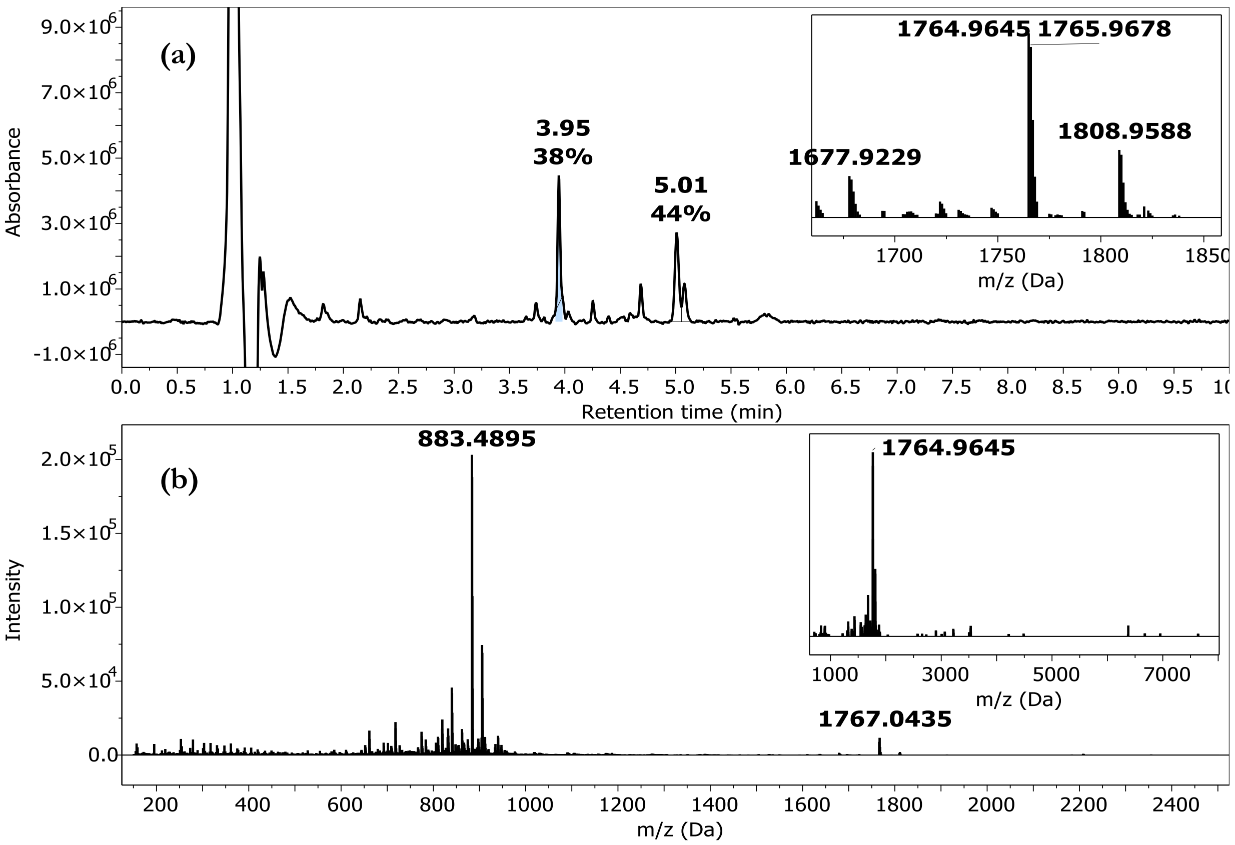

SI Figure 173. LC-MS Profile of crude peptide Barstar[75–90]. (**a**) Absorbance chromatogram (λ = 214 nm) of peptide Barstar[75–90]; Rt 3.95 min, 38% purity. (**b**) ESI-TOF spectrum found within Rt 2–9 min (insert: deconvoluted masses); Monoisotopic mass (ESI+) calcd. for $C_{77}H_{128}N_{20}O_{25}S$ 1764.9080, found 1764.9645. LCMS Gradient A (**Section 2.7**).

6.2.5 Barstar[75–90]: Rink amide and Photolinker on Novabiochem® NovaPEG resin

```
                    10        16
H2N-FREAKAEGCD  ITIILS-CONH2   (16 AA)
```

Fmoc-Photolinker-OH (0.13 g, 0.24 mmol) was coupled on Novabiochem® NovaPEG resin (0.30 g, 0.12 mmol) resin using the protocol outline in **Section 2.3.3**. Then, Fmoc-Rink-OH (0.13 g, 0.24 mmol) was coupled to the functionalized resin using the same protocol. The loading of the resin was determined using the Fmoc-loading test described in **Section 2.4**, which resulted in a loading of 0.16 mmol/g. The peptide Barstar[75–90] was then synthesized on the Fmoc-Rink-Photolinker grafted resin (50 mg, 8.0 μmol) using the standard AFPS protocol (**Section 2.2.1**, 40 mL/min flowrate) (**SI Figure 174**). Total synthesis time to afford resin-bound Barstar[75–90] was approximately 45 minutes. Cleavage of the peptidyl-resin (10 mg, approx. 1.3 μmol) according to Cleavage Protocol A (**Section 2.5.1**) afforded the crude peptide (1.6 mg, 8% purity by LCMS [**SI Figure 175**], N/A by UHPLC [**SI Figure 176**]).

UV-Vis synthesis trace

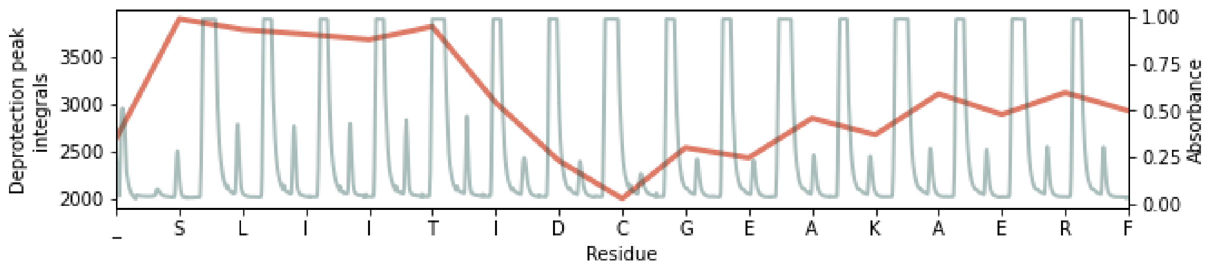

SI Figure 174. UV trace (λ = 310 nm) from AFPS of Barstar[75–90] (green) and deprotection peak integrals (red), synthesized on Rink amide linker coupled to photolinker on Novabiochem® NovaPEG resin.

LC-MS of crude Barstar[75–90]

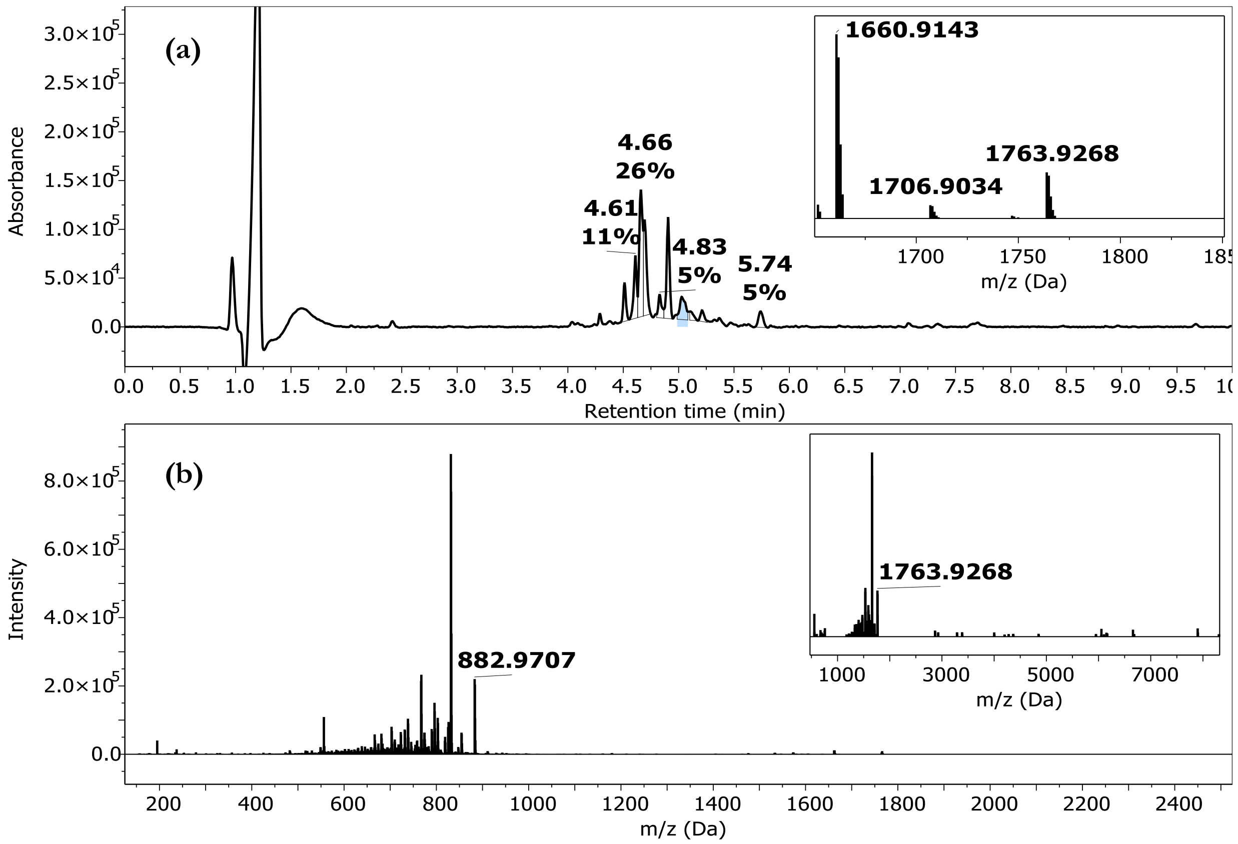

SI Figure 175. LCMS Profile of Barstar[75–90]. (**a**) Absorbance chromatogram (λ = 214 nm) Barstar[75–90]; Rt 5.03 min, 8% purity. (**b**) ESI-TOF spectrum found within Rt 2–9 min (insert: deconvoluted masses); Monoisotopic mass (ESI+) calcd. For $C_{77}H_{129}N_{21}O_{24}S$ 1763.9240, found 1763.9268. LCMS Gradient A (**Section 2.7**).

UHPLC of crude Barstar[75–90]

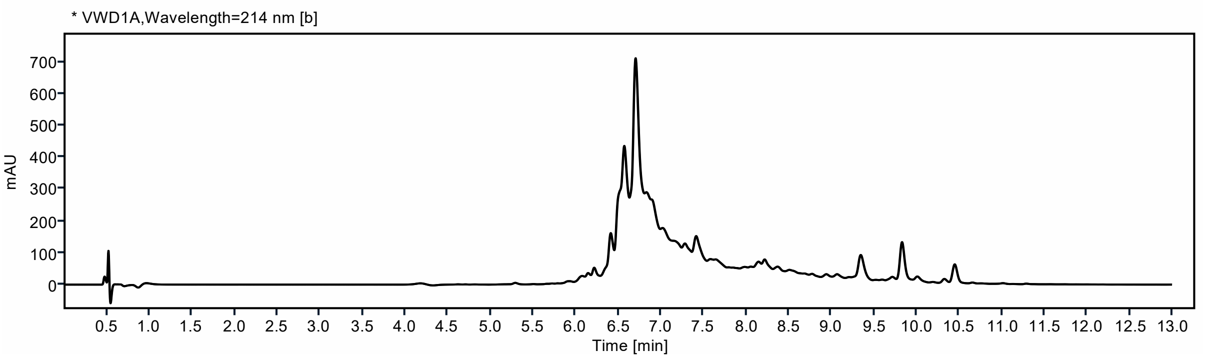

SI Figure 176. UHPLC profile of Barstar[75–90] synthesized on Rink amide-Photolinker on Novabiochem® NovaPEG resin. Purity N/A. (Agilent Zorbax 300SB-C18 column, 5 µm, 2.1 × 150 mm, 5–95% MeCN over 10 min, ca. 9%B/min).

6.2.6 Barstar[75–90]-[Arg(Pbf)]$_6$ Rink amide and Photolinker on Novabiochem® NovaPEG resin

```
                    10                  20
H2N-FREAKAEGCD  ITIILSRRRR RR-CONH2          (22 AA)
```

Fmoc-Photolinker-OH (0.13 mg, 0.24 mmol) was coupled on Novabiochem® NovaPEG resin (0.30 g, 0.12 mmol) using the protocol outline in **Section 2.3.3**. Then, Fmoc-Rink-OH (0.13 mg, 0.24 mmol) was coupled to the functionalized resin using the same protocol. The loading of the resin was determined using the Fmoc-loading test described in **Section 2.4**, which resulted in a loading of 0.16 mmol/g. The peptide Barstar[75–90]-[Arg(Pbf)]$_6$ was then synthesized on the Fmoc-Rink-Photolinker grafted resin (56 mg, approx. 8.9 µmol) using the standard AFPS protocol (**Section 2.2.1**, 40 mL/min flowrate) (**SI Figure 177**). Total synthesis time to afford resin-bound Barstar[75–90]-[Arg(Pbf)]$_6$ was approximately 1 hour. Cleavage of the peptidyl-resin (14 mg, approx. 1.2 µmol) according to Cleavage Protocol A (**Section 2.5.1**) afforded the crude peptide (0.70 mg, 62% purity by LCMS [**SI Figure 178**], 62% by UHPLC [**SI Figure 179**]).

UV-Vis synthesis trace

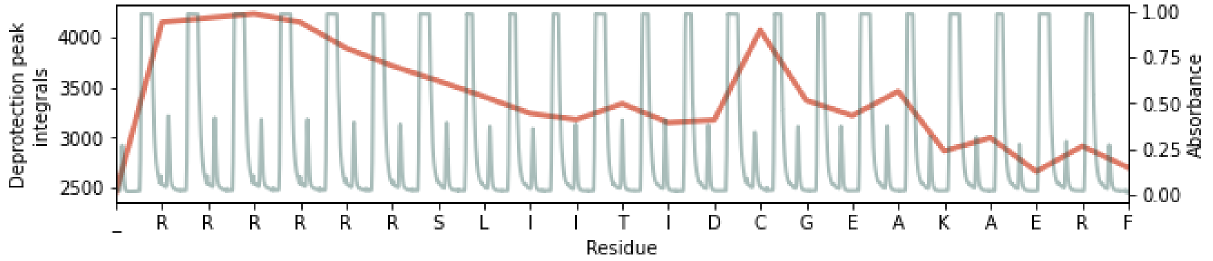

SI Figure 177. UV trace (λ = 310 nm) from AFPS of Barstar[75–90]-[Arg(Pbf)]$_6$ (green) and deprotection peak integrals (red), synthesized on Rink amide linker coupled to Photolinker on Novabiochem® NovaPEG resin.

LC-MS of crude Barstar[75–90]-(Arg)$_6$

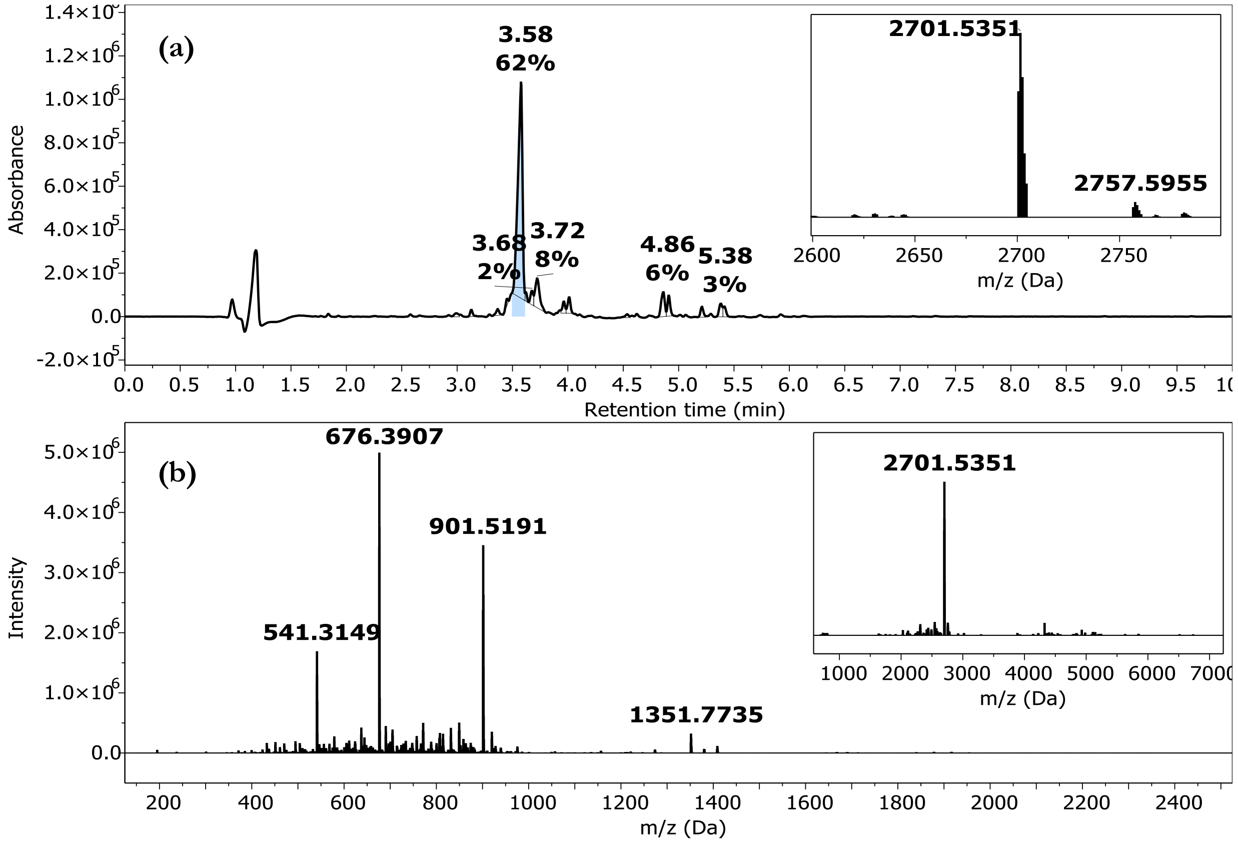

SI Figure 178. LCMS Profile of Barstar[75–90] bearing (Arg)$_6$ synthesized on Rink-Photolinker on Novabiochem® NovaPEG resin. (**a**) Absorbance chromatogram (λ = 214 nm) of Barstar[75–90]-Arg$_6$; Rt 3.58 min, 62% purity. (**b**) ESI-TOF spectrum found within Rt 2–9 min (insert: deconvoluted masses); Monoisotopic mass (ESI+) calcd. for $C_{113}H_{200}N_{44}O_{31}S$ 2700.5307, found 2700.5332. LCMS Gradient A (**Section 2.7**).

UHPLC of crude Barstar[75–90]-(Arg)$_6$

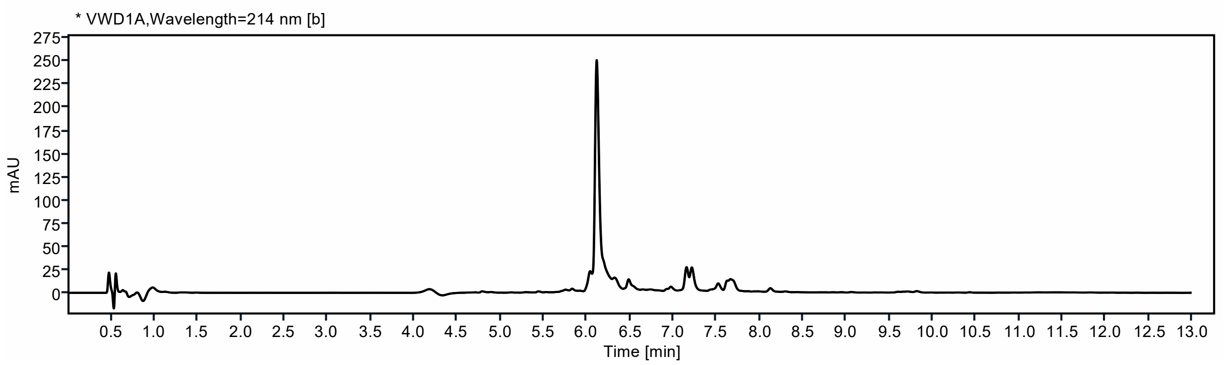

SI Figure 179. UHPLC profile of crude Barstar[75–90] bearing (Arg)$_6$ synthesized on Rink-Photolinker on Novabiochem® NovaPEG resin. Rt 6.11 min (Agilent Zorbax 300SB-C18 column, 5 µm, 2.1 × 150 mm, 5–95% MeCN over 10 min, ca. 9%B/min), 62% purity based on Area Under Curve (AUC) at λ = 214 nm.

6.2.7 Cumulative synthesis traces of Barstar[75–90] synthesized on Rink-Photolinker (double linker) on Novabiochem® NovaPEG resin

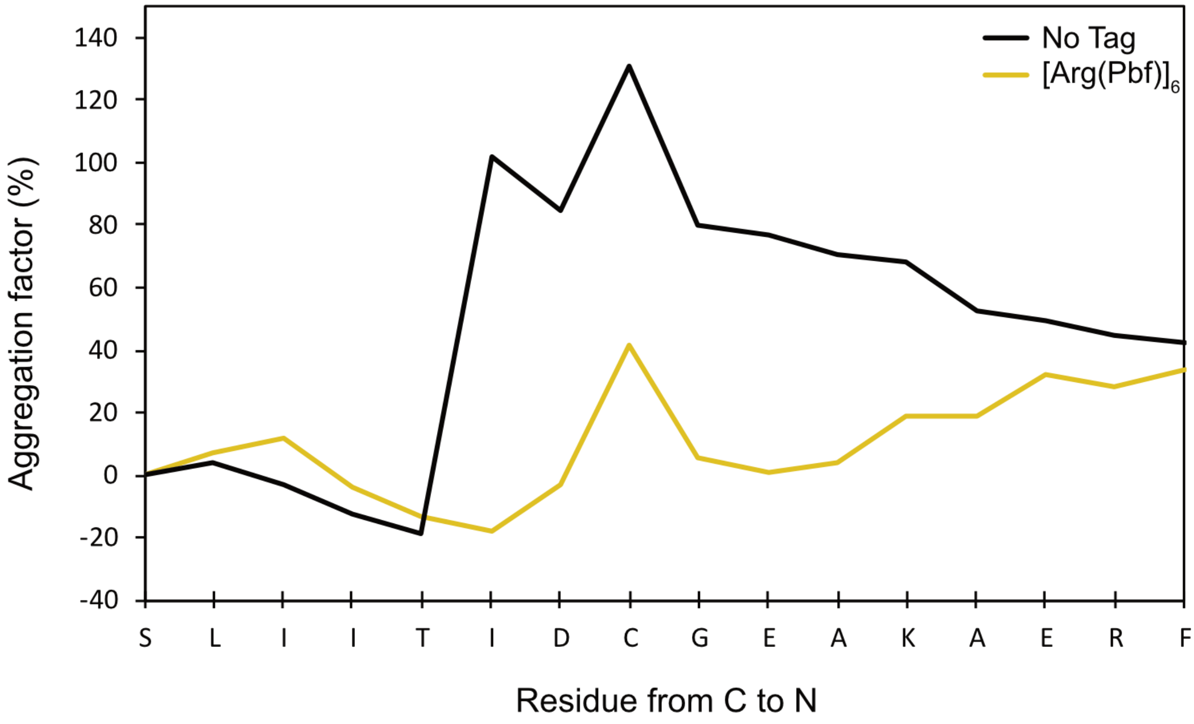

SI Figure 180. Aggregation as a function of Fmoc-deprotection peak broadening by in-line UV-Vis (310 nm) in flow-SPPS for Barstar[75–90] with and without Arg(Pbf)]$_6$ tag, synthesized on photolinker resin grafted with Rink amide linker, normalized at Ser[90].

6.2.8 Barstar[75–90]: Rink amide linker on HMPB ChemMatrix® resin

```
                10          16
H2N-FREAKAEGCD ITIILS-CONH2       (16 AA)
```

Fmoc-Rink-OH (0.67 g, 1.2 mmol) was coupled to HMPB ChemMatrix® resin (0.62 mmol/g, 0.20 g, 0.12 mmol) using the protocol outline in **Section 2.3.2**. The loading of the resin was determined using the Fmoc-loading test described in **Section 2.4**, which resulted in a loading of 0.18 mmol/g. The peptide Barstar[75–90]-[Arg(Pbf)]$_6$ was then synthesized on the Fmoc-Rink-Photolinker grafted resin (69 mg, approx. 12 µmol) using the standard AFPS protocol (**Section 2.2.1**, 40 mL/min flowrate) (**SI Figure 181**). Total synthesis time to afford resin-bound Barstar[75–90] was approximately 45 minutes. Cleavage of the peptidyl-resin (9.8 mg, approx. 1.1 µmol) according to Cleavage Protocol A (**Section 2.5.1**) afforded the crude peptide (1.4 mg, 2% purity by LCMS [**SI Figure 182**], N/A by UHPLC [**SI Figure 183**]).

UV-Vis synthesis trace

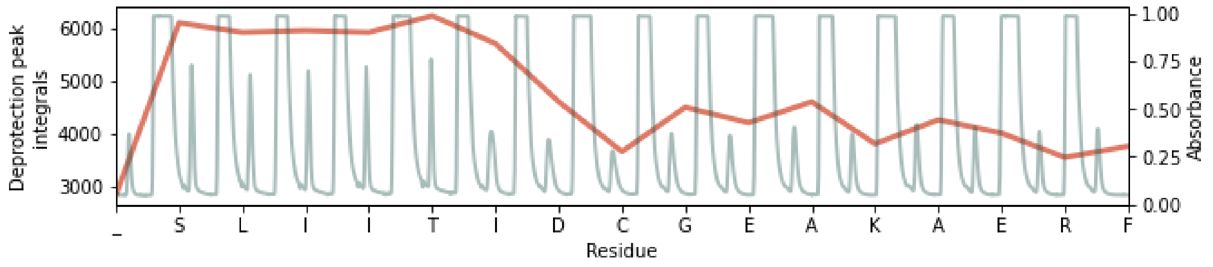

SI Figure 181. UV trace (λ = 310 nm) from AFPS of Barstar[75–90] (green) and deprotection peak integrals (red), synthesized on Rink amide linker coupled to HMPB ChemMatrix® resin.

LC-MS of crude Barstar[75–90]

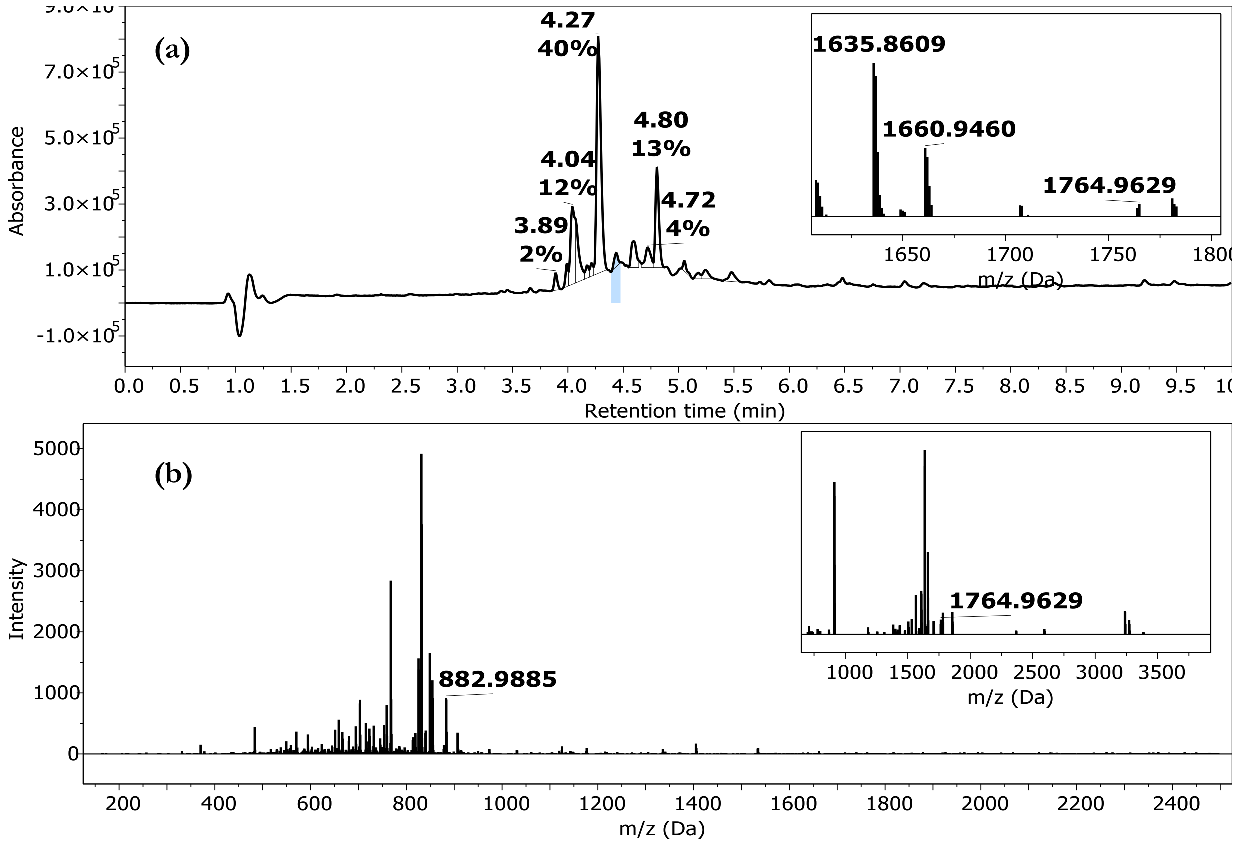

SI Figure 182. LCMS Profile of Barstar[75–90]. (**a**) Absorbance chromatogram (λ = 214 nm) Barstar[75–90] ; Rt 4.44 min, 2% purity. (**b**) ESI-TOF spectrum found within Rt 2–9 min (insert: deconvoluted masses); Monoisotopic mass (ESI+) calcd. for $C_{77}H_{129}N_{21}O_{24}S$ 1763.9240, found 1763.9268. LCMS Gradient A (**Section 2.7**).

UHPLC of crude Barstar[75–90]

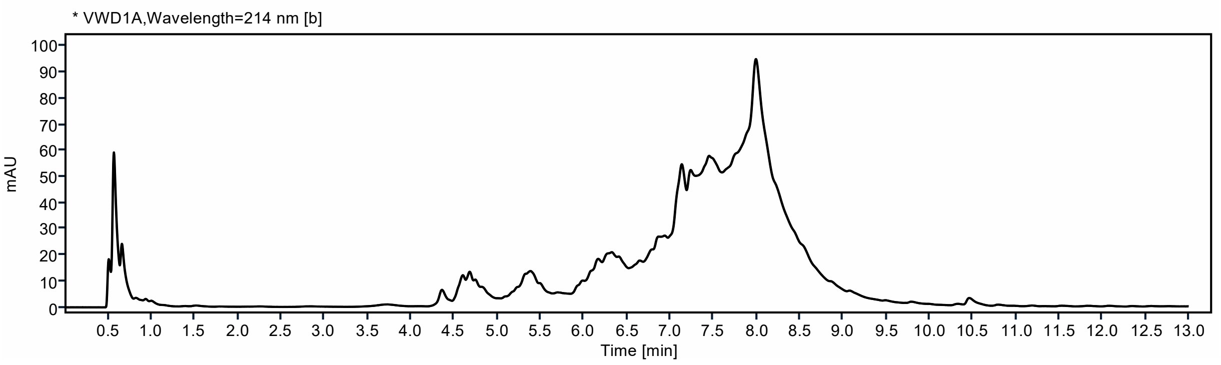

SI Figure 183. UHPLC profile of crude Barstar[75–90] synthesized on Rink amide-HMPB ChemMatrix resin. Purity N/A. (Agilent Zorbax 300SB-C18 column, 5 µm, 2.1 × 150 mm, 5–95% MeCN over 10 min, ca. 9%B/min).

6.2.9 Barstar[75–90]: Novabiochem® NovaPEG resin (no linker)

```
           10          16
H2N-FREAKAEGCD ITIILS-CONH2      (16 AA)
```

The peptide Barstar[75–90] was synthesized on commercially Novabiochem NovaPEG resin (0.48 mmol/g, 31 mg, 15 µmol) using the standard AFPS protocol (**Section 2.2.1**, 40 mL/min flowrate) (**SI Figure 184**). Total synthesis time to afford resin-bound Barstar[75–90] was approximately 45 min.

UV-Vis synthesis trace

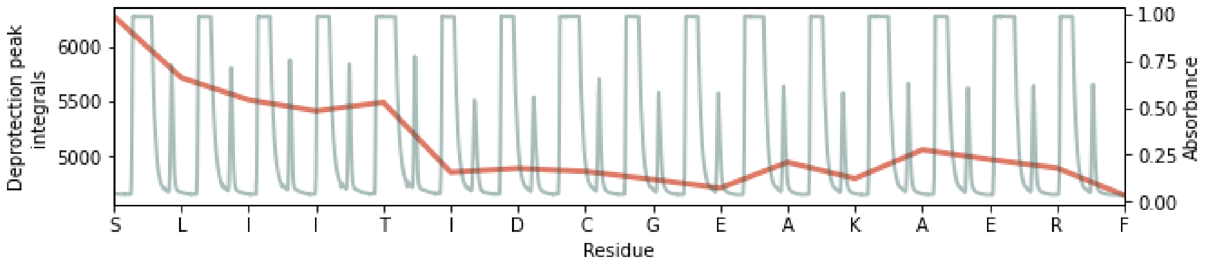

SI Figure 184. UV trace (λ = 310 nm) from AFPS of Barstar[75–90] (green) and deprotection peak integrals (red), synthesized on Novabiochem® NovaPEG resin.

6.2.10 Barstar[75–90]-Photolinker-[Arg(Pbf)]$_6$ on Novabiochem® NovaPEG Rink Amide resin

```
           10                            23
H2N-FREAKAEGCD   ITIILS-Photolinker   -RRRRRR-CONH2        (23 AA)
```

The peptide [Arg(Pbf)]$_6$ was synthesized on Novabiochem® NovaPEG Rink Amide resin (0.41 mmol/g, 0.18 mg, 74 µmol) using the standard AFPS protocol (**Section 2.2.1**, 20 mL/min flowrate). Resin bound [Arg(Pbf)]$_6$-Rink amide (0.16 mmol/g, 0.12 mg, 19 µmol) was functionalized with the photolinker as described in **Section 2.3.3** and the peptide Barstar[75–90] was synthesized using the standard AFPS protocol (**Section 2.2.1**, 20 mL/min flowrate) (**SI Figure 185**). Total synthesis time to afford resin-bound Barstar[75–90] was approximately 45 min. Resin was not cleaved.

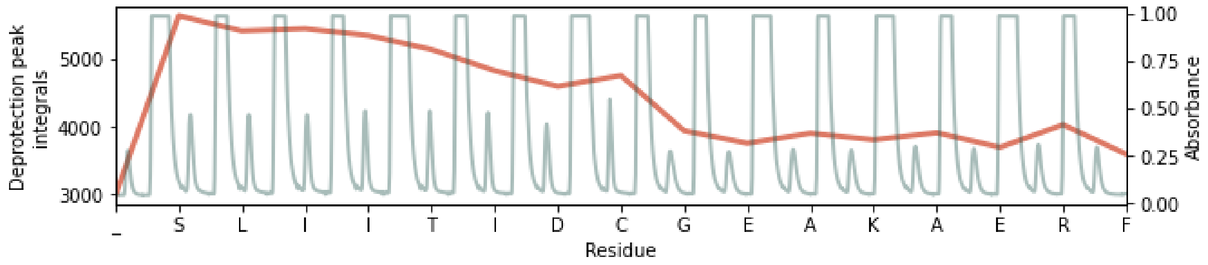

SI Figure 185. UV trace (λ = 310 nm) from AFPS of Barstar[75–90] (green) and deprotection peak integrals (red), synthesized on Photolinker-[Arg(Pbf)]$_6$-Rink amide Novabiochem® NovaPEG resin.

6.2.11 Comparison of Barstar[75–90] Photolinker-[Arg(Pbf)]$_6$ with [Arg(Pbf)]$_6$

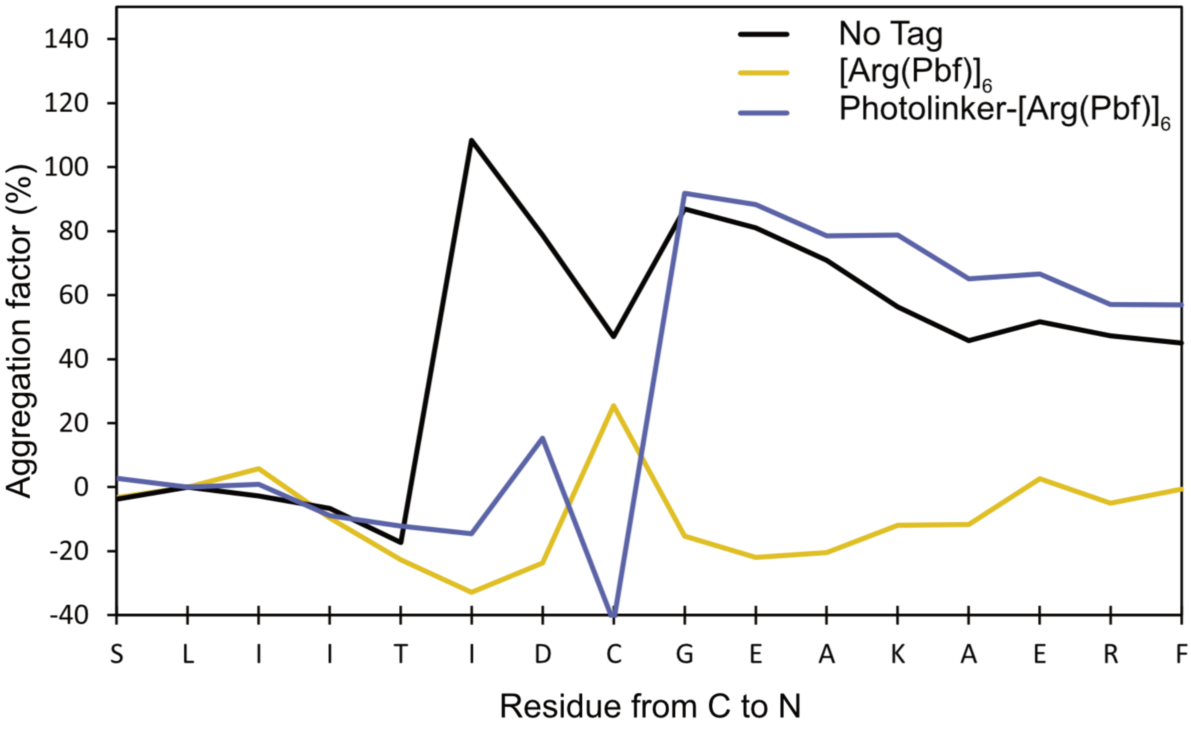

SI Figure 186. Aggregation as a function of Fmoc-deprotection peak broadening by in-line UV-Vis (310 nm) in flow-SPPS for Barstar[75–90] with and without [Arg(Pbf)]$_6$ tag, or with a Photolinker-[Arg(Pbf)]$_6$ tag, synthesized on Novabiochem® NovaPEG Rink Amide resin, normalized at Leu[89].

6.2.12 Comparison of impurities

Impurities detected and identified via LCMS for each synthesis of Barstar[75–90] on different linkers are given in the following table. No data was obtained for the NovaPEG resin and the MeDbz linker.

Linker	I/L deletion	I/L deletion	Aspartimide	T deletion	C deletion	F deletion	E deletion	D deletion	K deletion	Double G
Rink linker	1 %	3 %	0 %	3 %	13 %	2 %	5 %	2 %	1 %	2 %
HMPB linker	<1%	3 %	0 %	4 %	1 %	0 %	<1%	5 %	0 %	6 %
Photo-linker	<1%	0 %	3 %	5 %	24 %	2 %	2 %	3 %	1 %	5 %

6.3 Recapitulative table of crude purities for test peptides determined by UHPLC

Linker System	Barstar[75–90]	GLP-1
**Rink**	63%	80%
**HMPB**	19%	66%
**Photolinker***	37%	60%
**MeDbz***	38%	88%
**Rink-Photolinker**	N/A	-
**Arg_6-Rink-Photolinker**	62%	-
**Rink-HMPB**	N/A	-

*Crude purity determined by LCMS

7 SynTag – combining [$Arg(Pbf)_6$] and MeDbz linker into a versatile synthesis tag

7.1 Preparation of MeDbz-[$Arg(Pbf)_6$]-Rink amide resin

```
MeDbz-RRRRRR-CONH2        (7 AA)
```

The peptide [Arg(Pbf)]$_6$ was synthesized on Novabiochem® NovaPEG Rink Amide resin (0.20 mmol/g, 0.25 mg, 50 μmol) using the standard AFPS protocol (**Section 2.2.1**, 20 mL/min flowrate). The resin was then collected and washed with DMF (3 × 5 mL). To a solution of Fmoc-MeDbz-OH (97 mg, 0.25 mmol) in DMF (3.0 mL) was added HATU (0.38 M in DMF, 0.63 mL) and DIPEA (85 μL, 0.50 mmol), and the solution was agitated gently for 30 s. The active ester solution was then added to the resin-bound [Arg(Pbf)]$_6$ and left to stand at 23 °C for 2 h, with occasional stirring. The resin was then drained and washed with DMF (3 × 5 mL). The Fmoc-group was then removed by treating the resin with a solution of 20% piperidine in DMF (*v*/*v*) (5.0 mL, 3 × 5 min). The resin was drained, washed with DMF (3 × 5 mL) and DCM (3 × 5 mL), then dried under reduced pressure. Final mass of the resin was 0.45 g (containing approx. 50 μmol peptide).

7.2 Barstar[75–90]-SynTag

```
           10                     23
H2N-FREAKAEGCD  ITIILS-MeNbz  -RRRRRR-CONH2   (23 AA)
```

Resin-bound MeDbz-[Arg(Pbf)]$_6$ (93 mg, 10 μmol) (see **Section 7.1**), was swelled in DMF (3 mL) for 10 min, then drained. To a solution of Fmoc-Ser(O*t*Bu)-OH (19 mg, 50 μmol) in DMF (1.0 mL) was added HATU (0.38 M in DMF, 0.10 mL) and DIPEA (13 μL, 0.10 mmol), and the solution was agitated gently for 30 s. The active ester solution was then added to the swelled resin, and left to stand at 23 °C for 1 h, with occasional stirring. The resin was then drained, washed with DMF (3 × 5 mL) and DCM (3 × 5 mL), then dried under reduced pressure. Residues 74–89 of the peptide Barstar[75–90] were then synthesized upon the Fmoc-Ser(O*t*Bu)-MeDbz-[Arg(Pbf)]$_6$ resin using the standard AFPS protocol (**Section 2.2.1**, 20 mL/min flowrate) (**SI Figure 187**). Boc-L-Phe-OH was coupled as the final amino acid using the standard AFPS protocol (HATU as activator), without an Fmoc-deprotection step. The resin was then washed with DMF (3 × 3 mL) and DCM (3 × 3 mL), then dried under reduced pressure.

UV-Vis synthesis trace

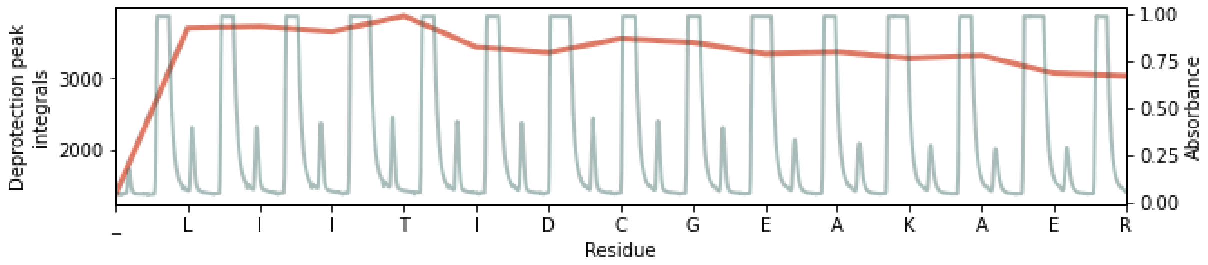

SI Figure 187. UV trace (λ = 310 nm) from AFPS of Barstar[75–90]-MeDbz-[Arg(Pbf)]$_6$ (green) and deprotection peak integrals (red).

Acylation and cyclisation of MeDbz to afford MeNbz.(43) The peptidyl-resin (15 mg, approx. 3.0 μmol) was swelled in DCM (1.0 mL) for 10 min, then drained. To the peptidyl-resin was added a solution of 4-nitrophenylchloroformate (4.9 mg, 24 μmol) in DCM (1.0 mL), and the reaction was allowed to proceed at 23 °C with occasional stirring for 1 h. The resin was then drained, and washed with DCM (3 × 3 mL) and DMF (3 × 3 mL). A solution of DIPEA (0.10 mL, 0.60 mmol) in DMF (1.0 mL) was then added to the resin affording a yellow mixture, and the reaction was allowed to proceed at 23 °C with occasional stirring for 30 min. The resin was then drained and washed with DMF (3 × 3 mL). The DIPEA reaction was repeated as necessary (7 × 30 min) until a yellow color no longer developed after 20 min reaction time. The resin was then washed with DCM (3 × 3 mL) and dried under reduced pressure. Cleavage of the peptidyl-resin (15 mg, approx. 3.0 μmol) according to Cleavage Protocol A (**Section 2.5.1**) afforded the crude peptide (1.5 mg, 50% purity by LCMS [**SI Figure 188**], 58% purity by UHPLC [**SI Figure 189**], monoisotopic mass calc. 2874.5736, found 2874.5747).

LCMS of crude Barstar[75–90]-MeNbz-(Arg)$_6$

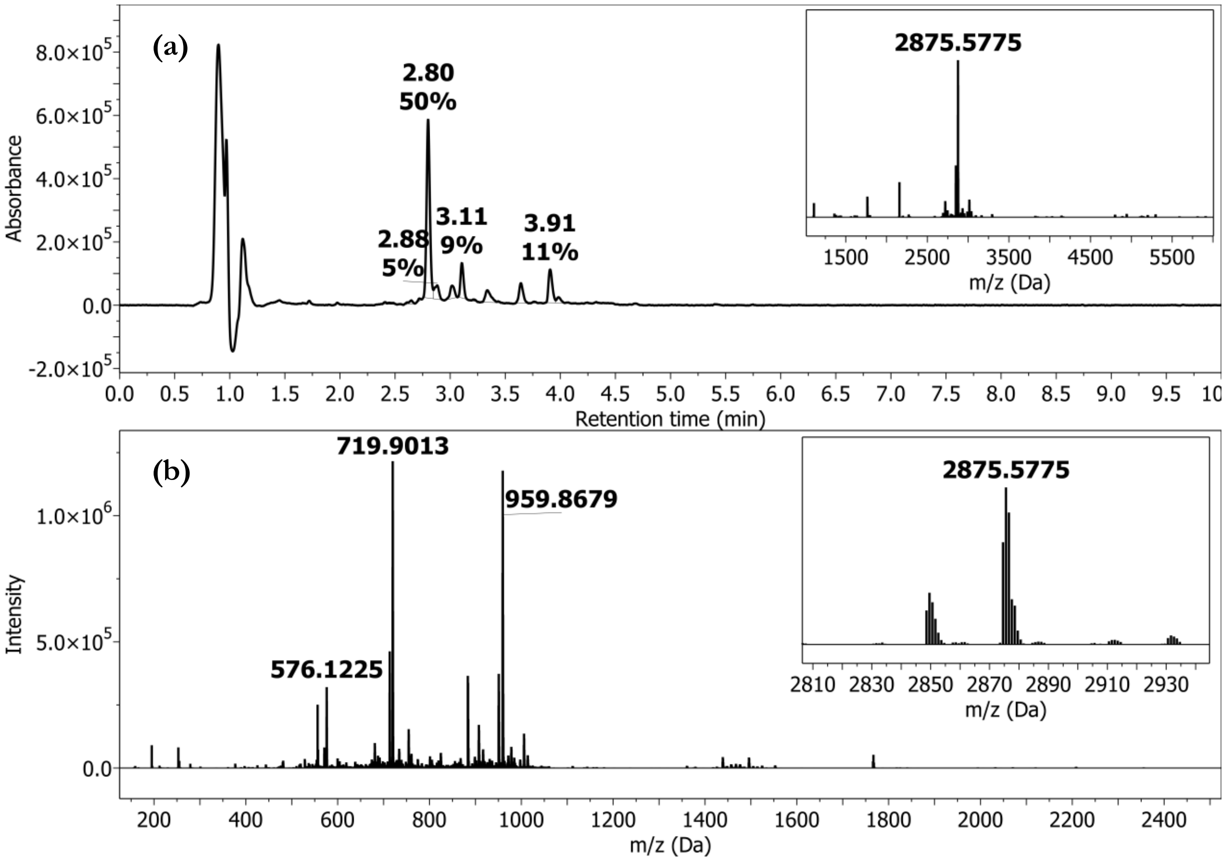

SI Figure 188. LCMS profile of crude Barstar[75–90]-MeNbz-(Arg)$_6$. (**a**) Absorbance chromatogram (λ = 214 nm); Rt 2.80 min, 50% purity. (**b**) ESI-TOF spectrum found within Rt 2–5 min. Inserts: deconvoluted masses. Monoisotopic mass (ESI+) calcd. for $C_{122}H_{207}N_{47}O_{32}S$ 2874.5736, found 2874.5747. LCMS Gradient A (**Section 2.7**).

UHPLC of crude Barstar[75–90]-MeNbz-(Arg)$_6$

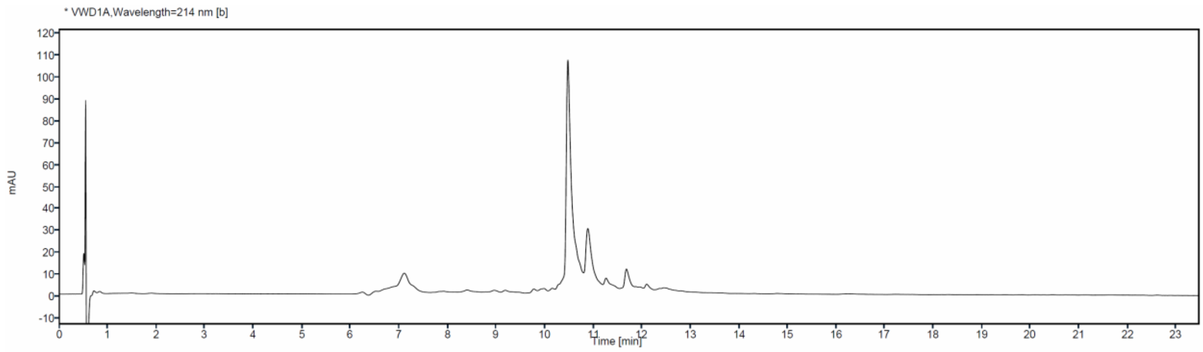

SI Figure 189. UHPLC profile of crude Barstar[75–90]-MeNbz-(Arg)$_6$. Rt 10.48 min. (Agilent Zorbax 300SB-C18 column, 5 µm, 2.1 × 150 mm, 5–95% MeCN over 20 min, *ca.* 4.5%B/min), 58% purity based on Area Under Curve (AUC) at λ = 214 nm.

7.2.1 Comparison of Barstar[75–90] synthesis using SynTag, [Arg(Pbf)]$_6$, or MeDbz

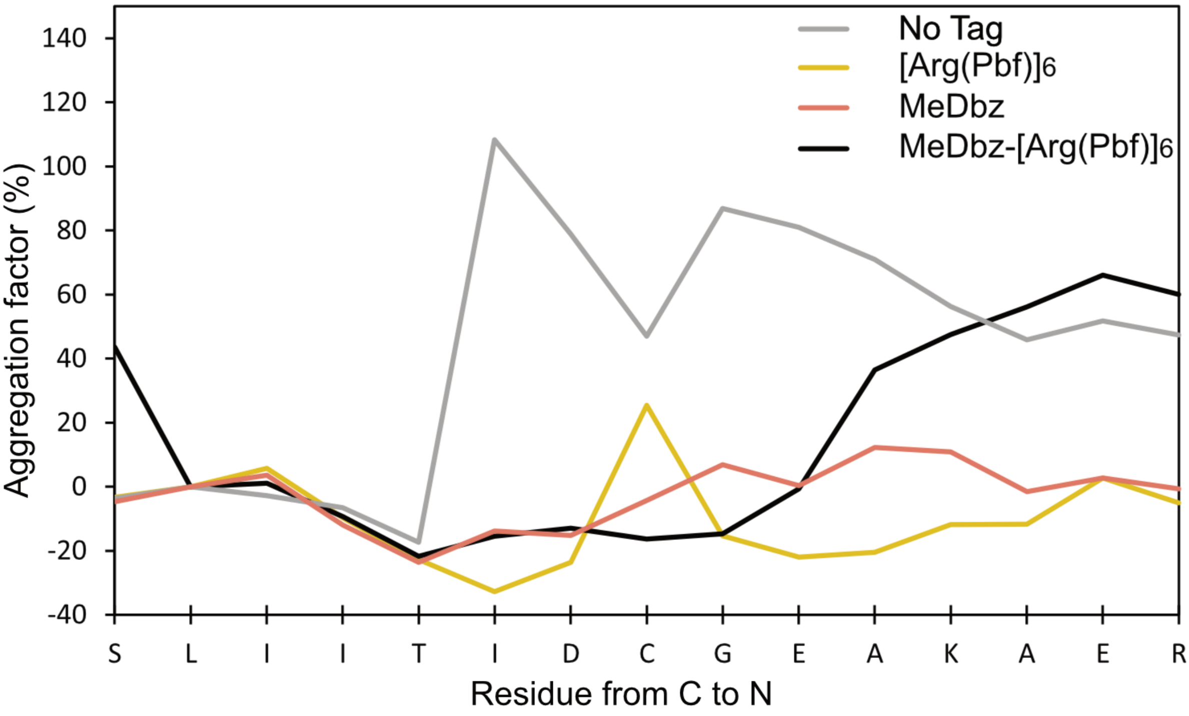

SI Figure 190. Aggregation as a function of Fmoc-deprotection peak broadening by in-line UV-Vis (310 nm) in flow-SPPS for Barstar[75–90] with [Arg(Pbf)]$_6$ tag, MeDbz linker, SynTag (MeDbz-[Arg(Pbf)]$_6$), or on Rink amide linker alone, normalized at Leu[89].

7.3 cAMP-dependent protein kinase inhibitor alpha (PKI-α)

```
                    10            20            30            40
H2N- MTDVETTYAD   FIASGRTGRR   NAIHDILVSS   ASGNSNELAL
                    50            60            70
     KLAGLDINKT   EGEEDAQRSS   TEQSGEAQGE  AAKSES-CONH2 (76 AA)
```

The protein PKI-α was synthesized on commercially available Novabiochem® NovaPEG Rink Amide resin (0.2 mmol/g, 100 mg, 20 µmol) using the standard AFPS protocol (**Section 2.2.1**, 20 mL/min flowrate) (**SI Figure 191**). Total synthesis time to afford resin-bound PKI-α was approximately 4 h. Cleavage of the peptidyl-resin (39 mg, approx. 3.6 µmol) according to Cleavage Protocol A (**Section 2.5.1**) afforded the crude peptide (8.6 mg, 11% purity by LCMS [**SI Figure 192**], 15% purity by UHPLC [**SI Figure 193**]) monoisotopic mass calc. 7982.8337, found 7982.8246).

UV-Vis synthesis trace

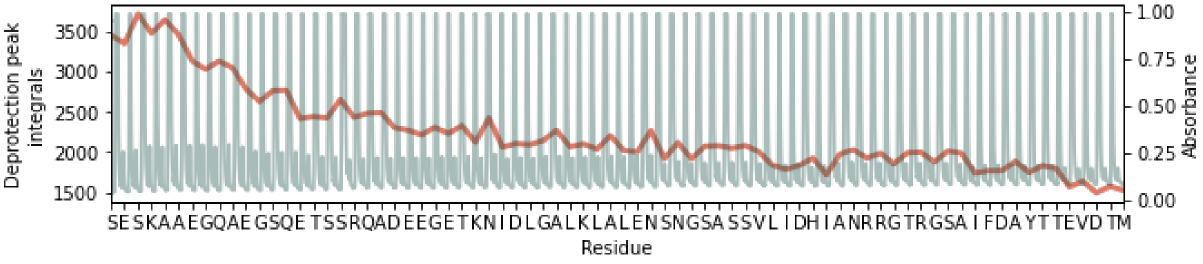

SI Figure 191. UV trace (λ = 310 nm) from AFPS of PKI-α (green) and deprotection peak integrals (red).

LCMS of crude PKI-α

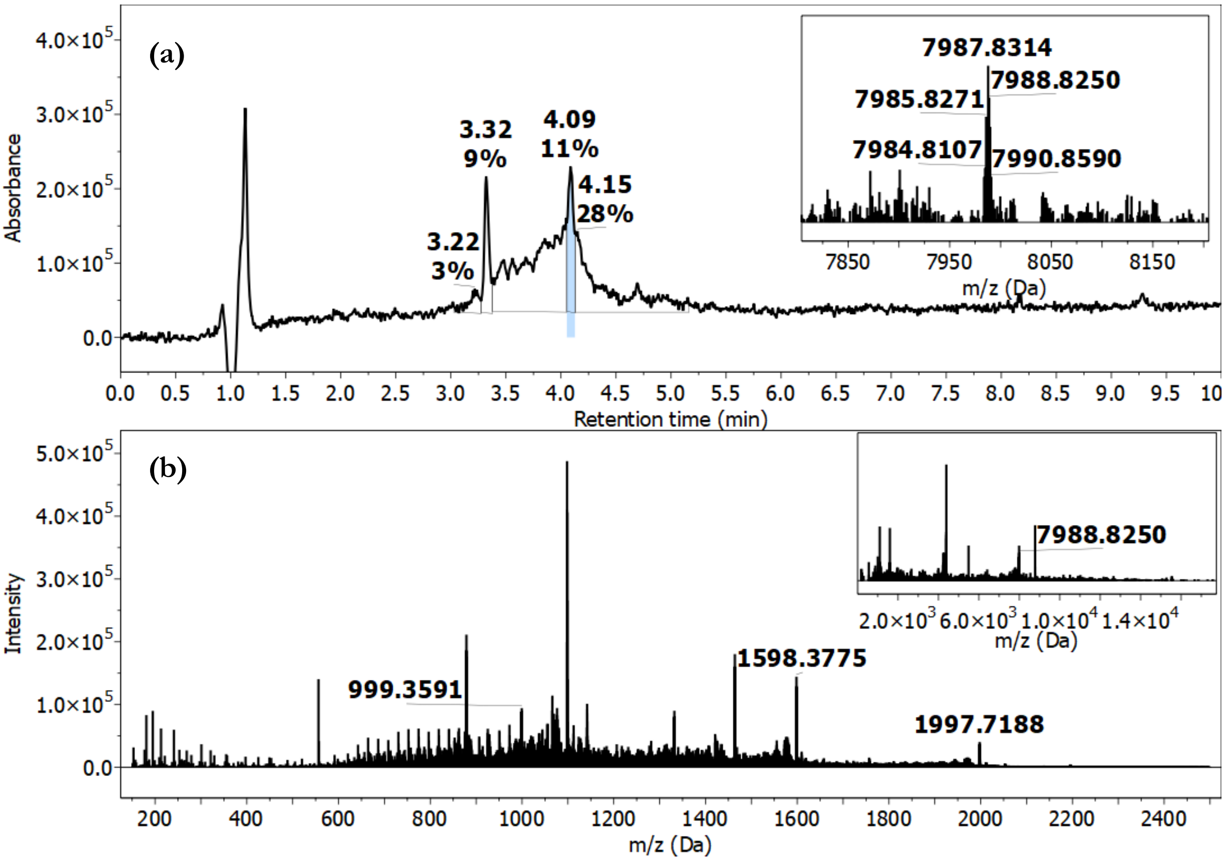

SI Figure 192. LCMS profile of crude PKI-α. (**a**) Absorbance chromatogram (λ = 214 nm); Rt 4.09 min, 11% purity. (**b**) ESI-TOF spectrum found within Rt 2–9 min. Inserts: deconvoluted masses. Monoisotopic mass (ESI+) calcd. for $C_{329}H_{537}N_{101}O_{128}S$ 7982.8337, found 7982.8246. LCMS Gradient A (**Section 2.7**).

UHPLC of crude PKI-α

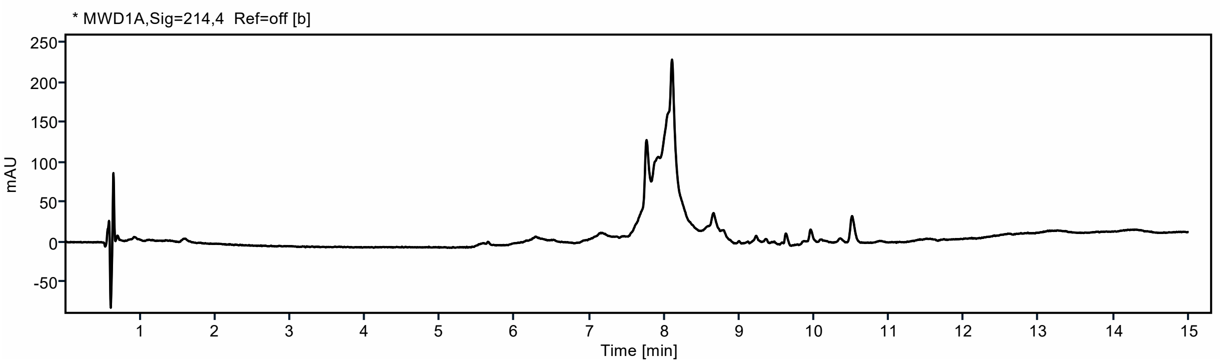

SI Figure 193. UHPLC profile of crude PKI-α. Rt 8.10 min. (Agilent Zorbax 300SB-C18 column, 5 µm, 2.1 × 150 mm, 5–95% MeCN over 10 min, *ca.* 9%B/min), 15% purity based on Area Under Curve (AUC) at λ = 214 nm.

7.3.1 cAMP-dependent protein kinase inhibitor alpha (PKI-α)-SynTag

```
                    10               20               30               40
H2N- MTDVETTYAD     FIASGRTGRR     NAIHDILVSS     ASGNSNELAL
                    50               60               70        76
     KLAGLDINKT     EGEEDAQRSS     TEQSGEAQGE  AAKSES- MeNbz
                    83
   - RRRRRR -CONH2                                         (83 AA)
```

Resin-bound MeDbz-[Arg(Pbf)]$_6$ (145 mg, 20 µmol) (see **Section 7.1**), was swelled in DMF (3 mL) for 10 min, then drained. To a solution of Fmoc-Ser-OH (39 mg, 100 µmol) in DMF (1.0 mL) was added HATU (0.38 M in DMF, 0.20 mL) and DIPEA (35 µL, 0.20 mmol), and the solution was agitated gently for 30 s. The active ester solution was then added to the swelled resin, and left to stand at 23 °C for 1 h, with occasional stirring. The resin was then drained, washed with DMF (3 × 5 mL) and DCM (3 × 5 mL), then dried under reduced pressure. Residues [75]–[62] of the peptide PKI-α were then synthesized upon the Fmoc-Ser(*t*Bu)-MeDbz-[Arg(Pbf)]$_6$ resin using the standard AFPS protocol (**Section 2.2.1**, 20 mL/min flowrate) (**SI Figure 194**). After full completion of the Glu[62] coupling cycle, the synthesis was briefly stopped due to a minor leaking issue before continuing with the elongation of the sequence (residues [61]–[2]) (**SI Figure 195**). Total synthesis time to afford resin-bound PKI-α-(SynTag) was approximately 4 h. The final amino acid, Boc-L-Met(Boc)-OH (25 mg, 0.10 mmol) was coupled manually using the standard batch coupling procedure (**SI Section 2.2.4**).

UV-Vis synthesis trace

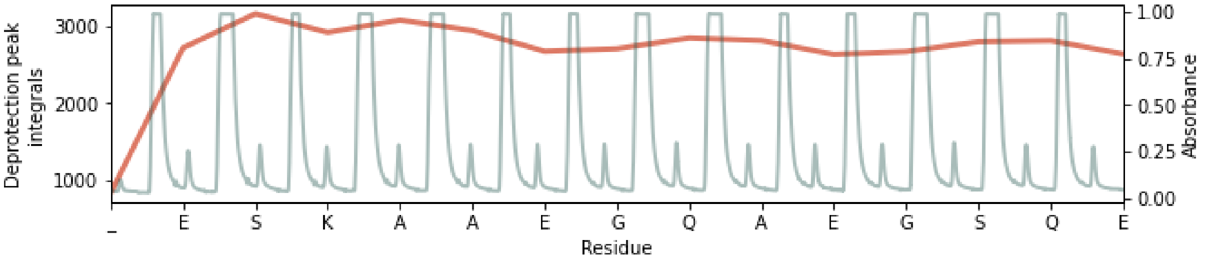

SI Figure 194. UV trace (λ = 310 nm) from AFPS of PKI-α-[62–75]-MeDbz-[Arg(Pbf)]$_6$ (green) and deprotection peak integrals (red).

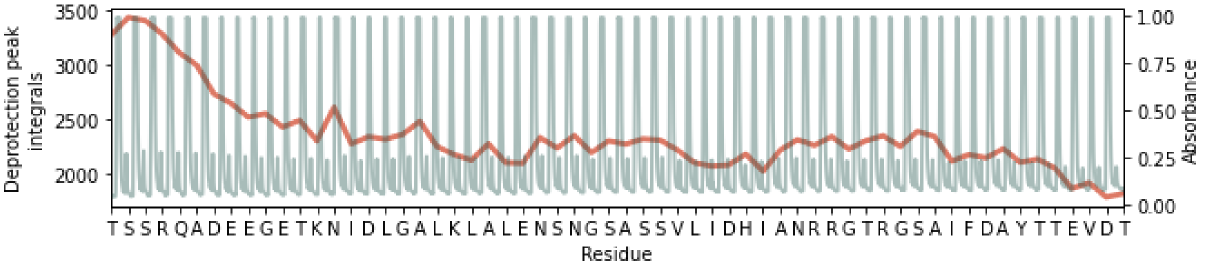

SI Figure 195. UV trace (λ = 310 nm) from AFPS of PKI-α[2–61]-MeDbz-[Arg(Pbf)]$_6$ (green) and deprotection peak integrals (red).

Acylation and cyclisation of MeDbz to afford MeNbz.(43) The peptidyl-resin (76 mg, approx. 6.2 µmol) was swelled in DCM (3 mL) for 10 min, then drained. To the peptidyl-resin was added a solution of 4-nitrophenylchloroformate (20 mg, 96 µmol) in DCM (1.0 mL), and the reaction was allowed to proceed at 23 °C with occasional stirring for 1 h. The resin was then drained and washed with DCM (3 × 3 mL) and DMF (3 × 3 mL). A solution of DIPEA (0.10 mL, 0.60 mmol) in DMF (1.0 mL) was then added to the resin affording a yellow mixture, and the reaction was allowed to proceed at 23 °C with occasional stirring for 30 min. The resin was then drained and washed with DMF (3 × 3 mL). The DIPEA reaction was repeated as necessary (3 × 30 min in total), until a yellow color no longer developed after 20 min reaction time. The resin was then washed with DCM (3 × 3 mL) and dried under reduced pressure.

Cleavage of the peptidyl-resin (76 mg, approx. 6.2 µmol) according to Cleavage Protocol A (**Section 2.5.1**) afforded the crude peptide (14 mg, 23% purity by LCMS [**SI Figure 196**], 49% purity by UHPLC [**SI Figure 197**]).

LCMS of crude PKI-α-(SynTag)

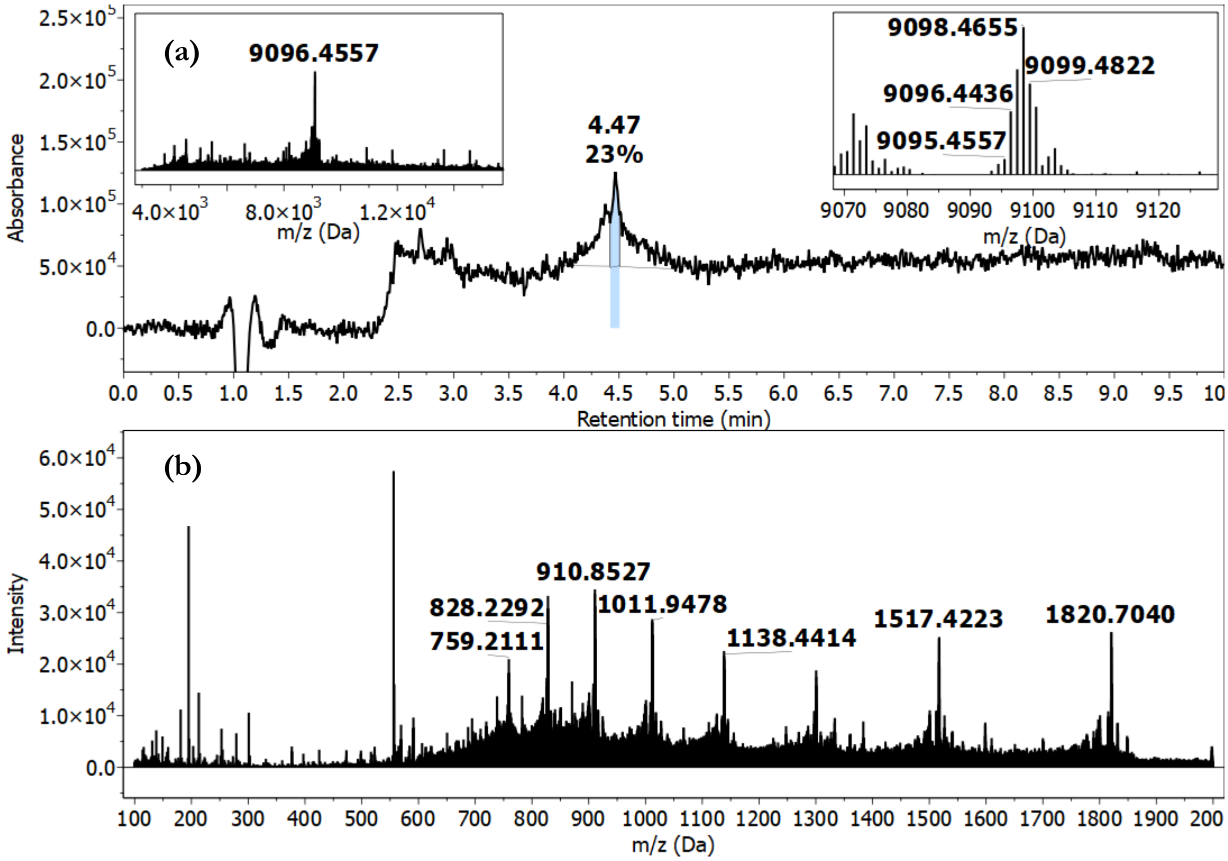

SI Figure 196. LCMS profile of crude PKI-α-MeNbz-Arg$_6$. (**a**) Absorbance chromatogram (λ = 214 nm); Rt 4.47 min, 23% purity. (**b**) ESI-TOF spectrum found within Rt 4–5 min. Inserts: deconvoluted masses. Monoisotopic mass (ESI+) calcd. for $C_{374}H_{614}N_{126}O_{137}S$ 9094.4673, found 9094.4721. LCMS Gradient B (**Section 2.7**).

UHPLC of crude PKI-α-(SynTag)

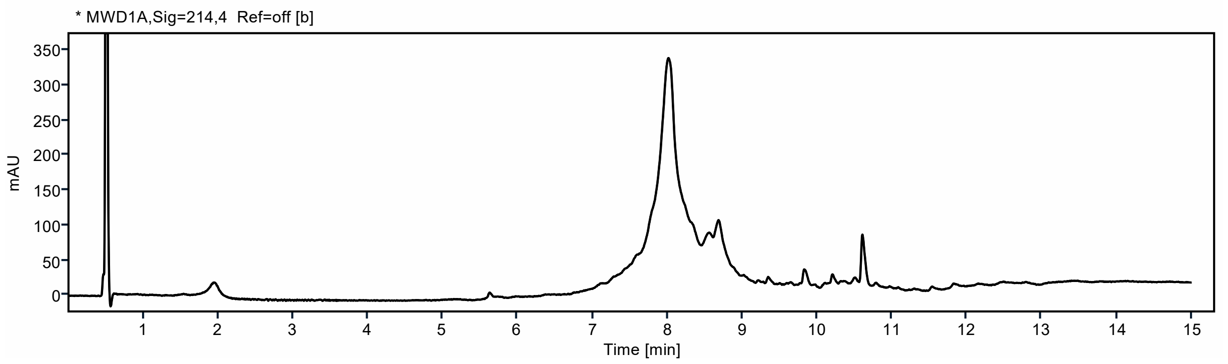

SI Figure 197. UHPLC profile of crude PKI-α-MeNbz-Arg$_6$. Rt 8.016min. (Agilent Zorbax 300SB-C18 column, 5 µm, 2.1 × 150 mm, 5–95% MeCN over 10 min, ca. 9%B/min), 49% purity based on Area Under Curve (AUC) at λ = 214.

7.3.2 Comparison of cAMP-dependent protein kinase inhibitor alpha (PKI-α) with and without SynTag

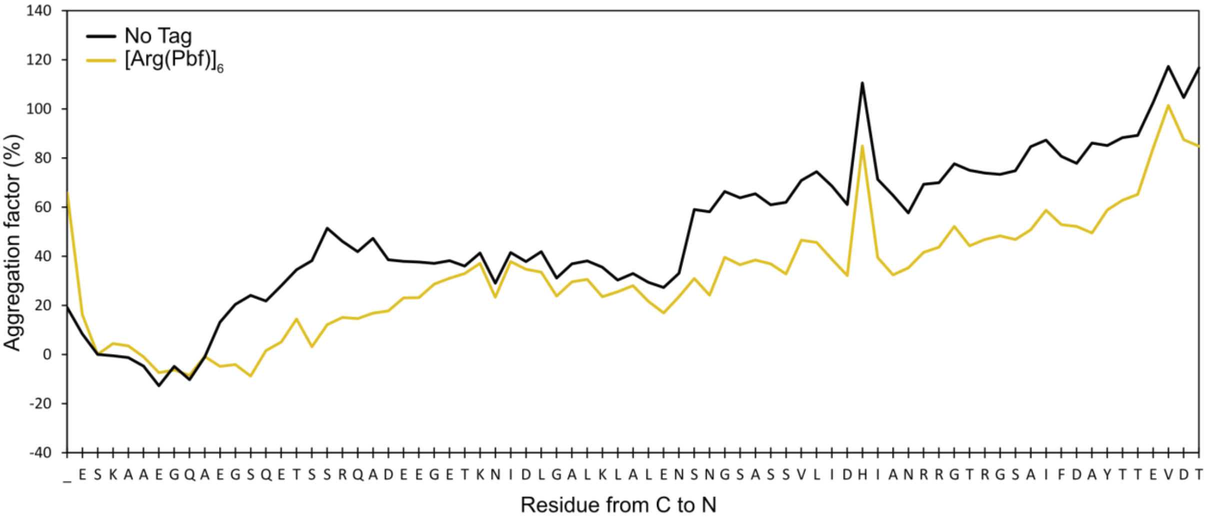

SI Figure 198. Aggregation as a function of Fmoc-deprotection peak broadening (%) by in-line UV-Vis (310 nm) in flow-SPPS for PKI-α with and without SynTag (MeDbz-[Arg(Pbf)]$_6$), normalized at Ser[74].

7.4 Crambin

7.4.1 Crambin (no tag)

```
                     10           20            30           40
H2N-TTCCPSIVAR  SNFNVCRLPG  TPEALCATYT  GCIIIPGATC

                     46
   PGDYAN-CONH2       (46 AA)
```

The peptide Crambin was synthesized on commercially available Novabiochem® NovaPEG Rink Amide resin (0.2 mmol/g, 153 mg, 31 μmol) using the standard AFPS protocol (**Section 2.2.1**, 20 mL/min flowrate). Total synthesis time to afford resin-bound Crambin (final resin weight: 322 mg) was approximately 2 h (**SI Figure 199**). Cleavage of the peptidyl-resin (33 mg, approx. 3.1 μmol) according to Cleavage Protocol A (**Section 2.5.1**) afforded the crude peptide (7.6 mg, *n/a* purity by LCMS [**SI Figure 200**], approx. 7% purity by UHPLC [**SI Figure 201**]) monoisotopic mass calc. 4732.2038, found 4732.2081).

UV-Vis synthesis trace

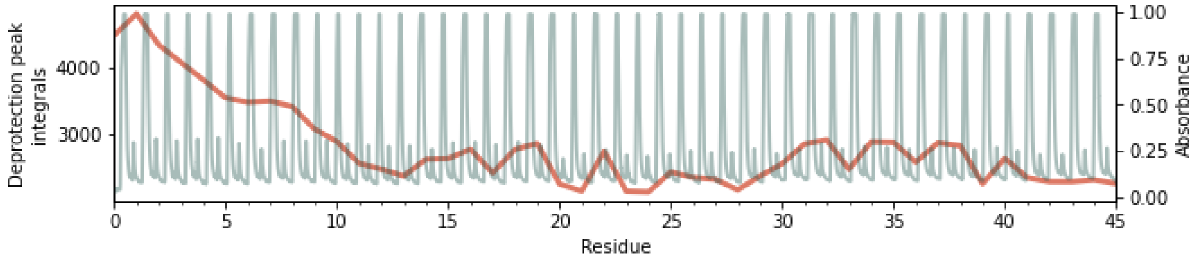

SI Figure 199. UV trace (λ = 310 nm) from AFPS of Crambin (green) and deprotection peak integrals (red).

LCMS of crude Crambin

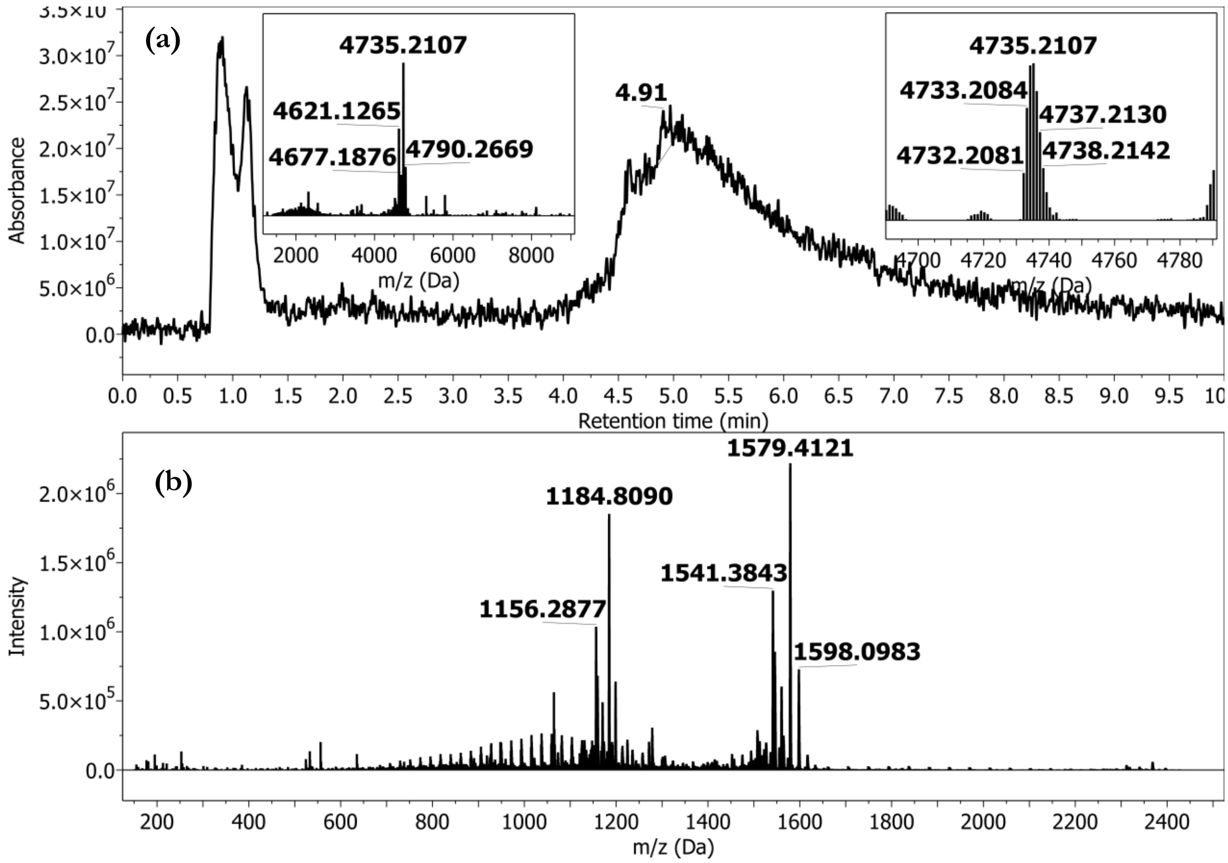

SI Figure 200. LCMS profile of crude Crambin. (**a**) Absorbance chromatogram (λ = 214 nm); Rt 4.91 min. (**b**) ESI-TOF spectrum found within Rt 2–9 min. Inserts: deconvoluted masses. Monoisotopic mass (ESI+) calcd. for $C_{202}H_{322}N_{56}O_{63}S_6$ 4732.2038, found 4732.2081. LCMS Gradient B (**Section 2.7**).

UHPLC of crude Crambin

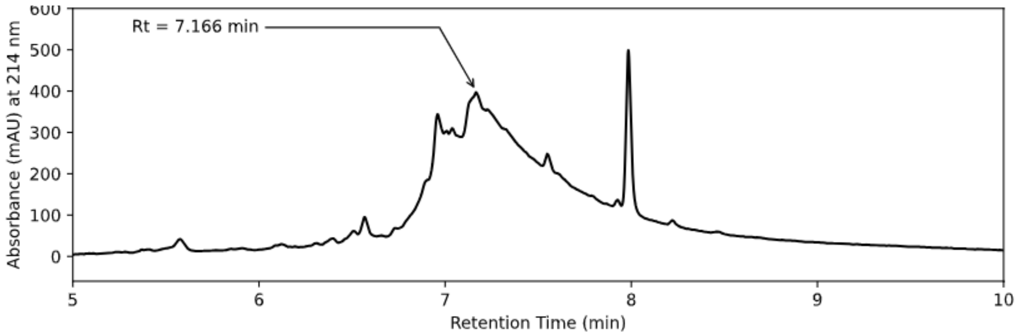

SI Figure 201. UHPLC profile of crude Crambin. Rt 7.17 min (Agilent Poroshell 300SB-C8 column, 5 µm, 2.1 × 75 mm, 5–95% MeCN over 10 min, ca. 9%B/min), approx. 7% crude purity based on Area Under Curve (AUC) at λ = 214 nm.

7.4.2 Crambin-SynTag

```
           10          20          30          40
H2N-TTCCPSIVAR  SNFNVCRLPG  TPEALCATYT  GCIIIPGATC

           46
PGDYAN-MeNbz- RRRRRR-CONH2      (53 AA)
```

Resin-bound MeDbz-[Arg(Pbf)]$_6$ (153 mg, 31 µmol) (see **Section 7.1**), was swelled in DMF (3 mL) for 10 min, then drained. To a solution of Fmoc-Asn(Trt)-OH (0.40 M in DMF, 2.0 mL) was added HATU (0.38 M in DMF, 2.0 mL) and DIPEA (0.50 mL), and the solution was agitated gently for 30 s. The active ester solution was then added to the swelled resin, and left to stand at 23 °C for 30 min, with occasional stirring. The resin was then drained, washed with DMF (3 × 5 mL) and DCM (3 × 5 mL), then dried under reduced pressure. Residues 2–46 of the peptide Crambin were then synthesized upon the Fmoc-Asn(Trt)-MeDbz-[Arg(Pbf)]$_6$ resin using the standard AFPS protocol (**Section 2.2.1**, 20 mL/min flowrate) (**SI Figure 202**). Total synthesis time to afford resin-bound Crambin[2–46]-MeDbz-[Arg(Pbf)]$_6$ (final resin weight: 358 mg) was approximately 2 h. The final amino acid, Boc-L-Thr(*t*Bu)-OH (0.11 g, 0.40 mmol) was coupled manually at 90 °C with HATU (0.38 M in DMF, 0.71 µL, 0.27 mmol) and DIPEA (0.20 mL) and the pre-activation solution was agitated gently for 30 s. The active ester solution was then added to the swelled resin, and left to stand at 23 °C for 1 h, with occasional stirring. The resin was then washed with DMF (3 × 3 mL) and DCM (3 × 3 mL), then dried under reduced pressure.

UV-Vis synthesis trace

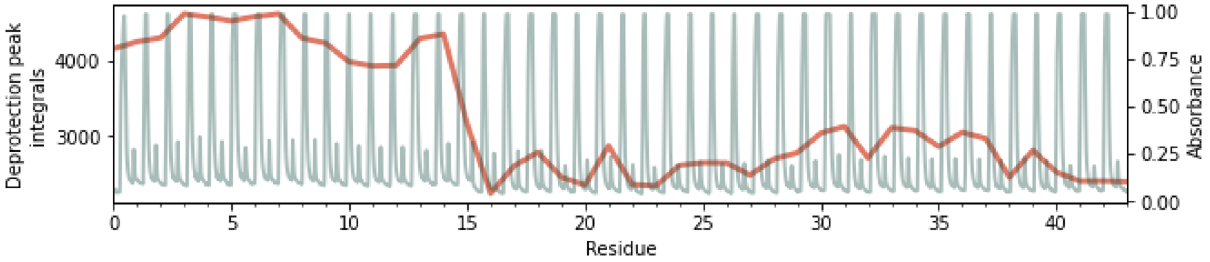

SI Figure 202. UV trace (λ = 310 nm) from AFPS of Crambin[2–46]-MeDbz-[Arg(Pbf)]$_6$ (green) and deprotection peak integrals (red).

Acylation and cyclisation of MeDbz to afford MeNbz.(43) The peptidyl-resin (35 mg, approx. 2.9 µmol) was swelled in DCM (3 mL) for 10 min, then drained. To the peptidyl-resin was added a solution of 4-nitrophenylchloroformate (11 mg, 55 µmol) in DCM (1.0 mL), and the reaction was allowed to proceed at 23 °C with occasional stirring for 1 h. The resin was then drained, washed with DCM (3 × 3 mL) and DMF (3 × 3 mL). A solution of DIPEA (0.10 mL, 0.60 mmol) in DMF (2.0 mL) was then added to the resin affording a yellow mixture, and the reaction was allowed to proceed at 23 °C with occasional stirring for 25 min. The resin was then drained and washed with DMF (3 × 3 mL). The DIPEA reaction was repeated as necessary (3 × 30 min in total), until a yellow color no longer developed after 20 min reaction time. The resin was then washed with DCM (3 × 3 mL) and dried under reduced pressure.

Cleavage of the peptidyl-resin (35 mg, approx. 2.9 µmol) according to Cleavage Protocol A (**Section 2.5.1**) afforded the crude peptide (3.3 mg, *n/a* purity by LCMS [**SI Figure 203**], approx. 19% purity by UHPLC [**SI Figure 204**], monoisotopic mass calc. 5842.8534, found 5842.7886).

LCMS of crude Crambin-MeNbz-Arg$_6$

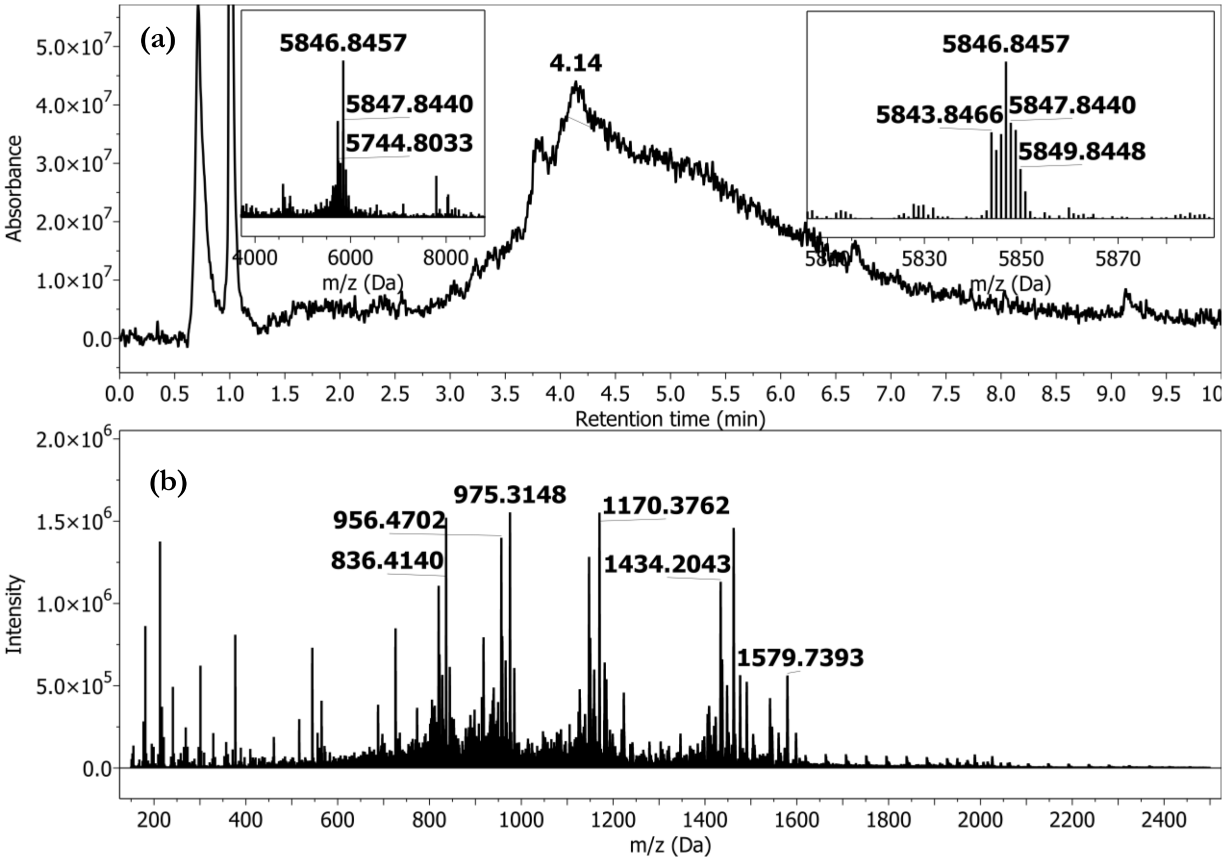

SI Figure 203. LCMS profile of crude Crambin-MeNbz-Arg$_6$. (**a**) Absorbance chromatogram (λ = 214 nm); Rt 4.14 min. (**b**) ESI-TOF spectrum found within Rt 2–9 min. Inserts: deconvoluted masses. Monoisotopic mass (ESI+) calcd. for $C_{247}H_{400}N_{82}O_{71}S_6$ 5842.8534, found 5842.7886. LCMS Gradient B (**Section 2.7**).

UHPLC of crude Crambin-MeNbz-Arg$_6$

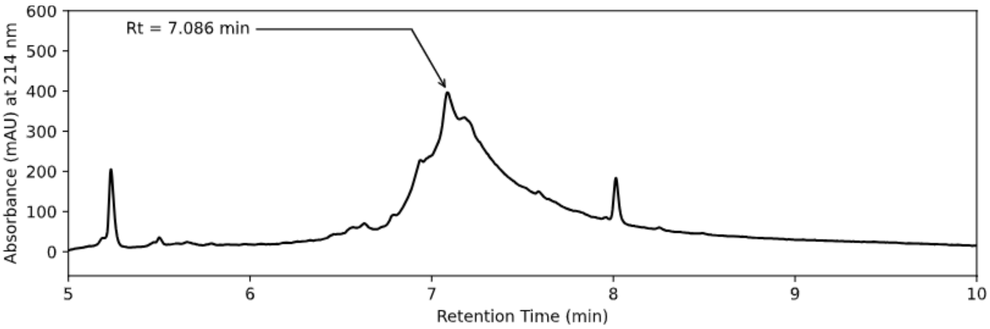

SI Figure 204. UHPLC profile of crude Crambin-MeNbz-Arg$_6$. Rt 7.09 min (Agilent Poroshell 300SB-C8 column, 5 µm, 2.1 × 75 mm, 5–95% MeCN over 10 min, ca. 9%B/min), approx. 19% crude purity based on Area Under Curve (AUC) at λ = 214 nm.

7.5 Amyloid-β42[27–42]-SynTag

```
                10                          22
H2N-KGAIIGLMVG   GVVIA-MeNbz  -RRRRRR-CONH2   (22 AA)
```

Resin-bound MeDbz-[Arg(Pbf)]$_6$ (93 mg, 10 μmol) (see **Section 7.1**), was swelled in DMF (3 mL) for 10 min, then drained. To a solution of Fmoc-Ala-OH (16 mg, 50 μmol) in DMF (1.0 mL) was added HATU (0.38 M in DMF, 0.10 mL) and DIPEA (13 μL, 0.10 mmol), and the solution was agitated gently for 30 s. The active ester solution was then added to the swelled resin, and left to

stand at 23 °C for 1 h, with occasional stirring. The resin was then drained, washed with DMF (3 × 5 mL) and DCM (3 × 5 mL), then dried under reduced pressure. Residues 28–41 of the peptide Amyloid-β42[27–42] were then synthesized upon the Fmoc-Ala-MeDbz-[Arg(Pbf)]$_6$ resin using the standard AFPS protocol (**Section 2.2.1**, 20 mL/min flowrate) (**SI Figure 205**). Total synthesis time to afford resin-bound Amyloid-β42[28–42] was approximately 1 h. The final amino acid, Boc-L-Lys(Boc)-OH·DHCA (0.43 g, 0.80 mmol) was coupled manually at 90 °C with HATU (0.38 M in DMF, 2.0 mL, 0.77 mmol) and DIPEA (0.10 mL, 0.60 mmol) for 3 × 5 min. The resin was then washed with DMF (3 × 3 mL) and DCM (3 × 3 mL), then dried under reduced pressure.

UV-Vis synthesis trace

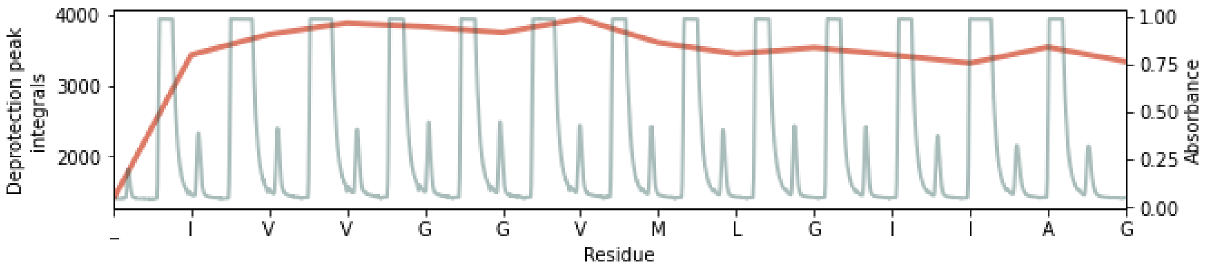

SI Figure 205. UV trace (λ = 310 nm) from AFPS of Amyloid-β42[27–42]-MeDbz-[Arg(Pbf)]$_6$ (green) and deprotection peak integrals (red).

Acylation and cyclisation of MeDbz to afford MeNbz.(*43*) The peptidyl-resin (39 mg, approx. 7.7 µmol) was swelled in DCM (3 mL) for 10 min, then drained. To the peptidyl-resin was added a solution of 4-nitrophenylchloroformate (20 mg, 96 µmol) in DCM (1.0 mL), and the reaction was allowed to proceed at 23 °C with occasional stirring for 1 h. The resin was then drained, washed with DCM (3 × 3 mL) and DMF (3 × 3 mL). A solution of DIPEA (0.10 mL, 0.60 mmol) in DMF (1.0 mL) was then added to the resin affording a yellow mixture, and the reaction was allowed to proceed at 23 °C with occasional stirring for 20 min. The resin was then drained and washed with DMF (3 × 3 mL). The DIPEA reaction was repeated as necessary (3 × 30 min), until a yellow color no longer developed after 20 min reaction time. The resin was then washed with DCM (3 × 3 mL) and dried under reduced pressure.

Cleavage of the peptidyl-resin (39 mg, approx. 7.7 µmol) according to Cleavage Protocol A (**Section 2.5.1**) afforded the crude peptide (5.9 mg, 70% purity by LCMS [**SI Figure 206**], 73% purity by UHPLC [**SI Figure 207**], monoisotopic mass calc. 2506.5132, found 2506.5127). The crude peptide (5.9 mg) was purified by semi-prep RP-HPLC using an Agilent Zorbax 300SB-C18 Semi-Preparative column (9.4 × 250 mm, 5 µm particle size) at a flow rate of 3.5 mL/min, with a gradient of 10–60%B over 50 min (*ca.* 1%B/min). Fractions were analyzed by LCMS and UHPLC, combined, and lyophilized to afford the *title compound* (1.3 mg, 93% purity by LCMS [**SI Figure 208**], >95% purity by UHPLC [**SI Figure 209**], 7% overall yield, monoisotopic mass calc. 2506.5132, found 2506.5137).

LCMS of crude Amyloid-β42[27–42]-MeNbz-Arg$_6$

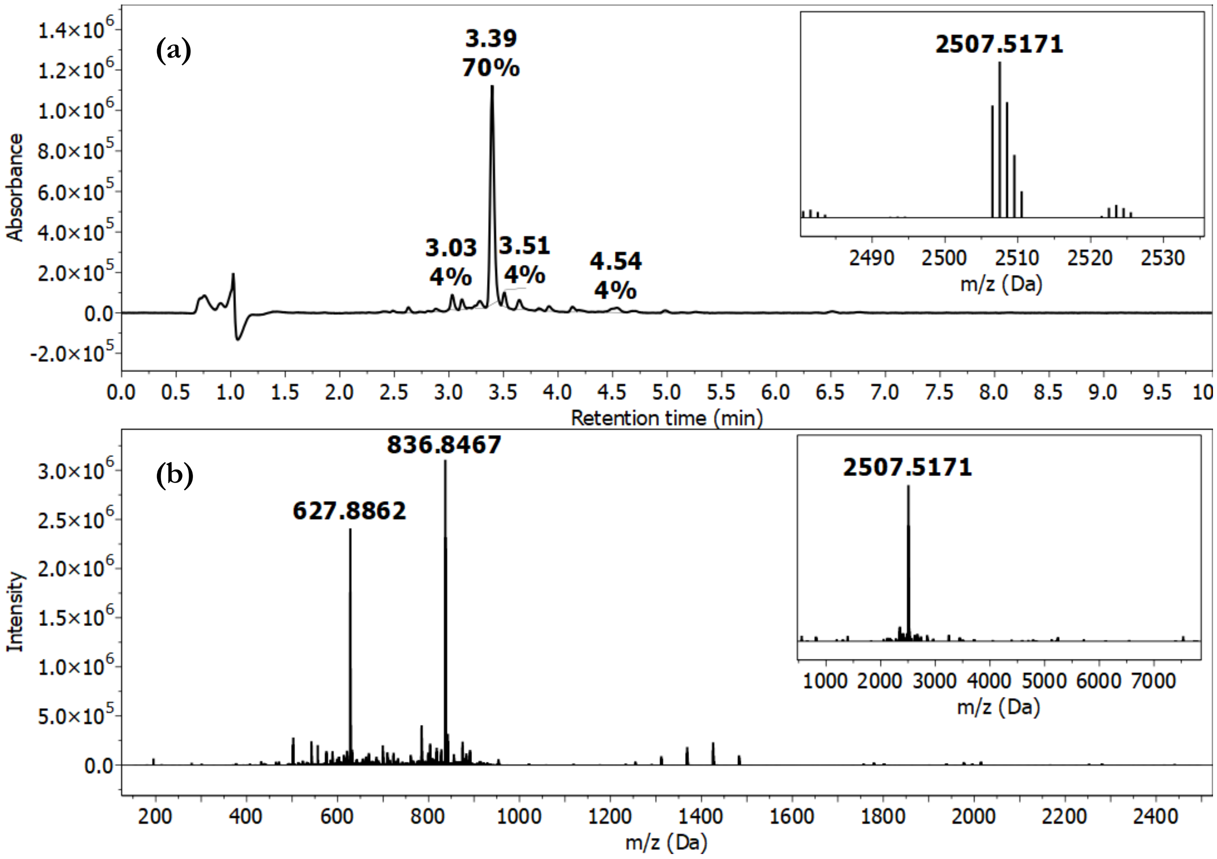

SI Figure 206. LCMS profile of crude Amyloid-β42[27–42]-MeNbz-Arg$_6$. (**a**) Absorbance chromatogram (λ = 214 nm); Rt 3.39 min, 70% purity. (**b**) ESI-TOF spectrum found within Rt 2–9 min. Inserts: deconvoluted masses. Monoisotopic mass (ESI+) calcd. for $C_{109}H_{195}N_{43}O_{23}S$ 2506.5132, found 2506.5127. LCMS Gradient A (**Section 2.7**).

UHPLC of crude Amyloid-β42[27–42]-MeNbz-Arg$_6$

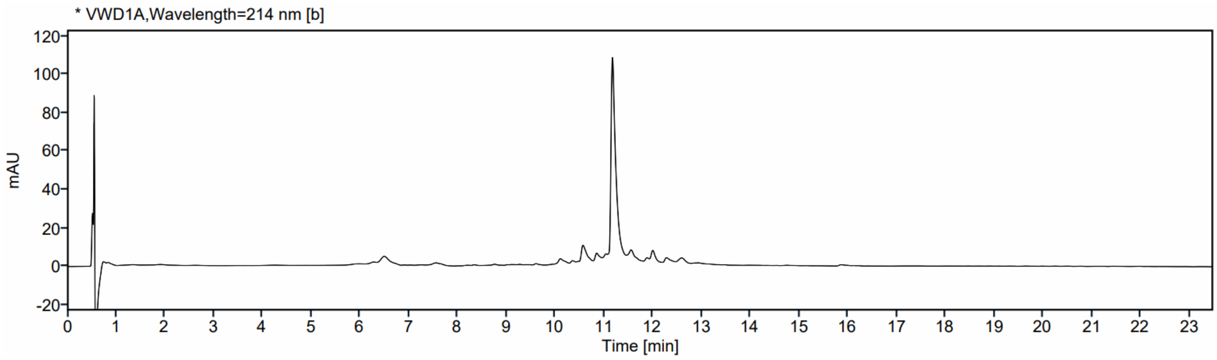

SI Figure 207. UHPLC profile of crude Amyloid-β42[27–42]-MeNbz-Arg$_6$. Rt 11.18 min. (Agilent Zorbax 300SB-C18 column, 5 µm, 2.1 × 150 mm, 5–95% MeCN over 20 min, *ca.* 4.5%B/min), 73% purity based on Area Under Curve (AUC) at λ = 214 nm.

LCMS of pure Amyloid-β42[27–42]-MeNbz-Arg$_6$

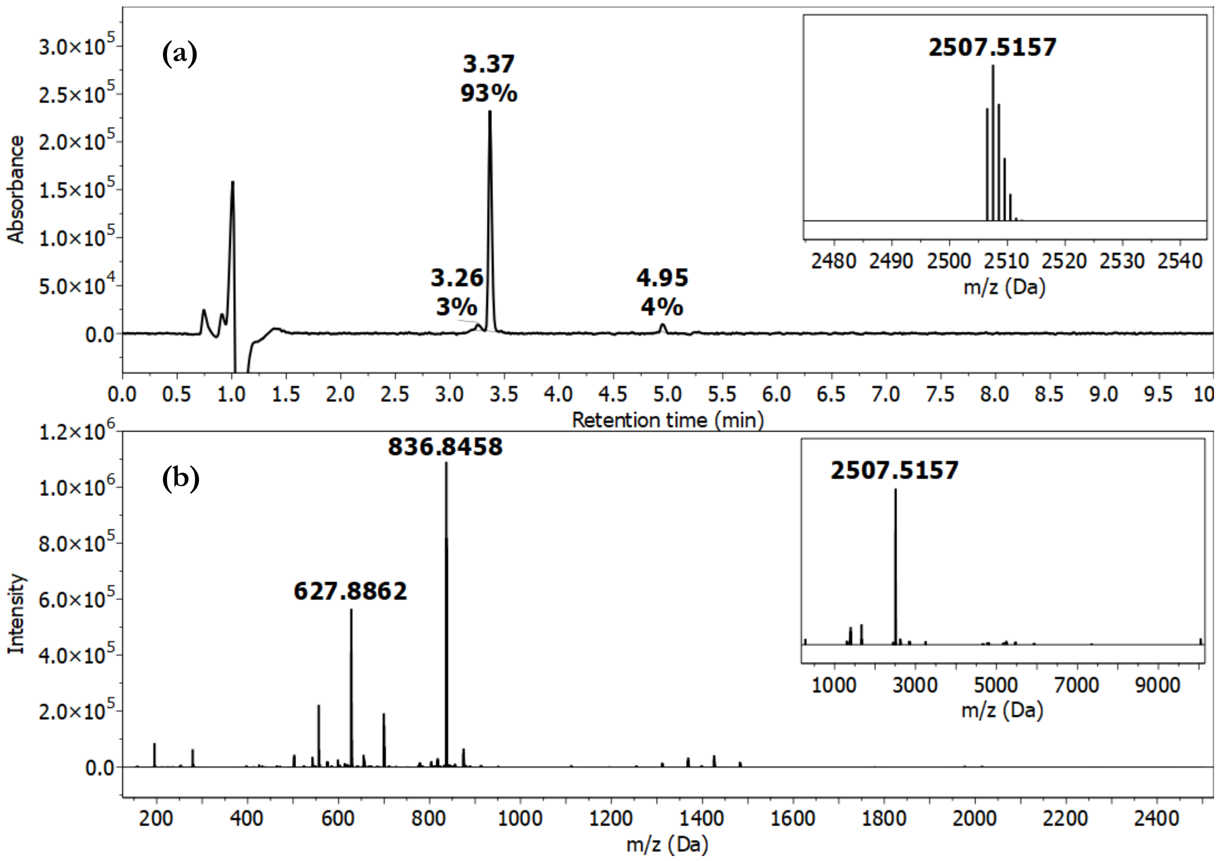

SI Figure 208. LCMS profile of purified Amyloid-β42[27–42]-MeNbz-Arg$_6$. (**a**) Absorbance chromatogram (λ = 214 nm); Rt 3.37 min, 93% purity. (**b**) ESI-TOF spectrum found within Rt 2–9 min. Inserts: deconvoluted masses. Monoisotopic mass (ESI+) calcd. for $C_{109}H_{195}N_{43}O_{23}S$ 2506.5132, found 2506.5137. LCMS Gradient A (**Section 2.7**).

UHPLC of pure Amyloid-β42[27–42]-MeNbz-Arg$_6$

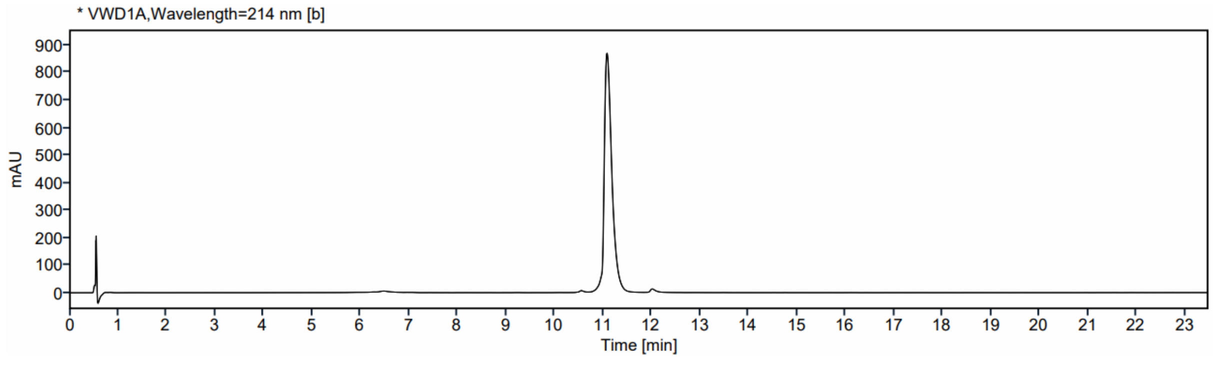

SI Figure 209. UHPLC profile of pure Amyloid-β42[27–42]-MeNbz-Arg$_6$. Rt 11.09 min. (Agilent Zorbax 300SB-C18 column, 5 µm, 2.1 × 150 mm, 5–95% MeCN over 20 min, *ca.* 4.5%B/min), 96% purity based on Area Under Curve (AUC) at λ = 214 nm.

7.6 Hydrolysis of SynTag to afford Amyloid-β42[27–42]-OH

```
               10
H2N-KGAIIGLMVG GVVIA-COOH (15 AA)
```

The peptide Amyloid-β42[27–42] bearing MeNbz-(Arg)$_6$ (SynTag) (1.3 mg) was dissolved in water (1.3 mL) and left to stand at 23 °C for 30 h. After 30 h, the reaction was 95% complete as evidenced by LCMS (**SI Figure 210**) and UHPLC (**SI Figure 211**). The reaction was then lyophilised, affording Amyloid-β42[27–42]-OH (0.60 mg, monoisotopic mass calc. 1396.8476, found 1396.8491).

LCMS of Amyloid-β42[27–42]-OH

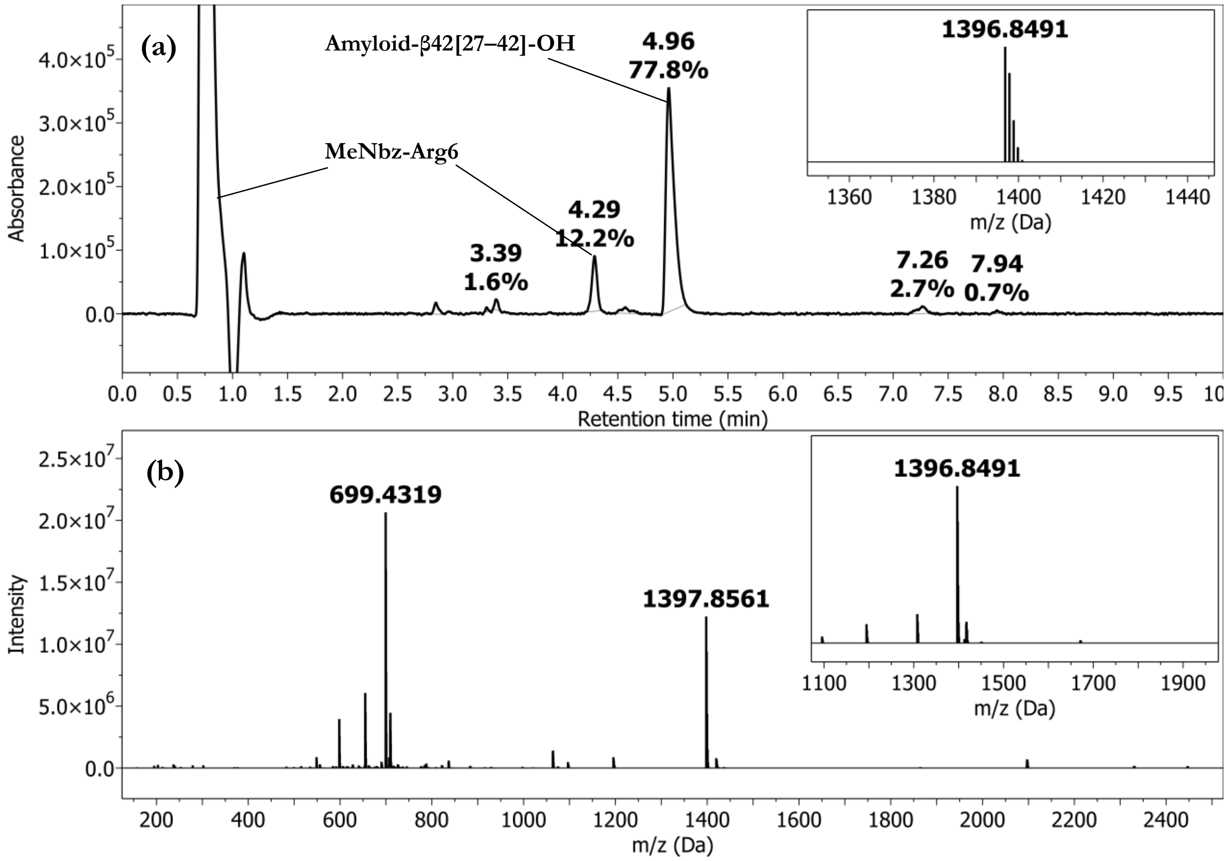

SI Figure 210. LCMS profile of Amyloid-β42[27–42]-OH afforded by hydrolysis of Amyloid-β42[27–42]-MeNbz-Arg$_6$. (**a**) Absorbance chromatogram (λ = 214 nm); Rt 4.96 min. (**b**) ESI-TOF spectrum found within Rt 2–9 min. Inserts: deconvoluted masses. Monoisotopic mass (ESI+) calcd. for $C_{64}H_{116}N_{16}O_{16}S$ 1396.8476, found 1396.8491. The MeNbz-Arg$_6$ hydrolysis product is found both at the injection peak (Rt ~0.7 min) and at Rt 4.29 min. LCMS Gradient A (**Section 2.7**).

UHPLC of Amyloid-β42[27–42]-OH

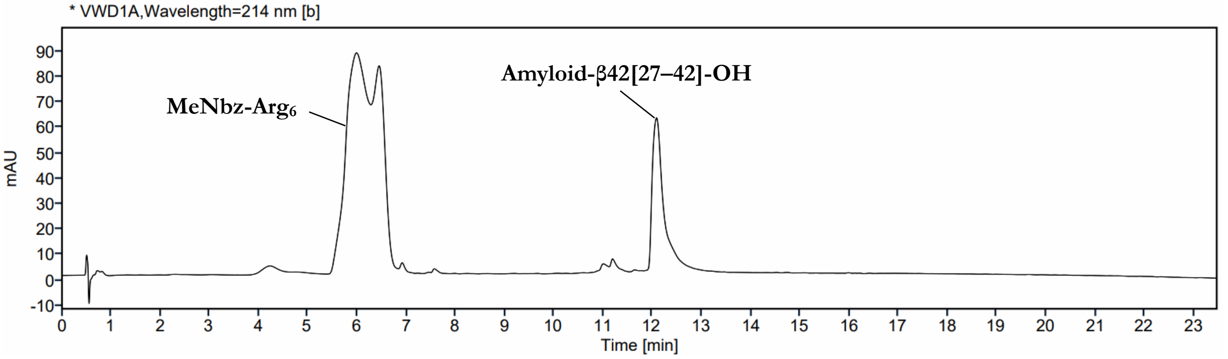

SI Figure 211. UHPLC profile of Amyloid-β42[27–42]-OH afforded by hydrolysis of Amyloid-β42[27–42]-MeNbz-(Arg)$_6$. Rt 12.08 min. (Agilent Zorbax 300SB-C18 column, 5 µm, 2.1 × 150 mm, 5–95% MeCN over 20 min, *ca.* 4.5%B/min, λ = 214 nm). Note: the hydrolysis products MeNbz-Arg$_6$ and Amyloid-β42[27–42]-OH are formed in equimolar amounts. However, the MeNbz-Arg$_6$ product is presumed to give a greater absorption at λ = 214 nm. Thereby, the Area Under Curve (AUC) of the peaks in this chromatogram does not provide an accurate measurement of the sample purity.

8 Chemical Synthesis of MYC[1–143] using SynTag

8.1 SynTag: MYC[1–84]-MeNbz-Arg$_6$

```
                10           20           30           40
H2N-MPLNVSFTNR   NYDLDYDSVQ   PYFYCDEEEN   FYQQQQQSEL
                50           60           70           80
    QPPAPSEDIW   KKFELLPTPP   LSPSRRSGLC   SPSYVAVTPF
                        91
    SLRG-MeNbz  -RRRRRR-CONH2                            (91 AA)
```

The peptide MYC[1–84] bearing the MeDbz-[Arg(Pbf)]$_6$-Tag (SynTag) on the C-terminus, and Boc-L-Met-OH at the N-terminus, was synthesized on Novabiochem® NovaPEG Rink Amide resin that was pre-loaded with Fmoc-MeDbz-[Arg(Pbf)]$_6$ (0.21 g, 30 µmol, see **Section 7.1**) using the standard AFPS protocol (**Section 2.2.1**, 20 mL/min flowrate). The synthesis was paused after the completion of the full coupling cycle of Pro[57], and the peptidyl-resin was dried over vacuum and stored overnight at -20 °C. Synthesis was completed the next day using the standard AFPS protocol (**Section 2.2.1** , 20 mL/min flowrate) (**SI Figure 212**). Total synthesis time to afford resin-bound Boc-MYC[1–84]-MeDbz-[Arg(Pbf)]$_6$ (0.42 g) was approximately 4.0 h.

UV-Vis synthesis traces

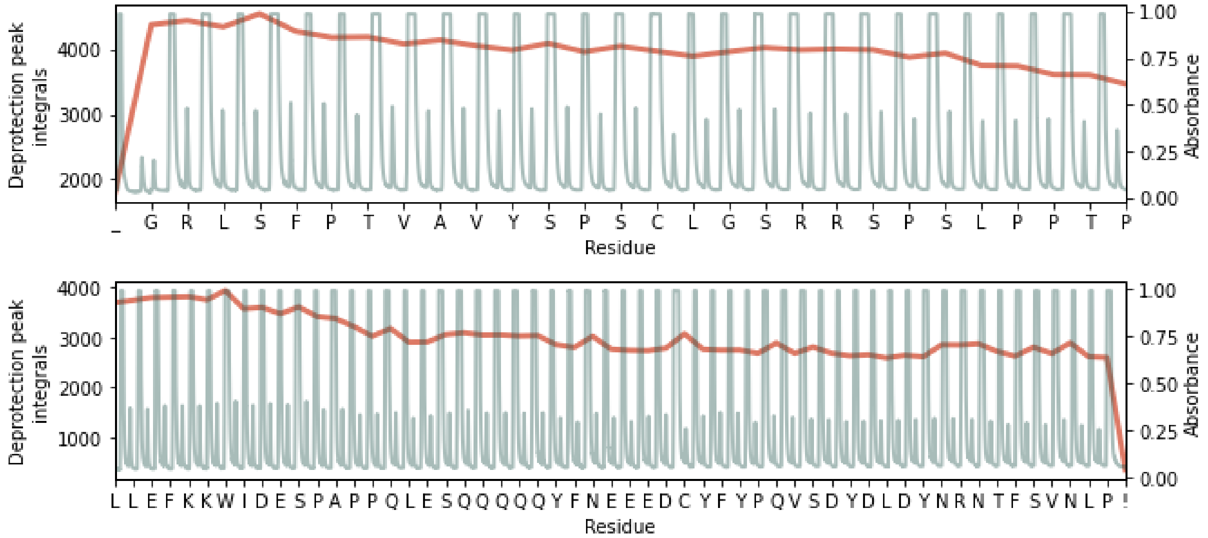

SI Figure 212. UV traces (λ = 310 nm) from AFPS of Boc-MYC[1–84]-MeDbz-[Arg(Pbf)]$_6$ (green) and deprotection peak integrals (red). ! = Boc-L-Met-OH.

Acylation and cyclisation of MeDbz to afford MeNbz.(*43*) The peptidyl-resin (0.21 g, approx. 15 µmol) was swelled in DCM (4 mL) for 10 min, then drained. To the peptidyl-resin was added a solution of 4-nitrophenylchloroformate (30 mg, 0.15 mmol) in DCM (3.0 mL), and the reaction was allowed to proceed at 23 °C with occasional stirring for 1 h. The resin was then drained, and washed with DCM (3 × 3 mL) and DMF (3 × 3 mL). A solution of DIPEA (0.10 mL, 0.60 mmol) in DMF (1.0 mL) was then added to the resin affording a yellow mixture, and the reaction was allowed to proceed at 23 °C with occasional stirring for 0.5 h. The resin was then drained and washed with DMF (3 × 3 mL). The DIPEA reaction was repeated as necessary (3 × 30 min), until a yellow color no longer developed after 20 min reaction time. The resin was then washed with DCM (3 × 3 mL) and dried under reduced pressure. Cleavage of the peptidyl-resin (0.21 g, approx. 15 µmol) according to Cleavage Protocol A (**Section 2.5.1**) afforded the crude peptide (24 mg, 40% purity by

LCMS [**SI Figure 213**], 39% purity by UHPLC [**SI Figure 214**], monoisotopic mass calc. 10858.2939, found 10858.2978). The crude peptide (24 mg) was purified by semi-prep RP-HPLC using an Agilent Eclipse XDB-C8 Semi-Preparative column (9.4 × 250 mm, 5 μm particle size) at a flow rate of 3.0 mL/min, with a two-step gradient of 5–30%B over 25 min (*ca.* 1%B/min), followed by 30–45%B over 60 min (*ca.* 0.25%B/min). Fractions were analyzed by LCMS and UHPLC, combined, and lyophilized to afford the *title compound* (0.86 mg, 95% purity by LCMS [**SI Figure 215**], >95% purity by UHPLC [**SI Figure 216**], 0.5% overall yield, monoisotopic mass calc. 10858.2939, found 10858.2329).

LCMS of crude MYC[1–84]-MeNbz-Arg$_6$

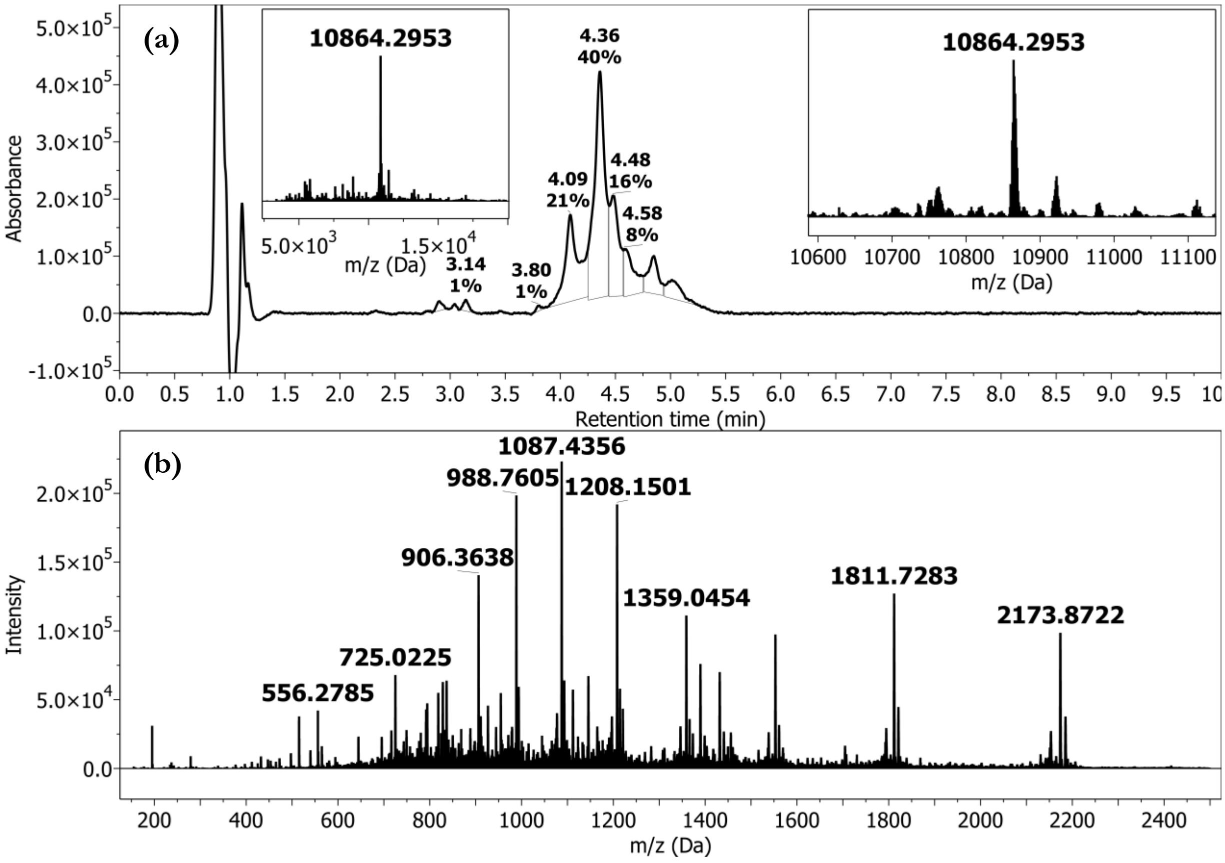

SI Figure 213. LCMS profile of crude MYC[1–84]-MeNbz-Arg$_6$. (**a**) Absorbance chromatogram (λ = 214 nm); Rt 4.36 min, 40% purity. (**b**) ESI-TOF spectrum found within Rt 2–9 min. Inserts: deconvoluted masses. Monoisotopic mass (ESI+) calcd. for $C_{484}H_{727}N_{137}O_{144}S_3$ 10858.2939, found 10858.2978. LCMS Gradient A (**Section 2.7**).

UHPLC of crude MYC[1–84]-MeNbz-Arg$_6$

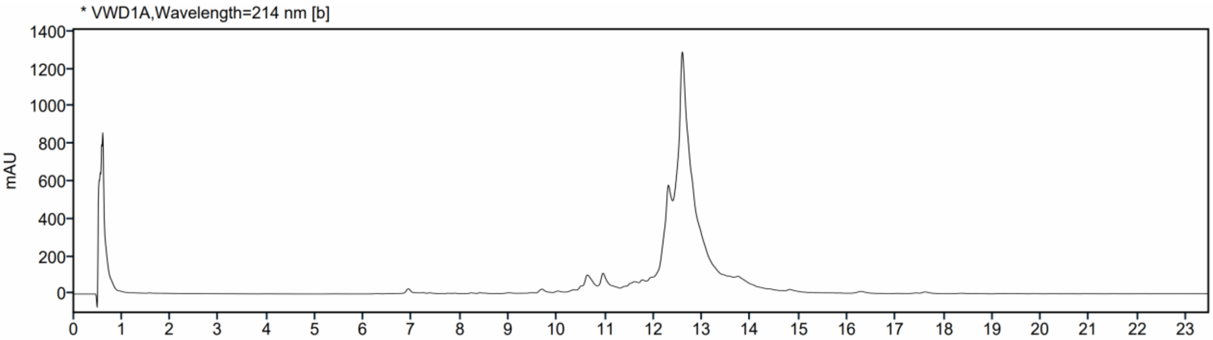

SI Figure 214. UHPLC profile of crude MYC[1–84]-MeNbz-Arg$_6$. Rt 12.60 min. (Agilent Zorbax 300SB-C18 column, 5 µm, 2.1 × 150 mm, 5–95% MeCN over 20 min, *ca.* 4.5%B/min), 39% purity based on Area Under Curve (AUC) at λ = 214 nm.

LCMS of pure MYC[1–84]-MeNbz-Arg$_6$

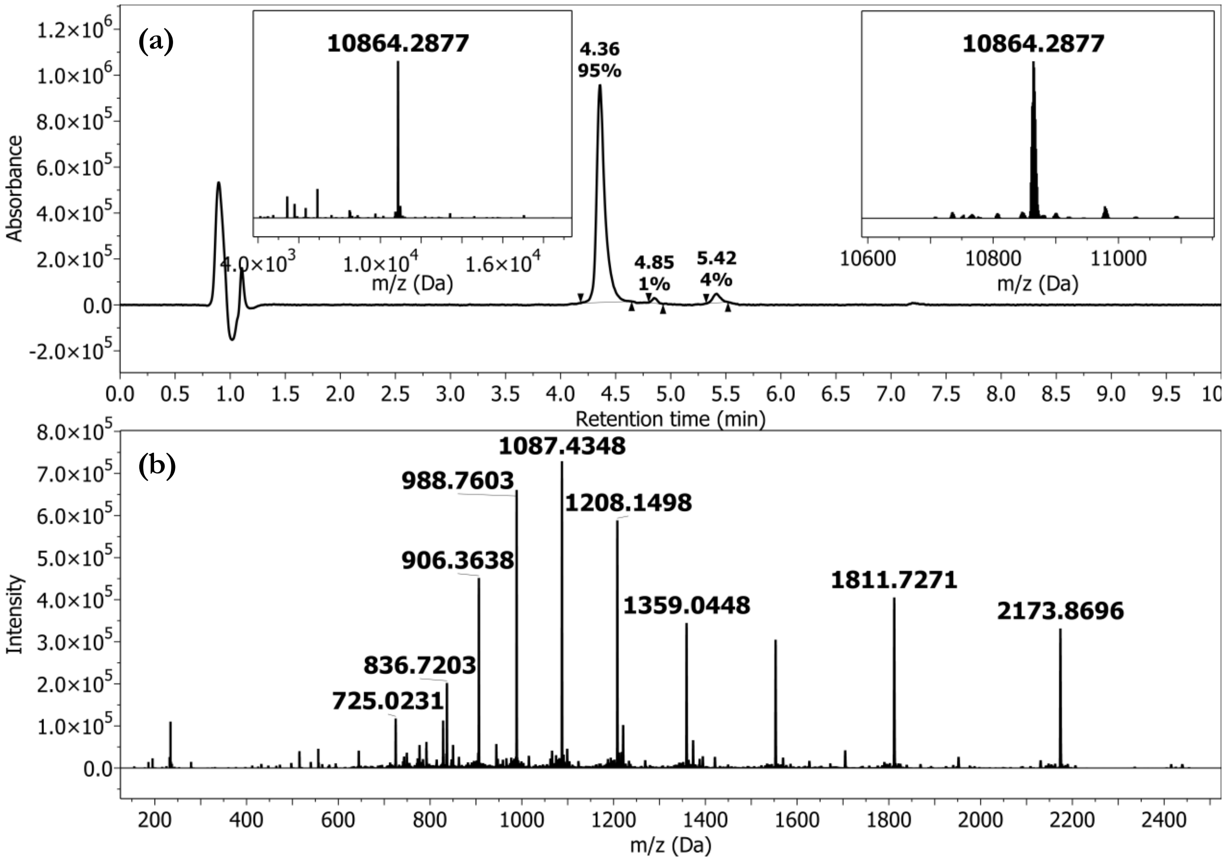

SI Figure 215. LCMS profile of pure MYC[1–84]-MeNbz-Arg$_6$. (**a**) Absorbance chromatogram (λ = 214 nm); Rt 4.36 min, 95% purity. (**b**) ESI-TOF spectrum found within Rt 2–9 min. Inserts: deconvoluted masses. Monoisotopic mass (ESI+) calcd. for $C_{484}H_{727}N_{137}O_{144}S_3$ 10858.2939, found 10858.2329. LCMS Gradient A (**Section 2.7**).

UHPLC of pure MYC[1–84]-MeNbz-Arg$_6$

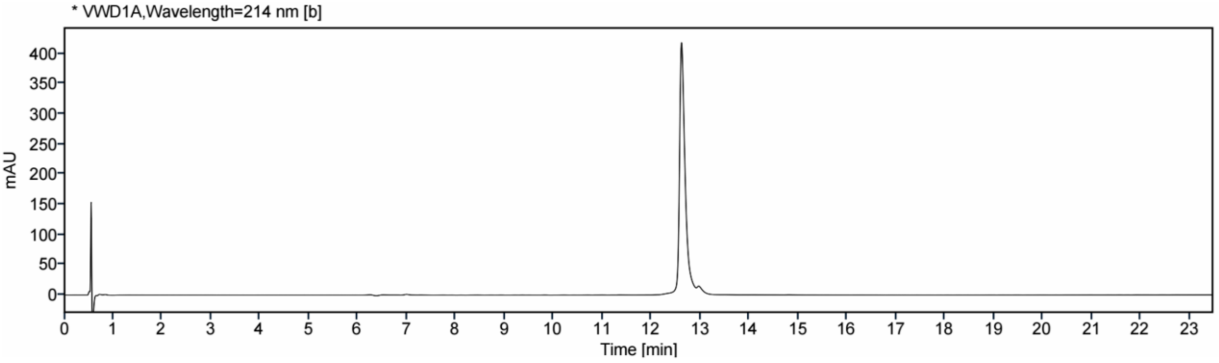

SI Figure 216. UHPLC profile of pure MYC[1–84]-MeNbz-Arg$_6$. Rt 12.62 min. (Agilent Zorbax 300SB-C18 column, 5 µm, 2.1 × 150 mm, 5–95% MeCN over 20 min, *ca.* 4.5%B/min), 96% purity based on Area Under Curve (AUC) at λ = 214 nm.

8.2 MYC[85–143]-(D85C)-Arg$_6$

```
                10              20              30              40
H2N-CNDGGGGSFS    TADQLEMVTE    LLGGDMVNQS    FICDPDDETF

                50              60
    IKNIIIQDCM    WSGFSAAAKR RRRRR-CONH2                    (65 AA)
```

The peptide MYC[85–143] (D85C) bearing the [Arg(Pbf)]$_6$-Tag (ArgTag) on the C-terminus was synthesized on commercially available Novabiochem® NovaPEG Rink Amide resin (0.20 mmol/g, 0.15 g, 30 µmol) using the standard AFPS protocol (**Section 2.2.1**, 20 mL/min flowrate) (**SI Figure**

217). Total synthesis time to afford resin-bound MYC[85–143] (D85C)-[Arg(Pbf)]$_6$ (0.44 g) was approximately 3.5 h. Cleavage of half of the peptidyl-resin (0.22 g, approx. 15 µmol) according to Cleavage Protocol A (**Section 2.5.1**) afforded the crude peptide (34 mg, 34% purity by LCMS [**SI Figure 218**], 17% purity by UHPLC [**SI Figure 219**], monoisotopic mass calc. 7226.4100, found 7226.4173). The crude peptide (34 mg, approx. 17 µmol) was dissolved in GnHCl (6.0 M, 11 mL) and then purified by semi-prep RP-HPLC using an Agilent Eclipse XDB-C8 Semi-Preparative column (9.4 × 250 mm, 5 µm particle size) at a flow rate of 3.0 mL/min, with a two-step gradient of 5–30%B over 25 min (*ca.* 1%B/min), followed by 30–60%B over 120 min (*ca.* 0.25%B/min). Fractions were analyzed by LCMS and UHPLC, combined, and lyophilized to afford the *title compound* (0.50 mg, 92% purity by LCMS [**SI Figure 220**], >95% purity by UHPLC [**SI Figure 221**], 0.5% overall yield, monoisotopic mass calc. 7226.4100, found 7226.4012).

UV-Vis synthesis trace

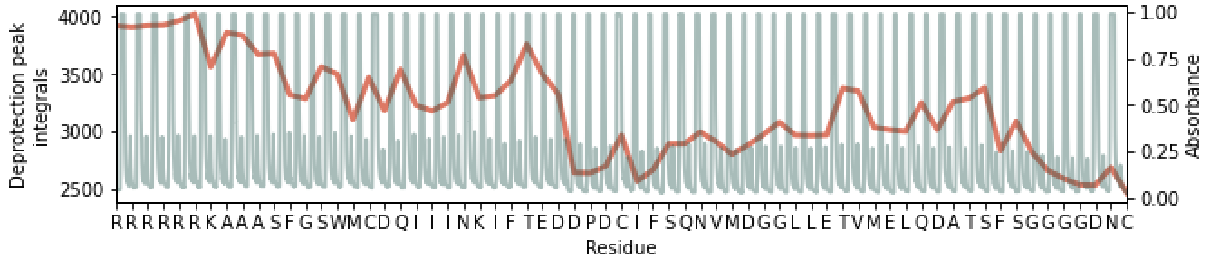

SI Figure 217. UV trace (λ = 310 nm) from AFPS of MYC[85–143] (D85C)-[Arg(Pbf)]$_6$ (green) and deprotection peak integrals (red).

LCMS of crude MYC[85–143]-(D85C)-(Arg)$_6$

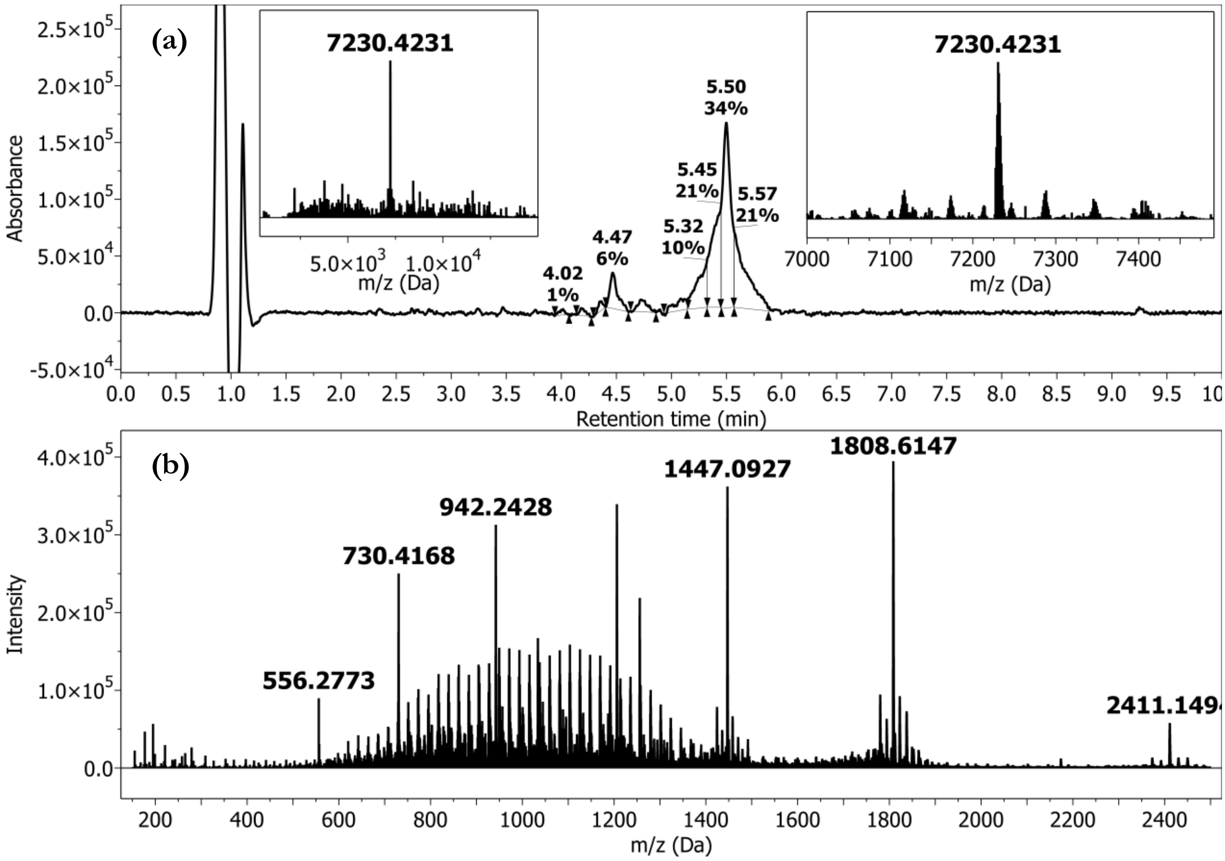

SI Figure 218. LCMS profile of crude MYC[85–143] (D85C)-Arg$_6$. (**a**) Absorbance chromatogram (λ = 214 nm); Rt 5.50 min. (**b**) ESI-TOF spectrum found within Rt 2–9 min, 34% purity. Inserts: deconvoluted masses. Monoisotopic mass (ESI+) calcd. for $C_{305}H_{485}N_{93}O_{99}S_6$ 7226.4100, found 7226.4173. LCMS Gradient A (**Section 2.7**).

UHPLC of crude MYC[85–143]-(D85C)-(Arg)$_6$

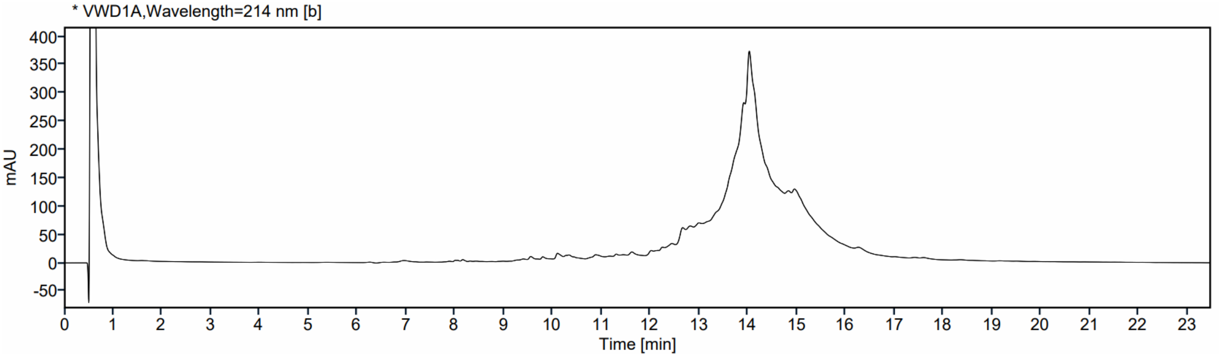

SI Figure 219. UHPLC profile of crude MYC[85–143]-(D85C)-Arg$_6$. Rt 14.03 min. (Agilent Zorbax 300SB-C18 column, 5 µm, 2.1 × 150 mm, 5–95% MeCN over 20 min, *ca.* 4.5%B/min), 17% purity based on Area Under Curve (AUC) at λ = 214 nm.

LCMS of pure MYC[85–143]-(D85C)-(Arg)$_6$

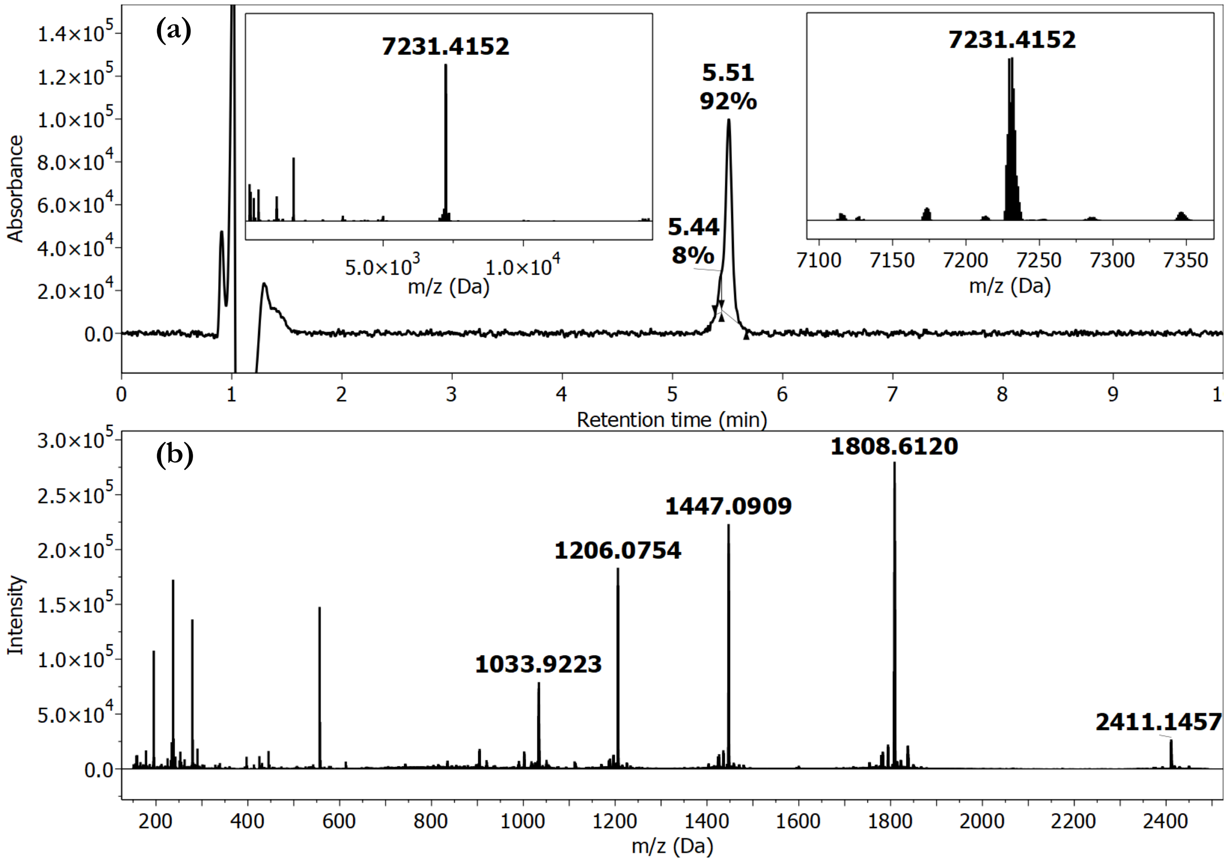

SI Figure 220. LCMS profile of pure MYC[85–143] (D85C)-Arg$_6$. (**a**) Absorbance chromatogram (λ = 214 nm); Rt 5.51 min. (**b**) ESI-TOF spectrum found within Rt 2–9 min, 92% purity. Inserts: deconvoluted masses. Monoisotopic mass (ESI+) calcd. for $C_{305}H_{485}N_{93}O_{99}S_6$ 7226.4100, found 7226.4012. LCMS Gradient A (**Section 2.7**).

UHPLC of pure MYC[85–143]-(D85C)-(Arg)$_6$

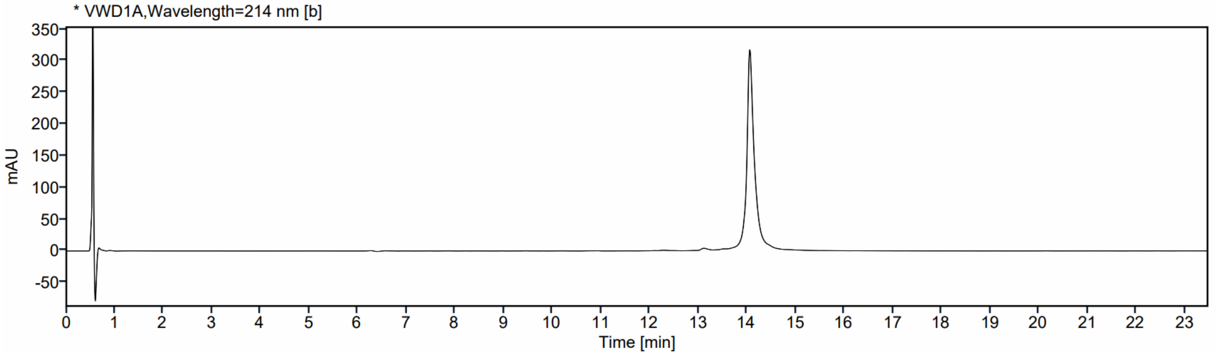

SI Figure 221. UHPLC profile of pure MYC[85–143]-(D85C)-Arg$_6$. Rt 14.06 min. (Agilent Zorbax 300SB-C18 column, 5 µm, 2.1 × 150 mm, 5–95% MeCN over 20 min, *ca.* 4.5%B/min), >95% purity based on Area Under Curve (AUC) at λ = 214 nm.

8.3 Native Chemical Ligation: MYC[1–143]-(D85C)-Arg$_6$

```
               10           20          30           40
   H2N-MPLNVSFTNR   NYDLDYDSVQ  PYFYCDEEEN   FYQQQQQSEL

               50           60          70           80
       QPPAPSEDIW   KKFELLPTPP  LSPSRRSGLC   SPSYVAVTPF

               90          100         110          120
       SLRGCNDGGG   GSFSTADQLE  MVTELLGGDM   VNQSFICDPD

              130          140         149
       DETFIKNIII   QDCMWSGFSA    AAKRRRRRR -CONH2            (149 AA)
```

Ligation Buffer: Sodium phosphate (200 mM) with guanidinium chloride (GnHCl, 6.0 M), pH 7.0.

MYC[1–84]-MeNbz-Arg$_6$ (0.75 mg, 69 µmol) and MYC[85–143] (D85C)-Arg$_6$ (0.49 mg, 66 µmol) were dissolved in degassed Ligation Buffer (33 µL) containing MPAA (0.10 M) and TCEP (20 mM). The solution was incubated at 37 °C with gentle agitation for 2 h, affording the crude peptide (39% purity by LCMS [**SI Figure 222**], 17% purity by UHPLC [**SI Figure 223**], monoisotopic mass calc. 16957.0277, found 16957.1976). Once the reaction was complete as indicated by UHPLC analysis, the solution was snap-frozen and stored at -80 °C. The crude material (30 µL) was then diluted with GnHCl (6.0 M, 120 µL) and purified by UHPLC using an Agilent Poroshell 300SB-C8 column (2.1 mm × 75 mm, 5 µm particle size) at a flow rate of 1.0 mL/min, with a gradient of 5–95%B over 30 min (*ca.* 3%B/min). Fractions were analyzed by UHPLC, combined, and lyophilized to afford the *title compound* (0.39 mg, 92% purity by LCMS [**SI Figure 224**], 96% purity by UHPLC [**SI Figure 225**], 35% isolated yield, monoisotopic mass calc. 16957.0277, found 16957.0462).

LCMS of crude MYC[1–143]-(D85C)-Arg_6

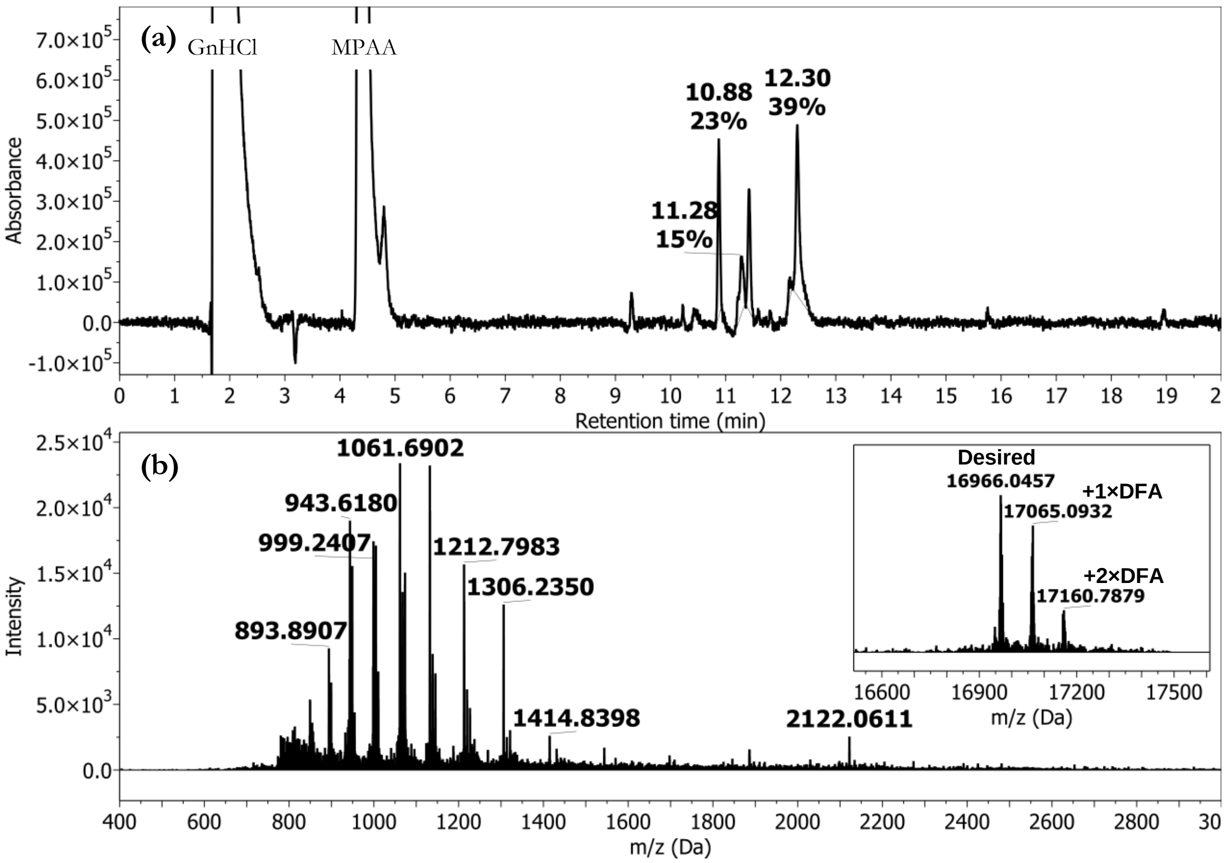

SI Figure 222. LCMS Profile of crude MYC[1–143]-(D85C)-$(Arg)_6$ after 2 h ligation. (**a**) Total absorbance chromatogram; Rt 12.30 min, 39% purity. (**b**) ESI-TOF spectrum found at Rt 12.38 min. Insert deconvoluted masses. Monoisotopic mass (ESI+) calcd. for $C_{744}H_{1131}N_{203}O_{235}S_9$ 16957.0277, found 16957.1976. The sample was injected into an ACQUITY UPLC BioResolve RP mAb Polyphenyl (2.1 mm × 150 mm, 2.7 µm particle size, 450 Å) column (Waters, USA) kept at 60 °C at a flow rate of 0.2 mL/min with UV detection at 214 nm. A binary solvent system was used, wherein Solvent A = 0.1% DFA in water, and Solvent B = 0.1% DFA in MeCN/*i*PrOH (1:4). A gradient of 0–100%B over 30 min was applied. Mass spectra were acquired using a Synapt G2-Si mass spectrometer in the positive-ion mode by scanning the m/z range from 400 to 5000 Da with a scan duration of 1 s and an interscan delay of 0.1 s. The spray voltage was set to 3 kV, the cone voltage to 50 V, and the source temperature to 100 °C. The data was recorded with the MassLynx 4.2 Software (Waters, UK). DFA = difluoroacetic acid.

UHPLC of crude MYC[1–143]-(D85C)-Arg_6

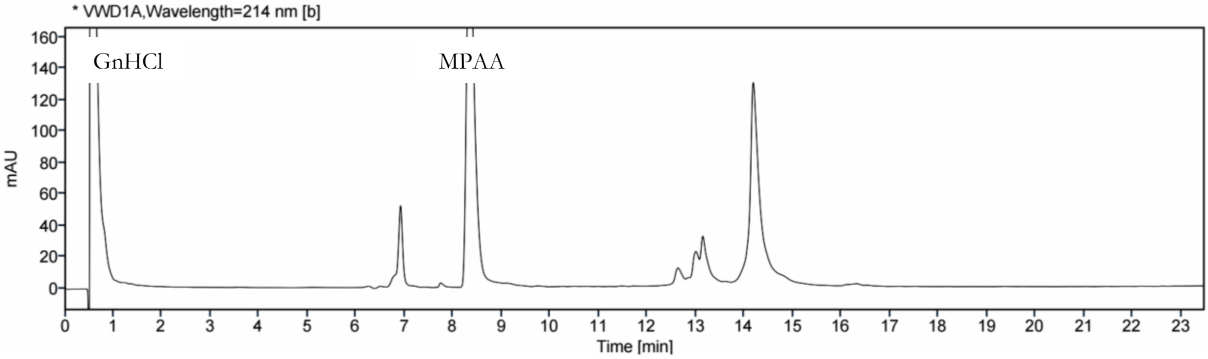

SI Figure 223. UHPLC profile of crude MYC[1–143]-(D85C)-Arg_6 after 2 h ligation. Rt 14.19 min. (Agilent Zorbax 300SB-C18 column, 5 µm, 2.1 × 150 mm, 5–95% MeCN over 20 min, *ca.* 4.5%B/min), 67% purity based on Area Under Curve (AUC) at λ = 214 nm.

LCMS of purified MYC[1–143]-(D85C)-Arg_6

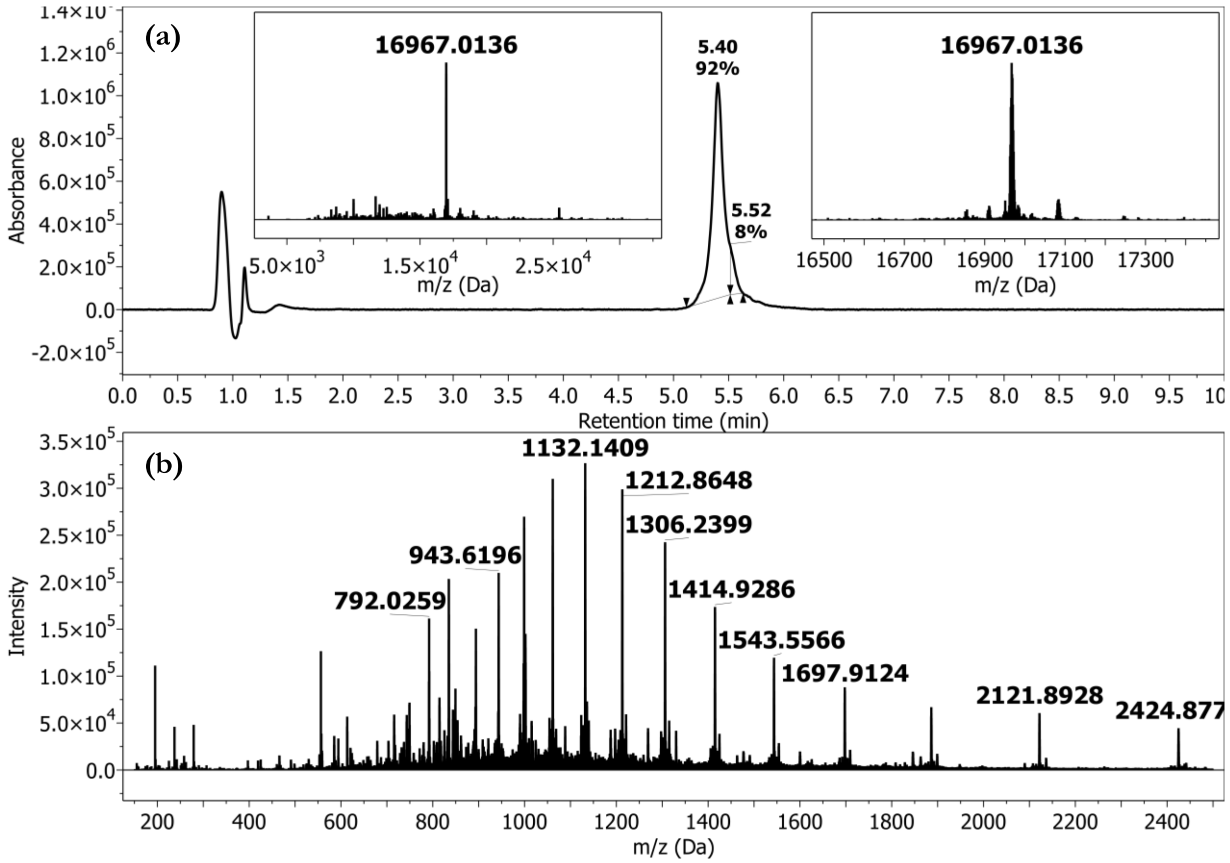

SI Figure 224. LCMS profile of purified MYC[1–143]-(D85C)-Arg_6. (**a**) Absorbance chromatogram (λ = 214 nm); Rt 5.40 min. (**b**) ESI-TOF spectrum found within Rt 2–9 min, 92% purity. Inserts: deconvoluted masses. Monoisotopic mass (ESI+) calcd. for $C_{744}H_{1131}N_{203}O_{235}S_9$ 16957.0277, found 16957.0462. LCMS Gradient A (**Section 2.7**).

UHPLC of pure MYC[1–143]-(D85C)-Arg_6

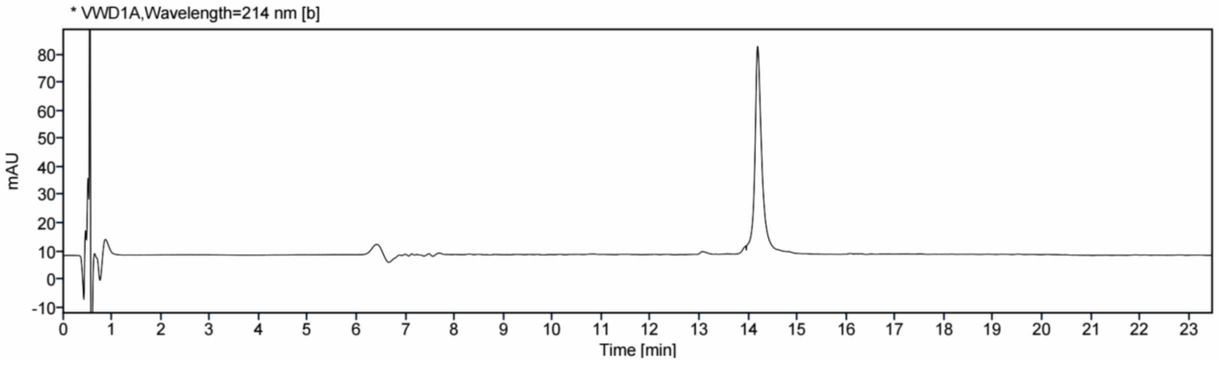

SI Figure 225. UHPLC profile of pure MYC[1–143]-(D85C)-Arg_6. Rt 14.19 min. (Agilent Zorbax 300SB-C18 column, 5 µm, 2.1 × 150 mm, 5–95% MeCN over 20 min, *ca.* 4.5%B/min), 96% purity based on Area Under Curve (AUC) at λ = 214 nm.

9 Appendix: IR Spectras

9.1 [Arg(Pbf)]$_9$ (photocleaved from resin)

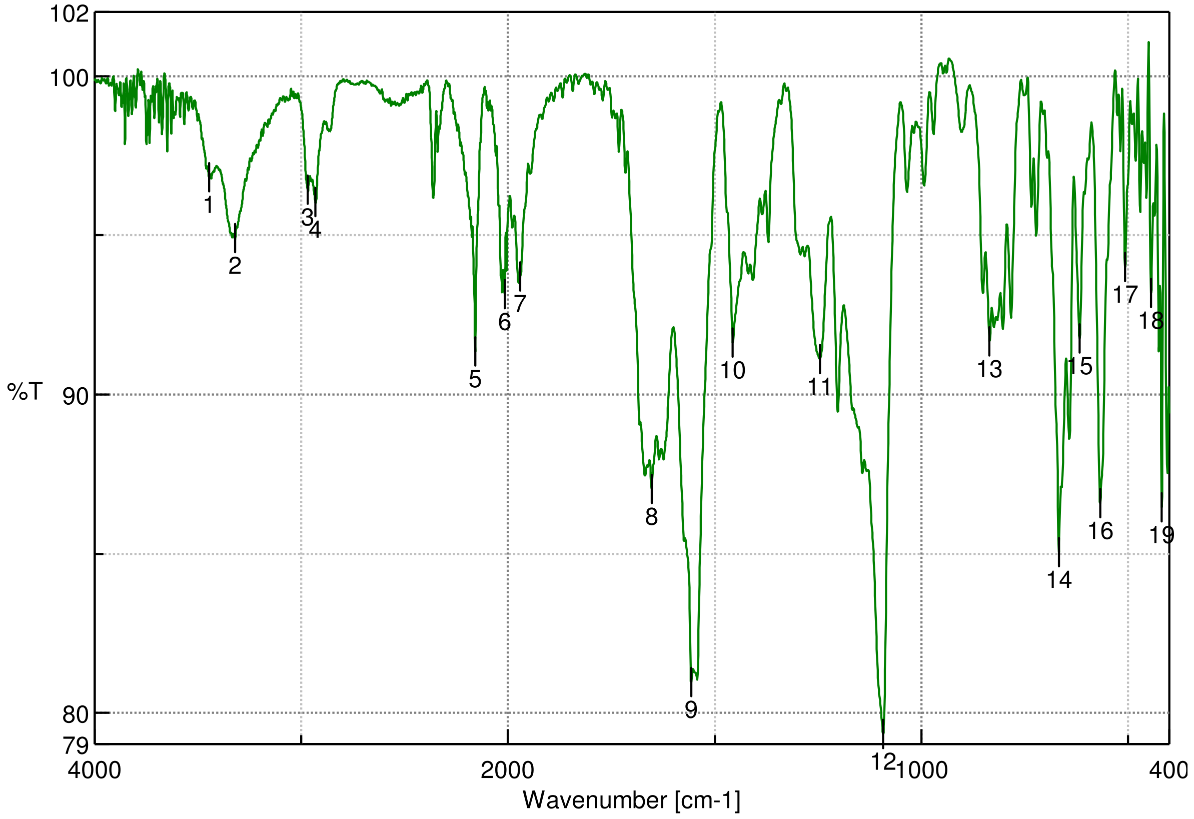

Result of Peak Picking

No.	Position	Intensity	No.	Position	Intensity
1	3445.2	96.8	2	3319.9	94.9
3	2969.8	96.4	4	2932.2	96.0
5	2158.0	91.3	6	2016.2	93.1
7	1969.9	93.7	8	1652.7	87.0
9	1556.3	80.9	10	1456.0	91.6
11	1245.8	91.1	12	1092.5	79.3
13	835.0	91.7	14	667.2	85.0
15	617.1	91.7	16	567.0	86.6
17	507.2	94.0	18	444.5	93.2
19	418.5	86.4			

[Comment]	
Sample Name	
Comment	
User	
Division	
Company	University of Zurich
[Measurement Information]	
Model Name	FT/IR-4100typeA
Serial Number	B079461016
Accessory	ATR PRO410-S
Accessory S/N	A042261044
Light Source	Standard
Detector	TGS
Accumulation	16
Resolution	4 cm-1
Zero Filling	On
Apodization	Cosine
Gain	Auto (128)
Aperture	Auto (7.1 mm)
Scanning Speed	Auto (2 mm/sec)
Filter	Auto (30000 Hz)

9.2 Resin-Bound [Arg(Pbf)]$_{15}$

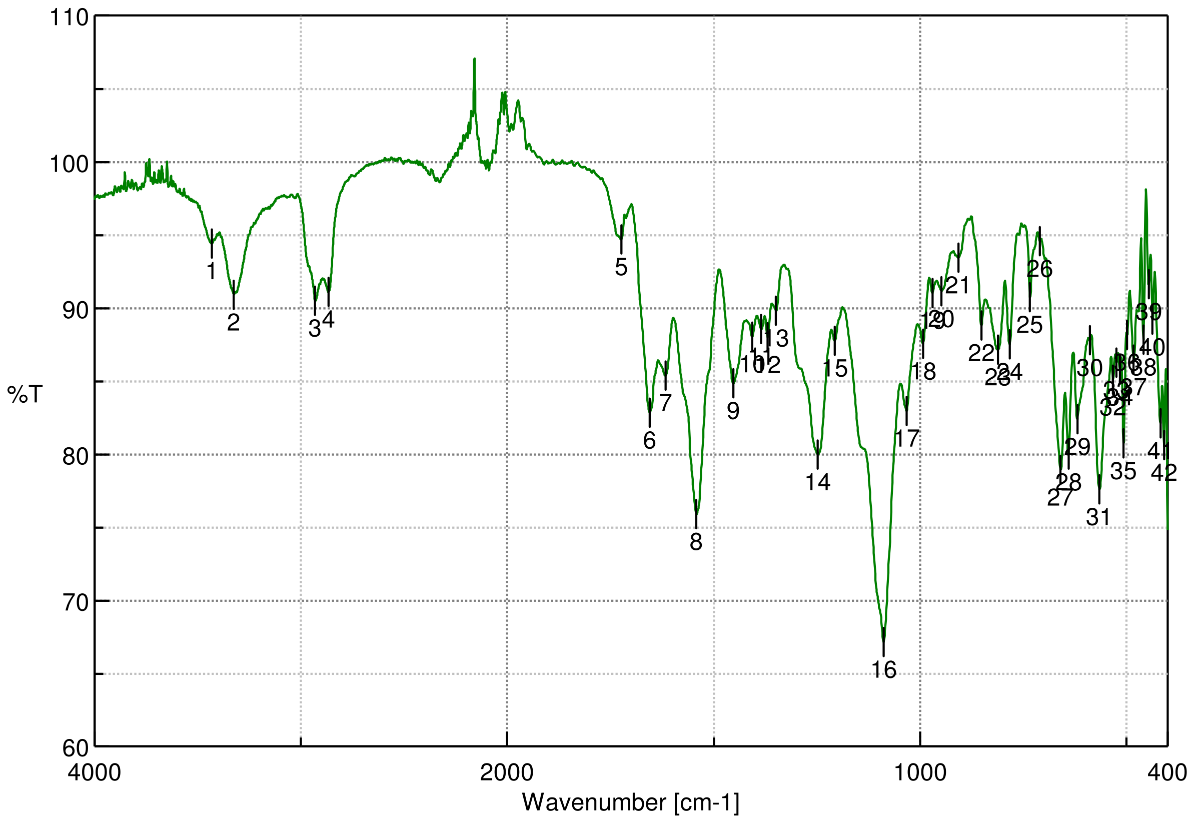

Result of Peak Picking

No.	Position	Intensity	No.	Position	Intensity
1	3432.7	94.4	2	3324.7	90.9
3	2931.3	90.5	4	2867.6	91.1
5	1724.0	94.7	6	1654.6	82.8
7	1617.0	85.4	8	1541.8	75.9
9	1452.1	84.8	10	1406.8	88.0
11	1384.6	88.6	12	1369.2	88.0
13	1349.0	89.8	14	1247.7	80.0
15	1207.2	87.7	16	1088.6	67.2
17	1032.7	83.0	18	993.2	87.6
19	970.0	91.0	20	947.8	91.1
21	907.3	93.4	22	851.4	88.8
23	811.9	87.1	24	783.0	87.5
25	733.8	90.7	26	709.7	94.6
27	659.5	78.9	28	640.3	80.0
29	619.0	82.4	30	589.1	87.8
31	565.0	77.6	32	532.3	85.1
33	523.6	86.3	34	515.9	85.8
35	507.2	80.7	36	498.5	88.2
37	482.1	86.5	38	459.0	87.8
39	445.5	91.6	40	436.8	89.2
41	417.5	82.1	42	408.8	80.6

[Comment]

Sample Name	
Comment	
User	
Division	
Company	University of Zurich

[Measurement Information]

Model Name	FT/IR-4100typeA
Serial Number	B079461016
Accessory	ATR PRO410-S
Accessory S/N	A042261044
Light Source	Standard
Detector	TGS
Accumulation	16
Resolution	4 cm-1
Zero Filling	On
Apodization	Cosine
Gain	Auto (128)
Aperture	Auto (7.1 mm)
Scanning Speed	Auto (2 mm/sec)
Filter	Auto (30000 Hz)

9.3 Resin-Bound Barstar[75–90]

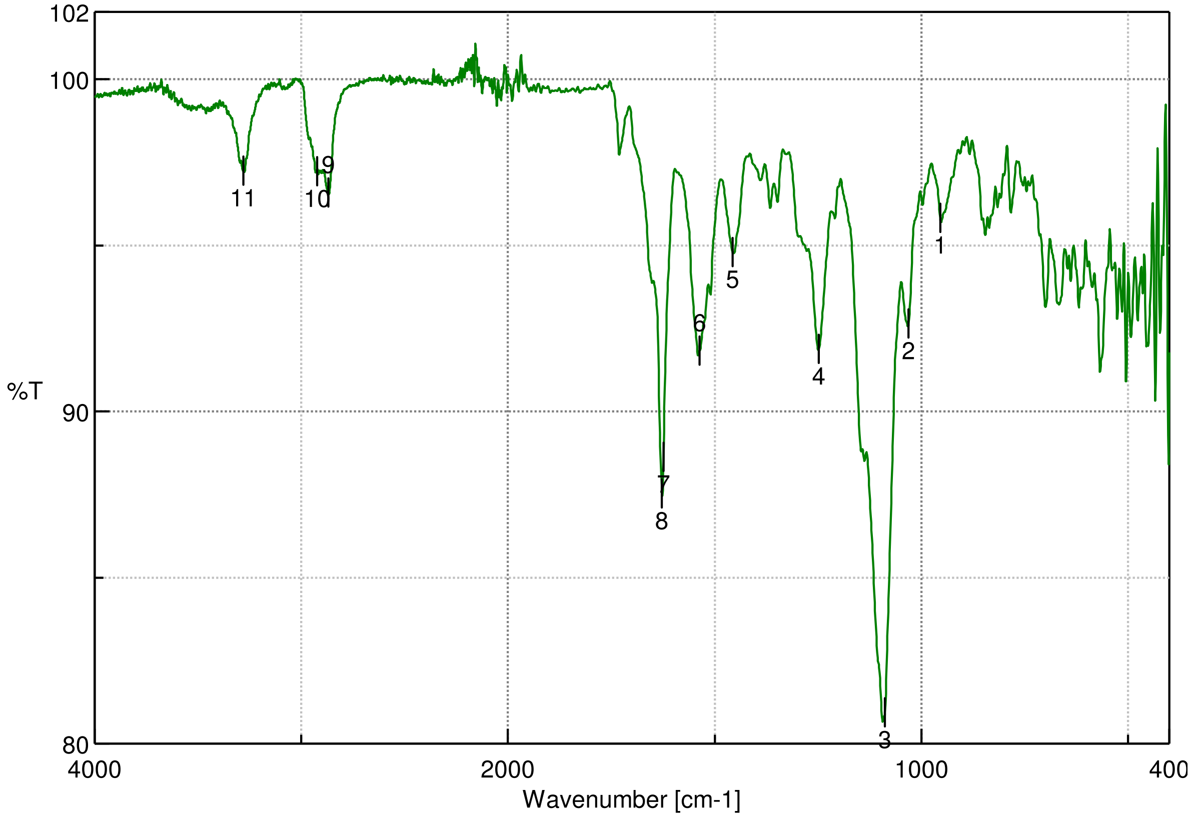

Result of Peak Picking

No.	Position	Intensity	No.	Position	Intensity
1	954.6	95.8	2	1030.8	92.6
3	1087.7	80.9	4	1247.7	91.9
5	1457.0	94.8	6	1537.0	91.8
7	1623.8	88.6	8	1627.6	87.5
9	2870.5	96.6	10	2924.5	97.2
11	3281.3	97.2			

[Comment]

Sample Name	
Comment	
User	
Division	
Company	University of Zurich

[Measurement Information]

Model Name	FT/IR-4100typeA
Serial Number	B079461016
Accessory	ATR PRO410-S
Accessory S/N	A042261044
Light Source	Standard
Detector	TGS
Accumulation	16
Resolution	4 cm-1
Zero Filling	On
Apodization	Cosine
Gain	Auto (64)
Aperture	Auto (7.1 mm)
Scanning Speed	Auto (2 mm/sec)
Filter	Auto (30000 Hz)

9.4 Resin-Bound Barstar[75–90]-[Arg(Pbf)]$_6$

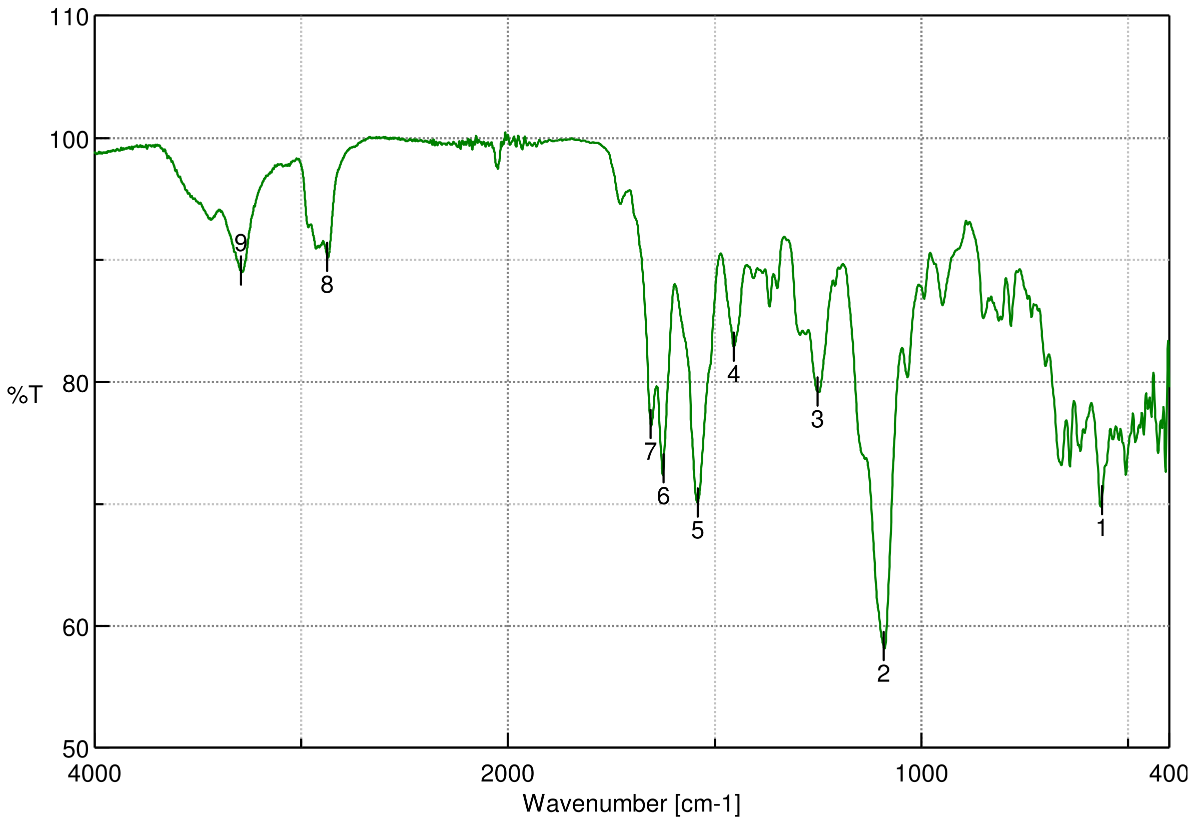

Result of Peak Picking

No.	Position	Intensity	No.	Position	Intensity
1	563.1	70.3	2	1091.5	58.3
3	1251.6	79.2	4	1453.1	82.9
5	1540.8	70.1	6	1623.8	72.9
7	1654.6	76.5	8	2874.4	90.2
9	3292.9	89.1			

[Comment]	
Sample Name	
Comment	
User	
Division	
Company	University of Zurich

[Measurement Information]	
Model Name	FT/IR-4100typeA
Serial Number	B079461016
Accessory	ATR PRO410-S
Accessory S/N	A042261044
Light Source	Standard
Detector	TGS
Accumulation	16
Resolution	4 cm-1
Zero Filling	On
Apodization	Cosine
Gain	Auto (64)
Aperture	Auto (7.1 mm)
Scanning Speed	Auto (2 mm/sec)
Filter	Auto (30000 Hz)
